# Enantioselective Synthesis of Fluorinated Indolizidinone Derivatives

**DOI:** 10.1021/acs.orglett.3c00903

**Published:** 2023-05-01

**Authors:** Marcos Escolano, Daniel Gaviña, Santiago Díaz-Oltra, María Sánchez-Roselló, Carlos del Pozo

**Affiliations:** Department of Organic Chemistry, University of Valencia, Avda Vicente Andrés Estellés s/n, 46100 Burjassot, Valencia, Spain

## Abstract

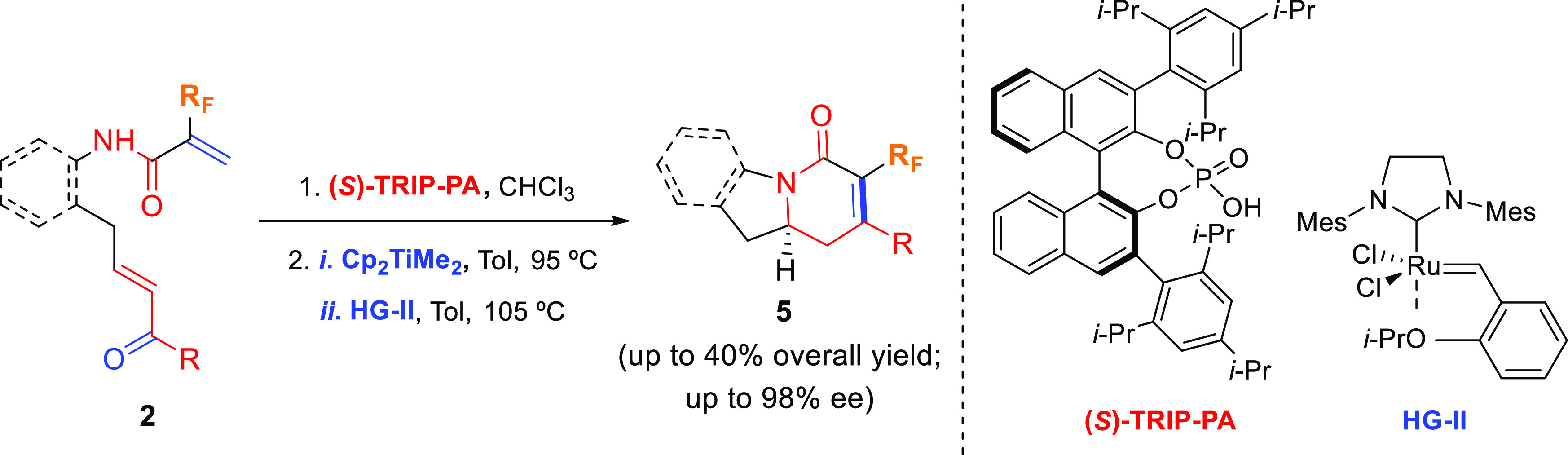

The enantioselective synthesis of fluorinated indolizidinone derivatives has been developed. The process involved an enantioselective intramolecular aza-Michael reaction of conjugated amides bearing a pendant α,β-unsaturated ketone moiety, catalyzed by the (*S*)-TRIP-derived phosphoric acid, followed by dimethyltitanocene methylenation and ring closing metathesis (RCM). Final indolizidine-derived products comprise a fluorine-containing tetrasubstituted double bond generated by the RCM reaction, which is a challenging task. The whole synthetic sequence took place in acceptable overall yields with excellent enantioselectivities.

The indolizidine scaffold is a widespread motif in a broad variety of alkaloids arising from extremely diverse natural sources. This type of chemical skeleton belongs to the *izidine* family, which constitutes ∼30% of the known alkaloids. Natural and non-natural indolizidines display a wide range of biological activities,^1^ and their pharmacological potential has made them privileged structures in medicinal chemistry research. The development of methodologies that gain access to those skeletons, especially in an asymmetric manner, is highly desirable and has attracted considerable attention from the synthetic chemistry community.^2^

The benefits of introducing fluorine atoms into organic molecules have been well recognized in the field of medicinal chemistry.^3^ Thus, fluorine substituents usually improve membrane permeability, metabolic pathways, and pharmacokinetic properties of the parent nonfluorinated molecules. As a consequence, fluorine-containing drugs account for >20% of all pharmaceuticals currently marketed.^4^ Moreover, some of the so-called blockbuster drugs contain fluorine atoms in their structures.^5^ Despite the abundance of fluorine in the Earth’s crust, its presence in biological systems is minimal and fluoroorganic compounds are mostly man-made. A wide variety of reagents for the selective introduction of fluorine or fluoroalkyl groups into specific locations of organic molecules have been devised, providing the chemistry community with the toolbox for the synthesis of tailor-made fluoroorganic derivatives. In the context of indolizidine scaffolds, despite their biological relevance, those bearing fluorinated substituents are very scarce.^6^

The first asymmetric synthesis of trifluoromethylated indolizidines was reported in 2002 by Okano and co-workers through a diastereoselective radical cyclization.^7^ Subsequently, the group of Kim performed an asymmetric synthesis of trifluoromethyl monomorine using a diastereoselective hydrogenation of a chiral fluorinated oxazoline as the key step.^8^ The synthesis of *gem*-difluoromethylene-containing indolizidines was carried out by Pohmakotr and co-workers by means of a fluoride-catalyzed nucleophilic addition of PhSCF_2_SiMe_3_ to chiral imides^9^ and nitrones,^10^ followed by intramolecular radical cyclization. In 2014, the group of Haufe reported the synthesis of a monofluorinated indolizidine from (*S*)-*N*-Boc-2-allyl pyrrolidine using a ring closing metathesis (RCM) reaction.^11^ Finally, Cordero and Brandi synthesized fluorinated analogues of the 1,2-dihydroxyindolizidine natural product lentiginosine.^12^

To date, all of the reported methodologies for accessing chiral nonracemic fluorinated indolizidines are based on either chiral pool or chiral auxiliary strategies. Alternatively, we devised a highly convenient enantioselective synthetic strategy that provides entry to enantiomerically enriched fluorinated indolizidinones in a three-step sequence. In the approach we described here, the six-membered ring was disconnected at the double bond by means of a RCM reaction on diolefinic pyrrolidines **4**, in turn prepared from chiral pyrrolidines **3** through carbonyl olefination. The chiral center was settled by an enantioselective intramolecular aza-Michael reaction (IMAMR) on conjugated amides **2** bearing a remote α,β-unsaturated ketone moiety (Scheme 1).

**Scheme 1:**
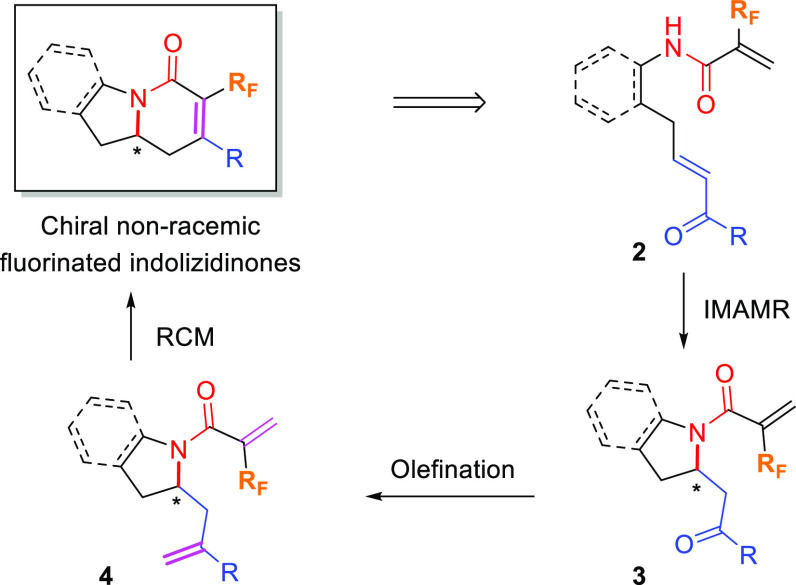
Strategy Devised for the Enantioselective Synthesis of Fluorinated Indolizidines

The starting fluorinated conjugated amides **2**, conveniently functionalized to carry out the intended synthetic strategy, were obtained by means of a selective cross-metathesis reaction between fluorinated amides **1** and conjugated ketones **6** in the presence of a Hoveyda–Grubbs second-generation catalyst (**HG-II**). Compounds **1** were in turn prepared from reduction of the corresponding nitriles followed by coupling with 2-fluoroacrylic acid or 2-(trifluoromethyl)acryloyl chloride (Scheme 2; see the Supporting Information for details).

**Scheme 2:**
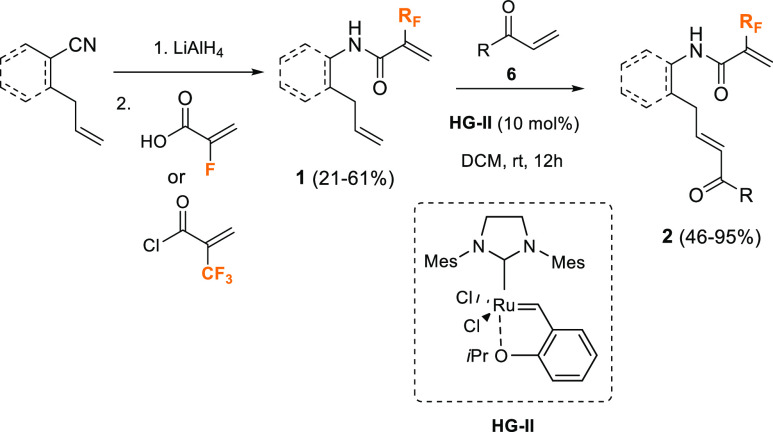
Synthesis of Fluorinated Conjugated Amides **2**

The feasibility of the proposed synthetic methodology was explored with α-fluoroacrylamide **2a** as a model substrate. The results of the optimization of the enantioselective intramolecular aza-Michael reaction (IMAMR) are summarized in Table 1. Initially, the reaction was evaluated with hydroquinine-derived primary amine **I** and trifluoroacetic acid as a co-catalyst, because this catalytic system usually provides good results for conjugated additions to enones^13^ (Table 1, entry 1); however, the reaction did not proceed, probably due to the low nucleophilicity of amides. Then, we moved to Brønsted acid catalysis, because previous results from our group had indicated that chiral BINOL-derived phosphoric acids (CPAs) were suitable catalysts for the IMAMR when amides were used as nitrogen sources.^14,15^

**Table 1:**
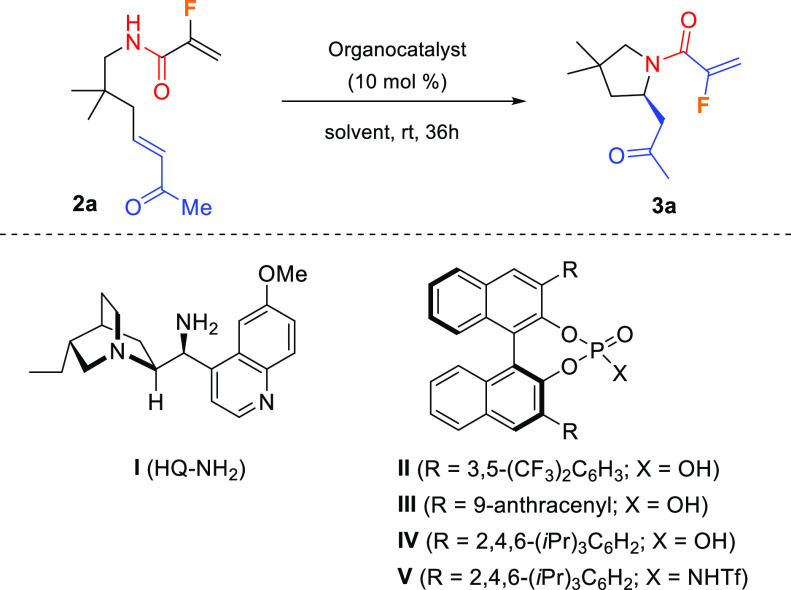
Optimization of the Enantioselective Cyclization of Substrate **2a** by an Intramolecular Aza-Michael Reaction^a^

entry	catalyst	solvent	yield^b^ (%)	ee (%)^c^
1	**I**/TFA	CHCl_3_	–	–
2	**II**	CHCl_3_	79	23
3	**III**	CHCl_3_	99	32
4	**IV**	CHCl_3_	86	95
5	**IV**	CHCl_3_	87	94^d^
6	**IV**	toluene	90	92
7	**IV**	THF	13	98
8	**V**	CHCl_3_	82	33

a Reactions were performed with **2a** (0.2 mmol) and a catalyst (10 mol %) in a dry solvent.

b Isolated yields after flash column chromatography.

c Enantiomeric ratios were determined by HPLC analysis on a chiral stationary phase (see the Supporting Information for details).

d Reaction performed at 60 °C.

Therefore, CPA **II** was tested, and the reaction afforded chiral pyrrolidine **3a** in 79% isolated yield but with a low enantiomeric excess (23%) (Table 1, entry 2). Changing the catalyst to anthracenyl-derived CPA **III** provided an excellent chemical yield of product **3a**, although, again, poor enantiocontrol (32% ee) (Table 1, entry 3). Fortunately, with (*S*)-TRIP-derived catalyst **IV**, the cyclization reaction took place in good yield (86%) with excellent enantioselectivity (95% ee) at room temperature (Table 1, entry 4). When the reaction was performed at 60 °C, comparable results were obtained in terms of yield and enantiocontrol (Table 1, entry 5), while the use of other solvents such as toluene or THF was detrimental with respect to the enantioselectivity or the chemical yield (Table 1, entry 6 or 7, respectively). Finally, when the more acidic triflimide **V** was employed, a remarkable decrease in the enantioselectivity was observed (Table 1, entry 8). In light of these results, the optimal conditions for the IMAMR of α-fluoroacrylamide **2a** involved its treatment with (*S*)-TRIP-PA **IV** (10 mol %) as the catalyst in chloroform at room temperature (Table 1, entry 4).

These reaction conditions were further extended to other starting fluorinated amides **2**, and the results are summarized in Scheme 3.

**Scheme 3:**
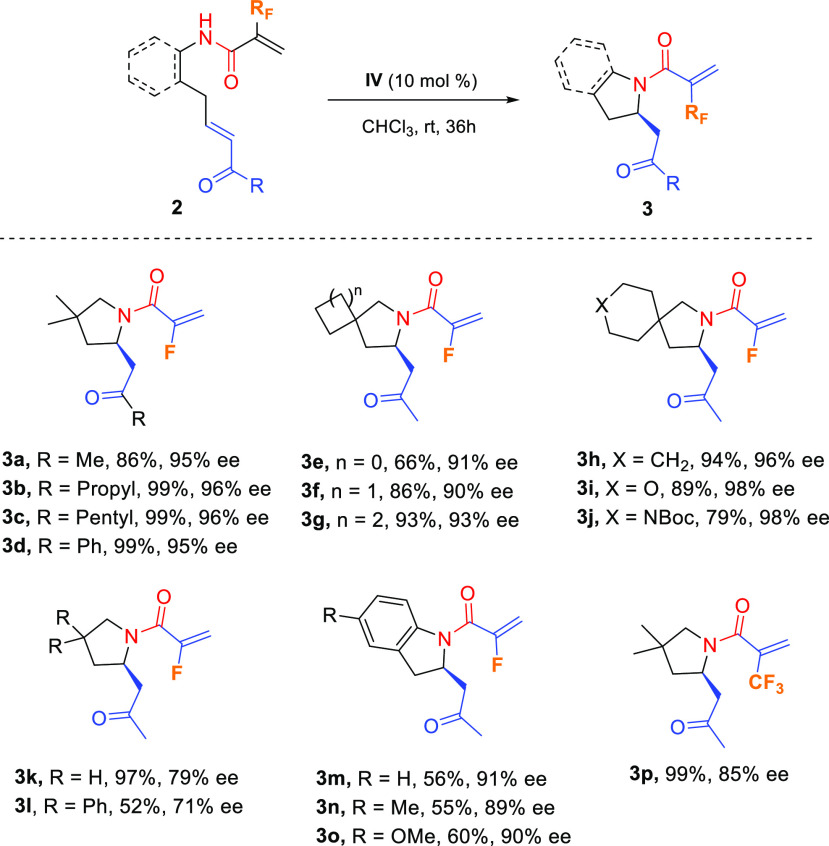
Scope of the Enantioselective Intramolecular Aza-Michael Reaction– Unless otherwise noted, reactions were carried out with **2** (0.2–0.5 mmol) and catalyst **IV** (10 mol %) in chloroform (2 mL) at room temperature for 36 h. Isolated yields after flash column chromatography. Enantiomeric ratios were determined by HPLC analysis on a chiral stationary phase (see the Supporting Information for details).

With regard to the vinyl ketone moiety, both aliphatic (**2a**–**c**) and aromatic (**2d**) conjugated ketones readily underwent the IMAMR, providing the corresponding fluorinated 4,4-dimethyl pyrrolidines **3a**–**d**, respectively, in excellent yields and enantioselectivities. Other modifications of the starting fluorinated conjugated amides **2** were also possible. Thus, pyrrolidines (**3e**–**j**) bearing spirocyclic moieties at position 4 such as cyclopropyl, cyclobutyl, cyclopentyl, cyclohexyl, tetrahydropyranyl, and *N*-Boc piperidinyl were obtained very efficiently. Likewise, 4-unsubstituted pyrrolidine **3k** and 4,4-diphenyl derivative **3l** were obtained in excellent (97%) and moderate (52%) yield, respectively, albeit with diminished enantioselectivity (79% and 71% ee, respectively). Benzo-fused substrates were also tolerated in this enantioselective cyclization, rendering 2-substituted indoline derivatives **3m**–**o** in moderate isolated yields and good ee values. Finally, α-trifluoromethyl acrylamide **2p** gave rise to the corresponding trifluoromethylated pyrrolidine **3p** in excellent yield and 85% ee (Scheme 3).

The absolute configuration of the newly created stereocenter in the IMAMR was assigned by X-ray analysis. Crystals of pyrrolidine **3p** suitable for single-crystal X-ray diffraction displayed the *R* absolute configuration,^16^ and an identical stereochemical outcome was assumed for all other pyrrolidines **3**.

With enantiomerically enriched fluorinated pyrrolidines **3** in hand, the next step of our study was the methylenation of the ketone carbonyl to subsequently effect the RCM reaction that would complete the indolizidine skeleton. The first attempt was made with compound **3a** as the model substrate and involved its treatment with Wittig ylide Ph_3_P=CH_2_; however, no reaction took place, but racemic substrate (±)-**3a** was isolated instead of the expected methylenation product (Scheme 4, eq 1). This racemization event could be explained considering that the IMAMR is a reversible process. The Wittig ylide would act as a base, promoting the retro-Michael/Michael sequence that would afford racemic product (±)-**3a**.

**Scheme 4:**
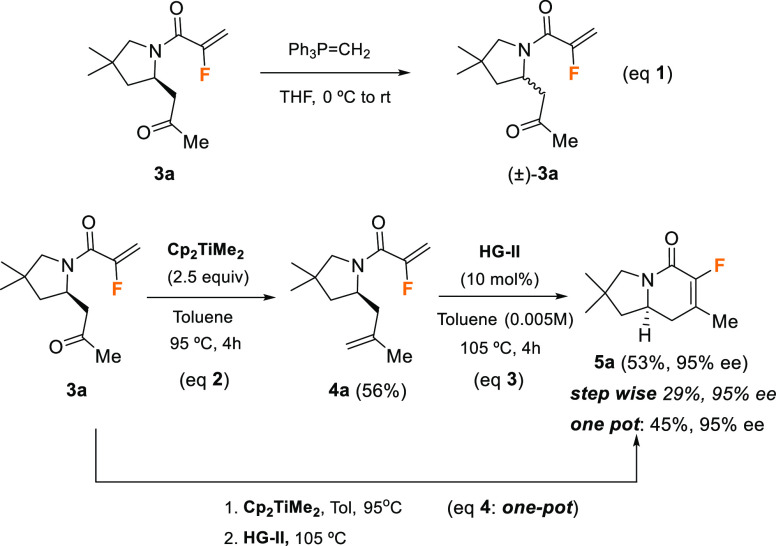
Methylenation/RCM Sequence on Fluorinated Pyrrolidine **3a**

To avoid this disappointing result, we consider the use of dimethyltitanocene^17^ (Cp_2_TiMe_2_, Petasis reagent) as an alternative to perform the desired carbonyl methylenation. This reagent would work by heating in toluene in the absence of a base, which would prevent the undesired retro-aza-Michael reaction, with the subsequent racemization of the chiral center. Therefore, this reaction was optimized (see the Supporting Information for details), and we found that, after compound **3a** had been heated with 2.5 equiv of Petasis reagent in toluene at 95 °C for 4 h, methylenation product **4a** was obtained in 56% isolated yield (Scheme 4, eq 2). Then, this compound was subjected to the RCM reaction, and after some optimization of the conditions (see the Supporting Information for details), we were able to isolate monofluorinated indolizidinone derivative **5a** in a noticeable 53% yield by heating compound **4a** in a highly diluted solution of toluene (0.005 M) at 105 °C for 48 h in the presence of the Hoveyda–Grubbs second-generation catalyst (**HG-II**) (Scheme 4, eq 3). It is important to mention that this RCM reaction generated a fluorine-containing tetrasubstituted double bond, which is a quite challenging task, given that cyclizations through RCM are very sensitive to steric issues.

On the contrary, during the purification of pyrrolidine **4a**, we observed that it was a volatile and unstable compound. For this reason, and to improve the efficiency of the process, we decided to evaluate the sequence of methylenation/RCM in a one-pot manner. Thus, when the reaction of **4a** with Petasis reagent had reached completion (determined by TLC analysis of the crude reaction mixture), titanium salts were filtered off and the resulting residue was directly treated with the **HG-II** catalyst and heated for 48 h at 105 °C. Under these conditions, the desired indolizidine derivative **5a** was isolated in 45% overall yield, indicating that the one-pot procedure is more efficient than the stepwise protocol (Scheme 4, eq 4). Importantly, chiral HPLC analysis of product **5a** (95% ee) showed no erosion of the stereochemical integrity of the stereocenter generated during the organocatalytic IMAMR step.

The optimized conditions for the one-pot sequence methylenation/RCM reaction were then applied to the rest of chiral pyrrolidines **3** (Scheme 5). While substrate **3a** bearing a methyl ketone rendered indolizidine derivative **5a** in 45% overall yield, the analogous propyl and pentyl ketones rendered the corresponding fluorinated products **5b** and **5c** in 25% and 24% yields, respectively, indicating that the increase in the steric requirements made the RCM reaction more difficult. In this context, aromatic ketone **3d** led to compound **5d** in trace amounts. Spirocyclic substrates **3e**–**j** allowed the synthesis of indolizidine derivatives **5e**–**j**, respectively, in valuable yields ranging from 33% to 51%. Likewise, the nonsubstituted substrate with five-membered ring **3k** and *gem*-diphenyl compound **3l** provided the corresponding indolizidinones **5k** and **5l**, respectively, in moderate yields (30% and 33%, respectively). Benzo-fused derivatives **5m**–**o** were also successfully obtained in comparable yields (31–42%), as well as trifluoromethyl compound **5p** (40% yield). In all cases, the stereochemical integrity of the carbon stereocenter was preserved and no erosion of enantiopurity was detected (Scheme 5).

**Scheme 5:**
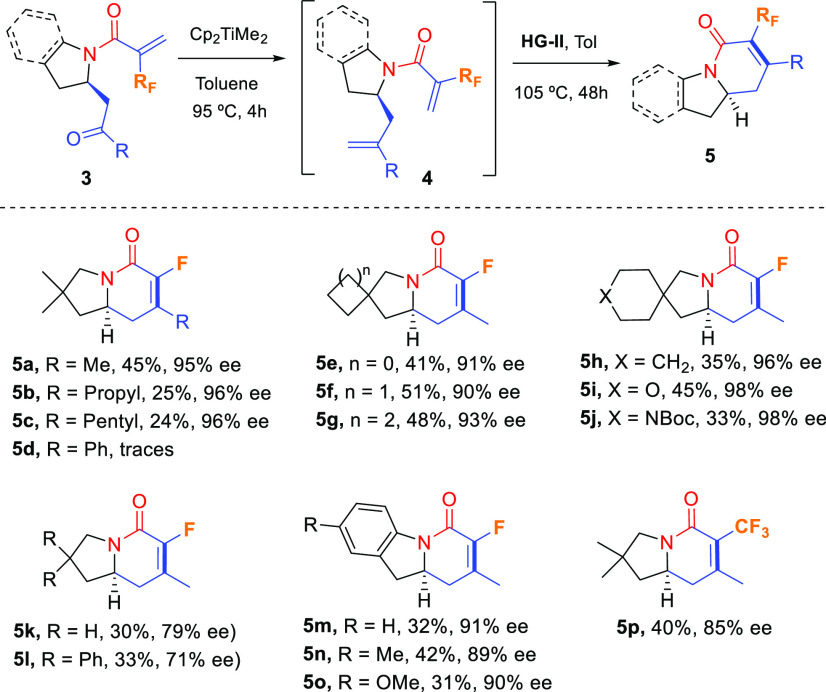
Synthesis of Fluorinated Indolizidine Derivatives **5** by the Methylenation/RCM Sequence– Reactions were carried out with **3** (0.1–0.3 mmol) and Cp_2_TiMe_2_ (2.5 equiv) in toluene (0.1 M) at 95 °C for 4 h. RCM was performed with **HG-II** (10 mol %) in toluene (0.005 M) at 105 °C for 48 h. Isolated yields after flash column chromatography. Enantiomeric ratios were determined by HPLC analysis on a chiral stationary phase (see the Supporting Information for details).

The whole synthesis of fluorinated indolizidinone **5a** was tested on a multigram scale. Thus, starting from 1.48 g of fluorinated amide **1a** (8 mmol), 540 mg of indolizidinone **5a** was obtained; i.e., this product was formed in 34% overall yield in four reaction steps without erosion of the ee value.

Finally, compound (±)-**5a** was derivatized to the corresponding indolizidinone through hydrogenation of the double bond. Treatment of (±)-**5a** with Pd/C in ethyl acetate under a hydrogen atmosphere for 24 h afforded indolizidinone (±)-**7** in 66% yield as a single diastereoisomer (Scheme 6). The relative disposition of the newly created stereocenters was determined by X-ray analysis.^18^

**Scheme 6:**
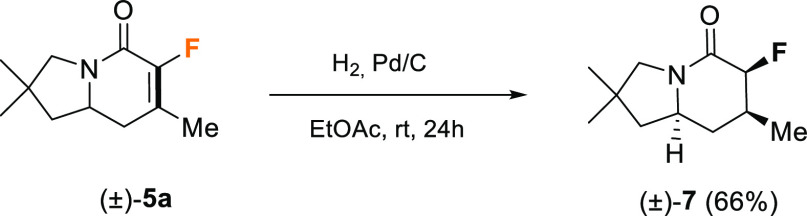
Hydrogenation of Compound (±)-**5a**

In conclusion, the enantioselective organocatalytic synthesis of a family of fluorinated indolizidinone derivatives has been described. Conjugated fluorinated amides **1** bearing a pendant olefin were subjected to the CM/IMAMR/methylenation/RCM sequence to render imidazolidinone derivatives **5** in synthetically useful overall yields and good enantioselectivities. A chiral sterocenter was efficiently generated with the participation of (*S*)-TRIP-derived phosphoric acid as the catalyst. Then, carbonyl methylenation with Petasis reagent followed by RCM with the Hoveyda–Grubbs second-generation catalyst occurred without erosion of the stereochemical integrity of the chiral center after both processes.

## Data Availability

The data underlying this study are available in the published article and its Supporting Information.

## Supporting Information Available

Experimental procedures, mechanistic studies, and characterization and NMR spectra of new compounds (PDF)
FAIR data, including the primary NMR FID files, for compounds **1a–m**, **2a–p**, **3a–p**, **4a**, **5a–c**, **5e–p**, and **7** (ZIP)

## Dedication

Dedicated to Prof. Dennis Curran on the occasion of his 70th birthday.
