# Supplementary material for: Enantioselective Synthesis of Fluorinated Indolizidinone Derivatives
Source: Org Lett. 2023 May 1;25(18):3222–7. doi: 10.1021/acs.orglett.3c00903 (PMC10186376; doi:10.1021/acs.orglett.3c00903)
Supplement: Supplementary file 1 — ol3c00903_si_001.pdf [file ol3c00903_si_001.pdf]

SUPPORTING INFORMATION

Enantioselective Synthesis of Fluorinated Indolizidinone Derivatives.

Marcos Escolano,[a] Daniel Gavina,[a] Santiago Díaz-Oltra,[a] María Sánchez-Roselló*,[a] and Carlos del Pozo*,[a]

[a] Departamento de Química Orgánica, Universidad de Valencia, 46100 Burjassot, España

Fax: (+34)963544939: e-mail: carlos.pozo@uv.es; maria.sanchez-rosello@uv.es

Contents

General Remarks S2

Cross metathesis (CM) reaction results of substrates **1** S3

Optimization of the enantioselective IMAMR S4

Optimization methylenation with Petasis reagent of **3a** S5

Optimization ring closing metathesis (RCM) of **4** S6

General procedure for the synthesis of fluoroacrylamides **1a-i** S7

General procedure for the synthesis of fluoroacrylamides **1j-l** S12

Synthesis of *N*-(2,2-dimethylpent-4-en-1-yl)-2-trifluoromethyl(acrylamide) **1m** S14

General procedure for the cross-metathesis reaction. Synthesis of enones **2** S15

General procedure for the intramolecular aza-Michael reaction.

Synthesis of pyrrolidines **3** S24

General procedure for the synthesis of indolizidines **5** S34

Scaled process example S44

Synthesis of (±)-**7** S45

X-Ray structure of compounds **3p** and **7** S46

HPLC traces of enantioenriched compounds **3** and **5** S49

NMR spectra of new compounds S80

GENERAL REMARKS

Reactions involving moisture-sensitive chemicals were carried out in flame-dried glassware with magnetic stirring under nitrogen atmosphere. The following solvents were purified prior to use: THF, diethyl ether and toluene were distilled from sodium/benzophenone, CH_2Cl_2 was distilled from calcium hydride. All other solvents and reagents were used as received. Catalysts (*S*)-TRIP-PA and HG-II were also purchased from commercial sources.The reactions were monitored with the aid of thin-layer chromatography (TLC) on 0.25 mm precoated silica gel plates. Visualization was carried out with UV light and aqueous ceric ammonium molybdate solution, potassium permanganate or vanillin stains. Flash column chromatography was performed with the indicated solvents on silica gel 60 (particle size 0.040-0.063 mm). ^{1}H, ^{13}C and ^{19}F NMR spectra were recorded on a 300 or 500 MHz spectrometer. Chemical shifts are given in ppm (δ), with reference to the residual proton resonances of the solvents. ^{19}F NMR spectra are referenced relative to $CFCl_3$ using the Bruker internal referencing procedure. Coupling constants (*J*) are given in Hertz (Hz). The letters m, s, d, t, and q stand for multiplet, singlet, doublet, triplet and quartet, respectively. The letter b indicate that the signal is broad. High-resolution mass spectra were carried out on VGmAutospec (VG Analytical, Micromass Instruments) by the Universidad de Valencia Mass Spectrometry Service. Enantiomeric ratios were determined with the aid of HPLC analysis with an appropriate chiral column (25 cm x 0.46 cm) with mixtures of n-hexane: *i*-propanol as eluents. The starting allyl nitriles **8a-i**[1] and anilines **8j-l**[2] were synthesized following literature procedures. Second generation Hoveyda-Grubbs catalyst and (*S*)-TRIP-phosphoric acid were commercially available.

[1] Chen, C.; Hou, C.; Chen, P.; Liu, G. Palladium(II)-Catalyzed Aminotrifluoromethoxylation of Alkenes: Mechanistic Insight into the Effect of N-Protecting Groups. *Chin. J. Chem*. **2020**, *38*, 346.
[2] Bovino, M. T.; Chemler, S. R. Catalytic Enantioselective Alkene Aminohalogenation/Cyclization Involving Atom Transfer. *Angew. Chem., Int. Ed*. **2012**, *51*, 3923.

CROSS METATHESIS (CM) RESULTS OF SUBSTRATES 1:

The results of the CM reaction of substrates **1** are summarized in Table S1.

Table S1 Cross metathesis reaction of substrates **1**

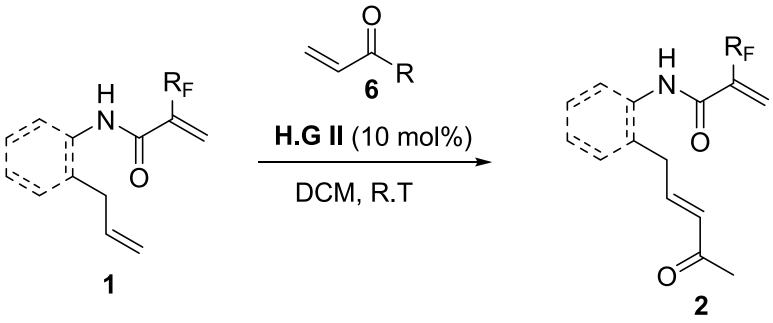

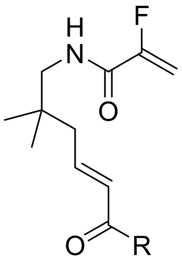

2a (R = Me, 90%)
2b (R = Propyl, 95%)
2c (R = Pentyl, 92%)
2d (R = Ph, 67%)

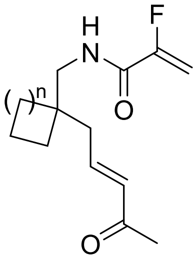

2e (n = 0, 89%)
2f (n = 1, 84%)
2g (n = 2, 90%)

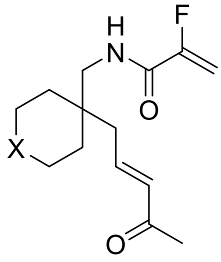

2h (X = CH_2, 75%)
2i (X = O, 76%)
2j (X = NBoc, 69%)

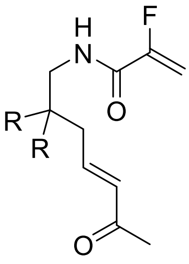

2k (R = H, 92%)
2l (R = Ph, 89%)

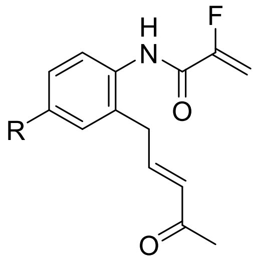

2m (R = H, 75%)
2n (R = Me, 79%)
2o (R = OMe, 79%)

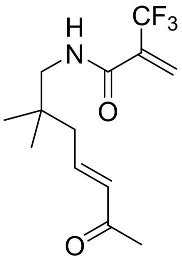

2p (46%)

Isolated yields after flash cromatography on silica gel

TABLE S2. OPTIMIZATION OF THE ENANTIOSELECTIVE CYCLIZATION OF SUBSTRATE 2a BY AN INTRAMOLECULAR AZA-MICHAEL REACTION

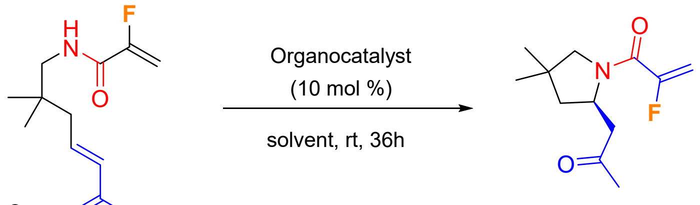

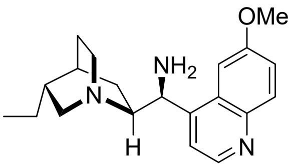

I (HQ-NH$_2$)

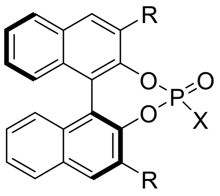

II (R = 3,5-$(CF_3)_2C_6H_3$; X = OH)
III (R = 9-anthracenyl; X = OH)
IV (R = 2,4,6-$(iPr)_3C_6H_2$; X = OH)
V (R = 2,4,6-$(iPr)_3C_6H_2$; X = NHTf)

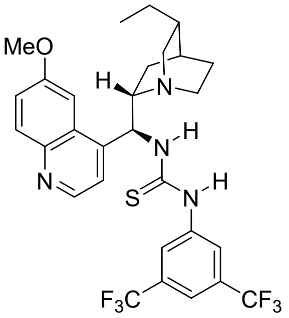

VI (Connon thiourea)

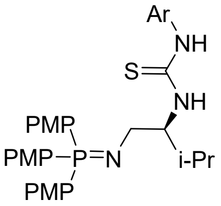

VII (Dixon phosphazene)
(Ar = 3,5-CF_3-C_6H_3;
PMP = 4-$MeOC_6H_4$)

entry	catalyst	solvent	Yield[b]	ee (%)[c]
1	I/ TFA	$CHCl_3$	--	--
2	**VI**	$CHCl_3$	--	--
3	**VII**	$CHCl_3$	--	--
4	**II**	$CHCl_3$	79%	23
5	**III**	$CHCl_3$	99%	32
6	**IV**	$CHCl_3$	86%	95
7	**IV**	$CHCl_3$	87%	94[d]
8	**IV**	toluene	90%	92
9	**IV**	THF	13%	98
10	**V**	$CHCl_3$	82%	33

[a] Reactions were performed with **2a** (0.2 mmol) and catalyst (10 mol %) in dry solvent. [b] Isolated yields after flash column chromatography. [c] Enantiomeric ratios were determined by HPLC analysis on a chiral stationary phase (see Supporting Information for details). [d] Reaction performed at 60ºC.

OPTIMIZATION OF THE METHYLENATION OF 3a WITH PETASIS REAGENT:

The results of the optimization to carry out the methylenation reaction of **3a** are summarized in Table S3.

Table S3. Optimization methylenation of **3a**.[a]

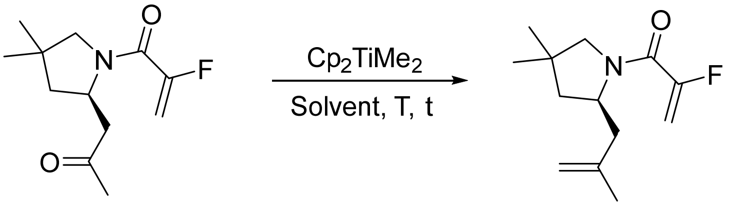

3a **4**

Entry	*Solvent*	*T (°C)*	*Equiv. (Cp)$_2$Ti(Me)$_2$*	*t (h)*	*Yield (%)*[b]
1	THF	66	1.5	12	10
2	THF	66	2.5	12	19
3	THF	66	2.5	24	9
4	DCE	84	1.5	4	15
5	DCE	84	1.5	12	11
6	DCE	84	2.5	2	23
7	DCE	84	2.5	4	38
8	DCE	84	2.5	6	35
9	Toluene	110	2.5	4	26
10	Toluene	95	2.5	4	56
11	Toluene	95	3.5	4	30
12	Toluene	95	2.5 + 1 equiv Cp_2TiCl_2	4	53

[a] Performed with 3a (0.2 mmol) in dry solvent [b] Yields were determined by 1H NMR analysis using trimethoxybenzene as internal standard

OPTIMIZATION RING CLOSING METHATESIS (RCM) OF 4:

The results of the optimization to carry out the RCM reaction of **4** are summarized in Table S4.

Table S4. Optimization ring closing metathesis of **4**.[a]

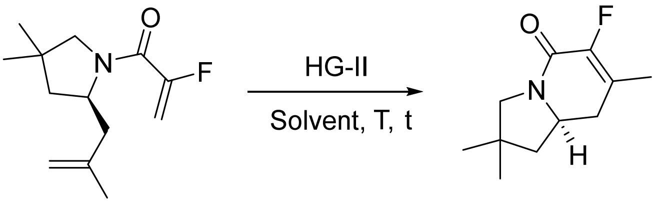

4 **5a**

Entry	*Solvent*	*T (°C)*	*HG-II (mol %)*	*Conc. (M)*	*t (h)*	*Yield (%)*
1	DCM	40	20	0.1	12	0
2	DCM	40	20	0.1	24	0
3	DCE	84	20	0.1	12	11
4	DCE	84	20	0.1	24	16
5	Toluene	110	20	0.1	24	23
6	Toluene	110	20	0.1	48	27
7	Toluene	110	2x10	0.1	48	33
8	Toluene	110	2x10	0.01	48	39
9	Toluene	110	2x10	0.005	48	51
10	Toluene	95	2x10	0.001	48	52
11	Toluene	95	2x5	0.005	48	50

[a] Performed with **4** (0.2 mmol) in dry solvent [b] Isolated yields after flash chromatography on silica gel

GENERAL PROCEDURE FOR THE SYNTHESIS OF FLUOROACRYLAMIDES 1a-i:

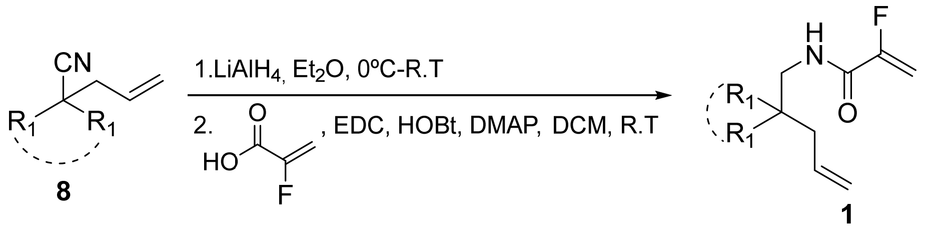

To a solution of the corresponding nitrile **8** in diethyl ether (0.2M), $LiAlH_4$ (3 equiv) was added at 0°C under N_2 atmosphere. The reaction mixture was stirred for 2 hours at room temperature (monitored by TLC), then $Na_2SO_4·10H_20$ was added with vigorous stirring until grey aluminum salts turned white. The suspension was filtered through a short pad of Celite washing with small portions of diethyl ether. The filtrate was dried with Na_2SO_4, filtered and concentrated under vacuum, obtaining a yellow oil that was employed without further purification. The crude primary amine was dissolved in CH_2Cl_2 (0.5 M), and was added to a solution of 1-Ethyl-3-(3-dimethylaminopropyl) carbodiimide hydrochloride (EDC·HCl) (1.2 equiv.), DMAP (1.6 equiv.), HOBt (1.2 equiv.) and 2-fluoroacrylic acid (1.1 equiv.) in CH_2Cl_2 (0.9 M) at 0°C under N_2 atmosphere. The reaction mixture was allowed to reach room temperature for 12 hours (monitored by TLC) and then it was hydrolyzed with H_2O, extracted with CH_2Cl_2 and dried over anhydrous Na_2SO_4. Finally, solvents were removed and the crude mixture was purified by flash chromatography on silica gel using mixtures of n-hexane and ethyl acetate as eluents.

***N*-(2,2-dimethylpent-4-en-1-yl)-2-fluoroacrylamide (1a).**

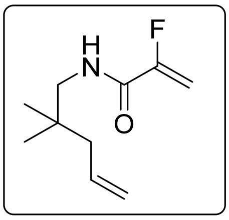

1a

By means of the general procedure described above, fluoroacrylamide **1a** (534 mg, 32% yield) was obtained as a yellow oil starting from 1 g (9.16 mmol) of **8a** after flash chromatography with 10:1 n-hexane: ethyl acetate. ^1H NMR (500 MHz, $CDCl_3$) δ 6.34 (bs, 1H), 5.83 (ddt, *J* = 16.8, 10.3, 7.5 Hz, 1H), 5.67 (dd, *J* = 48.1, 3.2 Hz, 1H), 5.13 – 5.03 (m, 3H), 3.18 (d, *J* = 6.5 Hz, 2H), 1.99 (dt, *J* = 7.4, 1.3 Hz, 2H), 0.91 (s, 6H). ^{19}F NMR (471 MHz, $CDCl_3$) δ -121.37. ^{13}C NMR (126 MHz, $CDCl_3$) δ 159.8 (d, *J* = 30.3 Hz), 156.6 (d, *J* = 270.4 Hz), 134.7, 117.9, 99.0 (d, *J* = 15.1 Hz), 48.9, 44.7, 35.0, 25.1. HRMS (ESI/Q-TOF): m/z $[M + H]^+$ calcd for $C_{10}H_{17}FNO$ $[M+H]^+$: 186.1289 found: 186.1287.

***N*-((1-allylcyclopropyl)methyl)-2-fluoroacrylamide (1b).**

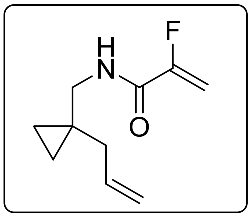

1b

By means of the general procedure described above, fluoroacrylamide **1b** (513 mg, 30% yield) was obtained as a yellow oil starting from 1 g (9.33 mmol) of **8b** after flash chromatography with 10:1 n-hexane: ethyl acetate. ^{1}H NMR (300 MHz, $CDCl_3$) δ 6.36 (bs, 1H), 5.82 (ddt, *J* = 17.3, 10.2, 7.1 Hz, 1H), 5.68 (dd, *J* = 48.0, 3.2 Hz, 1H), 5.15 – 5.04 (m, 3H), 3.26 (d, *J* = 5.8 Hz, 2H), 2.08 (d, *J* = 7.1 Hz, 2H), 0.51 – 0.42 (m, 4H). ^{19}F NMR (282 MHz, $CDCl_3$) δ -121.69. ^{13}C NMR (126 MHz, $CDCl_3$) δ 159.6 (d, *J* = 30.5 Hz), 156.6 (d, *J* = 270.3 Hz), 135.7, 117.3, 98.9 (d, *J* = 15.1 Hz), 46.0, 39.6, 19.9, 10.5. HRMS (ESI/Q-TOF): m/z $[M + H]^+$ calcd for $C_{10}H_{15}FNO$ $[M+H]^+$: 184.1132 found: 184.1135.

***N*-((1-allylcyclobutyl)methyl)-2-fluoroacrylamide (1c).**

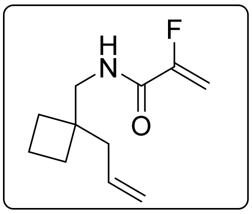

1c

By means of the general procedure described above, fluoroacrylamide **1c** (553 mg, 34% yield) was obtained as a colorless oil starting from 1 g (8.25 mmol) of **8c** after flash chromatography with 12:1 n-hexane: ethyl acetate. ^{1}H NMR (300 MHz, $CDCl_3$) δ 6.32 (bs, 1H), 5.88 – 5.75 (m, 1H), 5.68 (dd, *J* = 48.0, 3.2 Hz, 1H), 5.14 – 5.07 (m, 3H), 3.40 (d, *J* = 5.8 Hz, 2H), 2.22 (dt, *J* = 7.3, 1.1 Hz, 2H), 1.97 – 1.86 (m, 2H), 1.83 – 1.77 (m, 4H). ^{19}F NMR (282 MHz, $CDCl_3$) δ -121.75. ^{13}C NMR (126 MHz, $CDCl_3$) δ 159.9 (d, *J* = 30.3 Hz), 156.6 (d, *J* = 270.3 Hz), 134.6, 117.8, 99.0 (d, *J* = 15.1 Hz), 46.3, 42.6, 41.7, 29.0, 15.1. HRMS (ESI/Q-TOF): m/z $[M + H]^+$ calcd for $C_{11}H_{17}FNO$ $[M+H]^+$: 198.1289 found: 198.1287.

N-((1-allylcyclopentyl)methyl)-2-fluoroacrylamide (1d).

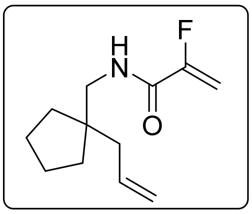

1d

By means of the general procedure described above, fluoroacrylamide **1d** (858 mg, 61% yield) was obtained as a colorless oil starting from 900 mg (6.66 mmol) of **8d** after flash chromatography with 15:1 n-hexane: ethyl acetate. ^{1}H NMR (300 MHz, $CDCl_3$) δ 6.37 (s, 1H), 5.91 – 5.79 (m, 1H), 5.67 (dd, *J* = 48.1, 3.2 Hz, 1H), 5.14 – 5.06 (m, 3H), 3.27 (dd, *J* = 6.3, 0.8 Hz, 2H), 2.11 (dt, *J* = 7.4, 1.1 Hz, 2H), 1.67 – 1.60 (m, 4H), 1.46 – 1.41 (m, 4H). ^{19}F NMR (282 MHz, $CDCl_3$) δ -121.81. ^{13}C NMR (126 MHz, $CDCl_3$) δ 159.8 (d, *J* = 30.2 Hz), 156.6 (d, *J* = 270.3 Hz), 135.6, 117.7, 98.9 (d, *J* = 15.1 Hz), 46.4, 46.3, 43.0, 35.4, 24.9. HRMS (ESI/Q-TOF): m/z [M + H]$^+$ calcd for $C_{12}H_{19}FNO$ [M+H]$^+$: 212.1445 found: 212.1445.

N-((1-allylcyclohexyl)methyl)-2-fluoroacrylamide (1e).

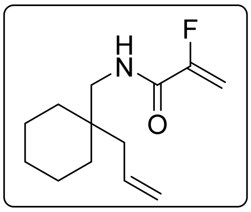

1e

By means of the general procedure described above, fluoroacrylamide **1e** (755 mg, 50% yield) was obtained as a white solid starting from 1 g (6.7 mmol) of **8e** after flash chromatography with 18:1 n-hexane: ethyl acetate. M. p. = 57-59.3 °C. ^{1}H NMR (300 MHz, $CDCl_3$) δ 6.33 (bs, 1H), 5.94 – 5.80 (m, 1H), 5.68 (dd, *J* = 48.1, 3.2 Hz, 1H), 5.14 – 5.06 (m, 3H), 3.28 (d, *J* = 6.4 Hz, 2H), 2.08 (d, *J* = 7.5 Hz, 2H), 1.53 – 1.39 (m, 6H), 1.35 – 1.31 (m, 4H). ^{19}F NMR (282 MHz, $CDCl_3$) δ -121.83. ^{13}C NMR (75 MHz, $CDCl_3$) δ 159.6 (d, *J* = 30.1 Hz), 156.5 (d, *J* = 270.5 Hz), 134.6, 117.7, 98.8 (d, *J* = 15.1 Hz), 45.7, 41.2, 37.1, 33.6, 26.1, 21.4. HRMS (ESI/Q-TOF): m/z [M + H]$^+$ calcd for $C_{13}H_{21}FNO$ [M+H]$^+$: 226.1602 found: 226.1600.

_N_-((4-allyltetrahydro-2H-pyran-4-yl)methyl)-2-fluoroacrylamide (1f).

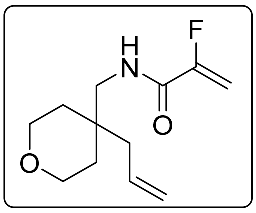

1f

By means of the general procedure described above, fluoroacrylamide **1f** (410 mg, 35% yield) was obtained as a white solid starting from 780 mg (5.16 mmol) of **8f** after flash chromatography with 3:1 n-hexane: ethyl acetate. M. p. = 54-55.4 °C. ^{1}H NMR (300 MHz, $CDCl_3$) δ 6.40 (bs, 1H), 5.89 (ddt, *J* = 15.9, 11.0, 7.5 Hz, 1H), 5.71 (dd, *J* = 48.0, 3.2 Hz, 1H), 5.20 – 5.13 (m, 3H), 3.74 (h, *J* = 6.8 Hz, 4H), 3.41 (dd, *J* = 6.7, 0.8 Hz, 2H), 2.20 (dt, *J* = 7.4, 1.0 Hz, 2H), 1.50 (t, *J* = 5.5 Hz, 4H). ^{19}F NMR (282 MHz, $CDCl_3$) δ -121.92. ^{13}C NMR (75 MHz, $CDCl_3$) δ 159.9 (d, *J* = 30.3 Hz), 156.4 (d, *J* = 270.1 Hz), 133.8, 118.7, 99.3 (d, *J* = 15.0 Hz), 63.5, 44.8, 41.5, 35.5, 33.5. HRMS (ESI/Q-TOF): m/z $[M + H]^+$ calcd for $C_{12}H_{19}FNO_2$ $[M+H]^+$: 228.1394 found: 228.1396.

tert-butyl 4-allyl-4-((2-fluoroacrylamido)methyl)piperidine-1-carboxylate (1g)

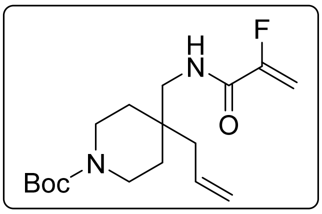

1g

By means of the general procedure described above, fluoroacrylamide **1g** (170 mg, 26% yield) was obtained as a colorless oil starting from 500 mg (2.00 mmol) of **8g** after flash chromatography with 4:1 n-hexane: ethyl acetate. ^{1}H NMR (300 MHz, $CDCl_3$) δ 6.36 (bs, 1H), 5.86 (ddt, *J* = 16.4, 10.7, 7.5 Hz, 1H), 5.68 (dd, *J* = 48.1, 3.2 Hz, 1H), 5.22 – 5.07 (m, 3H), 3.59 – 3.18 (m, 6H), 2.12 (d, *J* = 7.4 Hz, 2H), 1.44 (s, 9H), 1.44 – 1.39 (m, 2H), 1.27 – 1.23 (m, 2H). ^{19}F NMR (282 MHz, $CDCl_3$) δ -121.92. ^{13}C NMR (75 MHz, $CDCl_3$) δ 159.9 (d, *J* = 30.4 Hz), 156.3 (d, *J* = 270.1 Hz), 155.0, 133.8, 118.7, 99.3 (d, *J* = 14.9 Hz), 79.6, 44.6, 40.9, 36.3, 32.7, 29.8, 28.6. HRMS (ESI/Q-TOF): m/z $[M + H]^+$ calcd for $C_{17}H_{28}FN_2O_3$ $[M+H]^+$: 327.2078 found: 327.2077.

N-(2,2-diphenylpent-4-en-1-yl)-2-fluoroacrylamide (1h).

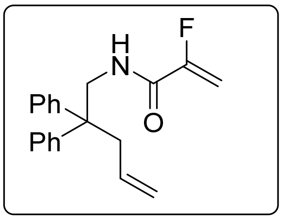

1h

By means of the general procedure described above, fluoroacrylamide **1h** (278 mg, 42% yield) was obtained as a yellow oil starting from 500 mg (2.14 mmol) of **8h** after flash chromatography with 10:1 n-hexane: ethyl acetate. ^{1}H NMR (300 MHz, $CDCl_3$) δ 7.38 – 7.33 (m, 4H), 7.30 – 7.26 (m, 2H), 7.25 – 7.21 (m, 4H), 5.91 (bs, 1H), 5.66 (dd, *J* = 47.8, 3.2 Hz, 1H), 5.45 (ddt, *J* = 18.6, 9.5, 7.1 Hz, 1H), 5.10– 5.00 (m, 3H), 4.06 (d, *J* = 5.8 Hz, 2H), 2.90 (d, *J* = 7.1 Hz, 2H). ^{19}F NMR (282 MHz, $CDCl_3$) δ -122.01. ^{13}C NMR (75 MHz, $CDCl_3$) δ 159.5 (d, *J* = 30.4 Hz), 156.3 (d, *J* = 270.4 Hz), 145.0, 133.6, 128.6, 128.0, 126.9, 119.0, 99.0 (d, *J* = 15.0 Hz), 50.3, 45.9, 42.1. HRMS (ESI/Q-TOF): m/z $[M + H]^+$ calcd for $C_{20}H_{21}FNO$ $[M+H]^+$: 310.1602 found: 310.1607.

2-fluoro-N-(pent-4-en-1-yl)acrylamide (1i).

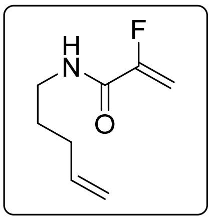

1i

By means of the general procedure described above, fluoroacrylamide **1i** (446 mg, 23% yield) was obtained as a yellow oil starting from 1 g (12.3 mmol) of **8i** after flash chromatography with 10:1 n-hexane: ethyl acetate. ^{1}H NMR (500 MHz, $CDCl_3$) δ 6.29 (bs, 1H), 5.81 (ddt, *J* = 16.9, 10.2, 6.7 Hz, 1H), 5.68 (dd, *J* = 48.0, 3.2 Hz, 1H), 5.10 (dd, *J* = 15.4, 3.2 Hz, 1H), 5.06 (dq, *J* = 17.1, 1.6 Hz, 1H), 5.00 (dq, *J* = 10.2, 1.3 Hz, 1H), 3.37 (q, *J* = 6.6 Hz, 2H), 2.14 – 2.10 (m, 2H), 1.67 (p, *J* = 7.3 Hz, 2H). ^{19}F NMR (471 MHz, $CDCl_3$) δ -121.29. ^{13}C NMR (126 MHz, $CDCl_3$) δ 159.7 (d, *J* = 30.4 Hz), 156.6 (d, *J* = 270.3 Hz), 137.6, 115.7, 98.8 (d, *J* = 14.9 Hz), 39.1, 31.2, 28.6. HRMS (ESI/Q-TOF): m/z $[M + H]^+$ calcd for $C_8H_{13}FNO$ $[M+H]^+$: 158.0976 found: 158.0974.

GENERAL PROCEDURE FOR THE SYNTHESIS OF FLUOROACRYLAMIDES 1j-l:

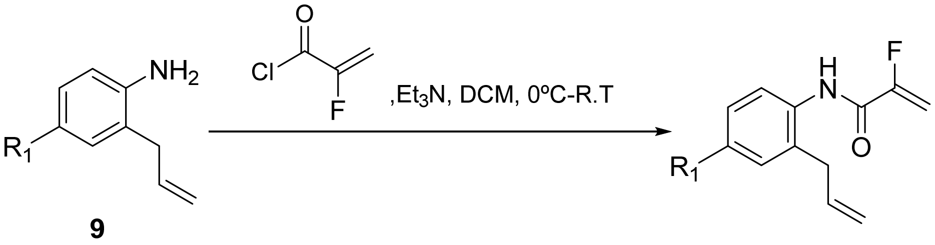

To a solution of the corresponding aniline **9** in CH_2Cl_2 (0.5 M), Et_3N (2.0 equiv) was added at 0 °C under N_2 atmosphere. Then a freshly prepared 2-fluoroacryloyl chloride[3] (1.5 equiv) was added dropwise, and the reaction mixture was allowed to reach room temperature for 12 hours (monitored by TLC). Later it was hydrolyzed with saturated NH_4Cl solution, extracted with CH_2Cl_2 and dried over anhydrous Na_2SO_4. Finally, solvents were removed and the crude mixture was purified by flash chromatography on silica gel using mixtures of n-hexane and ethyl acetate as eluents.

***N*-(2-allylphenyl)-2-fluoroacrylamide (1j).**

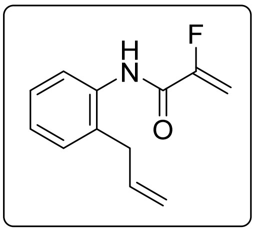

1j

By means of the general procedure described above, fluoroacrylamide **1j** (320 mg, 70% yield) was obtained as a yellow solid starting from 300 mg (2.25 mmol) of aniline **9a** after flash chromatography with 25:1 n-hexane: ethyl acetate. M. p. = 44-45 °C. ^{1}H NMR (300 MHz, $CDCl_3$) δ 8.12 (bs, 1H), 8.08 – 8.03 (m, 1H), 7.33 – 7.27 (m, 1H), 7.23 – 7.09 (m, 2H), 5.95 (ddt, *J* = 17.1, 10.2, 6.2 Hz, 1H), 5.82 (dd, *J* = 47.8, 3.3 Hz, 1H), 5.30 – 5.11 (m, 3H), 3.42 (dt, *J* = 6.2, 1.7 Hz, 2H). ^{19}F NMR (282 MHz, $CDCl_3$) δ -121.43. ^{13}C NMR (126 MHz, $CDCl_3$) δ 157.5 (d, *J* = 29.9 Hz), 156.6 (d, *J* = 271.6 Hz), 135.7, 135.1, 130.5, 130.1, 127.8, 126.0, 123.3, 117.3, 100.0 (d, *J* = 15.0 Hz), 37.2. HRMS (ESI/Q-TOF): m/z $[M + H]^+$ calcd for $C_{12}H_{13}FNO$ $[M+H]^+$: 206.0976 found: 206.0975.

[3] Jiao, X.; Zhang, Q.; Zhang, Y.; Shao, J.; Ding, L.; Tang, C.; Feng, B. Synthesis and biological evaluation of new series of quinazoline derivatives as EGFR/HER2 dual-target inhibitors. *Bioorg. Med. Chem. Lett.* **2022**, *67*, 128703.

***N*-(2-allyl-4-methylphenyl)-2-fluoroacrylamide (1k).**

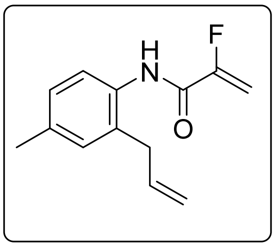

1k

By means of the general procedure described above, fluoroacrylamide **1k** (381 mg, 79% yield) was obtained as a yellow solid starting from 324 mg (2.2 mmol) of aniline **9b** after flash chromatography with 30:1 n-hexane: ethyl acetate. M. p. = 40-41 °C. ^{1}H NMR (300 MHz, $CDCl_3$) δ 8.05 (bs, 1H), 7.84 (d, *J* = 8.2 Hz, 1H), 7.10 (dd, *J* = 8.2, 2.1 Hz, 1H), 7.02 (d, *J* = 2.1 Hz, 1H), 6.00 – 5.89 (m, 1H), 5.79 (dd, *J* = 47.9, 3.3 Hz, 1H), 5.25 – 5.09 (m, 3H), 3.36 (dt, *J* = 6.2, 1.7 Hz, 2H), 2.33 (s, 3H). ^{19}F NMR (282 MHz, $CDCl_3$) δ -121.36. ^{13}C NMR (75 MHz, $CDCl_3$) δ 157.5 (d, *J* = 29.6 Hz), 156.7 (d, *J* = 271.7 Hz), 135.8, 135.7, 132.3, 131.1, 130.2, 128.2, 123.4, 117.0, 99.7 (d, *J* = 15.0 Hz), 37.1, 21.0. HRMS (ESI/Q-TOF): m/z $[M + H]^+$ calcd for $C_{13}H_{15}FNO$ $[M+H]^+$: 220.1132 found: 220.1126.

***N*-(2-allyl-4-methoxyphenyl)-2-fluoroacrylamide (1l).**

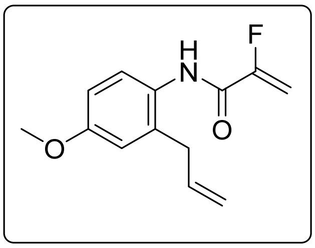

1l

By means of the general procedure described above, fluoroacrylamide **1l** (568 mg, 80% yield) was obtained as a yellow solid starting from 490 mg (3 mmol) of aniline **9c** after flash chromatography with 20:1 n-hexane: ethyl acetate. M. p. = 51-52 °C. ^{1}H NMR (300 MHz, $CDCl_3$) δ 7.93 (bs, 1H), 7.79 (d, *J* = 8.8 Hz, 1H), 6.82 (dd, *J* = 8.8, 2.9 Hz, 1H), 6.76 (d, *J* = 2.9 Hz, 1H), 6.00 – 5.88 (m, 1H), 5.79 (dd, *J* = 47.9, 3.3 Hz, 1H), 5.25 – 5.09 (m, 3H), 3.80 (s, 3H), 3.36 (dt, *J* = 6.2, 1.7 Hz, 2H). ^{19}F NMR (282 MHz, $CDCl_3$) δ -121.45. ^{13}C NMR (126 MHz, $CDCl_3$) δ 157.7, 156.7 (d, *J* = 267.0 Hz), 157.6 (d, *J* = 33.6 Hz), 135.6, 132.8, 127.8, 125.4, 117.2, 116.1, 112.3, 99.7 (d, *J* = 15.2 Hz), 55.6, 37.2. HRMS (ESI/Q-TOF): m/z $[M + H]^+$ calcd for $C_{13}H_{15}FNO_2$ $[M+H]^+$: 236.1081 found: 236.1077.

SYNTHESIS OF *N*-(2,2-DIMETHYLPENT-4-EN-1-YL)-2-(TRIFLUOROMETHYL)ACRYLAMIDE (1m):

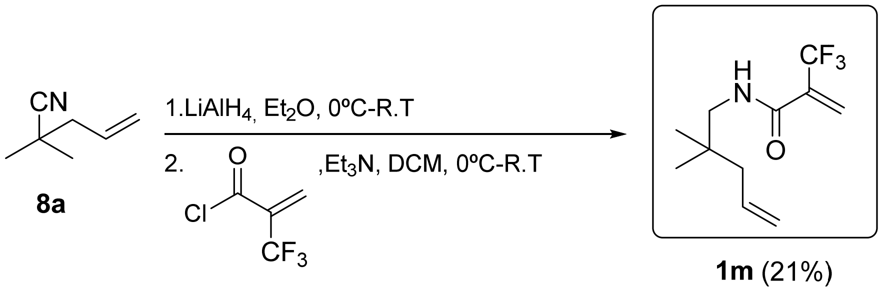

1m (21%)

To a solution of nitrile **8a** (1 g, 9.88 mmol) in diethyl ether (49.4 mL, 0.2M), $LiAlH_4$ (1.125 g, 3 equiv) was added at 0°C under N_2 atmosphere. The reaction mixture was stirred for 2 hours at room temperature (monitored by TLC), then $Na_2SO_4·10H_20$ was added with vigorous stirring until grey aluminum salts turned white. The suspension was filtered through a short pad of Celite washing with small portions of diethyl ether. The filtrate was dried with Na_2SO_4, filtered and concentrated under vacuum, obtaining a yellow oil that was employed without further purification. The crude primary amine was dissolved in CH_2Cl_2 (19.8 mL, 0.5 M), and Et_3N (2.75 mL, 2.0 equiv) was added at 0 °C, followed by a freshly prepared 2-(trifluoromethyl)acryloyl chloride[4] (1.722 g, 1.1 equiv) The reaction mixture was allowed to reach room temperature for 12 hours (monitored by TLC) and then it was hydrolyzed with saturated NH_4Cl solution, extracted with CH_2Cl_2 and dried over anhydrous Na_2SO_4. Finally, solvents were removed and the crude mixture was purified by flash chromatography on silica gel with 9:1 n-hexane:ethyl acetate as eluent to afford **1m** (488 mg, 21% yield) as a yellow oil. ^{1}H NMR (500 MHz, $CDCl_3$) δ 6.60 (q, *J* = 1.8 Hz, 1H), 6.24 (q, *J* = 1.5 Hz, 1H), 5.99 (bs, 1H), 5.84 (ddt, *J* = 17.5, 10.2, 7.5 Hz, 2H), 5.10 – 5.04 (m, 2H), 3.23 (d, *J* = 6.2 Hz, 2H), 2.01 (d, *J* = 7.5 Hz, 2H), 0.93 (s, 6H). ^{19}F NMR (282 MHz, $CDCl_3$) δ -64.01. ^{13}C NMR (75 MHz, $CDCl_3$) δ 160.8, 135.6, 133.9 (q, *J* = 30.7 Hz), 129.7 (q, *J* = 5.7 Hz), 122.5 (q, *J* = 273.05 Hz), 117.7, 46.8, 46.4, 43.2, 35.5, 24.8. HRMS (ESI/Q-TOF): m/z $[M + H]^+$ calcd for $C_{11}H_{17}F_3NO$ $[M+H]^+$: 236.1257 found: 236.1252.

[4] Yamazaki, T.; Ichige, T.; Takei, S.; Kawashita, S.; Kitazume, T.; Kubota, T. Effect of allylic CH_{3}-nFn groups (n = 1-3) on π-facial diastereoselection. *Org. Lett*. **2021**, *3*, 2915.

GENERAL PROCEDURE FOR THE CROSS-METATHESIS REACTON. SYNTHESIS OF ENONES 2:

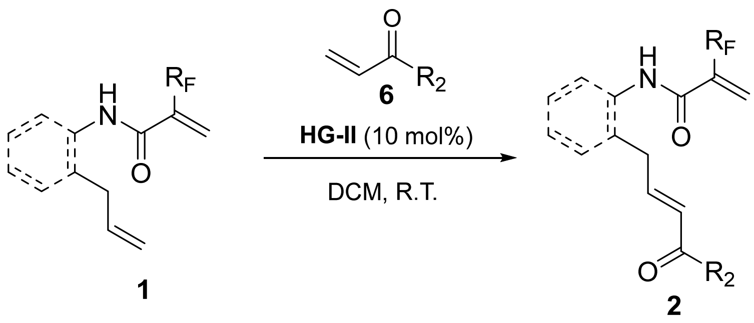

HG-II = $Cl_2(IMes)Ru{=}CH(o\text{-}iPrC_6H_4)$

To a solution of the corresponding fluoroacrylamide **1** in dichloromethane (0.3 M), the appropiate vinyl ketone **6** (3.0 equiv) and second generation Hoveyda-Grubbs catalyst (10 mol %) were successively added. The resulting mixture was stirred for 12 h at room temperature (monitored by TLC) and then concentrated to dryness and purified by means of flash column chromatography on silica gel using mixtures of n-hexane and ethyl acetate as eluents.

***(E)-N*-(2,2-dimethyl-6-oxohept-4-en-1-yl)-2-fluoroacrylamide (2a).**

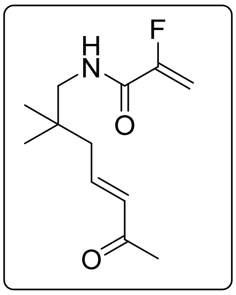

2a

By means of the general procedure described above, enone **2a** (331 mg, 90% yield) was obtained as a brown oil starting from 300 mg (1.62 mmol) of **1a** after flash chromatography with 4:1 n-hexane: ethyl acetate. ^{1}H NMR (300 MHz, $CDCl_3$) δ 6.82 (dt, *J* = 15.7, 7.8 Hz, 1H), 6.36 (bs, 1H), 6.08 (dt, *J* = 15.8, 1.3 Hz, 1H), 5.68 (dd, *J* = 48.1, 3.2 Hz, 1H), 5.13 (dd, *J* = 15.4, 3.3 Hz, 1H), 3.22 (d, *J* = 6.7 Hz, 2H), 2.24 (s, 3H), 2.14 (dd, *J* = 7.8, 1.4 Hz, 2H), 0.96 (s, 6H). ^{19}F NMR (282 MHz, $CDCl_3$) δ -121.33. ^{13}C NMR (75 MHz, $CDCl_3$) δ 198.3, 159.9 (d, *J* = 30.4 Hz), 156.3 (d, *J* = 270.2 Hz), 144.0, 134.0, 99.3 (d, *J* = 15.1 Hz), 49.0, 42.9, 35.8, 27.2, 25.1. HRMS (EI) calcd for $C_{12}H_{19}FNO_2$ $[M+H]^+$: 228.1394 found: 228.1392.

(*E*)-*N*-(2,2-dimethyl-6-oxonon-4-en-1-yl)-2-fluoroacrylamide (2b).

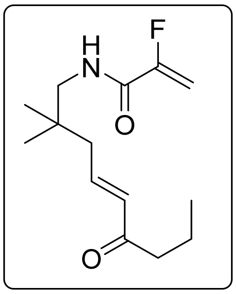

2b

By means of the general procedure described above, enone **2b** (155 mg, 95% yield) was obtained as a brown oil starting from 150 mg (0.64 mmol) of **1a** after flash chromatography with 6:1 n-hexane: ethyl acetate. ^{1}H NMR (300 MHz, $CDCl_3$) δ 6.84 (dt, *J* = 15.6, 7.8 Hz, 1H), 6.32 (bs, 1H), 6.12 (dt, *J* = 15.7, 1.3 Hz, 1H), 5.69 (dd, *J* = 48.1, 3.2 Hz, 1H), 5.14 (dd, *J* = 15.4, 3.2 Hz, 1H), 3.22 (d, *J* = 6.3 Hz, 2H), 2.52 (t, *J* = 7.3 Hz, 2H), 2.14 (dd, *J* = 7.8, 1.4 Hz, 2H), 1.70 – 1.58 (m, 2H), 0.96 (s, 6H), 0.94 (t, *J* = 7.4 Hz, 3H). ^{19}F NMR (282 MHz, $CDCl_3$) δ -121.96. ^{13}C NMR (75 MHz, $CDCl_3$) δ 200.4, 159.9 (d, *J* = 30.3 Hz), 156.4 (d, *J* = 270.1 Hz), 142.7, 133.2, 99.4 (d, *J* = 15.0 Hz), 49.1, 43.0, 42.4, 35.8, 25.1, 17.8, 13.9. HRMS (ESI/Q-TOF): m/z $[M + H]^+$ calcd for $C_{14}H_{23}FNO_2$ $[M+H]^+$: 256.1707 found: 256.1698.

(*E*)-*N*-(2,2-dimethyl-6-oxoundec-4-en-1-yl)-2-fluoroacrylamide (2c).

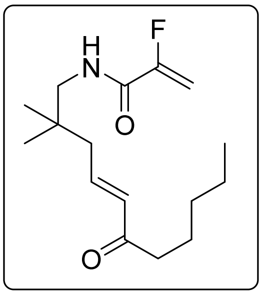

2c

By means of the general procedure described above, enone **2c** (166 mg, 92% yield) was obtained as a yellow oil starting from 150 mg (0.64 mmol) of **1a** after flash chromatography with 6:1 n-hexane: ethyl acetate. ^{1}H NMR (300 MHz, $CDCl_3$) δ 6.83 (dt, *J* = 15.6, 7.8 Hz, 1H), 6.33 (bs, 1H), 6.11 (dt, *J* = 15.7, 1.3 Hz, 1H), 5.69 (dd, *J* = 48.1, 3.2 Hz, 1H), 5.13 (dd, *J* = 15.4, 3.2 Hz, 1H), 3.21 (d, *J* = 6.4 Hz, 2H), 2.52 (t, *J* = 7.68 Hz, 2H), 2.14 (dd, *J* = 7.8, 1.4 Hz, 2H), 1.65 – 1.55 (m, 2H), 1.39 – 1.22 (m, 4H), 0.95 (s, 6H), 0.88 (t, *J* = 6.95 Hz, 3H). ^{19}F NMR (282 MHz, $CDCl_3$) δ -121.82. ^{13}C NMR (75 MHz, $CDCl_3$) δ 200.6, 159.9 (d, *J* = 30.4 Hz), 156.4 (d, *J* = 270.2 Hz), 142.6, 133.1, 99.3 (d, *J* = 15.0 Hz), 49.1, 43.0, 40.5, 35.8, 31.6, 25.1, 24.1, 22.6, 14.1. HRMS (ESI/Q-TOF): m/z $[M + H]^+$ calcd for $C_{16}H_{27}FNO_2$ $[M+H]^+$: 284.2020 found: 284.2034.

(*E*)-*N*-(2,2-dimethyl-6-oxo-6-phenylhex-4-en-1-yl)-2-fluoroacrylamide (2d).

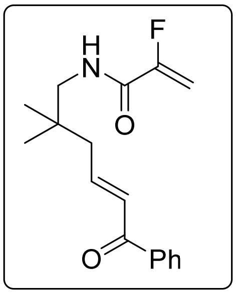

2d

By means of the general procedure described above, enone **2d** (157 mg, 67% yield) was obtained as a white solid starting from 190 mg (0.81 mmol) of **1a** after flash chromatography with 6:1 n-hexane: ethyl acetate. M. p. = 65-66.5 °C. ^{1}H NMR (300 MHz, $CDCl_3$) δ 7.96 – 7.90 (m, 2H), 7.61 – 7.53 (m, 1H), 7.51 – 7.43 (m, 2H), 7.08 (dt, *J* = 15.2, 7.6 Hz, 1H), 6.93 (dt, *J* = 15.2, 1.1 Hz, 1H), 6.33 (bs, 1H), 5.69 (dd, *J* = 48.1, 3.2 Hz, 1H), 5.13 (dd, *J* = 15.4, 3.2 Hz, 1H), 3.26 (d, *J* = 6.3 Hz, 2H), 2.27 (dd, *J* = 7.6, 1.1 Hz, 2H), 1.01 (s, 6H). ^{19}F NMR (282 MHz, $CDCl_3$) δ -121.83. ^{13}C NMR (75 MHz, $CDCl_3$) δ 190.3, 159.9 (d, *J* = 30.3 Hz), 156.4 (d, *J* = 270.1 Hz), 145.3, 137.9, 132.9, 128.8, 128.72, 128.69, 99.4 (d, *J* = 14.9 Hz), 49.3, 43.4, 36.0, 25.1.HRMS (ESI/Q-TOF): m/z [M + H]$^+$ calcd for $C_{17}H_{21}FNO_2$ [M+H]$^+$: 290.1551 found: 290.1557.

***(E)*-2-fluoro-*N*-((1-(4-oxopent-2-en-1-yl)cyclopropyl)methyl)acrylamide (2e).**

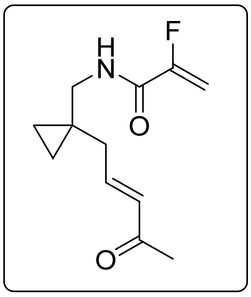

2e

By means of the general procedure described above, enone **2e** (273 mg, 89% yield) was obtained as a brown oil starting from 250 mg (1.36 mmol) of **1b** after flash chromatography with 4:1 n-hexane: ethyl acetate. ^{1}H NMR (300 MHz, $CDCl_3$) δ 6.80 (dt, *J* = 15.9, 7.2 Hz, 1H), 6.34 (bs, 1H), 6.12 (d, *J* = 17.1 Hz, 1H), 5.67 (dd, *J* = 48.0, 3.2 Hz, 1H), 5.12 (dd, *J* = 15.4, 3.2 Hz, 1H), 3.26 (d, *J* = 6.1 Hz, 2H), 2.25 (s, 3H), 2.21 (dd, *J* = 7.2, 1.5 Hz, 2H), 0.58 – 0.47 (m, 4H). ^{19}F NMR (282 MHz, $CDCl_3$) δ -121.66. ^{13}C NMR (75 MHz, $CDCl_3$) δ 198.4, 159.7 (d, *J* = 30.5 Hz), 156.3 (d, *J* = 270.1 Hz), 144.8, 132.9, 99.3 (d, *J* = 15.0 Hz), 45.7, 38.0, 27.2, 20.0, 10.7. HRMS (ESI/Q-TOF): m/z [M + H]$^+$ calcd for $C_{12}H_{17}FNO_2$ [M+H]$^+$: 226.1238 found: 226.1236.

(*E*)-2-fluoro-*N*-((1-(4-oxopent-2-en-1-yl)cyclobutyl)methyl)acrylamide (2f).

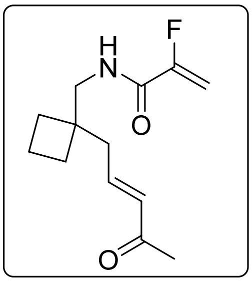

2f

By means of the general procedure described above, enone **2f** (204 mg, 84% yield) was obtained as a brown oil starting from 200 mg (1.01 mmol) of **1c** after flash chromatography with 5:1 n-hexane: ethyl acetate. ^{1}H NMR (500 MHz, $CDCl_3$) δ 6.80 (dt, *J* = 15.9, 7.5 Hz, 1H), 6.31 (bs, 1H), 6.13 (dt, *J* = 15.9, 1.4 Hz, 1H), 5.69 (dd, *J* = 48.0, 3.3 Hz, 1H), 5.13 (dd, *J* = 15.4, 3.3 Hz, 1H), 3.42 (d, *J* = 6.2 Hz, 2H), 2.36 (dd, *J* = 7.6, 1.4 Hz, 2H), 2.25 (s, 3H), 1.96 – 1.83 (m, 6H). ^{19}F NMR (471 MHz, $CDCl_3$) δ -121.18. ^{13}C NMR (126 MHz, $CDCl_3$) δ 198.3, 160.0 (d, *J* = 30.5 Hz), 156.3 (d, *J* = 270.0 Hz), 143.8, 133.7, 99.3 (d, *J* = 14.9 Hz), 46.2, 42.2, 40.8, 29.1, 27.1, 15.1. HRMS (ESI/Q-TOF): m/z $[M + H]^+$ calcd for $C_{13}H_{19}FNO_2$ $[M+H]^+$: 240.1392 found: 240.1392.

(*E*)-2-fluoro-*N*-((1-(4-oxopent-2-en-1-yl)cyclopentyl)methyl)acrylamide (2g).

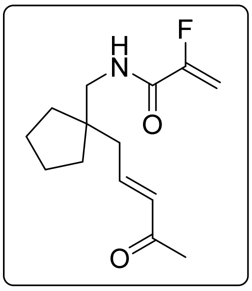

2g

By means of the general procedure described above, enone **2g** (108 mg, 90% yield) was obtained as a brown oil starting from 100 mg (6.66 mmol) of **1d** after flash chromatography with 6:1 n-hexane: ethyl acetate. ^{1}H NMR (300 MHz, $CDCl_3$) δ 6.83 (dt, *J* = 15.5, 7.7 Hz, 1H), 6.31 (bs, 1H), 6.10 (dt, *J* = 15.8, 1.4 Hz, 1H), 5.68 (dd, *J* = 48.1, 3.2 Hz, 1H), 5.13 (dd, *J* = 15.4, 3.2 Hz, 1H), 3.31 – 3.29 (m, 2H), 2.25 (dd, *J* = 7.6, 1.3 Hz, 2H), 2.25 (s, 3H), 1.72 – 1.62 (m, 4H), 1.54 – 1.45 (m, 4H). ^{19}F NMR (282 MHz, $CDCl_3$) δ -121.77. ^{13}C NMR (126 MHz, $CDCl_3$) δ 198.4, 159.9 (d, *J* = 30.3 Hz), 156.4 (d, *J* = 270.0 Hz), 144.7, 133.8, 99.4 (d, *J* = 14.9 Hz), 47.0, 46.4, 41.0, 35.5, 27.2, 24.9.HRMS (ESI/Q-TOF): m/z $[M + H]^+$ calcd for $C_{14}H_{21}FNO_2$ $[M+H]^+$: 254.1551 found: 254.1544.

(*E*)-2-fluoro-*N*-((1-(4-oxopent-2-en-1-yl)cyclohexyl)methyl)acrylamide (2h).

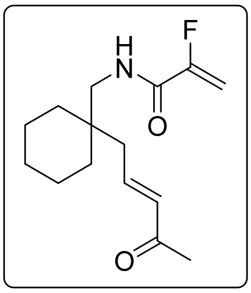

2h

By means of the general procedure described above, enone **2h** (222 mg, 75% yield) was obtained as a white solid starting from 250 mg (1.11 mmol) of **1e** after flash chromatography with 4:1 n-hexane: ethyl acetate. M. p. = 59.5-61 °C. ^{1}H NMR (300 MHz, $CDCl_3$) δ 6.87 (dt, *J* = 15.7, 7.8 Hz, 1H), 6.26 (bs, 1H), 6.10 (dt, *J* = 15.8, 1.3 Hz, 1H), 5.68 (dd, *J* = 48.1, 3.2 Hz, 1H), 5.13 (dd, *J* = 15.5, 3.2 Hz, 1H), 3.30 (d, *J* = 6.5 Hz, 2H), 2.25 (s, 3H), 2.21 (dd, *J* = 7.8, 1.4 Hz, 2H), 1.54 – 1.32 (m, 10H). ^{19}F NMR (282 MHz, $CDCl_3$) δ -121.77. ^{13}C NMR (75 MHz, $CDCl_3$) δ 198.4, 159.9 (d, *J* = 30.2 Hz), 156.4 (d, *J* = 270.1 Hz), 144.2, 133.9, 99.3 (d, *J* = 15.0 Hz), 46.0, 39.2, 38.2, 33.7, 27.1, 26.0, 21.4. HRMS (ESI/Q-TOF): m/z $[M + H]^+$ calcd for $C_{15}H_{23}FNO_2$ $[M+H]^+$: 268.1707 found: 268.1706.

(*E*)-2-fluoro-*N*-((4-(4-oxopent-2-en-1-yl)tetrahydro-2H-pyran-4-yl)methyl)acrylamide (2i).

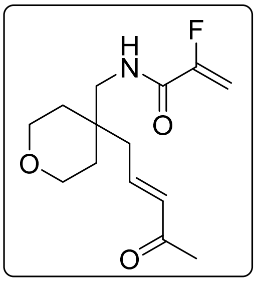

2i

By means of the general procedure described above, enone **2i** (270 mg, 76% yield) was obtained as a brown oil starting from 300 mg (1.32 mmol) of **1f** after flash chromatography with 1:2 n-hexane: ethyl acetate. ^{1}H NMR (300 MHz, $CDCl_3$) δ 6.85 (dt, *J* = 15.7, 7.8 Hz, 1H), 6.34 (bs, 1H), 6.14 (dt, *J* = 15.9, 1.3 Hz, 1H), 5.69 (dd, *J* = 48.1, 3.3 Hz, 1H), 5.15 (dd, *J* = 15.4, 3.3 Hz, 1H), 3.71 (qt, *J* = 12.1, 5.3 Hz, 4H), 3.39 (d, *J* = 6.6 Hz, 2H), 2.31 (dd, *J* = 7.7, 1.3 Hz, 2H), 2.26 (s, 3H), 1.50 (t, *J* = 5.4 Hz, 4H). ^{19}F NMR (282 MHz, $CDCl_3$) δ -121.78.^{13}C NMR (126 MHz, $CDCl_3$) δ 198.0, 160.0 (d, *J* = 30.5 Hz), 156.1 (d, *J* = 269.8 Hz), 142.8, 134.3, 99.6 (d, *J* = 14.9 Hz), 63.4, 45.1, 39.2, 36.3, 33.4, 27.3. HRMS (ESI/Q-TOF): m/z $[M + H]^+$ calcd for $C_{14}H_{21}FNO_3$ $[M+H]^+$: 270.1500 found: 270.1500.

(*E*)-tert-butyl 4-((2-fluoroacrylamido)methyl)-4-(4-oxopent-2-en-1-yl)piperidine-1-carboxylate (2j)

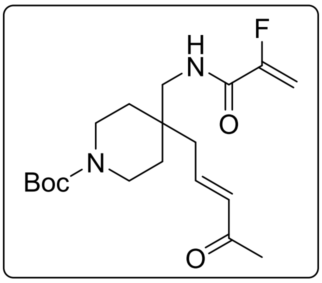

2j

By means of the general procedure described above, enone **2j** (117 mg, 69% yield) was obtained as brown oil starting from 150 mg (0.46 mmol) of **1g** after flash chromatography with 1:1 n-hexane: ethyl acetate. ^{1}H NMR (500 MHz, $CDCl_3$) δ 6.84 (dt, *J* = 15.6, 7.8 Hz, 1H), 6.31 (bs, 1H), 6.14 (d, *J* = 15.8 Hz, 1H), 5.70 (dd, *J* = 48.0, 3.3 Hz, 1H), 5.16 (dd, *J* = 15.4, 3.3 Hz, 1H), 3.54 – 3.48 (m, 2H), 3.41 – 3.28 (m, 4H), 2.28 (d, *J* = 1.3 Hz, 1H), 2.26 (s, 3H), 1.70 – 1.63 (m, 1H), 1.49 – 1.40 (m, 13H). ^{19}F NMR (471 MHz, $CDCl_3$) δ -121.33. ^{13}C NMR (126 MHz, $CDCl_3$) δ 198.0, 160.0 (d, *J* = 30.4 Hz), 156.1 (d, *J* = 269.9 Hz), 154.9, 142.7, 134.4, 99.7 (d, *J* = 14.9 Hz), 79.9, 44.9, 38.7, 37.2, 32.7, 28.6, 27.37, 27.36. HRMS (ESI/Q-TOF): m/z [M + H]$^+$ calcd for $C_{19}H_{30}FN_2O_4$ [M+H]$^+$: 369.2184 found: 369.2176.

(*E*)-2-fluoro-*N*-(6-oxohept-4-en-1-yl)acrylamide (2k).

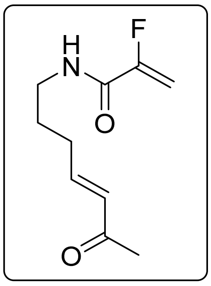

2k

By means of the general procedure described above, enone **2k** (292 mg, 92% yield) was obtained as a brown oil starting from 250 mg (1.59 mmol) of **1i** after flash chromatography with 2:1 n-hexane: ethyl acetate. ^{1}H NMR (300 MHz, $CDCl_3$) δ 6.76 (dt, *J* = 16.0, 6.8 Hz, 1H), 6.48 (bs, 1H), 6.08 (dt, *J* = 15.9, 1.5 Hz, 1H), 5.65 (dd, *J* = 47.9, 3.2 Hz, 1H), 5.09 (dd, *J* = 15.4, 3.2 Hz, 1H), 3.37 (q, *J* = 6.7 Hz, 2H), 2.31 – 2.23 (m, 2H), 2.22 (s, 3H), 1.74 (p, *J* = 7.4 Hz, 2H). ^{19}F NMR (282 MHz, $CDCl_3$) δ -121.63. ^{13}C NMR (75 MHz, $CDCl_3$) δ 198.5, 159.8 (d, *J* = 30.8 Hz), 156.4 (d, *J* = 270.1 Hz), 146.6, 131.9, 99.0 (d, *J* = 14.9 Hz), 38.9, 29.8, 28.0, 27.1. HRMS (ESI/Q-TOF): m/z [M + H]$^+$ calcd for $C_{10}H_{15}FNO_2$ [M+H]$^+$: 200.1081 found: 200.1081

***(E)*-2-fluoro-*N*-(6-oxo-2,2-diphenylhept-4-en-1-yl)acrylamide (2l).**

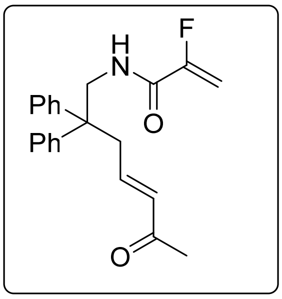

2l

By means of the general procedure described above, enone **2l** (253 mg, 89% yield) was obtained as a brown oil starting from 250 mg (0.81 mmol) of **1h** after flash chromatography with 5:1 n-hexane: ethyl acetate. ^{1}H NMR (300 MHz, $CDCl_3$) δ 7.45 – 7.38 (m, 4H), 7.37 – 7.31 (m, 2H), 7.27 – 7.23 (m, 4H), 6.52 (dt, *J* = 15.9, 7.4 Hz, 1H), 5.99 (dt, *J* = 15.9, 1.3 Hz, 1H), 5.91 (bs, 1H), 5.72 (dd, *J* = 47.8, 3.3 Hz, 1H), 5.14 (dd, *J* = 15.3, 3.3 Hz, 1H), 4.13 (d, *J* = 6.0 Hz, 2H), 3.06 (dd, *J* = 7.4, 1.3 Hz, 2H), 2.15 (s, 3H). ^{19}F NMR (282 MHz, $CDCl_3$) δ -122.12. ^{13}C NMR (75 MHz, $CDCl_3$) δ 198.2, 159.6 (d, *J* = 30.6 Hz), 156.0 (d, *J* = 270.2 Hz), 144.2, 143.0, 134.6, 128.8, 127.8, 127.3, 99.4 (d, *J* = 14.9 Hz), 51.0, 46.1, 40.6, 26.8. HRMS (ESI/Q-TOF): m/z $[M + H]^+$ calcd for $C_{22}H_{23}FNO_2$ $[M+H]^+$: 352.1707 found: 352.1715.

(*E*)-2-fluoro-N-(2-(4-oxopent-2-en-1-yl)phenyl)acrylamide (2m).

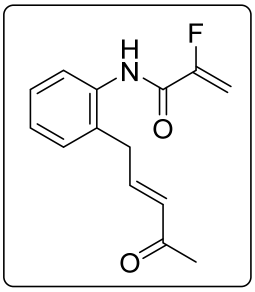

2m

By means of the general procedure described above, enone **2m** (144 mg, 75% yield) was obtained as a brown oil starting from 160 mg (0.78 mmol) of **1j** after flash chromatography with 4:1 n-hexane: ethyl acetate. ^{1}H NMR (500 MHz, $CDCl_3$) δ 7.83 – 7.80 (m, 2H), 7.79 – 7.30 (m, 1H), 7.23 – 7.21 (m, 2H), 6.87 (dt, *J* = 16.0, 6.4 Hz, 1H), 6.09 (dt, *J* = 16.0, 1.7 Hz, 1H), 5.82 (dd, *J* = 48.1, 3.4 Hz, 1H), 5.26 (dd, *J* = 15.4, 3.4 Hz, 1H), 3.56 (dd, *J* = 6.5, 1.7 Hz, 2H), 2.24 (s, 3H). ^{19}F NMR (282 MHz, $CDCl_3$) δ -121.40. ^{13}C NMR (126 MHz, $CDCl_3$) δ 198.0, 157.7 (d, *J* = 29.6 Hz), 156.3 (d, *J* = 270.9 Hz), 144.1, 134.5, 132.5, 130.6, 129.9, 128.4, 126.9, 124.6, 100.4 (d, *J* = 14.8 Hz), 35.2, 27.4. HRMS (ESI/Q-TOF): m/z $[M + H]^+$ calcd for $C_{14}H_{15}FNO_2$ $[M+H]^+$: 248.1081 found: 248.1082.

(*E*)-2-fluoro-N-(4-methyl-2-(4-oxopent-2-en-1-yl)phenyl)acrylamide (2n).

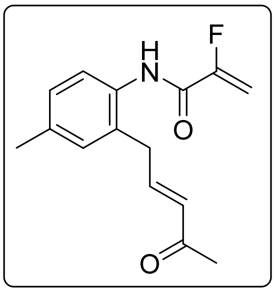

2n

By means of the general procedure described above, enone **2n** (320 mg, 79% yield) was obtained as a brown oil starting from 341 mg (1.55 mmol) of **1k** after flash chromatography with 4:1 n-hexane: ethyl acetate. ^{1}H NMR (500 MHz, $CDCl_3$) δ 7.77 (bs, 1H), 7.59 (d, *J* = 8.1 Hz, 1H), 7.11 (dd, *J* = 8.1, 2.1 Hz, 1H), 7.01 (d, *J* = 2.1 Hz, 1H), 6.85 (dt, *J* = 16.0, 6.5 Hz, 1H), 6.06 (dt, *J* = 16.0, 1.7 Hz, 1H), 5.78 (dd, *J* = 48.1, 3.4 Hz, 1H), 5.23 (dd, *J* = 15.4, 3.4 Hz, 1H), 3.49 (dd, *J* = 6.5, 1.8 Hz, 2H), 2.33 (s, 3H), 2.23 (s, 3H). ^{19}F NMR (471 MHz, $CDCl_3$) δ -120.78. ^{13}C NMR (75 MHz, $CDCl_3$) δ 198.2, 157.9 (d, *J* = 29.9 Hz), 156.3 (d, *J* = 271.2 Hz), 144.5, 136.9, 132.3, 131.7, 131.1, 130.3, 128.9, 124.9, 100.1 (d, *J* = 14.8 Hz), 35.1, 27.3, 21.1. HRMS (ESI/Q-TOF): m/z $[M + H]^+$ calcd for $C_{15}H_{17}FNO_2$ $[M+H]^+$: 262.1238 found: 262.1246.

(*E*)-2-fluoro-N-(4-methoxy-2-(4-oxopent-2-en-1-yl)phenyl)acrylamide (2o).

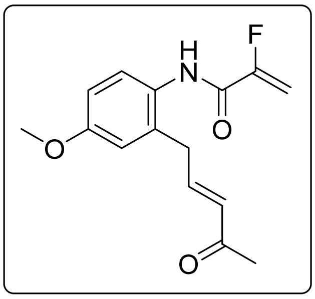

2o

By means of the general procedure described above, enone **2o** (501 mg, 79% yield) was obtained as a brown oil starting from 341 mg (2.41 mmol) of **1l** after flash chromatography with 2:1 n-hexane: ethyl acetate. ^{1}H NMR (300 MHz, $CDCl_3$) δ 7.70 (bs, 1H), 7.54 (d, *J* = 8.8 Hz, 1H), 6.89 – 6.79 (m, 2H), 6.74 (d, *J* = 2.9 Hz, 1H), 6.06 (dt, *J* = 16.0, 1.7 Hz, 1H), 5.78 (dd, *J* = 48.1, 3.3 Hz, 1H), 5.23 (dd, *J* = 15.4, 3.3 Hz, 1H), 3.80 (s, 3H), 3.49 (dd, *J* = 6.5, 1.7 Hz, 2H), 2.23 (s, 3H). ^{19}F NMR (282 MHz, $CDCl_3$) δ -121.23. ^{13}C NMR (75 MHz, $CDCl_3$) δ 198.2, 158.4, 158.1 (d, *J* = 29.7 Hz), 156.3 (d, *J* = 270.9 Hz), 144.3, 132.9, 132.4, 127.00, 126.98 116.1, 112.9, 100.1 (d, *J* = 14.9 Hz), 55.6, 35.3, 27.3. HRMS (ESI/Q-TOF): m/z $[M + H]^+$ calcd for $C_{15}H_{17}FNO_3$ $[M+H]^+$: 278.1187 found: 278.1189.

(*E*)-*N*-(2,2-dimethyl-6-oxohept-4-en-1-yl)-2-(trifluoromethyl)acrylamide (2p).

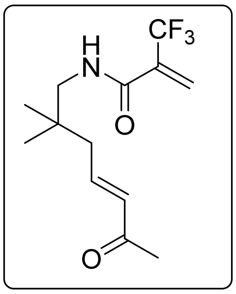

2p

By means of the general procedure described above, enone **2p** (271 mg, 46% yield) was obtained as a brown oil starting from 500 mg (2.12 mmol) of **1m** after flash chromatography with 3:1 n-hexane: ethyl acetate. ^{1}H NMR (300 MHz, $CDCl_3$) δ 6.82 (dt, *J* = 15.6, 7.8 Hz, 1H), 6.59 (q, *J* = 1.7 Hz, 1H), 6.26 (q, *J* = 1.5 Hz, 1H), 6.09 (dt, *J* = 15.8, 1.3 Hz, 1H), 5.99 (bs, 1H), 3.25 (d, *J* = 6.4 Hz, 2H), 2.25 (s, 3H), 2.15 (dd, *J* = 7.8, 1.3 Hz, 2H), 0.96 (s, 6H). ^{19}F NMR (282 MHz, $CDCl_3$) δ -63.56. ^{13}C NMR (75 MHz, $CDCl_3$) δ 198.3, 161.0, 143.8, 134.0, 133.7 (q, *J* = 31.1 Hz), 130.0 (q, *J* = 5.7 Hz), 122.5 (q, *J* = 271.3 Hz), 49.4, 42.95, 35.8, 27.3, 25.1. HRMS (ESI/Q-TOF): m/z $[M + H]^+$ calcd for $C_{13}H_{19}F_3NO_2$ $[M+H]^+$: 278.1362 found: 278.1358.

GENERAL PROCEDURE FOR THE INTRAMOLECULAR AZA-MICHAEL REACTION. SYNTHESIS OF PYRROLIDINES 3:

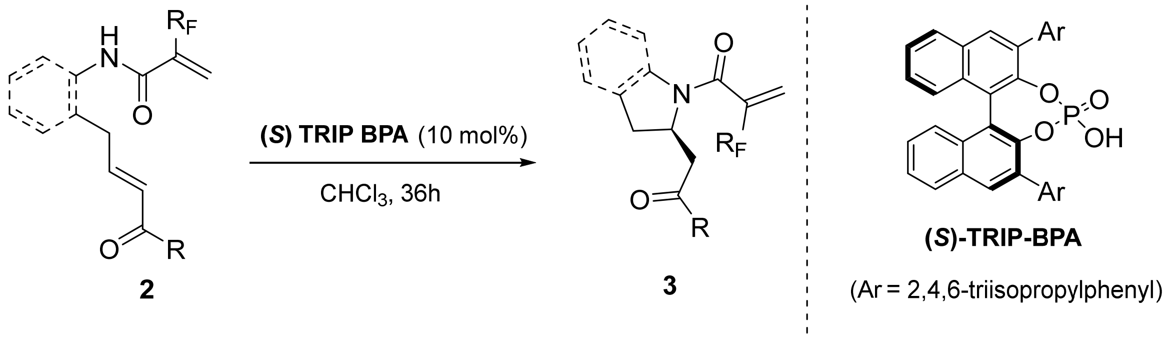

In a round bottomed flask, the corresponding enone **2** was dissolved in chloroform (0.1 M). Then 10 mol% of (*S*)-TRIP BPA was added and the resulting solution was stirred at room temperature for 36 hours (monitored by TLC). The solvent was removed under reduced pressure and the residue was chromatographed on silica gel.

(*R*)-1-(4,4-dimethyl-2-(2-oxopropyl)pyrrolidin-1-yl)-2-fluoroprop-2-en-1-one (3a).

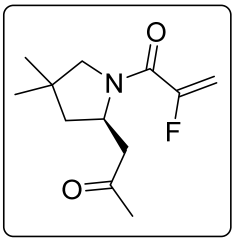

3a

By means of the general procedure described above, pyrrolidine **3a** (86 mg, 86% yield) was obtained from 100 mg (0.44 mmol) of **2a** as a white solid in 95% ee after flash chromatography with 4:1 n-hexane: ethyl acetate. M. p. = 38-39 °C. The ee value was determined by HPLC analysis using a Phenomenex Celulose 2 column (hexane: isopropanol 95:5); flow rate = 1.0 mL/min, t_{major}= 23.2 min, t_{minor}= 21.3 min. $[\alpha]_D^{25}$ = -70.0 (*c* 1.0, $CHCl_3$). ^1H NMR (300 MHz, $CDCl_3$) δ 5.41 (dd, *J* = 46.7, 3.2 Hz, 1H), 5.09 (dd, *J* = 16.0, 3.2 Hz, 1H), 4.44 – 4.35 (m, 1H), 3.39 – 3.25 (m, 3H), 2.54 (dd, *J* = 17.1, 8.6 Hz, 1H), 2.13 (s, 3H), 2.12 – 2.04 (m, 1H), 1.39 (dd, *J* = 12.7, 9.9 Hz, 1H), 1.10 (s, 3H), 0.98 (s, 3H). ^{19}F NMR (282 MHz, $CDCl_3$) δ -108.80. ^{13}C NMR (75 MHz, $CDCl_3$) δ 206.8, 160.8 (d, *J* = 30.4 Hz), 157.8 (d, *J* = 272.0 Hz), 100.0 (d, *J* = 15.8 Hz), 61.1 (d, *J* = 9.1 Hz), 54.4, 47.1, 44.9, 38.2 (d, J = 2.8 Hz), 30.5, 25.9, 25.8. HRMS (EI) calcd for $C_{12}H_{19}FNO_2$ $[M+H]^+$: 228.1394 found: 228.1395.

(*R*)-1-(1-(2-fluoroacryloyl)-4,4-dimethylpyrrolidin-2-yl)pentan-2-one (3b).

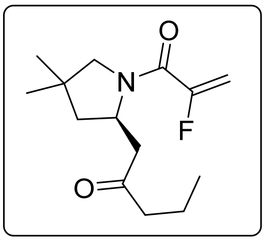

3b

By means of the general procedure described above, pyrrolidine **3b** (99 mg, 99% yield) was obtained from 100 mg (0.39 mmol) of **2n** as a colorless oil in 96% ee after flash chromatography with 4:1 n-hexane: ethyl acetate. The ee value was determined by HPLC analysis using a Chiralpak IC column (hexane: isopropanol 95:5); flow rate = 1.0 mL/min, t_{major}= 28.8 min, t_{minor}= 21.8 min. $[\alpha]_D^{25}$ = -72.3 (*c* 2.0, $CHCl_3$). ^1H NMR (300 MHz, $CDCl_3$) δ 5.41 (dd, *J* = 46.7, 3.2 Hz, 1H), 5.10 (dd, *J* = 16.0, 3.2 Hz, 1H), 4.45 – 4.35 (m, 1H), 3.39 – 3.30 (m, 2H), 3.26 (dd, *J* = 17.0, 3.3 Hz, 1H), 2.52 (dd, *J* = 16.9, 8.8 Hz, 1H), 2.39 – 2.33 (m, 2H), 2.07 (dd, *J* = 12.6, 7.3 Hz, 1H), 1.58 (h, *J* = 7.4 Hz, 2H), 1.41 (dd, *J* = 12.6, 10.0 Hz, 1H), 1.10 (s, 3H), 0.98 (s, 3H), 0.90 (t, *J* = 7.4 Hz, 3H). ^{19}F NMR (282 MHz, $CDCl_3$) δ -109.22. ^{13}C NMR (75 MHz, $CDCl_3$) δ 209.3, 160.7 (d, *J* = 30.5 Hz), 157.9 (d, *J* = 272.0 Hz), 99.9 (d, *J* = 15.7 Hz), 61.2 (d, *J* = 8.9 Hz), 54.5, 46.1, 45.3, 44.9, 38.2 (d, *J* = 2.5 Hz), 25.9, 25.8, 17.3, 13.8. HRMS (ESI/Q-TOF): m/z $[M + H]^+$ calcd for $C_{14}H_{23}FNO_2$ $[M+H]^+$: 256.1707 found: 256.1710.

(*R*)-1-(1-(2-fluoroacryloyl)-4,4-dimethylpyrrolidin-2-yl)heptan-2-one (3c).

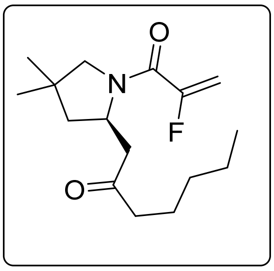

3c

By means of the general procedure described above, pyrrolidine **3c** (99 mg, 99% yield) was obtained from 100 mg (0.35 mmol) of **2o** as a colorless oil in 96% ee after flash chromatography with 6:1 n-hexane: ethyl acetate. The ee value was determined by HPLC analysis using a Chiralpak IC column (hexane: isopropanol 95:5); flow rate = 1.0 mL/min, t_{major}= 24.2 min, t_{minor}= 19.4 min. $[\alpha]_D^{25}$ = -70.7 (*c* 3.0, $CHCl_3$). ^1H NMR (300 MHz, $CDCl_3$) δ 5.40 (dd, *J* = 46.7, 3.2 Hz, 1H), 5.09 (dd, *J* = 16.0, 3.2 Hz, 1H), 4.42 – 4.37 (m, 1H), 3.40 – 3.34 (m, 2H), 3.25 (dd, *J* = 17.0, 3.3 Hz, 1H), 2.53 (dd, *J* = 17.0, 8.8 Hz, 1H), 2.43 – 2.31 (m, 2H), 2.07 (dd, *J* = 12.7, 7.3 Hz, 1H), 1.55 (p, *J* = 7.5 Hz, 2H), 1.40 (dd, *J* = 12.7, 9.9 Hz, 1H), 1.31 – 1.20 (m, 4H), 1.10 (s, 3H), 0.97 (s, 3H), 0.87

(t, J = 6.9 Hz, 3H). ^{19}F NMR (282 MHz, $CDCl_3$) δ -109.21. ^{13}C NMR (75 MHz, $CDCl_3$) δ 209.4, 160.7 (d, J = 30.6 Hz), 157.8 (d, J = 272.2 Hz), 99.9 (d, J = 15.7 Hz), 61.2 (d, J = 8.8 Hz), 54.5, 46.0, 44.9, 43.4, 38.2 (d, J = 2.6 Hz), 31.5, 25.9, 25.8, 23.6, 22.6, 14.0.HRMS (ESI/Q-TOF): m/z $[M + H]^+$ calcd for $C_{16}H_{27}FNO_2$ $[M+H]^+$: 284.2020 found: 284.2028.

(*R*)-1-(4,4-dimethyl-2-(2-oxo-2-phenylethyl)pyrrolidin-1-yl)-2-fluoroprop-2-en-1-one (3d).

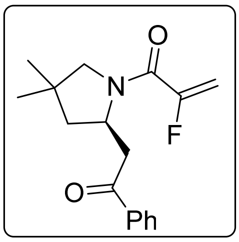

3d

By means of the general procedure described above, pyrrolidine **3d** (99 mg, 99% yield) was obtained from 100 mg (0.35 mmol) of **2p** as a white solid in 95% ee after flash chromatography with 6:1 n-hexane: ethyl acetate. The ee value was determined by HPLC analysis using a Chiralpak IC column (hexane: isopropanol 95:5); flow rate = 1.0 mL/min, t_{major}= 48.8 min, t_{minor}= 15.7 min. $[\alpha]_D^{25}$ = -56.0 (c 2.0, $CHCl_3$). M. p. = 58-60 °C. ^{1}H NMR (300 MHz, $CDCl_3$) δ 8.04 – 8.00 (m, 2H), 7.59 – 7.53 (m, 1H), 7.48 – 7.43 (m, 2H), 5.45 (dd, J = 46.6, 3.2 Hz, 1H), 5.13 (dd, J = 16.0, 3.2 Hz, 1H), 4.64 – 4.54 (m, 1H), 3.99 (dd, J = 15.8, 3.2 Hz, 1H), 3.44 – 3.34 (m, 2H), 2.90 (dd, J = 15.8, 9.5 Hz, 1H), 2.10 (dd, J = 12.8, 7.3 Hz, 1H), 1.53 (dd, J = 12.8, 9.8 Hz, 1H), 1.12 (s, 3H), 0.99 (s, 3H). ^{19}F NMR (282 MHz, $CDCl_3$) δ -109.19. ^{13}C NMR (75 MHz, $CDCl_3$) δ 198.5, 160.9 (d, J = 30.7 Hz), 157.8 (d, J = 271.9 Hz), 136.8, 133.4, 128.8, 128.4, 100.1 (d, J = 15.8 Hz), 61.2 (d, J = 8.9 Hz), 55.3, 44.7, 42.8, 38.3 (d, J = 2.6 Hz), 26.0, 25.9. HRMS (ESI/Q-TOF): m/z $[M + H]^+$ calcd for $C_{17}H_{21}FNO_2$ $[M+H]^+$: 290.1551 found: 290.1562.

(*R*)-2-fluoro-1-(6-(2-oxopropyl)-5-azaspiro[2.4]heptan-5-yl)prop-2-en-1-one (3e).

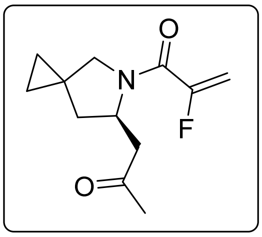

3e

By means of the general procedure described above, pyrrolidine **3e** (53 mg, 66% yield) was obtained from 80 mg (0.35 mmol) of **2b** as a colorless oil in 91% ee after flash chromatography with 5:1 n-hexane: ethyl acetate. The ee value was determined by HPLC analysis using a

Phenomenex Amilose 1 column (hexane: isopropanol 90:10); flow rate = 1.0 mL/min, t_{major}= 10.3 min, t_{minor}= 9.4 min. $[\alpha]_D^{25}$ = -12.7 (*c* 1.0, $CHCl_3$). NMR (300 MHz, $CDCl_3$) δ 5.49 (dd, *J* = 46.7, 3.1 Hz, 1H), 5.10 (dd, *J* = 16.1, 3.1 Hz, 1H), 4.64 – 4.56 (m, 1H), 3.57 (dd, *J* = 11.0, 3.7 Hz, 1H), 3.49 (dd, *J* = 10.9, 5.5 Hz, 1H), 3.28 (dd, *J* = 16.9, 3.5 Hz, 1H), 2.64 (dd, *J* = 16.8, 9.5 Hz, 1H), 2.16 (s, 3H), 1.62 – 1.56 (m, 2H), 0.68 – 0.56 (m, 4H). ^{19}F NMR (282 MHz, $CDCl_3$) δ -110.95. ^{13}C NMR (75 MHz, $CDCl_3$) δ 207.1, 160.0 (d, *J* = 26.2 Hz), 157.9 (d, *J* = 268.4 Hz), 100.4 (d, *J* = 15.8 Hz), 55.8 (d, *J* = 4.4 Hz), 46.9, 38.3, 30.4, 29.8, 21.1 (d, *J* = 4.3 Hz), 11.3, 9.5.HRMS (ESI/Q-TOF): m/z $[M + H]^+$ calcd for $C_{12}H_{17}FNO_2$ $[M+H]^+$: 226.1238 found: 226.1230.

(*R*)-2-fluoro-1-(7-(2-oxopropyl)-6-azaspiro[3.4]octan-6-yl)prop-2-en-1-one (3f).

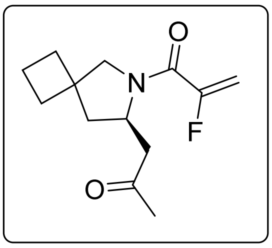

3f

By means of the general procedure described above, pyrrolidine **3f** (69 mg, 86% yield) was obtained from 80 mg (0.33 mmol) of **2c** as a white solid in 90% ee after flash chromatography with 5:1 n-hexane: ethyl acetate. M. p. = 30.5-32 °C. The ee value was determined by HPLC analysis using a Chiralpak IC column (hexane: isopropanol 90:10); flow rate = 1.0 mL/min, t_{major}= 32.9 min, t_{minor}= 51.4 min. $[\alpha]_D^{25}$ = -70.1 (*c* 3.0, $CHCl_3$). NMR (300 MHz, $CDCl_3$) δ 5.44 (dd, *J* = 46.7, 3.1 Hz, 1H), 5.11 (dd, *J* = 16.1, 3.1 Hz, 1H), 4.36 – 4.27 (m, 1H), 3.63 (dd, *J* = 11.0, 6.1 Hz, 1H), 3.52 (dd, *J* = 11.0, 2.2 Hz, 1H), 3.29 (dd, *J* = 17.0, 3.5 Hz, 1H), 2.48 (dd, *J* = 17.0, 8.9 Hz, 1H), 2.33 (dd, *J* = 12.7, 7.3 Hz, 1H), 2.14 (s, 3H), 2.04 – 1.96 (m, 2H), 1.94 – 1.86 (m, 4H), 1.57 (dd, *J* = 12.7, 8.4 Hz, 1H). ^{19}F NMR (282 MHz, $CDCl_3$) δ -109.84. ^{13}C NMR (75 MHz, $CDCl_3$) δ 206.9, 160.6 (d, *J* = 30.8 Hz), 157.9 (d, *J* = 272.5 Hz), 100.1 (d, *J* = 15.8 Hz), 59.6 (d, *J* = 9.9 Hz), 54.3, 46.9, 44.5 (d, *J* = 3.1 Hz), 42.8, 33.0, 30.5, 29.5, 16.1. HRMS (ESI/Q-TOF): m/z $[M + H]^+$ calcd for $C_{13}H_{19}FNO_2$ $[M+H]^+$: 240.1394 found: 240.1389.

(*R*)-2-fluoro-1-(3-(2-oxopropyl)-2-azaspiro[4.4]nonan-2-yl)prop-2-en-1-one (3g).

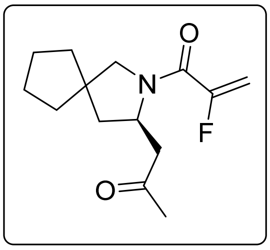

3g

By means of the general procedure described above, pyrrolidine **3g** (84 mg, 93% yield) was obtained from 90 mg (0.36 mmol) of **2d** as a white solid in 93% ee after flash chromatography with 5:1 n-hexane: ethyl acetate. M. p. = 44-46 °C. The ee value was determined by HPLC analysis using a Chiralpak IC column (hexane: isopropanol 90:10); flow rate = 1.0 mL/min, t_{major}= 35.7 min, t_{minor}= 30.6 min. $[\alpha]_D^{25}$ = -49.5 (*c* 2.0, $CHCl_3$). ^{1}H NMR (300 MHz, $CDCl_3$) δ 5.42 (dd, *J* = 46.7, 3.2 Hz, 1H), 5.10 (dd, *J* = 16.0, 3.2 Hz, 1H), 4.38 – 4.28 (m, 1H), 3.43 – 3.40 (m, 2H), 3.32 (dd, *J* = 17.1, 3.5 Hz, 1H), 2.53 (dd, *J* = 17.1, 8.7 Hz, 1H), 2.21 – 2.14 (m, 1H), 2.14 (s, 3H), 1.65 – 1.56 (m, 6H), 1.53 – 1.42 (m, 3H). ^{19}F NMR (282 MHz, $CDCl_3$) δ -109.37. ^{13}C NMR (75 MHz, $CDCl_3$) δ 206.9, 160.6 (d, *J* = 30.7 Hz), 157.9 (d, *J* = 272.3 Hz), 100.0 (d, *J* = 15.8 Hz), 59.7 (d, *J* = 9.2 Hz), 54.7, 49.2 (d, *J* = 2.7 Hz), 47.1, 43.4, 37.3, 35.6, 30.5, 24.95, 24.90. HRMS (ESI/Q-TOF): m/z $[M + H]^+$ calcd for $C_{14}H_{21}FNO_2$ $[M+H]^+$: 254.1551 found: 254.1544.

(*R*)-2-fluoro-1-(3-(2-oxopropyl)-2-azaspiro[4.5]decan-2-yl)prop-2-en-1-one (3h).

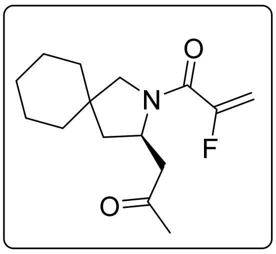

3h

By means of the general procedure described above, pyrrolidine **3h** (75 mg, 94% yield) was obtained from 80 mg (0.3 mmol) of **2e** as a colorless oil in 96% ee after flash chromatography with 5:1 n-hexane: ethyl acetate. The ee value was determined by HPLC analysis using a Chiralpak IC column (hexane: isopropanol 90:10); flow rate = 1.0 mL/min, t_{major}= 34.6 min, t_{minor}= 31.3 min. $[\alpha]_D^{25}$ = -49.1 (*c* 3.0, $CHCl_3$). ^{1}H NMR (300 MHz, $CDCl_3$) δ 5.40 (dd, *J* = 46.7, 3.2 Hz, 1H), 5.10 (dd, *J* = 16.0, 3.2 Hz, 1H), 4.40 – 4.31 (m, 1H), 3.56 (dd, *J* = 10.3, 7.9 Hz, 1H), 3.31 – 3.22 (m, 1H), 2.52 (dd, *J* = 17.0, 8.7 Hz, 1H), 2.26 (dd, *J* = 12.7, 7.6 Hz, 1H), 2.13 (s, 3H), 1.50 – 1.24 (m,

12H). ^{19}F NMR (282 MHz, $CDCl_3$) δ -109.01. ^{13}C NMR (75 MHz, $CDCl_3$) δ 206.9, 160.8 (d, *J* = 30.7 Hz), 157.9 (d, *J* = 272.1 Hz), 99.9 (d, *J* = 15.7 Hz), 58.8 (d, *J* = 9.1 Hz), 53.6, 47.3, 42.2, 42.1 (d, *J* = 2.5 Hz), 36.3, 33.8, 30.5, 26.2, 23.8, 22.7. HRMS (ESI/Q-TOF): m/z $[M + H]^+$ calcd for $C_{15}H_{23}FNO_2$ $[M+H]^+$: 268.1707 found: 268.1705.

(*R*)-2-fluoro-1-(3-(2-oxopropyl)-8-oxa-2-azaspiro[4.5]decan-2-yl)prop-2-en-1-one (3i).

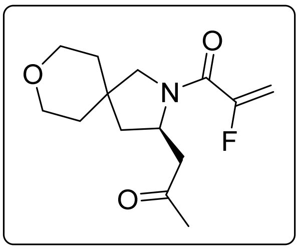

3i

By means of the general procedure described above, pyrrolidine **3i** (89 mg, 89% yield) was obtained from 100 mg (0.37 mmol) of **2f** as a white solid in 98% ee after flash chromatography with 1:2 n-hexane: ethyl acetate. M. p. = 35-36.5 °C. The ee value was determined by HPLC analysis using a Chiralpak IC column (hexane: isopropanol 70:30); flow rate = 1.0 mL/min, t_{major}= 28.9 min, t_{minor}= 21.4 min. $[\alpha]_D^{25}$ = -61.7 (*c* 3.0, $CHCl_3$). ^{1}H NMR (300 MHz, $CDCl_3$) δ 5.42 (dd, *J* = 46.7, 3.2 Hz, 1H), 5.12 (dd, *J* = 16.0, 3.2 Hz, 1H), 4.41 – 4.31 (m, 1H), 3.69 – 3.59 (m, 4H), 3.34 – 3.25 (m, 2H), 2.57 (dd, *J* = 17.4, 8.6 Hz, 1H), 2.41 (dd, *J* = 12.9, 7.4 Hz, 1H), 2.13 (s, 3H), 1.60 – 1.54 (m, 3H), 1.49 – 1.43 (m, 2H), 1.35 (dd, *J* = 12.9, 9.9 Hz, 1H). ^{19}F NMR (282 MHz, $CDCl_3$) δ -109.39. ^{13}C NMR (75 MHz, $CDCl_3$) δ 206.7, 160.7 (d, *J* = 31.0 Hz), 157.6 (d, *J* = 272.3 Hz), 100.3 (d, *J* = 15.6 Hz), 65.4, 64.6, 58.5 (d, *J* = 9.5 Hz), 53.4, 47.0, 41.4, 39.8, 35.6, 34.2, 30.5. HRMS (ESI/Q-TOF): m/z $[M + H]^+$ calcd for $C_{14}H_{21}FNO_3$ $[M+H]^+$: 270.1500 found: 270.1503.

(*R*)-tert-butyl 2-(2-fluoroacryloyl)-3-(2-oxopropyl)-2,8-diazaspiro[4.5]decane-8-carboxylate (3j)

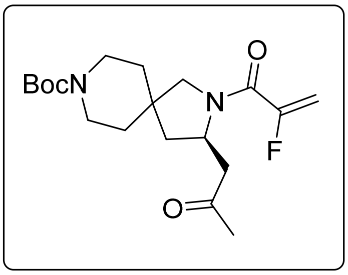

3j

By means of the general procedure described above, pyrrolidine **3j** (67 mg, 79% yield) was obtained from 85 mg (0.23 mmol) of **2g** as a colorless oil in 98% ee after flash chromatography with 1:1 n-hexane: ethyl acetate. The ee value was determined by HPLC analysis using a

Chiralpak IC column (hexane: isopropanol 50:50); flow rate = 1.0 mL/min, t_{major}= 53.5 min, t_{minor}= 37.4 min. $[\alpha]_D^{25}$ = -35.3 (*c* 3.0, $CHCl_3$). ^{1}H NMR (500 MHz, $CDCl_3$) ^{1}H NMR (500 MHz, Chloroform-*d*) δ 5.43 (dd, *J* = 46.7, 3.2 Hz, 1H), 5.12 (dd, *J* = 16.0, 3.3 Hz, 1H), 4.40 – 4.34 (m, 1H), 3.61 – 3.57 (m, 1H), 3.41 – 3.27 (m, 5H), 2.58 (dd, *J* = 17.3, 8.6 Hz, 1H), 2.34 (dd, *J* = 12.7, 7.5 Hz, 1H), 2.14 (s, 3H), 1.54 – 1.50 (m, 2H), 1.44 (s, 9H), 1.41 – 1.34 (m, 4H). ^{19}F NMR (282 MHz, $CDCl_3$) δ -109.35. ^{13}C NMR (75 MHz, $CDCl_3$) δ 206.7, 161.5, 160.7 (d, *J* = 30.7 Hz), 157.6 (d, *J* = 272.3 Hz), 154.9, 100.3 (d, *J* = 15.5 Hz), 79.8, 58.1 (d, *J* = 9.5 Hz), 53.5, 47.0, 40.9, 40.70, 40.66, 35.1, 33.2, 30.6, 28.6. HRMS (ESI/Q-TOF): m/z $[M + H]^+$ calcd for $C_{19}H_{30}FN_2O_4$ $[M+H]^+$: 369.2184 found: 369.2179.

(*R*)-2-fluoro-1-(2-(2-oxopropyl)pyrrolidin-1-yl)prop-2-en-1-one (3k).

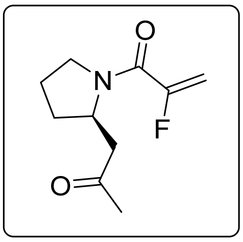

3k

By means of the general procedure described above, pyrrolidine **3k** (97 mg, 97% yield) was obtained from 100 mg (0.50 mmol) of **2i** as a colorless oil in 79% ee after flash chromatography with 4:1 n-hexane: ethyl acetate. The ee value was determined by HPLC analysis using a Phenomenex Amilose 1 column (hexane: isopropanol 85:15); flow rate = 1.0 mL/min, t_{major}= 9.4 min, t_{minor}= 8.4 min. $[\alpha]_D^{25}$ = -42.7 (*c* 3.0, $CHCl_3$). ^{1}H NMR (300 MHz, $CDCl_3$) δ 5.44 (dd, *J* = 46.7, 3.1 Hz, 1H), 5.09 (dd, *J* = 16.1, 3.1 Hz, 1H), 4.43 – 4.35 (m, 1H), 3.66 – 3.60 (m, 2H), 3.21 (dd, *J* = 16.8, 3.4 Hz, 1H), 2.45 (dd, *J* = 16.8, 9.2 Hz, 1H), 2.19 (dd, *J* = 13.4, 6.5 Hz, 1H), 2.14 (s, 3H), 1.96 – 1.85 (m, 2H), 1.67 – 1.59 (m, 1H). ^{19}F NMR (282 MHz, $CDCl_3$) δ -110.30. ^{13}C NMR (75 MHz, $CDCl_3$) δ 206.9, 160.2 (d, *J* = 31.1 Hz), 158.0 (d, *J* = 272.6 Hz), 100.1 (d, *J* = 16.0 Hz), 55.0, 48.2 (d, *J* = 11.6 Hz), 46.7, 30.4, 30.2, 24.8 (d, *J* = 3.7 Hz). HRMS (ESI/Q-TOF): m/z $[M + H]^+$ calcd for $C_{10}H_{15}FNO_2$ $[M+H]^+$: 200.1081 found: 200.1082.

(*R*)-2-fluoro-1-(2-(2-oxopropyl)-4,4-diphenylpyrrolidin-1-yl)prop-2-en-1-one (3l).

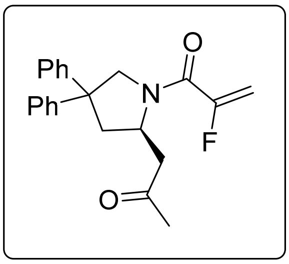

3l

By means of the general procedure described above, pyrrolidine **3l** (62 mg, 52% yield) was obtained from 120 mg (0.34 mmol) of **2h** as a colorless oil in 71% ee after flash chromatography with 5:1 n-hexane: ethyl acetate. The ee value was determined by HPLC analysis using a Chiralpak IC column (hexane: isopropanol 95:5; flow rate = 1.0 mL/min, t_{major}= 48.3 min, t_{minor}= 70.2 min. $[\alpha]_D^{25}$ = -38.5 (*c* 2.0, $CHCl_3$). ^1H NMR (300 MHz, $CDCl_3$) δ 7.24 – 7.16 (m, 5H), 7.15 – 7.07 (m, 5H), 5.38 (dd, *J* = 46.8, 3.3 Hz, 1H), 5.11 (dd, *J* = 16.1, 3.3 Hz, 1H), 4.49 (ddd, *J* = 11.6, 6.8, 2.0 Hz, 1H), 4.22 – 4.12 (m, 1H), 3.96 (d, *J* = 11.6 Hz, 1H), 3.25 (dd, *J* = 17.4, 3.4 Hz, 1H), 3.05 (ddd, *J* = 12.8, 6.8, 2.0 Hz, 1H), 2.51 (dd, *J* = 17.4, 8.7 Hz, 1H), 2.26 (dd, *J* = 12.8, 9.9 Hz, 1H), 2.04 (s, 3H). ^{19}F NMR (282 MHz, $CDCl_3$) δ -109.20. ^{13}C NMR (75 MHz, $CDCl_3$) δ 206.7, 160.5 (d, *J* = 30.7 Hz), 157.7 (d, *J* = 272.1 Hz), 145.2, 144.1, 128.9, 128.8, 126.9, 126.8, 126.7, 126.3, 100.4 (d, *J* = 15.5 Hz), 57.9 (d, *J* = 10.0 Hz), 54.0, 53.3 (d, *J* = 2.4 Hz), 46.5, 42.6, 30.6.HRMS (ESI/Q-TOF): m/z [M + H]$^+$ calcd for $C_{22}H_{23}FNO_2$ [M+H]$^+$: 352.1707 found: 352.1691.

(*R*)-2-fluoro-1-(2-(2-oxopropyl)indolin-1-yl)prop-2-en-1-one (3m).

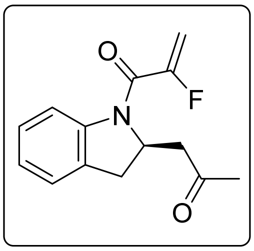

3m

By means of the general procedure described above, pyrrolidine **3m** (36 mg, 56% yield) was obtained from 64 mg (0.26 mmol) of **2j** as a yellow solid in 91% ee after flash chromatography with 6:1 n-hexane: ethyl acetate. M. p. = 81-82 °C. The ee value was determined by HPLC analysis using a Chiralpak IC column (hexane: isopropanol 90:10); flow rate = 1.0 mL/min, t_{major}= 18.5 min, t_{minor}= 21.9 min. $[\alpha]_D^{25}$ = +123.6 (*c* 1.0, $CHCl_3$). ^1H NMR (500 MHz, DMSO-d_6) δ 7.87 – 7.68 (m, 1H), 7.31 – 7.29 (m, 1H), 7.24 – 7.21 (m, 1H), 7.12 – 7.09 (m, *J* = 7.4, 1H), 5.54 (dd, *J* = 49.3, 3.8 Hz, 1H), 5.42 (dd, *J* = 17.6, 3.9 Hz, 1H), 5.01 – 4.96 (m, 1H), 3.40 (dd, *J* = 16.1, 8.6 Hz, 1H), 2.83 (d, *J* = 7.3 Hz, 2H), 2.72 – 2.68 (m, 1H), 2.09 (s, 3H). ^{19}F NMR (471 MHz, DMSO-d_6) δ -109.18. ^{13}C NMR (126 MHz, DMSO-d_6) δ 206.3, 158.2 (d, *J* = 32.2 Hz), 156.6 (d, *J* = 269.4 Hz), 140.9, 131.6, 127.1, 125.6, 124.8, 117.0, 100.9 (d, *J* = 14.7 Hz), 56.4 (d, *J* = 7.6 Hz), 48.1, 34.6, 30.2. HRMS (ESI/Q-TOF): m/z [M + H]$^+$ calcd for $C_{14}H_{15}FNO_2$ [M+H]$^+$: 248.1081 found: 248.1086.

(*R*)-2-fluoro-1-(5-methyl-2-(2-oxopropyl)indolin-1-yl)prop-2-en-1-one (3n).

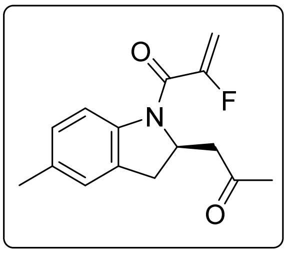

3n

By means of the general procedure described above, pyrrolidine **3n** (55 mg, 55% yield) was obtained from 100 mg (0.38 mmol) of **2k** as a yellow solid in 89% ee after flash chromatography with 5:1 n-hexane: ethyl acetate. M. p. = 108-110 °C. The ee value was determined by HPLC analysis using a Phenomenex Amilose 1 column (hexane: isopropanol 90:10); flow rate = 1.0 mL/min, t_{major}= 19.7 min, t_{minor}= 18.0 min. $[\alpha]_D^{25}$ = +148.0 (*c* 1.0, $CHCl_3$). ^1H NMR (500 MHz, DMSO-*d*$_6$) δ 7.75 – 6.99 (m, 1H), 7.13 – 6.99 (m, 2H), 5.52 (dd, *J* = 49.3, 3.9 Hz, 1H), 5.40 (dd, *J* = 17.6, 3.8 Hz, 1H), 5.01 – 4.93 (m, 1H), 3.36 (dd, *J* = 16.1, 8.6 Hz, 1H), 2.81 (d, *J* = 6.6 Hz, 2H), 2.69 – 2.58 (m, 1H), 2.27 (s, 3H), 2.09 (s, 3H). ^{19}F NMR (471 MHz, DMSO-*d*$_6$) δ -109.41. ^{13}C NMR (126 MHz, DMSO-*d*$_6$) δ 206.4, 157.9 (d, *J* = 32.1 Hz), 156.7 (d, *J* = 269.5 Hz), 138.6, 134.1, 131.7, 127.5, 126.1, 116.7, 100.8 (d, *J* = 14.7 Hz), 56.5 (d, *J* = 7.6 Hz), 48.1, 34.5, 30.2, 20.6. HRMS (ESI/Q-TOF): m/z $[M + H]^+$ calcd for $C_{15}H_{17}FNO_2$ $[M+H]^+$: 262.1238 found: 262.1246.

(*R*)-2-fluoro-1-(5-methoxy-2-(2-oxopropyl)indolin-1-yl)prop-2-en-1-one (3o).

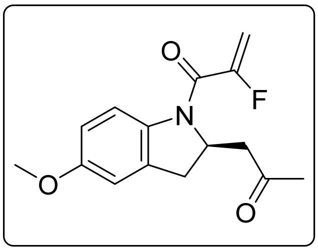

3o

By means of the general procedure described above, pyrrolidine **3o** (60 mg, 60% yield) was obtained from 100 mg (0.36 mmol) of **2l** as a yellow solid in 90% ee after flash chromatography with 5:1 n-hexane: ethyl acetate. M. p. = 85-86 °C. The ee value was determined by HPLC analysis using a Phenomenex Amilose 1 column (hexane: isopropanol 90:10); flow rate = 1.0 mL/min, t_{major}= 32.7 min, t_{minor}= 28.5 min. $[\alpha]_D^{25}$ = +116.0 (*c* 5.0, $CHCl_3$). ^1H NMR (500 MHz, DMSO-*d*$_6$) δ 7.75 – 7.47 (m, 1H), 6.91 – 6.90 (m, 1H), 6.80 – 6.77 (m, 1H), 5.52 (dd, *J* = 49.3, 3.8 Hz, 1H), 5.39 (dd, *J* = 17.6, 3.8 Hz, 1H), 4.99 – 4.97 (m, 1H), 3.73 (s, 3H), 3.38 (dd, *J* = 16.3, 8.6 Hz, 1H), 2.86 –

2.81 (m, 2H), 2.68 – 2.63 (m, 1H), 2.09 (s, 3H). ^{19}F NMR (471 MHz, DMSO-d_6) δ -109.64. ^{13}C NMR (126 MHz, DMSO-d_6) δ 206.4, 157.5 (d, *J* = 31.7 Hz), 156.8, 156.7 (d, *J* = 269.8 Hz), 134.4, 133.3, 117.9, 112.2, 111.2, 100.7 (d, *J* = 15.0 Hz), 56.6 (d, *J* = 8.0 Hz), 55.4, 48.2, 34.8, 30.2. HRMS (ESI/Q-TOF): m/z $[M + H]^+$ calcd for $C_{15}H_{17}FNO_3$ $[M+H]^+$: 278.1187 found: 278.1195.

(*R*)-1-(4,4-dimethyl-2-(2-oxopropyl)pyrrolidin-1-yl)-2-(trifluoromethyl)prop-2-en-1-one (3p).

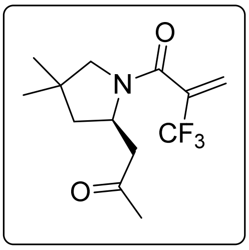

3p

By means of the general procedure described above, pyrrolidine **3p** (148 mg, 99% yield) was obtained from 150 mg (0.54 mmol) of **2m** as a white solid in 85% ee after flash chromatography with 3:1 n-hexane: ethyl acetate. M. p. =35-36 °C. The ee value was determined by HPLC analysis using a Phenomenex Celulose 4 column (hexane: isopropanol 95:5); flow rate = 1.0 mL/min, t_{major}= 19.6 min, t_{minor}= 17.0 min. $[\alpha]_D^{25}$ = -54.0 (*c* 2.0, $CHCl_3$). ^1H NMR (300 MHz, $CDCl_3$) δ 6.07 (q, *J* = 1.6 Hz, 1H), 5.71 (q, *J* = 1.2 Hz, 1H), 4.43 – 4.33 (m, 1H), 3.26 (s, 2H), 3.20 (dd, *J* = 17.2, 3.4 Hz, 1H), 2.75 (dd, *J* = 17.2, 8.1 Hz, 1H), 2.14 (s, 3H), 2.11 – 2.04 (m, 1H), 1.51 (dd, *J* = 13.4, 9.9 Hz, 1H), 1.09 (s, 3H), 0.98 (s, 3H). ^{19}F NMR (282 MHz, $CDCl_3$) δ -65.30. ^{13}C NMR (75 MHz, $CDCl_3$) δ 206.8, 163.2, 136.1 (q, *J* = 31.8 Hz), 123.5 (q, *J* = 5.3 Hz), 121.6 (q, *J* = 273.2 Hz), 62.1, 53.6, 46.6, 45.3, 38.1, 30.6, 25.574, 25.568. HRMS (ESI/Q-TOF): m/z $[M + H]^+$ calcd for $C_{13}H_{19}F_3NO_2$ $[M+H]^+$: 278.1362 found: 278.1365.

GENERAL PROCEDURE FOR THE SYNTHESIS OF INDOLIZIDINES 5:

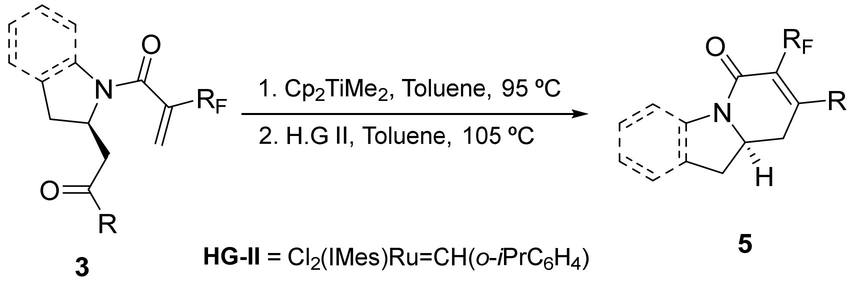

In a two neck round bottom flask, the corresponding pyrrolidine **3** was dissolved in toluene (0.1 M) under N_2 atmosphere. Then, a freshly prepared 0.5 M solution of dimethyl titanocene in toluene[5] was added (2.5 equiv.), and the reaction mixture was heated at 95 °C in a metal block for 4h (monitored by TLC). The resulting suspension was filtered through a short pad of silica gel washing with small portions of dichloromethane. The filtrate was concentrated carefully at 350 mb, obtaining a yellow oil that was employed without further purification. The crude olefine was dissolved in toluene (0.005 M), and an initial 5 mol% loading of second generation Hoveyda-Grubbs catalyst was added. The resulting mixture was heated at 105 °C in a metal block for 24h. Then it was cooled to room temperature, and one more portion of catalyst (5 mol%) was added. The reaction mixture was reheated at 105 °C for another 24 h and then concentrated to dryness and purified by means of flash column chromatography on silica gel using mixtures of n- hexane and ethyl acetate as eluents.

(*S*)-1-(4,4-dimethyl-2-(2-methylallyl)pyrrolidin-1-yl)-2-fluoroprop-2-en-1-one (4a).

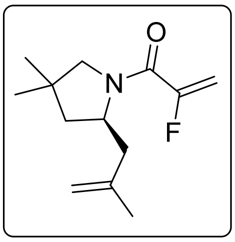

4a

By means of first part of the general procedure described above, pyrrolidine **4a** (25 mg, 56% yield) was obtained from 45 mg (0.20 mmol) of **3a** as a yellow oil after filtration on short pad column of silica gel with dichloromethane. ^{1}H NMR (300 MHz, $CDCl_3$) δ 5.42 (dd, J = 46.8, 3.1 Hz, 1H), 5.10 (dd, J = 16.1, 3.2 Hz, 1H), 4.38 – 4.29 (m, 1H), 3.40 – 3.26 (m, 2H), 2.83 (dd, J = 13.2,

[5] Synthesis of d-erythro-Dihydrosphingosine and d-xylo-Phytosphingosine from a Serine-Derived 1,5-Dioxaspiro[3.2]hexane. Ndakala, A. J.; Hashemzadeh, M.; So, R. C.; Howell, A. R. *Org. Lett.* **2002**, *4*, 1719.

3.1 Hz, 1H), 1.98 (dd, J = 13.1, 9.6 Hz, 1H), 1.85 – 1.81 (m, 1H), 1.77 (s, 3H), 1.44 (dd, J = 13.3, 10.3 Hz, 1H), 1.11 (s, 3H), 0.96 (s, 3H). ^{19}F NMR (282 MHz, $CDCl_3$) δ -108.94.

(*R*)-6-fluoro-2,2,7-trimethyl-2,3,8,8a-tetrahydroindolizin-5(1H)-one (5a).

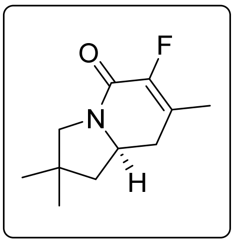

5a

By means of the general procedure described above, indolizidine **5a** (43 mg, 45% yield) was obtained from 50 mg (0.22 mmol) of **3a** as a white solid in 95% ee after flash chromatography with 1:1 n-hexane: ethyl acetate. M. p. = 75-77 °C. The ee value was determined by HPLC analysis using a Chiralpak IC column (hexane: isopropanol 80:20); flow rate = 1.0 mL/min, t_{major}= 43.2 min, t_{minor}= 51.5 min. $[\alpha]_D^{25}$ = -16.0 (*c* 1.0, $CHCl_3$). ^{1}H NMR (500 MHz, $CDCl_3$) δ 4.02 – 3.95 (m, 1H), 3.39 (d, *J* = 11.7 Hz, 1H), 3.25 (d, *J* = 11.8 Hz, 1H), 2.40 – 2.31 (m, 1H), 2.24 (dt, *J* = 16.4, 4.6 Hz, 1H), 1.89 (dd, *J* = 12.1, 5.7 Hz, 1H), 1.84 (dd, *J* = 3.1, 1.4 Hz, 3H), 1.53 (dd, *J* = 12.1, 10.6 Hz, 1H), 1.17 (s, 3H), 1.09 (s, 3H). ^{19}F NMR (471 MHz, $CDCl_3$) δ -138.41. ^{13}C NMR (126 MHz, $CDCl_3$) δ 158.2 (d, *J* = 31.7 Hz), 147.1 (d, *J* = 248.4 Hz), 122.9 (d, *J* = 12.3 Hz), 57.3 (d, *J* = 1.7 Hz), 55.0, 47.0, 37.4, 34.7 (d, *J* = 3.8 Hz), 27.6, 27.5, 14.9 (d, *J* = 4.0 Hz). HRMS (EI) calcd for $C_{11}H_{17}FNO$ $[M+H]^+$: 198.1289 found: 198.1289.

(*R*)-6-fluoro-2,2-dimethyl-7-propyl-2,3,8,8a-tetrahydroindolizin-5(1H)-one (5b).

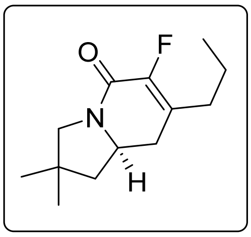

5b

By means of the general procedure described above, indolizidine **5b** (9 mg, 25% yield) was obtained from 40 mg (0.16 mmol) of **3b** as a white solid in 96% ee after flash chromatography with 3:1 n-hexane: ethyl acetate. M. p. = 42-43.5 °C. The ee value was determined by HPLC analysis using a Chiralpak IC column (hexane: isopropanol 85:15); flow rate = 1.0 mL/min, t_{major}= 51.3 min, t_{minor}= 46.3 min. $[\alpha]_D^{25}$ = -31.1 (*c* 1.0, $CHCl_3$). ^{1}H NMR (300 MHz, $CDCl_3$) δ 4.01 – 3.90 (m, 1H), 3.40 (d, *J* = 11.9 Hz, 1H), 3.25 (d, *J* = 11.9 Hz, 1H), 2.32 – 2.20 (m, 4H), 1.89 (dd, *J* = 11.9, 5.8 Hz, 1H), 1.54 – 1.45 (m, 3H), 1.18 (s, 3H), 1.09 (s, 3H), 0.94 (t, *J* = 7.4 Hz, 3H). ^{19}F NMR (282 MHz,

$CDCl_3$) δ -138.95. ^{13}C NMR (126 MHz, $CDCl_3$) δ 158.4 (d, *J* = 32.2 Hz), 147.0 (d, *J* = 248.5 Hz), 126.7 (d, *J* = 11.9 Hz), 57.3, 55.2, 47.1, 37.4, 32.6 (d, *J* = 3.9 Hz), 30.6 (d, *J* = 2.6 Hz), 27.6, 27.5, 20.3 (d, *J* = 2.2 Hz), 13.9. HRMS (ESI/Q-TOF): m/z $[M + H]^+$ calcd for $C_{13}H_{21}FNO$ $[M+H]^+$: 226.1602 found: 226.1608.

(*R*)-6-fluoro-2,2-dimethyl-7-pentyl-2,3,8,8a-tetrahydroindolizin-5(1H)-one (5c).

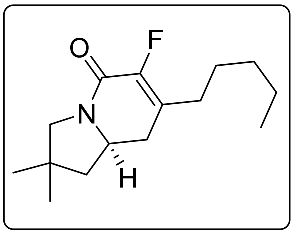

5c

By means of the general procedure described above, indolizidine **5c** (11 mg, 24% yield) was obtained from 50 mg (0.17 mmol) of **3c** as a semisolid in 96% ee after flash chromatography with 4:1 n-hexane: ethyl acetate. The ee value was determined by HPLC analysis using a Chiralpak IC column (hexane: isopropanol 85:15); flow rate = 1.0 mL/min, t_{major}= 47.7 min, t_{minor}= 40.9 min. $[\alpha]_D^{25}$ = -6.7 (*c* 1.0, $CHCl_3$). ^1H NMR (500 MHz, $CDCl_3$) δ 3.98 – 3.91 (m, 1H), 3.39 (d, *J* = 11.8 Hz, 1H), 3.25 (d, *J* = 11.8 Hz, 1H), 2.30 – 2.27 (m, 2H), 2.25 – 2.21 (m, 2H), 1.89 (dd, *J* = 12.2, 5.7 Hz, 1H), 1.54 (dd, *J* = 12.1, 10.6 Hz, 1H), 1.45 – 1.42 (m, 2H), 1.35 – 1.27 (m, 4H), 1.18 (s, 3H), 1.09 (s, 3H), 0.89 (t, *J* = 7.0 Hz, 3H). ^{19}F NMR (471 MHz, $CDCl_3$) δ -138.55. ^{13}C NMR (126 MHz, $CDCl_3$) δ 158.4 (d, *J* = 32.1 Hz), 146.8 (d, *J* = 248.5 Hz), 127.0 (d, *J* = 11.9 Hz), 57.3, 55.2, 47.1, 37.4, 32.6 (d, *J* = 3.8 Hz), 31.5, 28.6 (d, *J* = 2.7 Hz), 27.6, 27.5, 26.7 (d, *J* = 1.9 Hz), 22.5, 14.1. HRMS (ESI/Q-TOF): m/z $[M + H]^+$ calcd for $C_{15}H_{25}FNO$ $[M+H]^+$: 254.1915 found: 254.1918.

(*R*)-6'-fluoro-7'-methyl-8',8a'-dihydro-1'H-spiro[cyclopropane-1,2'-indolizin]-5'(3'H)-one (5e).

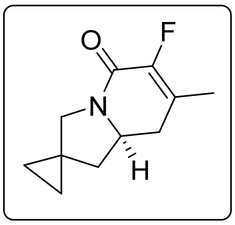

5e

By means of the general procedure described above, indolizidine **5e** (25 mg, 41% yield) was obtained from 70 mg (0.31 mmol) of **3e** as a semi solid in 91% ee after flash chromatography with 1:1 n-hexane: ethyl acetate. The ee value was determined by HPLC analysis using a Phenomenex Celulose 4 column (hexane: isopropanol 80:20); flow rate = 1.0 mL/min, t_{major}= 21.3 min, t_{minor}= 28.6 min. $[\alpha]_D^{25}$ = -30.0 (*c* 1.0, $CHCl_3$). ^1H NMR (500 MHz, $CDCl_3$) δ 4.09 – 4.02 (m, 1H),

3.65 (d, *J* = 11.7 Hz, 1H), 3.24 (d, *J* = 11.7 Hz, 1H), 2.46 – 2.38 (m, 1H), 2.29 (dt, *J* = 16.4, 4.7 Hz, 1H), 2.00 (dd, *J* = 11.9, 10.5 Hz, 1H), 1.86 (dd, *J* = 3.1, 1.5 Hz, 3H), 1.63 (dd, *J* = 12.1, 5.6 Hz, 1H), 0.73 – 0.65 (m, 2H), 0.58 – 0.52 (m, 2H). ^{19}F NMR (471 MHz, $CDCl_3$) δ -138.44. ^{13}C NMR (126 MHz, $CDCl_3$) δ 158.2 (d, *J* = 31.7 Hz), 146.9 (d, *J* = 247.9 Hz), 122.8 (d, *J* = 12.4 Hz), 56.2, 52.2 (d, *J* = 1.7 Hz), 42.0, 34.4 (d, *J* = 3.8 Hz), 20.1, 15.1, 14.9 (d, *J* = 3.8 Hz), 7.3. HRMS (ESI/Q-TOF): m/z $[M + H]^+$ calcd for $C_{11}H_{15}FNO$ $[M+H]^+$: 196.1132 found: 196.1133.

(*R*)-6'-fluoro-7'-methyl-8',8a'-dihydro-1'H-spiro[cyclobutane-1,2'-indolizin]-5'(3'H)-one (5f).

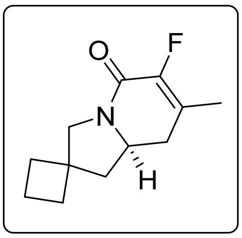

5f

By means of the general procedure described above, indolizidine **5f** (31 mg, 51% yield) was obtained from 70 mg (0.29 mmol) of **3f** as a white solid in 90% ee after flash chromatography with 2:1 n-hexane: ethyl acetate. M. p. = 67-68.5 °C. The ee value was determined by HPLC analysis using a Phenomenex Celulose 4 column (hexane: isopropanol 85:15); flow rate = 1.0 mL/min, t_{major}= 27.7 min, t_{minor}= 38.6 min. $[\alpha]_D^{25}$ = -80.0 (*c* 1.0, $CHCl_3$). ^{1}H NMR (500 MHz, $CDCl_3$) δ 3.83 – 3.67 (m, 1H), 3.68 (d, *J* = 12.0 Hz, 1H), 3.37 (d, *J* = 12.0 Hz, 1H), 2.38 – 2.32 (m, 1H), 2.27 – 2.20 (m, 2H), 2.12 – 2.09 (m, 1H), 2.02 – 1.96 (m, 2H), 1.93 – 1.87 (m, 2H), 1.83 (dd, *J* = 3.1, 1.5 Hz, 3H), 1.65 – 1.61 (m, 2H). ^{19}F NMR (471 MHz, $CDCl_3$) δ -138.44. ^{13}C NMR (126 MHz, $CDCl_3$) δ 158.2 (d, *J* = 31.8 Hz), 147.0 (d, *J* = 248.2 Hz), 122.9 (d, *J* = 12.4 Hz), 56.1 (d, *J* = 1.6 Hz), 54.6, 45.1, 43.9, 34.7, 34.3 (d, *J* = 3.7 Hz), 29.9, 16.3, 14.9 (d, *J* = 4.0 Hz).HRMS (ESI/Q-TOF): m/z $[M + H]^+$ calcd for $C_{12}H_{17}FNO$ $[M+H]^+$: 210.1289 found: 210.1291.

(*R*)-6'-fluoro-7'-methyl-8',8a'-dihydro-1'H-spiro[cyclopentane-1,2'-indolizin]-5'(3'H)-one (5g).

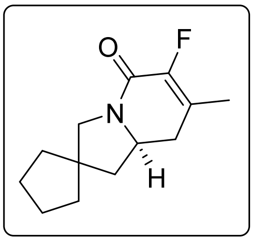

5g

By means of the general procedure described above, indolizidine **5g** (25 mg, 48% yield) was obtained from 60 mg (0.24 mmol) of **3g** as a white solid in 93% ee after flash chromatography

with 3:1 n-hexane: ethyl acetate. M. p. = 76-78 °C. The ee value was determined by HPLC analysis using a Phenomenex Celulose 4 column (hexane: isopropanol 85:15); flow rate = 1.0 mL/min, t_{major}= 25.8 min, t_{minor}= 32.6 min. $[\alpha]_D^{25}$ = -97.7 (*c* 1.0, $CHCl_3$). ^1H NMR (300 MHz, $CDCl_3$) δ 3.96 – 3.84 (m, 1H), 3.48 (d, *J* = 11.8 Hz, 1H), 3.31 (d, *J* = 11.8 Hz, 1H), 2.43 – 2.30 (m, 1H), 2.24 (dt, *J* = 16.3, 4.9 Hz, 1H), 1.96 (dd, *J* = 12.0, 5.6 Hz, 1H), 1.84 (dd, *J* = 3.1, 1.4 Hz, 3H), 1.68 – 1.59 (m, 8H), 1.50 – 1.43 (m, 1H). ^{19}F NMR (282 MHz, $CDCl_3$) δ -138.95. ^{13}C NMR (75 MHz, $CDCl_3$) δ 158.2 (d, *J* = 31.8 Hz), 147.0 (d, *J* = 248.2 Hz), 122.9 (d, *J* = 12.2 Hz), 56.0 (d, *J* = 1.5 Hz), 55.4, 48.5, 45.0, 38.4, 37.3, 34.5 (d, *J* = 3.8 Hz), 24.6, 24.5, 14.9 (d, *J* = 3.9 Hz). HRMS (ESI/Q-TOF): m/z $[M + H]^+$ calcd for $C_{13}H_{19}FNO$ $[M+H]^+$: 224.1445 found: 224.1449.

(*R*)-6'-fluoro-7'-methyl-8',8a'-dihydro-1'H-spiro[cyclohexane-1,2'-indolizin]-5'(3'H)-one (5h).

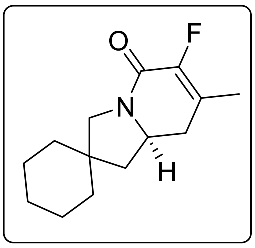

5h

By means of the general procedure described above, indolizidine **5h** (31 mg, 35% yield) was obtained from 100 mg (0.37 mmol) of **3h** as a white solid in 96% ee after flash chromatography with 2:1 n-hexane: ethyl acetate. M. p. = 69-71 °C. The ee value was determined by HPLC analysis using a Chiralpak IC column (hexane: isopropanol 70:30); flow rate = 1.0 mL/min, t_{major}= 30.3 min, t_{minor}= 28.5 min. $[\alpha]_D^{25}$ = -90.1 (*c* 1.0, $CHCl_3$). ^1H NMR (300 MHz, $CDCl_3$) δ 3.99 – 3.87 (m, 1H), 3.53 (d, *J* = 12.1 Hz, 1H), 3.23 (d, *J* = 12.1 Hz, 1H), 2.40 – 2.19 (m, 2H), 2.02 (dd, *J* = 12.3, 5.8 Hz, 1H), 1.84 (dd, *J* = 3.1, 1.3 Hz, 3H), 1.60 – 1.50 (m, 3H), 1.44 – 1.25 (m, 6H), 0.88 – 0.81 (m, 2H). ^{19}F NMR (282 MHz, $CDCl_3$) δ -139.00. ^{13}C NMR (126 MHz, $CDCl_3$) δ 158.3 (d, *J* = 31.7 Hz), 147.0 (d, *J* = 248.2 Hz), 122.8 (d, *J* = 12.2 Hz), 54.8, 54.2, 45.3, 41.4, 37.9, 35.7, 34.7 (d, *J* = 3.7 Hz), 26.0, 24.0, 22.9, 14.9 (d, *J* = 3.8 Hz).HRMS (ESI/Q-TOF): m/z $[M + H]^+$ calcd for $C_{14}H_{21}FNO$ $[M+H]^+$: 238.1602 found: 238.1607.

(*R*)-6-fluoro-7-methyl-2',3',5',6',8,8a-hexahydro-1H-spiro[indolizine-2,4'-pyran]-5(3H)-one (5i).

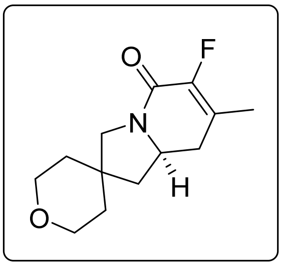

5i

By means of the general procedure described above, indolizidine **5i** (28 mg, 45% yield) was obtained from 70 mg (0.26 mmol) of **3i** as a white solid in 98% ee after flash chromatography with 1:3 n-hexane: ethyl acetate. M. p. = 108-110 °C. The ee value was determined by HPLC analysis using a Chiralpak IC column (hexane: isopropanol 70:30); flow rate = 1.0 mL/min, t_{major}= 34.2 min, t_{minor}= 33.0 min. $[\alpha]_D^{25}$ = -25.0 (*c* 1.0, $CHCl_3$). ^1H NMR (300 MHz, $CDCl_3$) δ 4.01 – 3.89 (m, 1H), 3.81 – 3.70 (m, 2H), 3.65 – 3.49 (m, 3H), 3.31 (d, *J* = 12.1 Hz, 1H), 2.38 – 2.22 (m, 2H), 2.11 (dd, *J* = 12.4, 5.8 Hz, 1H), 1.84 (dd, *J* = 3.1, 1.3 Hz, 3H), 1.69 – 1.59 (m, 4H), 1.55 – 1.48 (m, 1H). ^{19}F NMR (282 MHz, $CDCl_3$) δ -138.87. ^{13}C NMR (75 MHz, $CDCl_3$) δ 158.3 (d, *J* = 31.8 Hz), 146.9 (d, *J* = 248.4 Hz), 123.1 (d, *J* = 12.4 Hz), 65.6, 64.7, 54.1, 53.9, 45.1, 39.1, 37.3, 35.9, 34.6 (d, *J* = 3.7 Hz), 14.9 (d, *J* = 3.8 Hz). HRMS (ESI/Q-TOF): m/z $[M + H]^+$ calcd for $C_{13}H_{19}FNO_2$ $[M+H]^+$: 240.1394 found: 240.1396. (In this case the olefin was filtered washing with acetone)

(*R*)-tert-butyl 6-fluoro-7-methyl-5-oxo-3,5,8,8a-tetrahydro-1H-spiro[indolizine-2,4'-piperidine]-1'-carboxylate (5j)

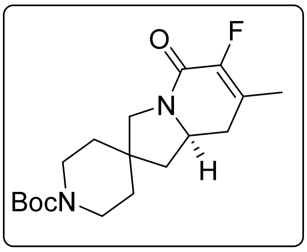

5j

By means of the general procedure described above, indolizidine **5j** (18 mg, 33% yield) was obtained from 60 mg (0.16 mmol) of **3j** as a white solid in 98% ee after flash chromatography with 1:1 n-hexane: ethyl acetate. M. p. = 124-125.5 °C. The ee value was determined by HPLC analysis using a Chiralpak IC column (hexane: isopropanol 30:70); flow rate = 1.0 mL/min, t_{major}= 53.5 min, t_{minor}= 37.4 min. $[\alpha]_D^{25}$ = -34.7 (*c* 1.0, $CHCl_3$). ^1H NMR (500 MHz, $CDCl_3$) δ 3.99 – 3.92 (m, 1H), 3.60 – 3.58 (m, 3H), 3.30 (d, *J* = 12.2 Hz, 1H), 3.25 (ddd, *J* = 13.9, 8.9, 3.8 Hz, 1H), 3.18 – 3.13 (m, 1H), 2.40 – 2.33 (m, 1H), 2.28 (dt, *J* = 16.5, 4.8 Hz, 1H), 2.07 (dd, *J* = 12.5, 5.8 Hz, 1H), 1.85

(dd, J = 3.1, 1.4 Hz, 3H), 1.55 – 1.52 (m, 5H), 1.46 (s, 9H). ^{19}F NMR (471 MHz, $CDCl_3$) δ -138.34. ^{13}C NMR (126 MHz, $CDCl_3$) δ 158.3 (d, J = 31.7 Hz), 154.8, 146.9 (d, J = 248.5 Hz), 123.1 (d, J = 12.2 Hz), 79.9, 54.0, 53.80, 53.79, 44.5, 40.0, 36.7, 35.00, 34.95, 34.6 (d, J = 3.6 Hz), 28.6, 14.9 (d, J = 3.8 Hz). HRMS (ESI/Q-TOF): m/z [M + H]$^+$ calcd for $C_{18}H_{28}FN_2O_3$ [M+H]$^+$: 339.2078 found: 339.2080. (In this case the olefin was filtered washing with acetone)

(*R*)-6-fluoro-7-methyl-2,3,8,8a-tetrahydroindolizin-5(1H)-one (5k).

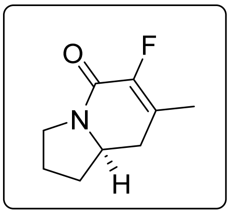

5k

By means of the general procedure described above, indolizidine **5k** (10 mg, 30% yield) was obtained from 39 mg (0.20 mmol) of **3k** as a semi solid in 79% ee after flash chromatography with 4:1 n-hexane: ethyl acetate. The ee value was determined by HPLC analysis using a Phenomenex Celulose 4 column (hexane: isopropanol 60:40); flow rate = 1.0 mL/min, t_{major}= 11.0 min, t_{minor}= 12.3 min. $[\alpha]_D^{25}$ = -9.6 (c 1.0, $CHCl_3$). ^{1}H NMR (300 MHz, $CDCl_3$) δ 3.83 – 3.71 (m, 1H), 3.67 – 3.60 (m, 1H), 3.48 (dd, J = 18.7, 10.6 Hz, 1H), 2.42 – 2.28 (m, 2H), 2.22 – 2.16 (m, 1H), 2.10 – 2.01 (m, 1H), 1.85 (dd, J = 3.0, 1.2 Hz, 3H), 1.68 – 1.61 (m, 2H). ^{19}F NMR (282 MHz, $CDCl_3$) δ -139.11. ^{13}C NMR (126 MHz, $CDCl_3$) δ 158.4 (d, J = 32.0 Hz), 147.1 (d, J = 248.0 Hz), 122.6 (d, J = 12.5 Hz), 56.0, 44.4 (d, J = 1.7 Hz), 34.4 (d, J = 3.8 Hz), 33.2, 23.6, 14.9 (d, J = 3.8 Hz). HRMS (ESI/Q-TOF): m/z [M + H]$^+$ calcd for $C_9H_{13}FNO$ [M+H]$^+$: 170.0976 found: 170.0973.

(*R*)-6-fluoro-7-methyl-2,2-diphenyl-2,3,8,8a-tetrahydroindolizin-5(1H)-one (5l).

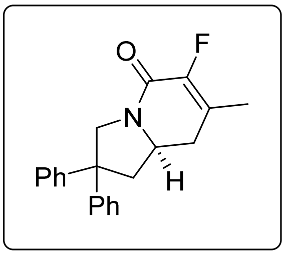

5l

By means of the general procedure described above, indolizidine **5l** (18 mg, 33% yield) was obtained from 60 mg (0.17 mmol) of **3l** as a white solid in 71% ee after flash chromatography with 2:1 n-hexane: ethyl acetate. M. p. = 160-161 °C. The ee value was determined by HPLC analysis using a Phenomenex Amilose 1 column (hexane: isopropanol 70:30; flow rate = 1.0 mL/min, t_{major}= 7.3 min, t_{minor}= 8.2 min. $[\alpha]_D^{25}$ = -36.5 (c 1.0, $CHCl_3$). ^{1}H NMR (500 MHz, $CDCl_3$) δ

7.33 – 7.27 (m, 4H), 7.26 – 7.17 (m, 6H), 4.76 (d, *J* = 12.5 Hz, 1H), 3.82 – 3.74 (m, 2H), 2.67 (dd, *J* = 11.8, 4.9 Hz, 1H), 2.48 – 2.39 (m, 2H), 2.23 (dt, *J* = 16.2, 4.5 Hz, 1H), 1.83 (dd, *J* = 3.2, 1.4 Hz, 3H). ^{19}F NMR (471 MHz, $CDCl_3$) δ -138.19. ^{13}C NMR (126 MHz, $CDCl_3$) δ 157.8 (d, *J* = 31.8 Hz), 147.0 (d, *J* = 248.6 Hz), 145.6, 145.3, 128.8, 128.7, 127.0, 126.8, 126.7, 123.4 (d, *J* = 12.3 Hz), 54.2 (d, *J* = 1.1 Hz), 53.8, 52.8, 44.7, 34.0 (d, *J* = 3.6 Hz), 15.0 (d, *J* = 3.7 Hz). HRMS (ESI/Q-TOF): m/z $[M + H]^+$ calcd for $C_{21}H_{21}FNO$ $[M+H]^+$: 322.1602 found: 322.1608.

(*R*)-7-fluoro-8-methyl-9a,10-dihydropyrido[1,2-a]indol-6(9H)-one (5m).

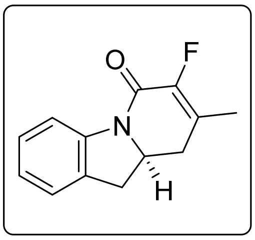

5m

By means of the general procedure described above, indolizidine **5m** (8 mg, 32% yield) was obtained from 28 mg (0.11 mmol) of **3m** as a yellow solid in 91% ee after flash chromatography with 4:1 n-hexane: ethyl acetate. M. p. = 104-105°C. The ee value was determined by HPLC analysis using a Chiralpak IC column (hexane: isopropanol 75:25); flow rate = 1.0 mL/min, t_{major}= 24.0 min, t_{minor}= 26.1 min. $[\alpha]_D^{25}$ = -72 (*c* 1.0, $CHCl_3$). ^{1}H NMR (500 MHz, $CDCl_3$) δ 8.20 – 8.18 (m, 1H), 7.25 – 7.22 (m, 2H), 7.07 – 7.04 (m, 1H), 4.31 (dddd, *J* = 13.6, 10.8, 8.4, 5.5 Hz, 1H), 3.30 (dd, *J* = 15.5, 8.3 Hz, 1H), 2.89 (dd, *J* = 15.5, 10.7 Hz, 1H), 2.66 – 2.58 (m, 1H), 2.56 – 2.52 (m, 1H), 1.96 (dd, *J* = 3.1, 1.5 Hz, 3H). ^{19}F NMR (282 MHz, $CDCl_3$) δ -138.36. ^{13}C NMR (126 MHz, $CDCl_3$) δ 156.8 (d, *J* = 32.9 Hz), 147.0 (d, *J* = 246.9 Hz), 142.1 (d, *J* = 2.7 Hz), 130.1, 128.1, 124.7, 124.09 (d, *J* = 12.4 Hz), 124.08, 116.1, 57.0, 35.7, 34.9 (d, *J* = 3.7 Hz), 15.2 (d, *J* = 3.8 Hz). HRMS (ESI/Q-TOF): m/z $[M + H]^+$ calcd for $C_{13}H_{13}FNO$ $[M+H]^+$: 218.0976 found: 218.0977.

(*R*)-7-fluoro-2,8-dimethyl-9a,10-dihydropyrido[1,2-a]indol-6(9H)-one (5n).

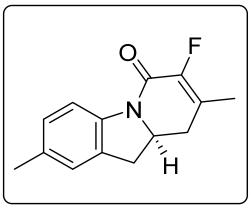

5n

By means of the general procedure described above, indolizidine **5n** (13 mg, 42% yield) was obtained from 35 mg (0.13 mmol) of **3n** as a yellow solid in 89% ee after flash chromatography with 4:1 n-hexane: ethyl acetate. M. p. = 156-157 °C. The ee value was determined by HPLC

analysis using a Phenomenex Amilose 1 column (hexane: isopropanol 80:20); flow rate = 1.0 mL/min, t_{major}= 13.6 min, t_{minor}= 12.1 min. $[\alpha]_D^{25}$ = -57 (*c* 1.0, $CHCl_3$). ^1H NMR (500 MHz, $CDCl_3$) δ 8.06 (d, *J* = 8.0 Hz, 1H), 7.06 – 7.03 (m, 2H), 4.28 (dddd, *J* = 13.6, 10.7, 8.3, 5.4 Hz, 1H), 3.25 (dd, *J* = 15.5, 8.3 Hz, 1H), 2.85 (dd, *J* = 15.5, 10.7 Hz, 1H), 2.64 – 2.57 (m, 1H), 2.55 – 2.50 (m, 1H), 2.33 (s, 3H), 1.95 (dd, *J* = 3.1, 1.4 Hz, 3H). ^{19}F NMR (471 MHz, $CDCl_3$) δ -137.94. ^{13}C NMR (126 MHz, $CDCl_3$) δ 156.5 (d, *J* = 32.5 Hz), 147.0 (d, *J* = 246.9 Hz), 139.8 (d, *J* = 2.4 Hz), 133.8, 130.3, 128.5, 125.4, 123.7 (d, *J* = 12.8 Hz), 115.8, 57.1, 35.7, 34.9 (d, *J* = 3.7 Hz), 21.3, 15.2 (d, *J* = 3.7 Hz). HRMS (ESI/Q-TOF): m/z $[M + H]^+$ calcd for $C_{14}H_{15}FNO$ $[M+H]^+$: 232.1132 found: 232.1135.

(*R*)-7-fluoro-2-methoxy-8-methyl-9a,10-dihydropyrido[1,2-a]indol-6(9H)-one (5o).

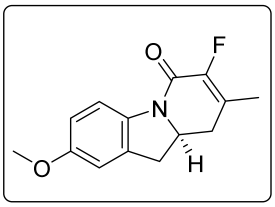

5o

By means of the general procedure described above, indolizidine **5o** (10 mg, 31% yield) was obtained from 36 mg (0.13 mmol) of **3o** as a yellow solid in 90% ee after flash chromatography with 2:1 n-hexane: ethyl acetate. M. p. = 124-125 °C. The ee value was determined by HPLC analysis using a Phenomenex Amilose 1 column (hexane: isopropanol 80:20); flow rate = 1.0 mL/min, t_{major}= 18.1 min, t_{minor}= 16.7 min. $[\alpha]_D^{25}$ = -63 (*c* 1.0, $CHCl_3$). ^1H NMR (500 MHz, $CDCl_3$) δ 8.10 (d, *J* = 8.5 Hz, 1H), 6.80 – 6.77 (m, 2H), 4.29 (dddd, *J* = 13.6, 10.7, 8.3, 5.3 Hz, 1H), 3.79 (s, 3H), 3.25 (dd, *J* = 15.6, 8.2 Hz, 1H), 2.86 (dd, *J* = 15.7, 10.7 Hz, 1H), 2.64 – 2.58 (m, 1H), 2.55 – 2.50 (m, 1H), 1.95 (dd, *J* = 3.1, 1.1 Hz, 3H). ^{19}F NMR (471 MHz, $CDCl_3$) δ -137.97. ^{13}C NMR (126 MHz, $CDCl_3$) δ 156.7, 156.2 (d, *J* = 32.9 Hz), 147.0 (d, *J* = 246.8 Hz), 135.9 (d, *J* = 2.6 Hz), 131.8, 123.3 (d, *J* = 12.8 Hz), 116.7, 112.5, 111.2, 57.2, 55.8, 35.8, 34.8 (d, *J* = 3.8 Hz), 15.1 (d, *J* = 3.8 Hz). HRMS (ESI/Q-TOF): m/z $[M + H]^+$ calcd for $C_{14}H_{15}FNO_2$ $[M+H]^+$: 248.1081 found: 248.1087.

(*S*)-2,2,7-trimethyl-6-(trifluoromethyl)-2,3,8,8a-tetrahydroindolizin-5(1H)-one (5p).

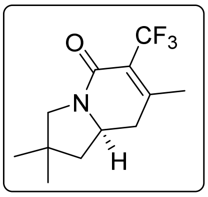

5p

By means of the general procedure described above, indolizidine **5p** (14 mg, 40% yield) was obtained from 39 mg (0.14 mmol) of **3p** as a colorless oil in 85% ee after flash chromatography with 1:1 n-hexane: ethyl acetate. M. p. = 72-73.5 °C. The ee value was determined by HPLC analysis using a Phenomenex Celulose 4 column (hexane: isopropanol 80:20); flow rate = 1.0 mL/min, t_{major}= 10.5 min, t_{minor}= 14.7 min. $[\alpha]_D^{25}$ = -128 (*c* 1.0, $CHCl_3$). ^{1}H NMR (500 MHz, $CDCl_3$) δ 3.92 – 3.85 (m, 1H), 3.39 (d, *J* = 11.8 Hz, 1H), 3.25 (d, *J* = 11.8 Hz, 1H), 2.42 (dd, *J* = 17.3, 4.8 Hz, 1H), 2.38 – 2.30 (m, 1H), 2.11 (qd, *J* = 2.7, 0.9 Hz, 3H), 1.93 (dd, *J* = 12.1, 5.9 Hz, 1H), 1.49 (dd, *J* = 12.1, 10.2 Hz, 1H), 1.18 (s, 3H), 1.09 (s, 3H). ^{19}F NMR (471 MHz, $CDCl_3$) δ -57.37. ^{13}C NMR (126 MHz, $CDCl_3$) δ 159.4, 154.2 (q, *J* = 2.7 Hz), 123.1 (q, *J* = 275.4 Hz), 122.8 (q, *J* = 28.7 Hz), 57.3, 53.8, 47.3, 39.8, 37.0, 27.24, 27.17, 21.0 (q, *J* = 2.8 Hz). HRMS (ESI/Q-TOF): m/z $[M + H]^+$ calcd for $C_{12}H_{17}F_3NO$ $[M+H]^+$: 248.1257 found: 248.1258.

SCALED PROCESS EXAMPLE:

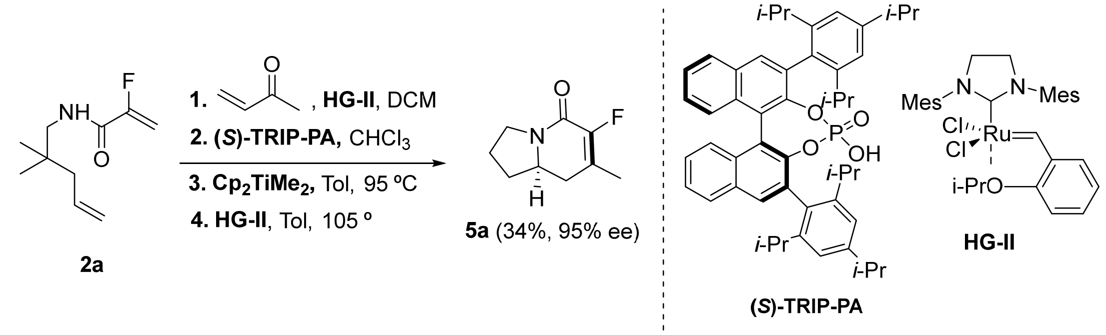

To a solution of fluoroacrylamide **1a** (1,48 g, 8 mmol) in dichloromethane (26.6 mL, 0.3 M), methyl vinyl ketone **6a** (1.98 mL, 3.0 equiv) and second generation Hoveyda-Grubbs catalyst (500 mg, 10 mol %) were successively added. The resulting mixture was stirred for 12 h at room temperature (monitored by TLC) and then concentrated to dryness and purified by flash chromatography on silica gel with 4:1 n-hexane:ethyl acetate as eluent to afford **2a** (1.6 g, 88% yield) as a brown oil. After that, the enone **2a** (1.6 g, 7 mmol) was dissolved in chloroform (70 mL, 0.1 M). Then (*S*)-TRIP BPA (526 mg, 10 mol%) was added and the resulting solution was stirred at room temperature for 36 hours. The solvent was removed under reduced pressure and the residue was chromatographed on silica gel with 4:1 n-hexane:ethyl acetate as eluent to afford **3a** (1.34 g, 84% yield) as a white solid. Afterwards, pyrrolidine **3a** (1.34 g, 5.9 mmol) was dissolved in toluene (59 mL, 0.1 M) under N_2 atmosphere. Then, a freshly prepared 0.5 M solution of dimethyl titanocene in toluene was added (29.5 mL, 2.5 equiv.), and the reaction mixture was heated at 95 °C in a metal block for 4h. The resulting suspension was filtered through a short pad of silica gel washing with small portions of dichloromethane. The filtrate was concentrated carefully at 350 mb, obtaining a yellow oil that was employed without further purification. The crude olefine was dissolved in toluene (1.18 L, 0.005 M), and an initial loading of second generation Hoveyda-Grubbs catalyst (185 mg, 5 mol %) was added. The resulting mixture was heated at 105 °C in a metal block for 24h. Then it was cooled to room temperature, and one more portion of catalyst (185 mg, 5 mol%) was added. The reaction mixture was reheated at 105 °C for another 24 h and then concentrated to dryness and purified by flash chromatography on silica gel with 1:1 n-hexane:ethyl acetate as eluent to afford **5a** (540 mg, 46% yield) as a white solid.

SYNTHESIS OF (6S*,7S*,8aS*)-6-FLUORO-2,2,7-TRIMETHYLHEXAHYDROINDOLIZIN-5(1H)-ONE ((±)-7):

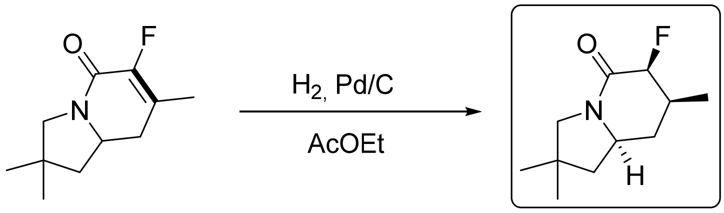

To a solution of indolizidine **5a** (30 mg, 0.15 mmol) in ethyl acetate (1.5 mL, 0.1M), Pd/C 10% w/t (10 mol%, 16 mg) was added. A balloon filled with hydrogen was then connected and the resulting black suspension was stirred 24 hours at room temperature. The suspension was filtered through a short pad of Celite washing with small portions of ethyl acetate. The filtrate was concentrated and purified by flash chromatography on silica gel with 1:1 n-hexane:ethyl acetate as eluent to afford **7** (20 mg, 66% yield) as a white solid. M. p. = 64-65 °C. ^{1}H NMR (300 MHz, $CDCl_3$) δ 4.64 (dd, *J* = 47.7, 3.8 Hz, 1H), 3.74 – 3.62 (m, 1H), 3.44 (d, *J* = 11.7 Hz, 1H), 3.19 (d, *J* = 12.4 Hz, 1H), 2.13 – 1.92 (m, 1H), 1.85 – 1.79 (m, 2H), 1.51 – 1.40 (m, 2H), 1.17 – 1.13 (m, 6H), 1.11 (s, 3H). ^{19}F NMR (282 MHz, $CDCl_3$) δ -196.17. ^{13}C NMR (75 MHz, $CDCl_3$) δ 164.5 (d, *J* = 20.4 Hz), 88.9 (d, *J* = 175.0 Hz), 58.3 (d, *J* = 3.3 Hz), 47.4, 35.9, 34.1, 33.8, 32.0, 28.2, 28.0, 15.7 (d, *J* = 7.8 Hz). HRMS (ESI/Q-TOF): m/z $[M + H]^+$ calcd for $C_{11}H_{19}FNO$ $[M+H]^+$: 200.1445 found: 200.1452.

X-RAY STRUCTURE OF COMPOUNDS 3p AND (±)-7:

Intensity data were collected on an Agilent Technologies Dual Super-Nova diffractometer, which was equipped with monochromated Cu kα radiation (λ=1.54184 Å) and Atlas CCD detector. Measurement was carried out at 170.00(10) K with the help of an Oxford Cryostream 700 PLUS temperature device. Data frames were processed (united cell determination, analytical absorption correction with face indexing, intensity data integration and correction for Lorentz and polarization effects) using the Crysalis software package.[6] The structure was solved using SHELXT[7] and refined by full-matrix least-squares with SHELXL-97.[8] Final geometrical calculations were carried out with Mercury[9]. and PLATON[10] as integrated in WinGX.[11]

The ellipsoid contour % probability levels in the caption for the images of the structures was 50% in both cases.

X-Ray structure of compound 3p[12].

Compound **3p** was crystallized using hexane as solvent, by heating until complete solution and cooling down slowly until rt.

Analysis of the absolute structure using likelihood methods (Hooft, Straver & Spek, 2008) was performed using PLATON (Spek, 2010). The results indicated that the absolute structure had been correctly assigned. The method calculated that the probability that the structure is inverted is smaller than 10^{-69}. The absolute structure parameter *y* (Hooft, Straver & Spek, 2008) was calculated using PLATON (Spek, 2010). The resulting value was *y*=0.02(6), which together with Flack parameter value, indicate that the absolute structure has probably been determined correctly.

Experimental

Single crystals of $C_{13}H_{18}F_3NO_2$ **[3p]**. A suitable crystal was selected and **mounted** on a **SuperNova, Dual, Cu at home/near, HyPix** diffractometer. The crystal was kept at 170.00(10) K during data

---

[6] CrysAlisPro, Agilent Technologies,Version 1.171.37.31 (release 14-01-2014 CrysAlis171 .NET)(compiled Jan 14 2014,18:38:05).

[7] Sheldrick, G.M. *Acta Cryst.* **2015**, *A71*, 3-8.

[8] Sheldrick, G. M. *Acta Cryst.* **2008**, *A64*,112.; Sheldrick, G. M. *Acta Cryst.* **2015**, *C71*, 3-8.

[9] Macrae, C. F., *J. Appl. Cryst.* **2008**, *41*, 466.

[10] Spek, A. L. (2010) PLATON, A Multipurpose Crystallographic Tool, Utrecht University, Utrecht, The Netherlands; Spek, A. L. *J. Appl. Cryst.* **2003**, *36*, 7.

[11] Farrugia, L. J., *J. Appl. Cryst.* **1999**, *32*, 837.

[12] CCDC 2247118 contains the supplementary crystallographic data of compound **3p**. These data can be obtained free of charge at www.ccdc.cam.ac.uk/conts/retrieving.html [or from the Cambridge Crystallographic Data Centre, 12 Union Road, Cambridge CB2 1EZ, UK; fax: (internat.) +44(1223)336-033, e-mail: deposit@ccdc.cam.ac.uk].

collection. Using Olex2,[13] the structure was solved with the ShelXT[14] structure solution program using Intrinsic Phasing and refined with the ShelXL[15] refinement package using Least Squares minimization.

Crystal structure determination

Crystal Data for $C_{13}H_{18}F_3NO_2$ (*M* =277.28 g/mol): orthorhombic, space group $P2_12_12_1$ (no. 19), a = 5.90610(10) Å, b = 13.6695(2) Å, c = 17.4738(3) Å, V = 1410.72(4) Å3, Z = 4, T = 170.00(10) K, μ(CuKα) = 0.980 mm^{-1}, *Dcalc* = 1.306 g/cm^3, 30574 reflections measured (8.212° ≤ 2Θ ≤ 137.948°), 2617 unique (R_{int} = 0.1018, R_{sigma} = 0.0329) which were used in all calculations. The final R^1 was 0.0370 (I > 2σ(I)) and wR_2 was 0.0976 (all data).

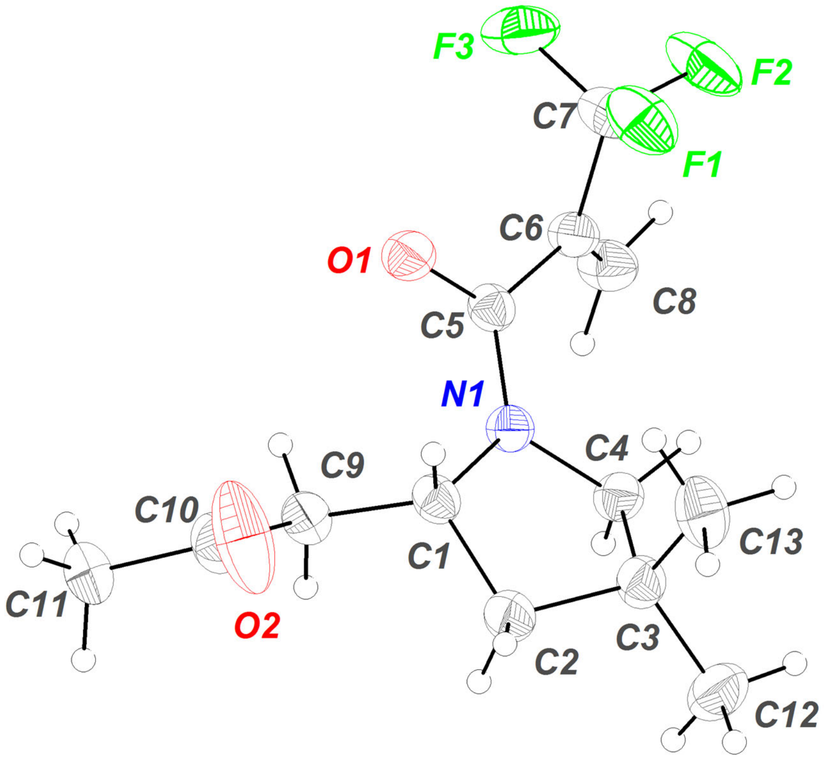

Figure S1. Ortep diagram for compound **3p**

X-Ray structure of compound (±)-7[16].

[13] Dolomanov, O. V., Bourhis, L. J., Gildea, R. J., Howard, J. A. K. & Puschmann, H. J. *Appl. Cryst.* **2009**, *42*, 339-341.
[14] Sheldrick, G. M. *Acta Cryst.* **2015**, *A71*, 3-8.
[15] Sheldrick, G.M. *Acta Cryst.* **2015**, *C71*, 3-8.
[16]. CCDC 2247169 contains the supplementary crystallographic data of compound **7**. These data can be obtained free of char0e at www.ccdc.cam.ac.uk/conts/retrieving.html [or from the Cambridge Crystallographic Data Centre, 12 Union Road, Cambridge CB2 1EZ, UK; fax: (internat.) +44(1223)336-033, e-mail: deposit@ccdc.cam.ac.uk].

Compound **7** was crystallized using hexane as solvent, by heating until complete solution and cooling down slowly until rt.

Experimental

Single crystals of $C_{11}H_{18}NOF$ **[(±)7]**. A suitable crystal was selected and **mounted** on a **SuperNova, Dual, Cu at home/near, HyPix** diffractometer. The crystal was kept at 170.00(10) K during data collection. Using Olex2[13], the structure was solved with the ShelXT[14] structure solution program using Intrinsic Phasing and refined with the ShelXL[15] refinement package using Least Squares minimization.

Crystal structure determination of [7]

Crystal Data for $C_{11}H_{18}NOF$ (M =199.26 g/mol): triclinic, space group P-1 (no. 2), a = 5.8802(2) Å, b = 10.2810(3) Å, c = 10.4680(3) Å, α = 63.042(3)°, β = 77.900(3)°, γ = 88.434(3)°, V = 549.88(3) Å^3, Z = 2, T = 170.00(10) K, μ(CuKα) = 0.714 mm^{-1}, $Dcalc$ = 1.203 g/cm^3, 9319 reflections measured (9.68° ≤ 2Θ ≤ 137.912°), 2024 unique (R_{int} = 0.0570, R_{sigma} = 0.0353) which were used in all calculations. The final R_1 was 0.0401 (I > 2σ(I)) and wR_2 was 0.1126 (all data).

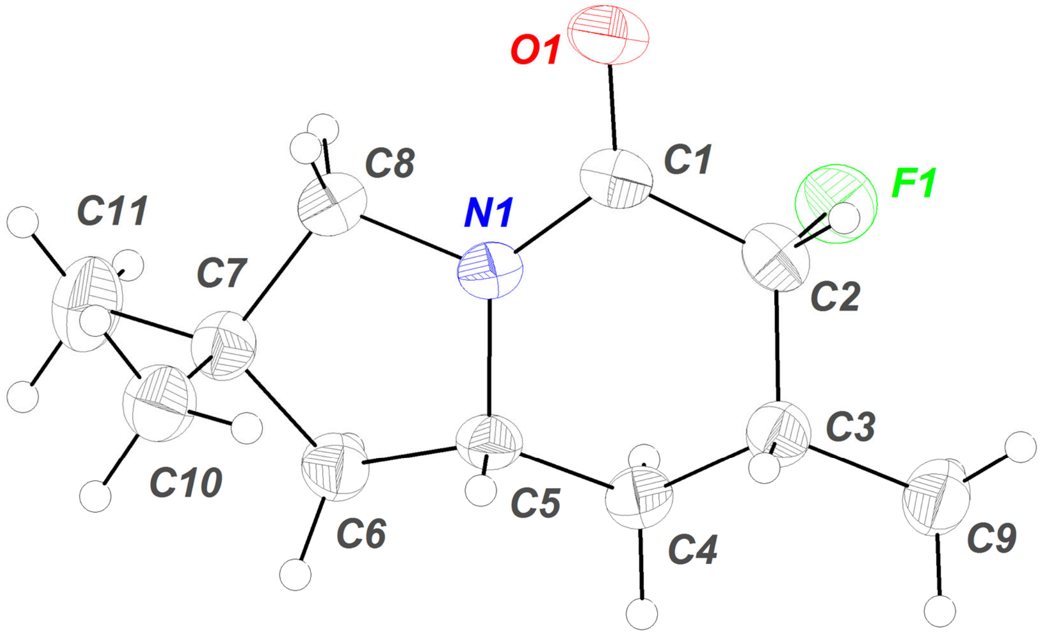

Figure S2. Ortep diagram for compound (±)-**7**

HPLC TRACES OF ENANTIOENRICHED COMPOUNDS 2 AND 3:

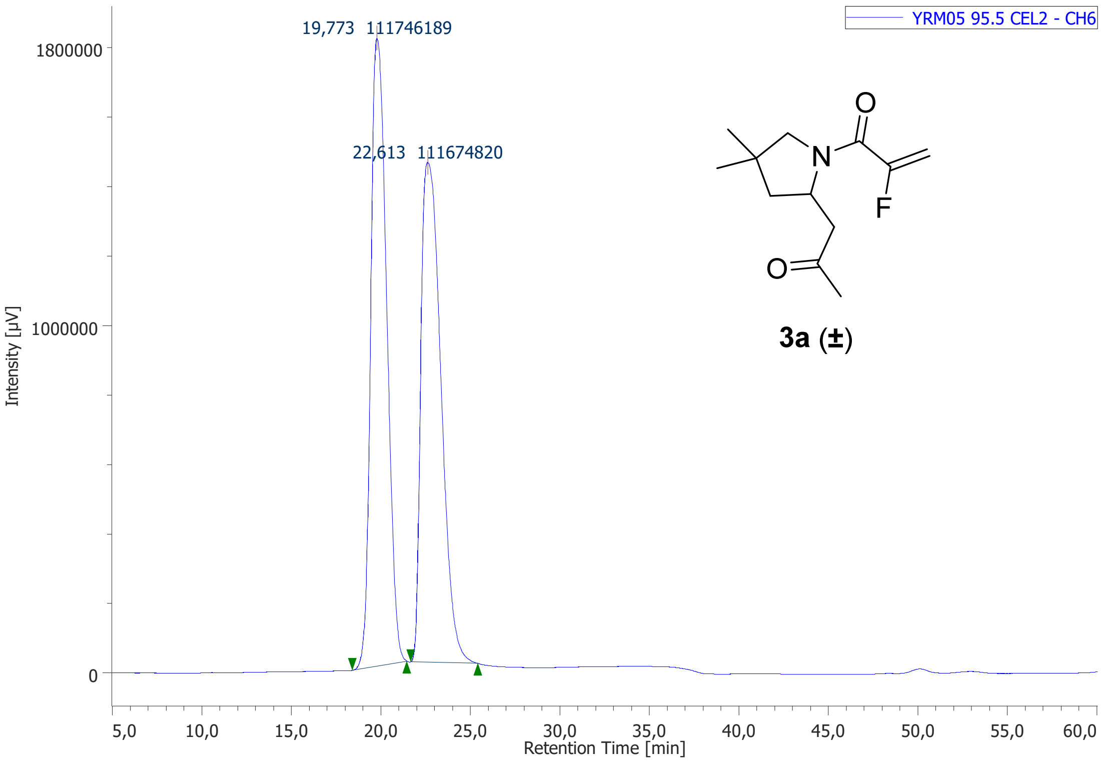

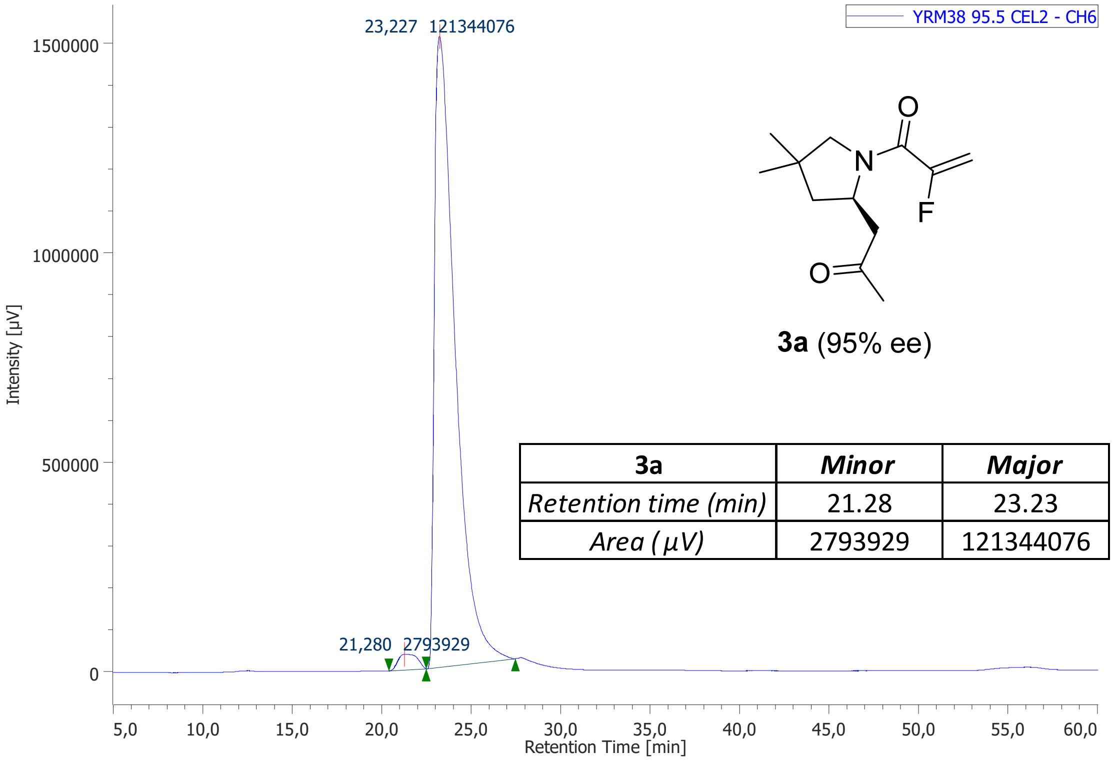

**3a**	***Minor***	***Major***
Retention time (min)	21.28	23.23
Area (µV)	2793929	121344076

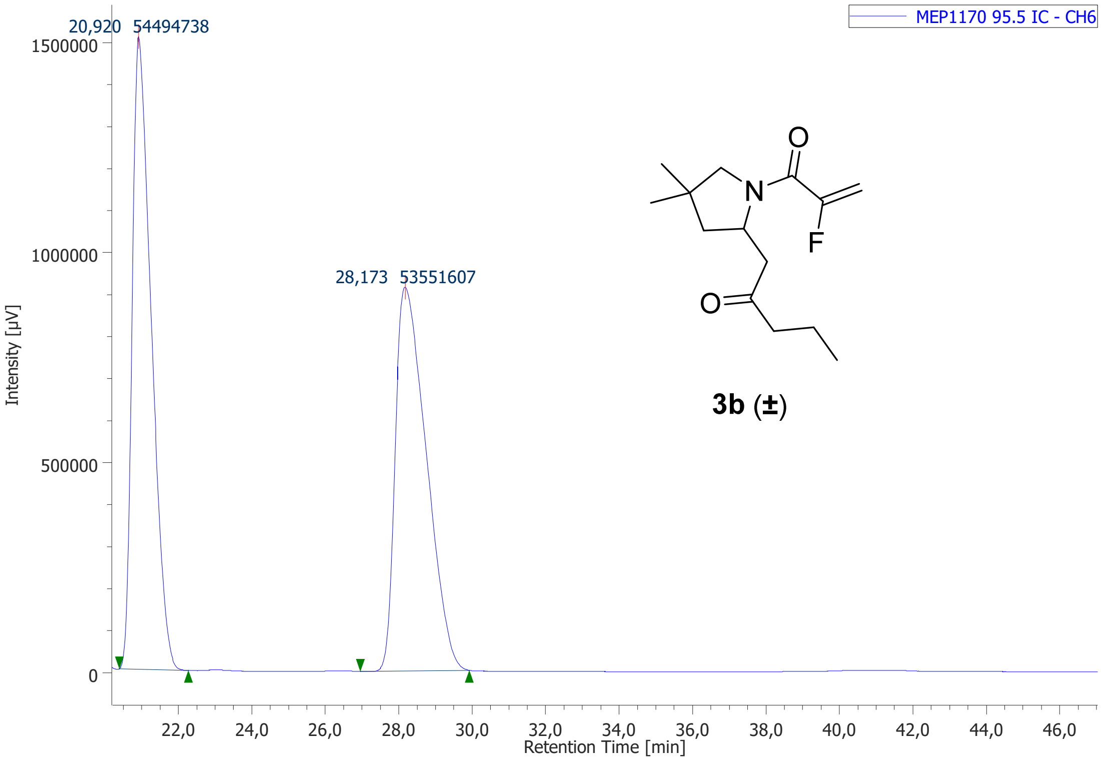

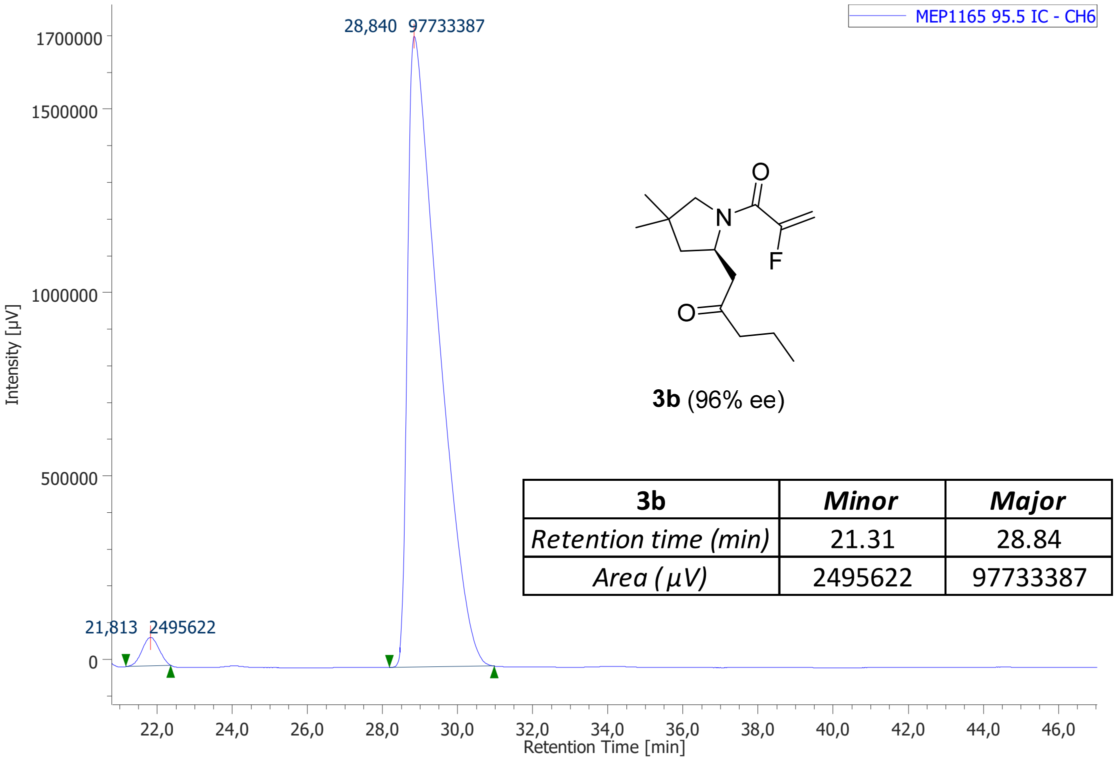

3b	*Minor*	*Major*
Retention time (min)	21.31	28.84
Area (µV)	2495622	97733387

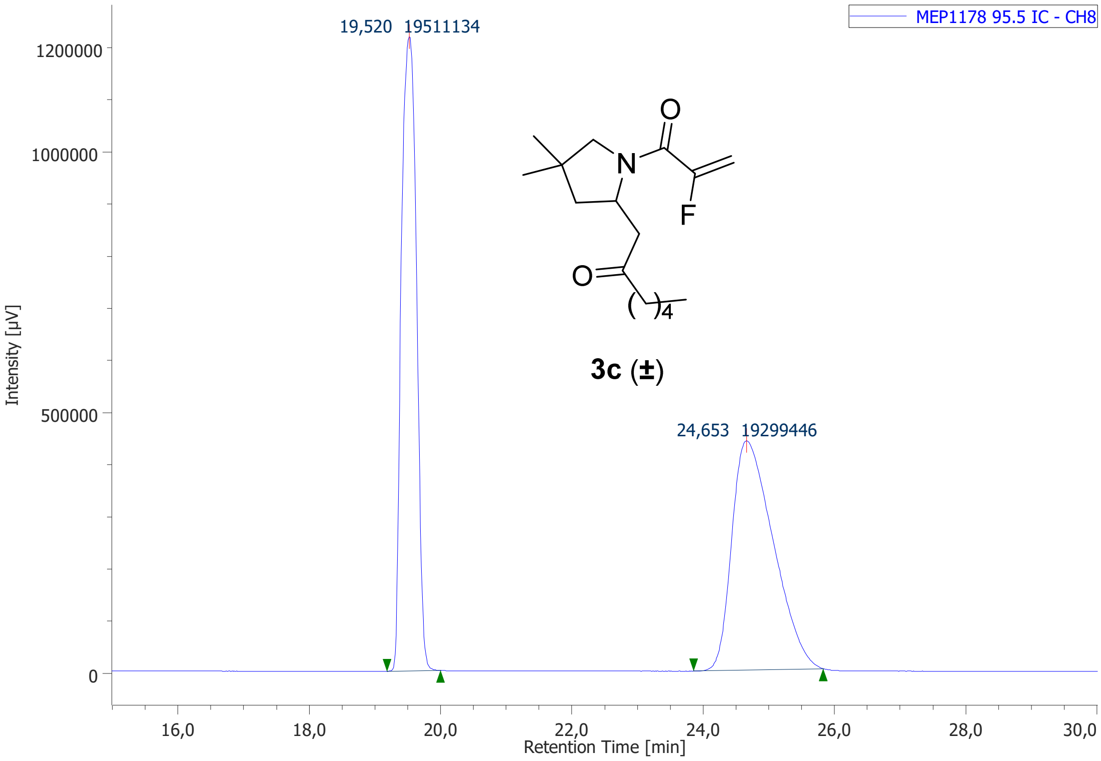

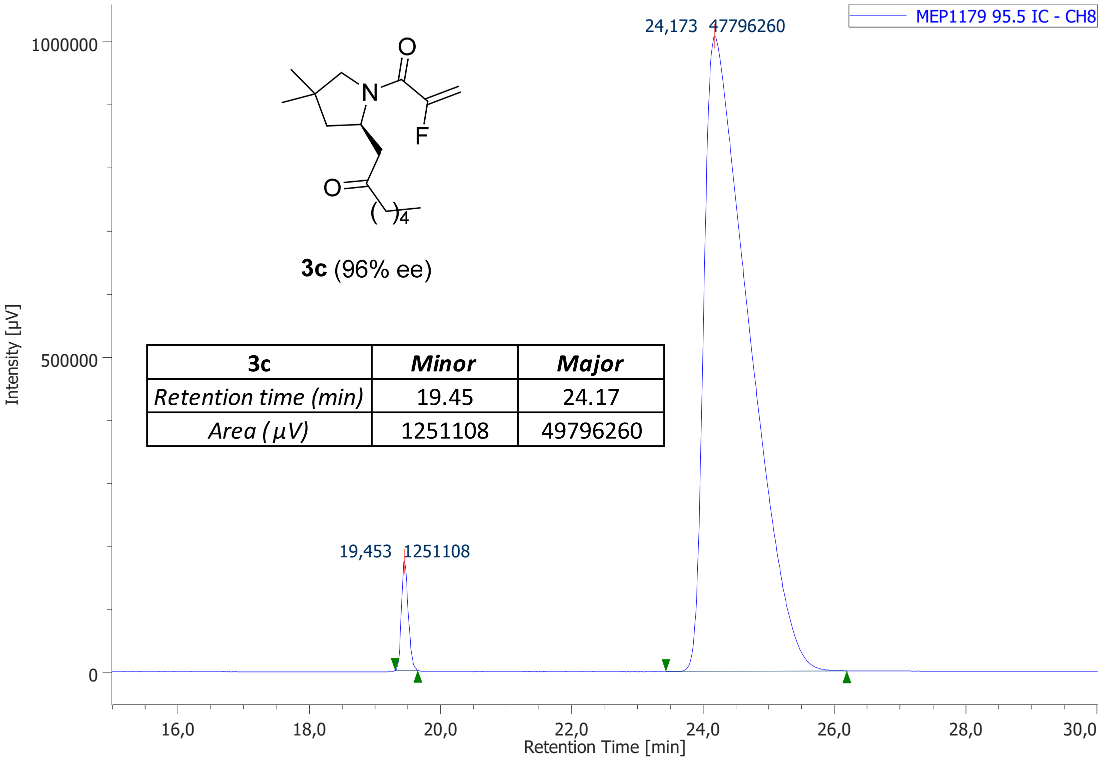

**3c**	***Minor***	***Major***
Retention time (min)	19.45	24.17
Area (µV)	1251108	49796260

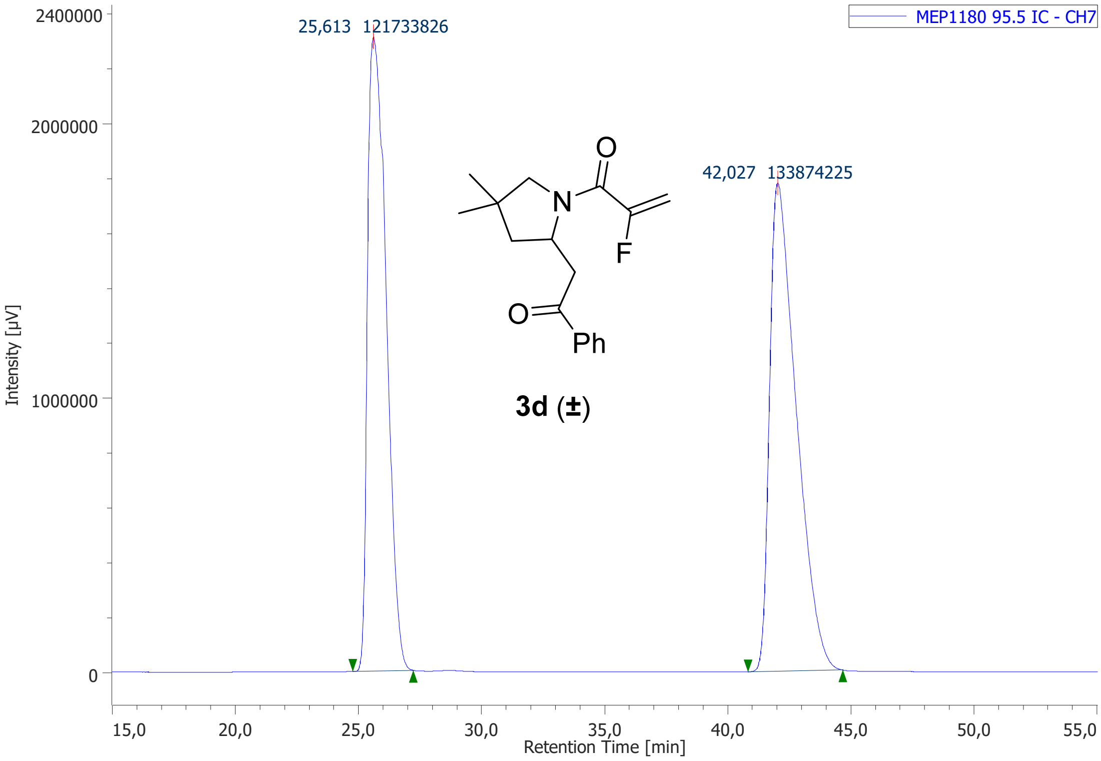

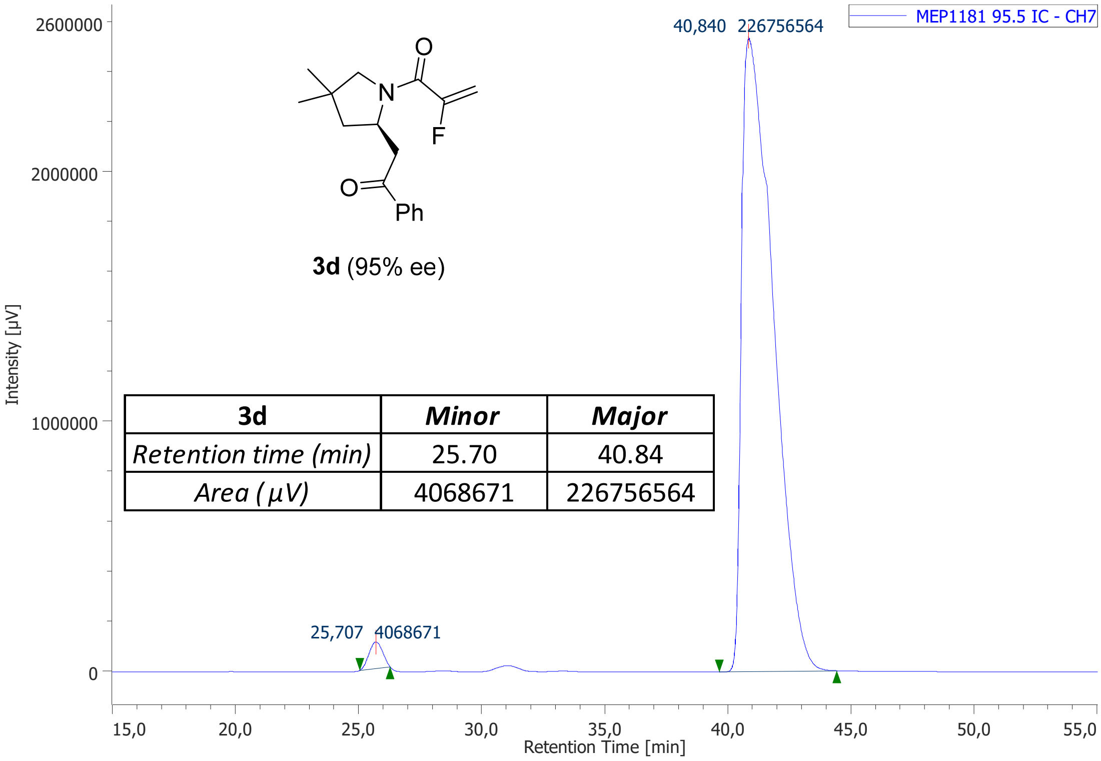

**3d**	***Minor***	***Major***
Retention time (min)	25.70	40.84
Area (µV)	4068671	226756564

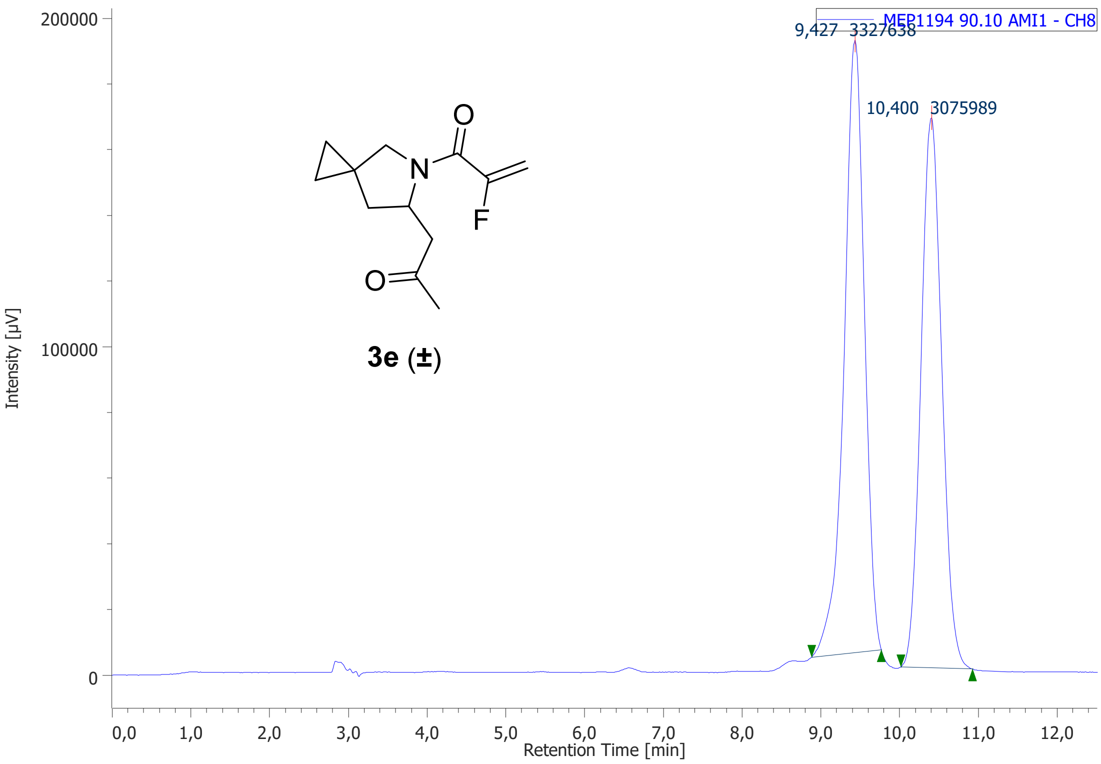

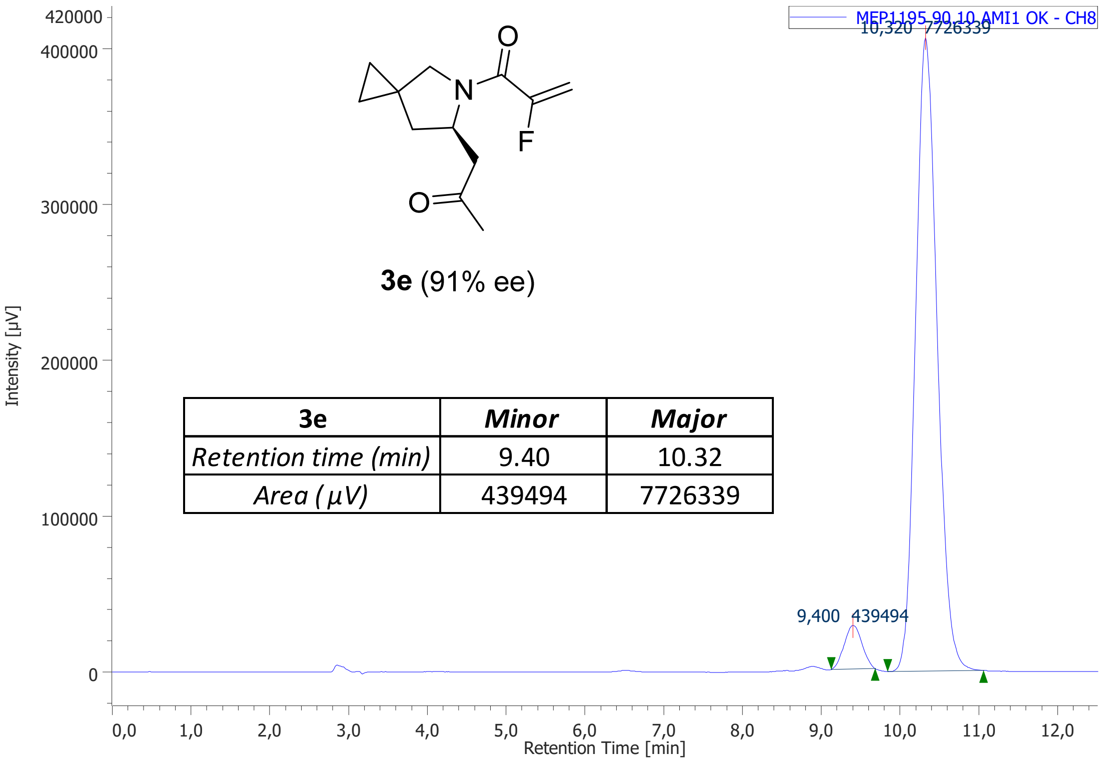

**3e**	***Minor***	***Major***
Retention time (min)	9.40	10.32
Area (µV)	439494	7726339

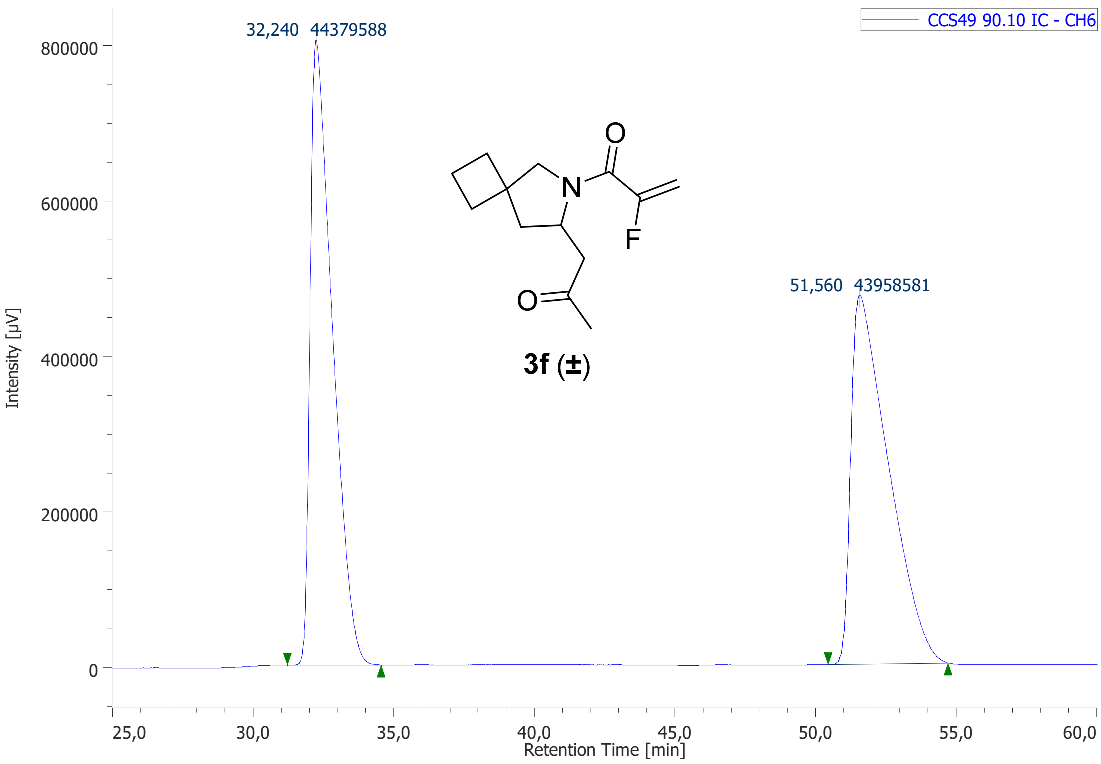

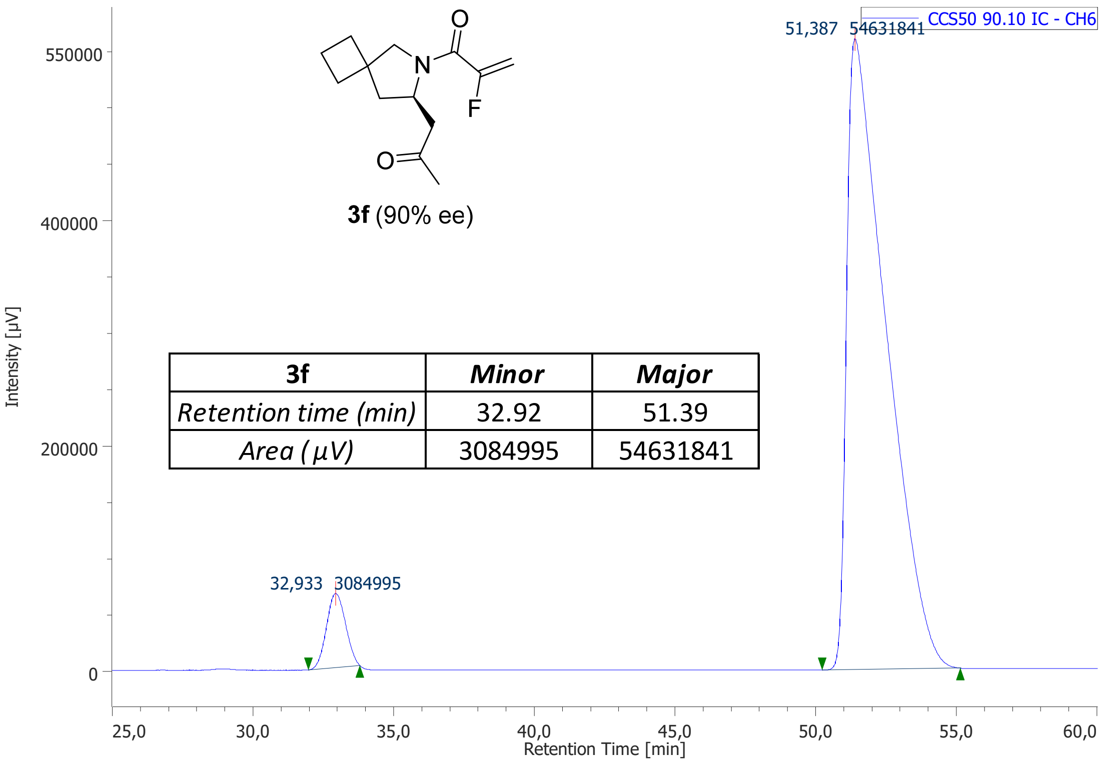

3f	*Minor*	*Major*
Retention time (min)	32.92	51.39
Area (µV)	3084995	54631841

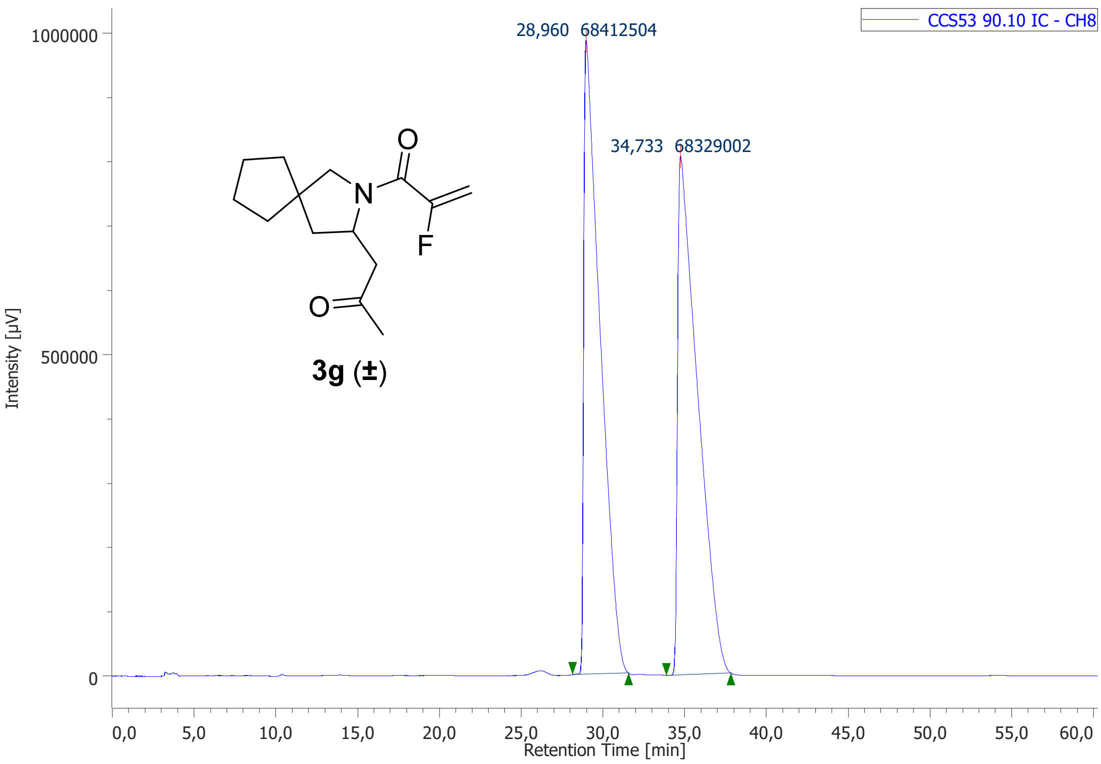

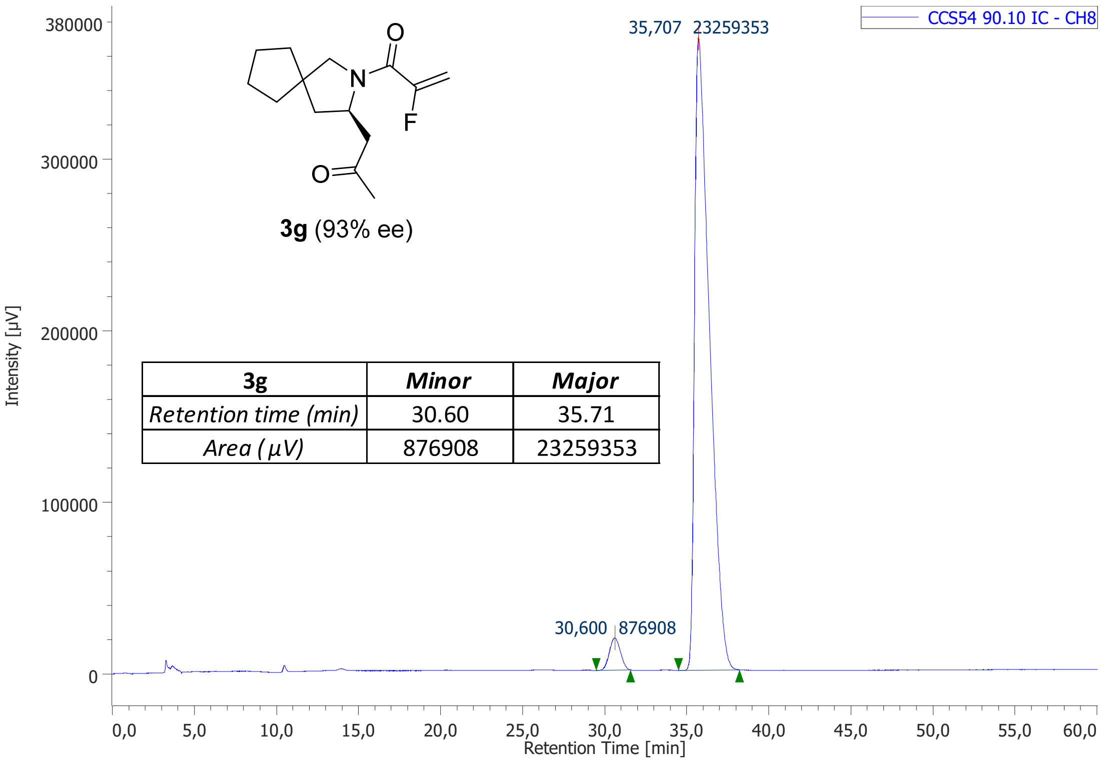

**3g**	***Minor***	***Major***
Retention time (min)	30.60	35.71
Area (µV)	876908	23259353

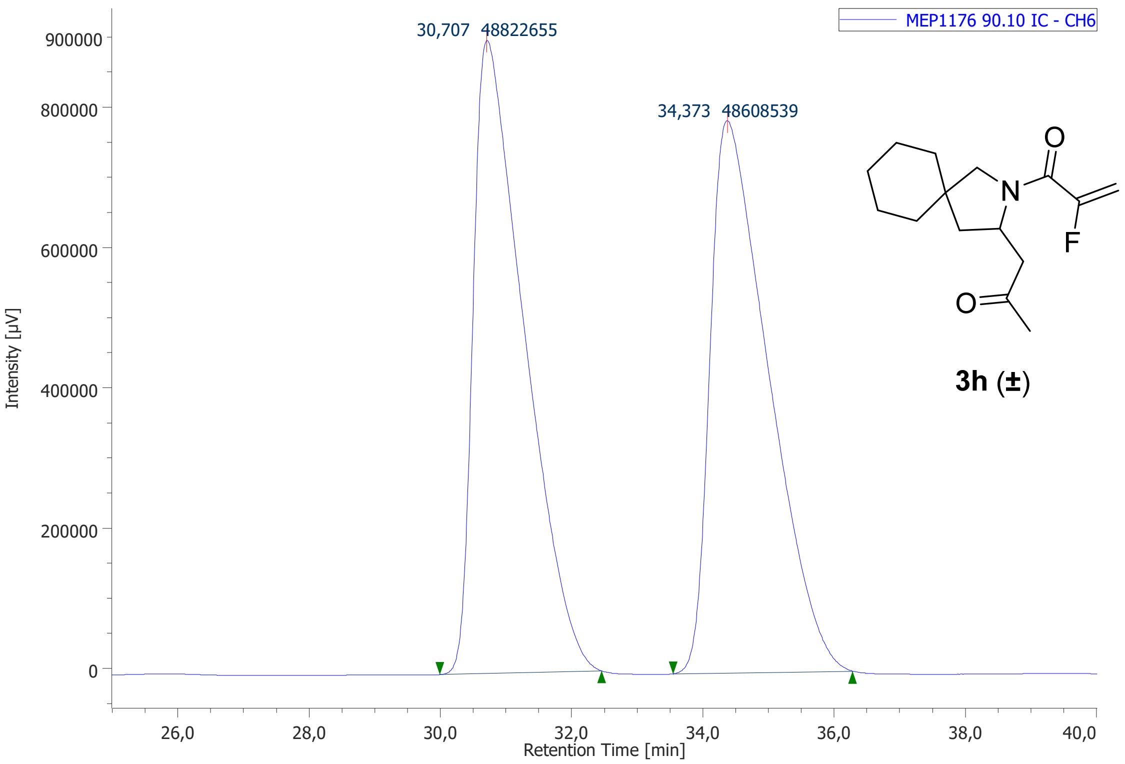

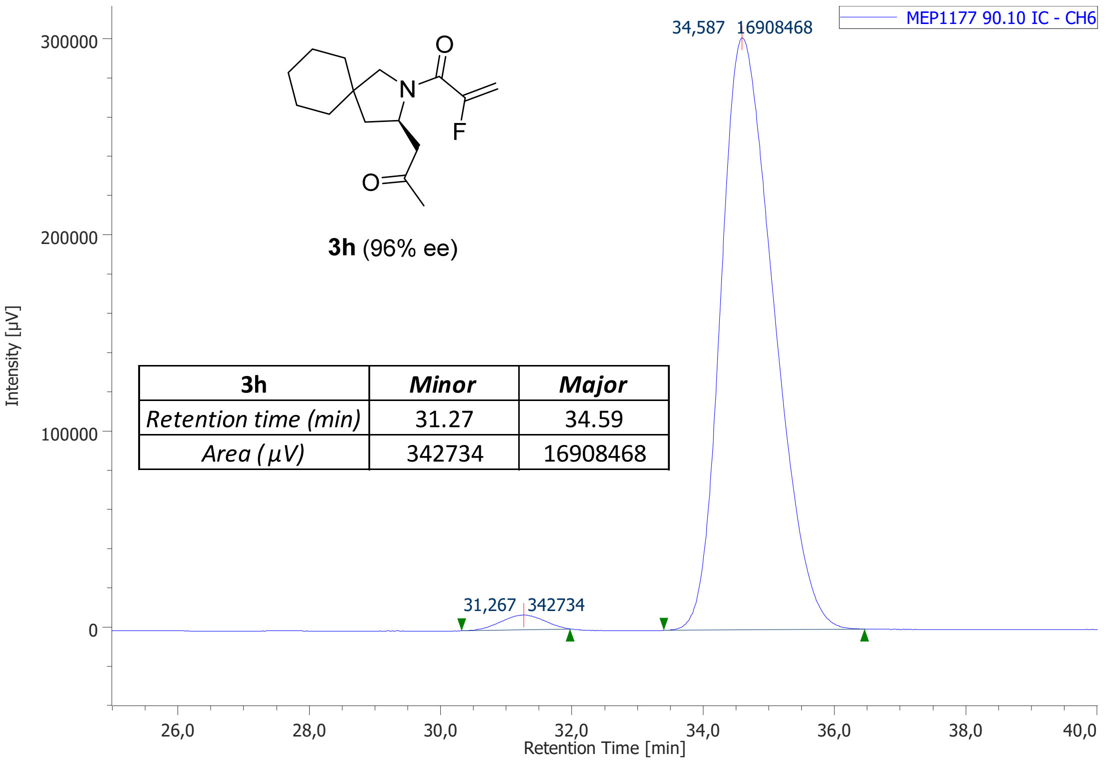

**3h**	***Minor***	***Major***
Retention time (min)	31.27	34.59
Area (µV)	342734	16908468

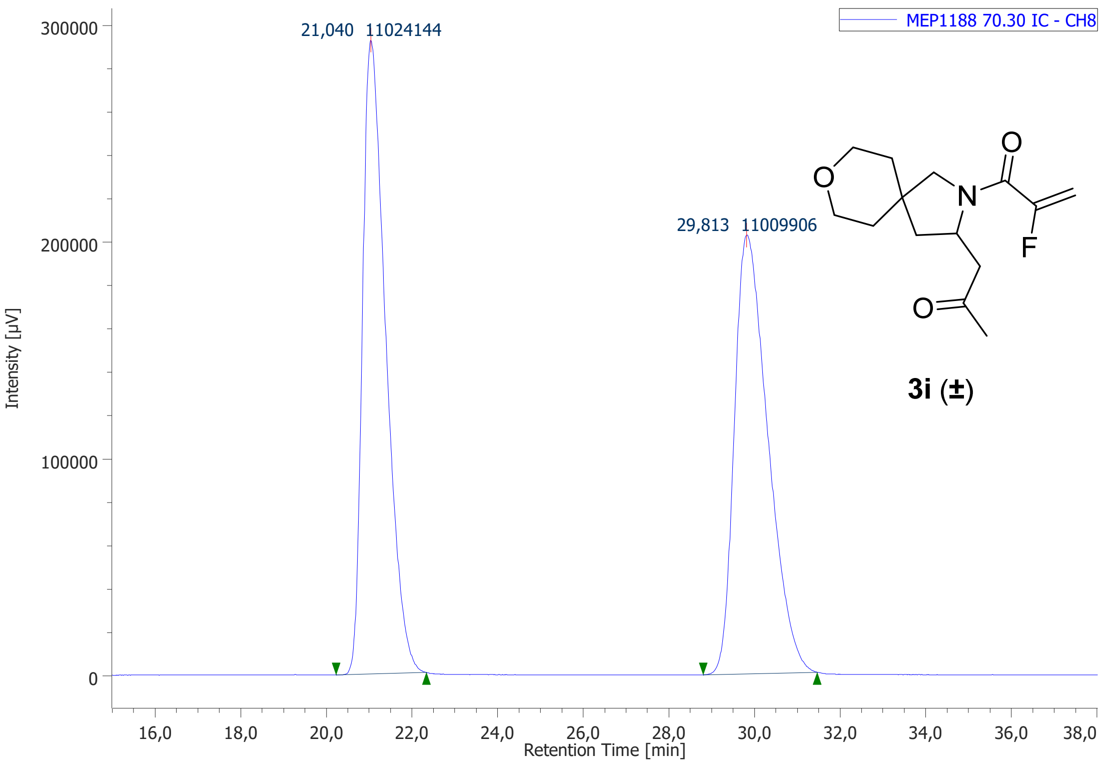
MEP1188 70.30 IC - CH8
21,040 11024144
29,813 11009906
3i (±)
Intensity [µV]
Retention Time [min]

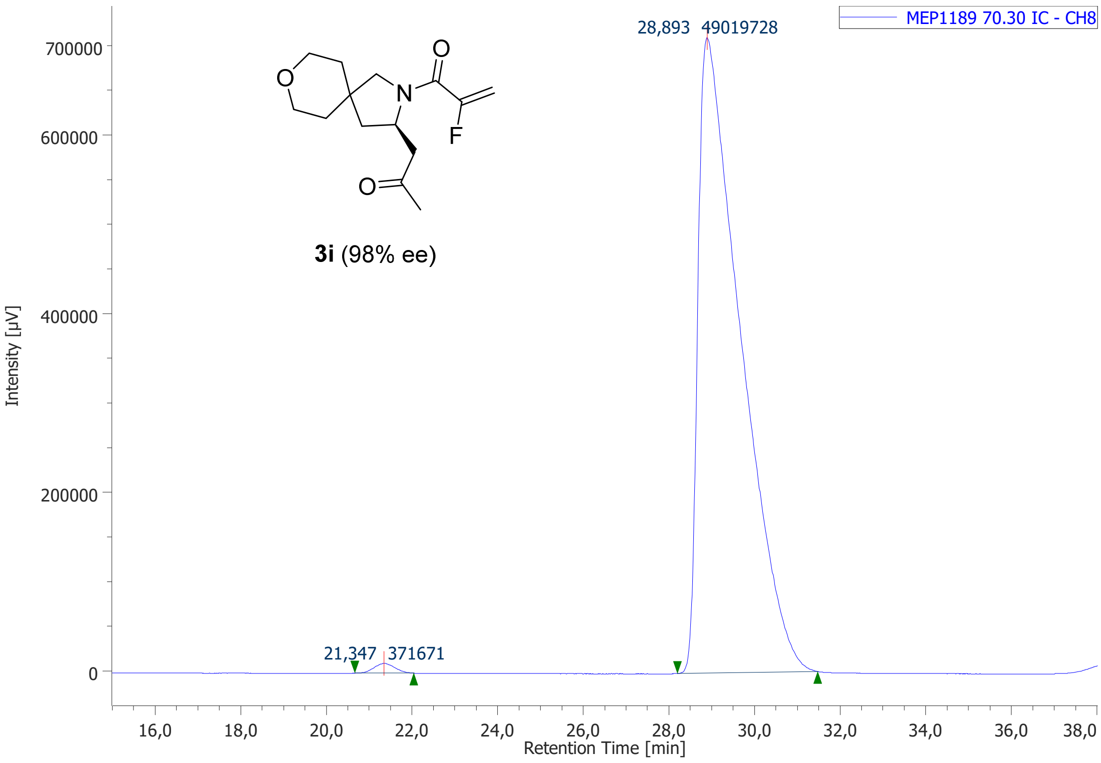
MEP1189 70.30 IC - CH8
28,893 49019728
21,347 371671
3i (98% ee)
Intensity [µV]
Retention Time [min]

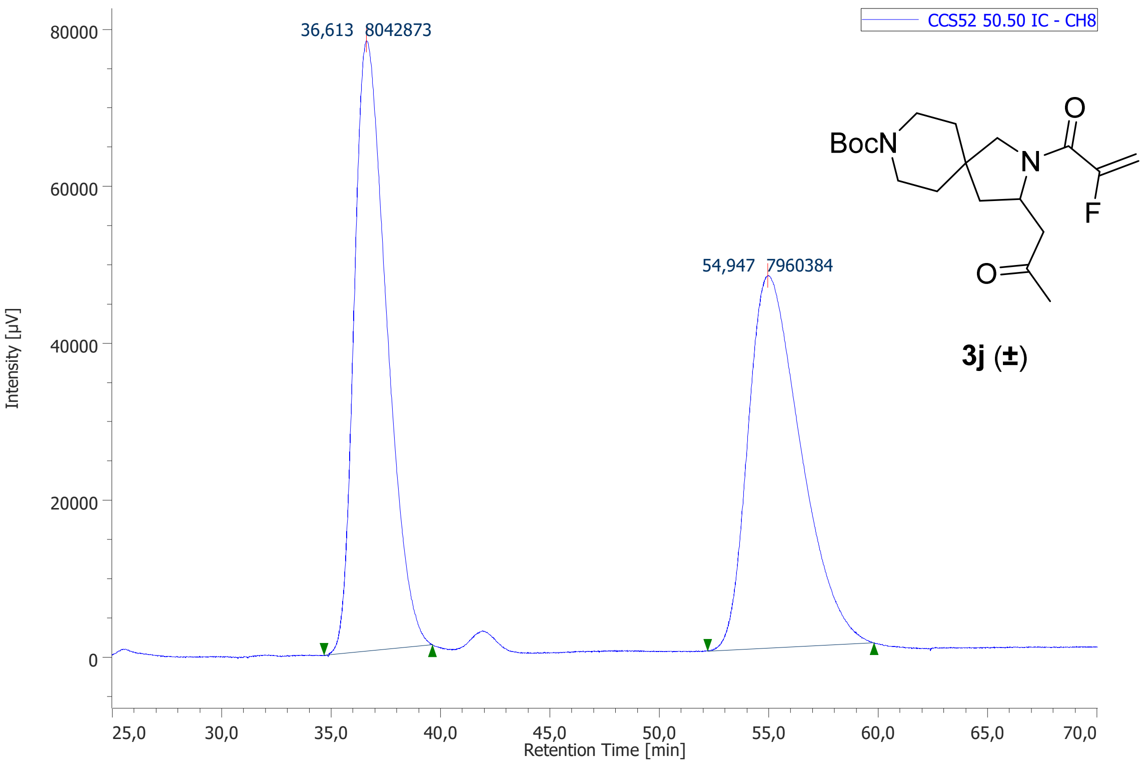

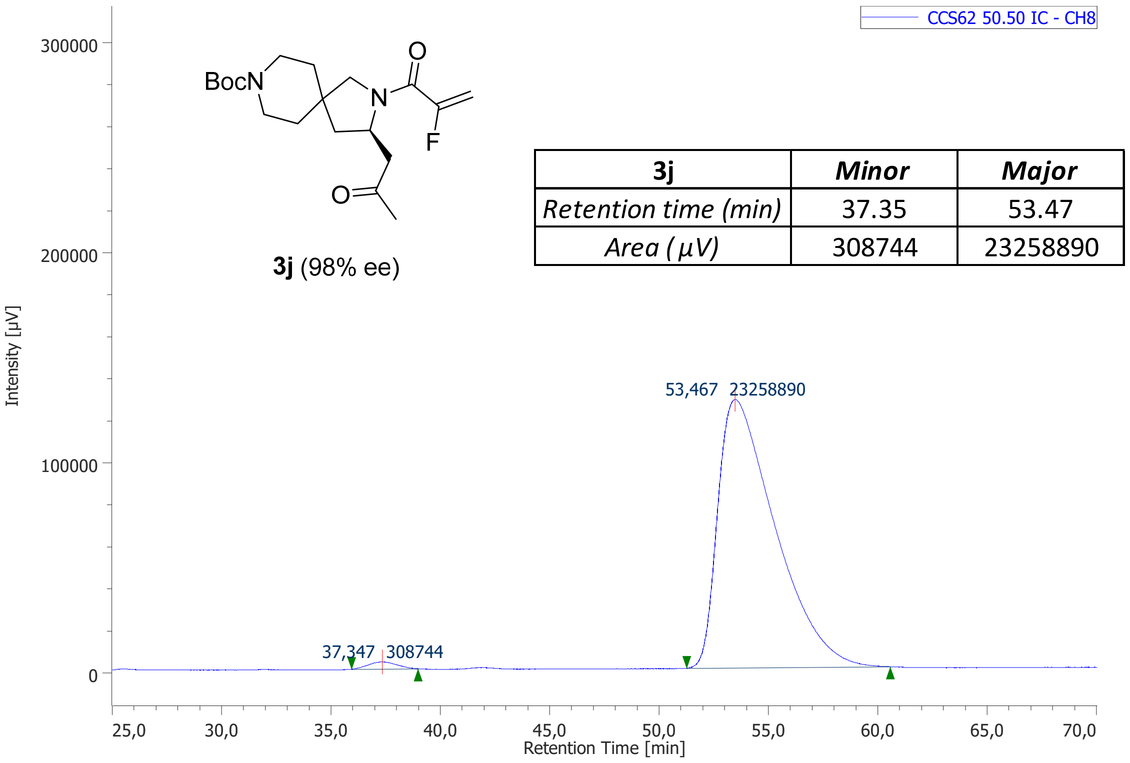

3j	*Minor*	*Major*
Retention time (min)	37.35	53.47
Area (µV)	308744	23258890

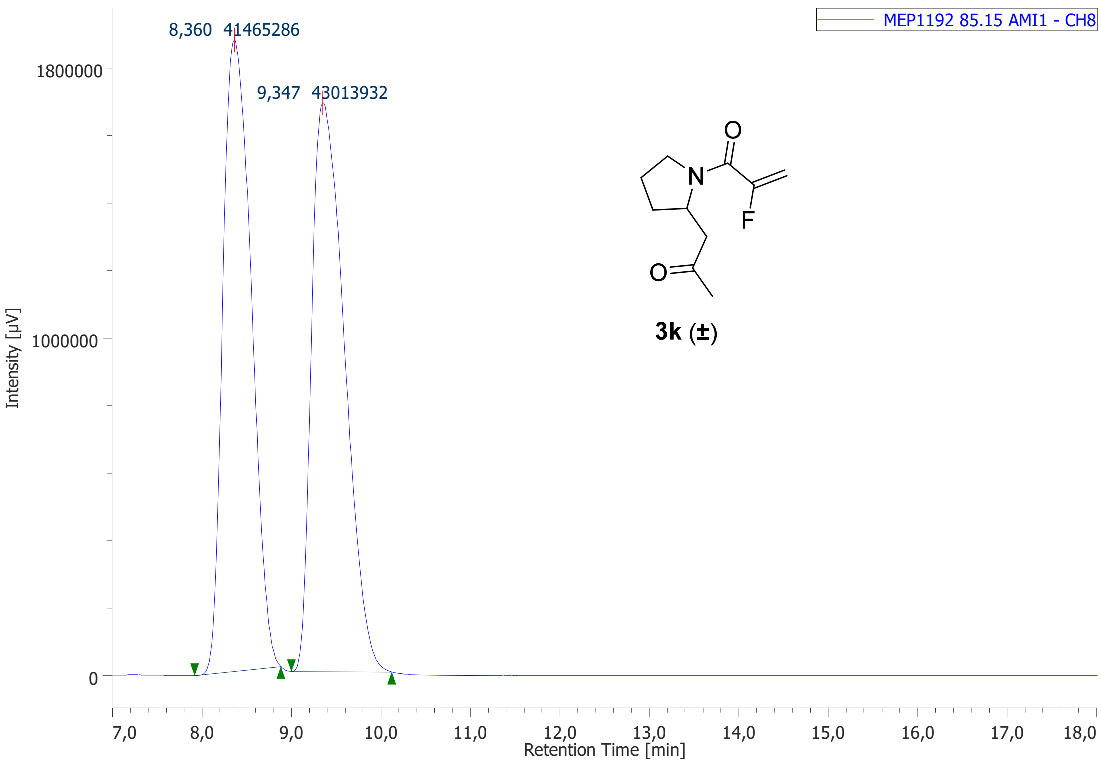

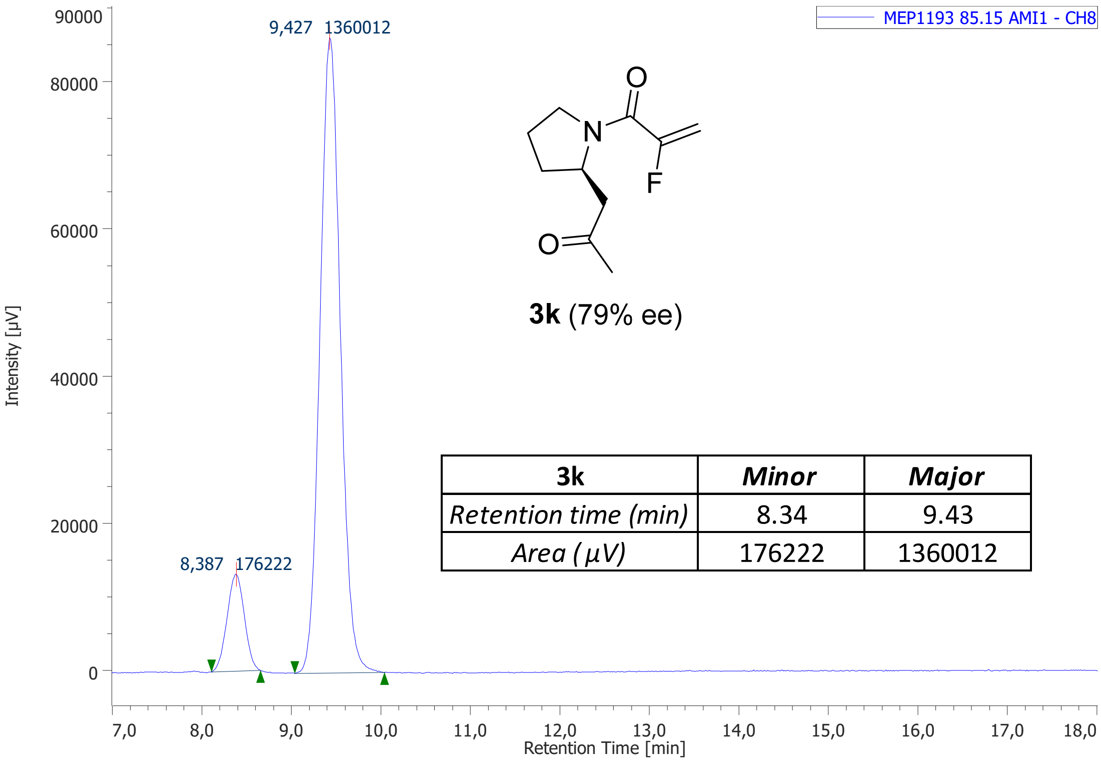

**3k**	***Minor***	***Major***
Retention time (min)	8.34	9.43
Area (µV)	176222	1360012

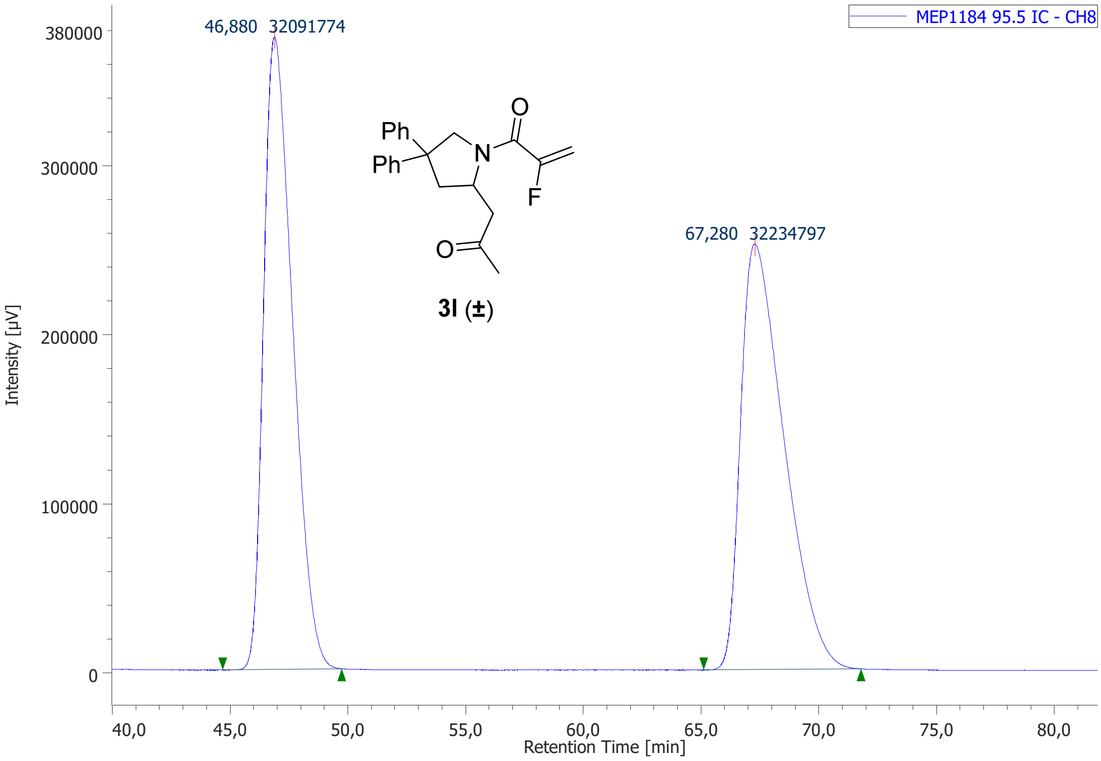

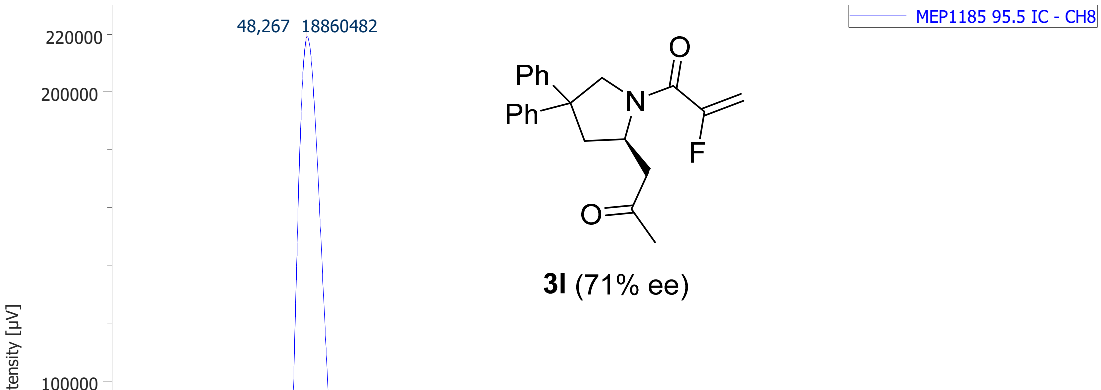

**3l**	***Minor***	***Major***
Retention time (min)	70.17	48.26
Area (µV)	2631707	18860482

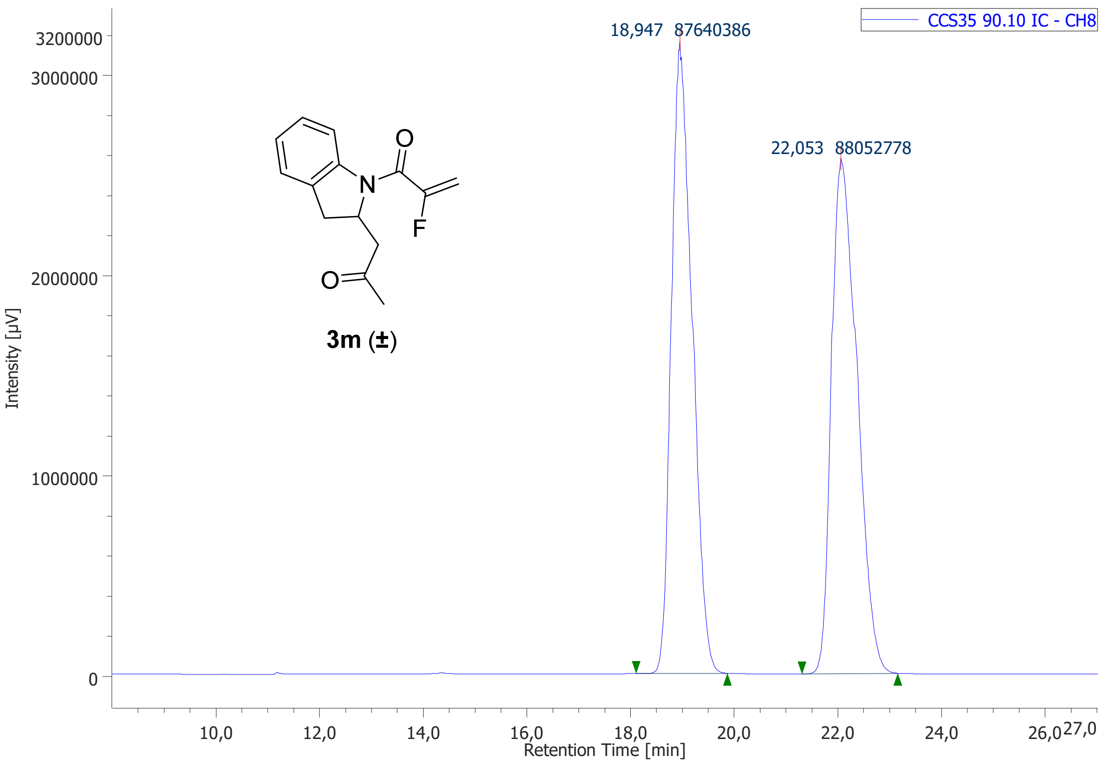

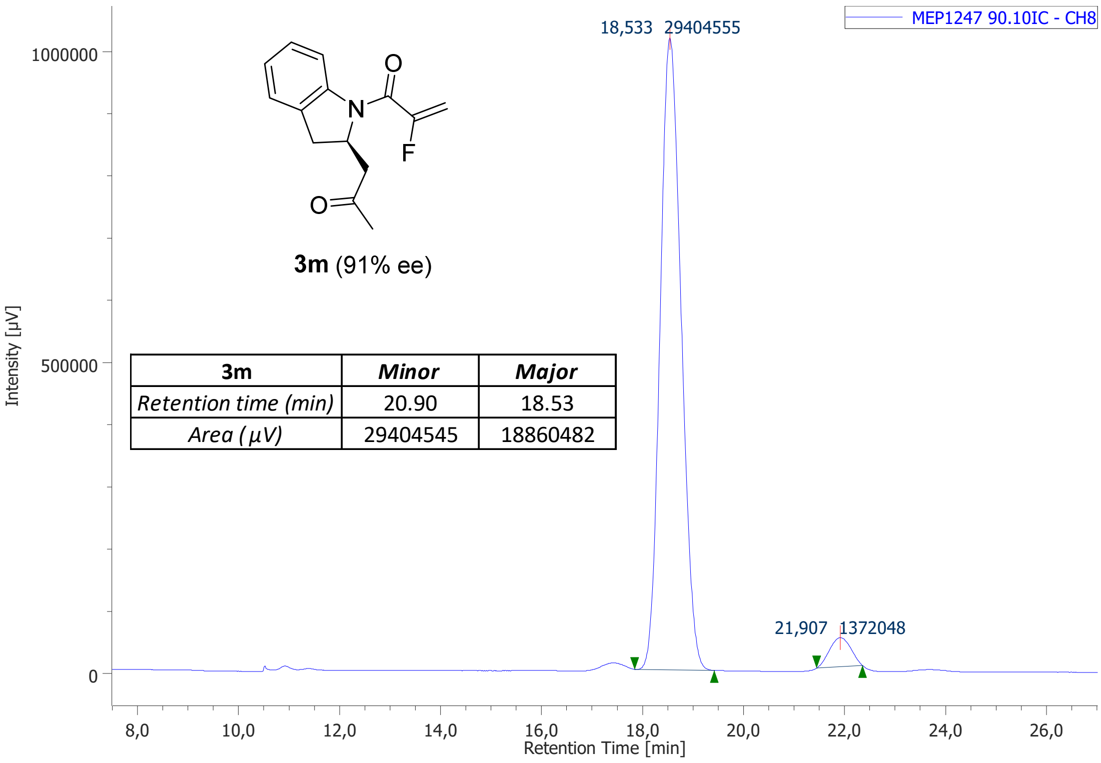

**3m**	***Minor***	***Major***
Retention time (min)	20.90	18.53
Area (µV)	29404545	18860482

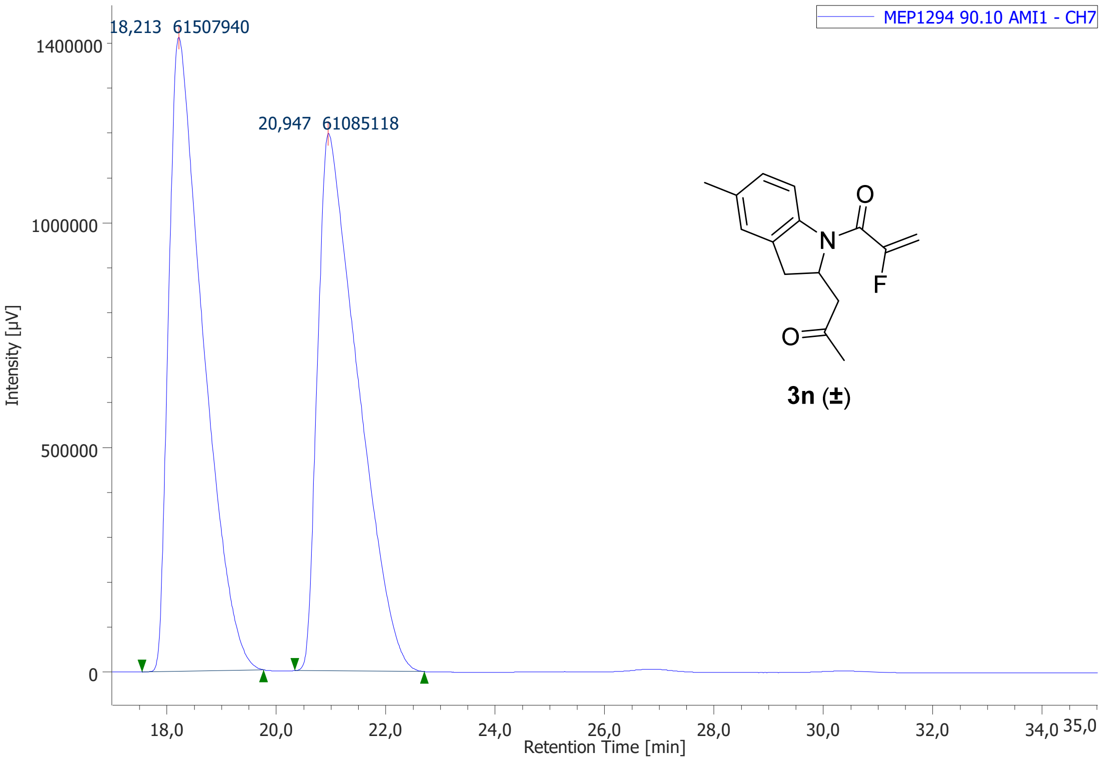

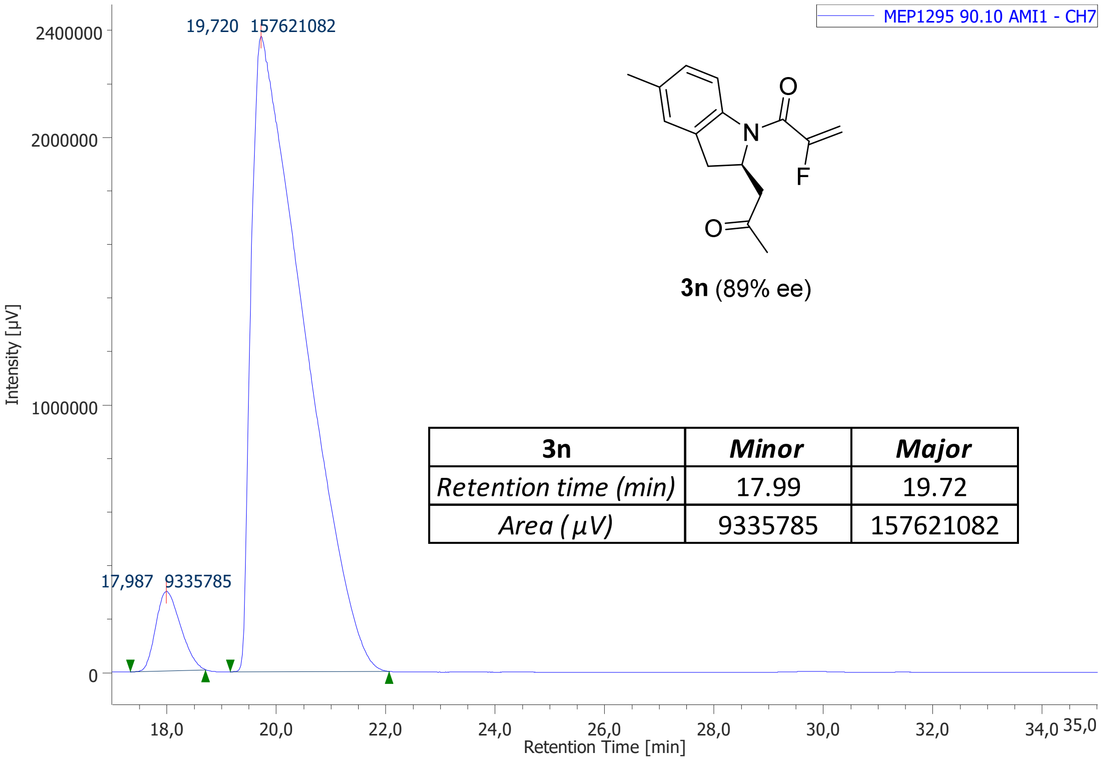

**3n**	***Minor***	***Major***
Retention time (min)	17.99	19.72
Area (µV)	9335785	157621082

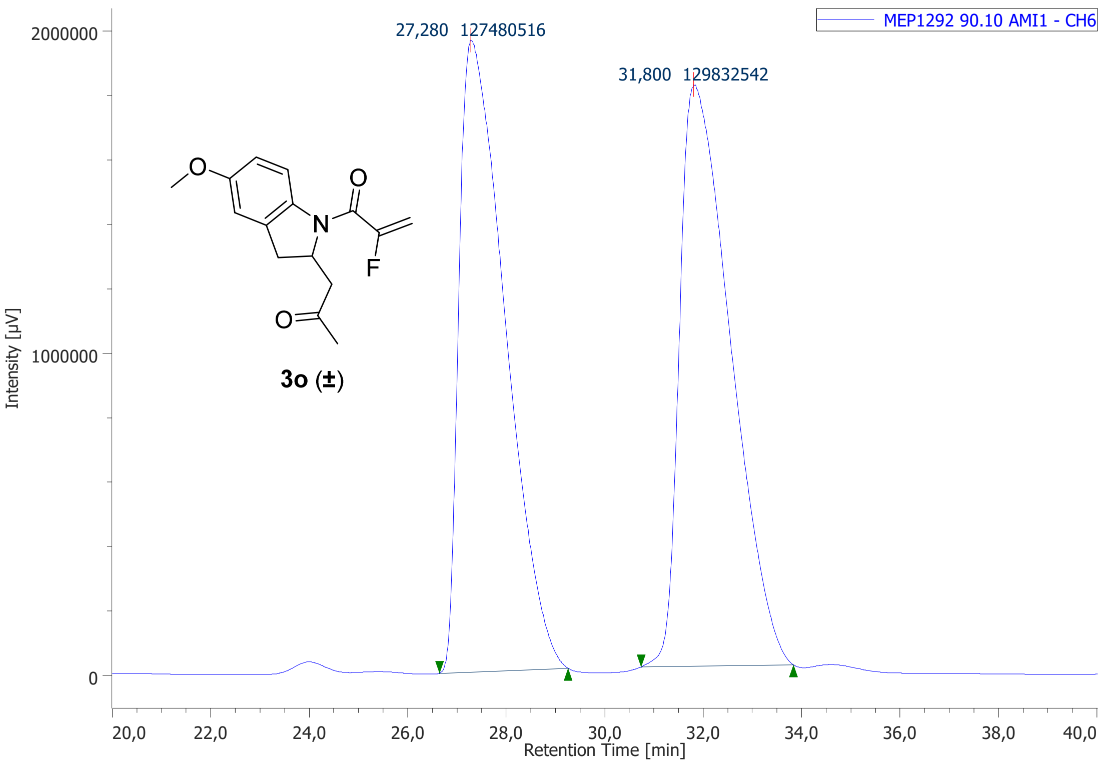

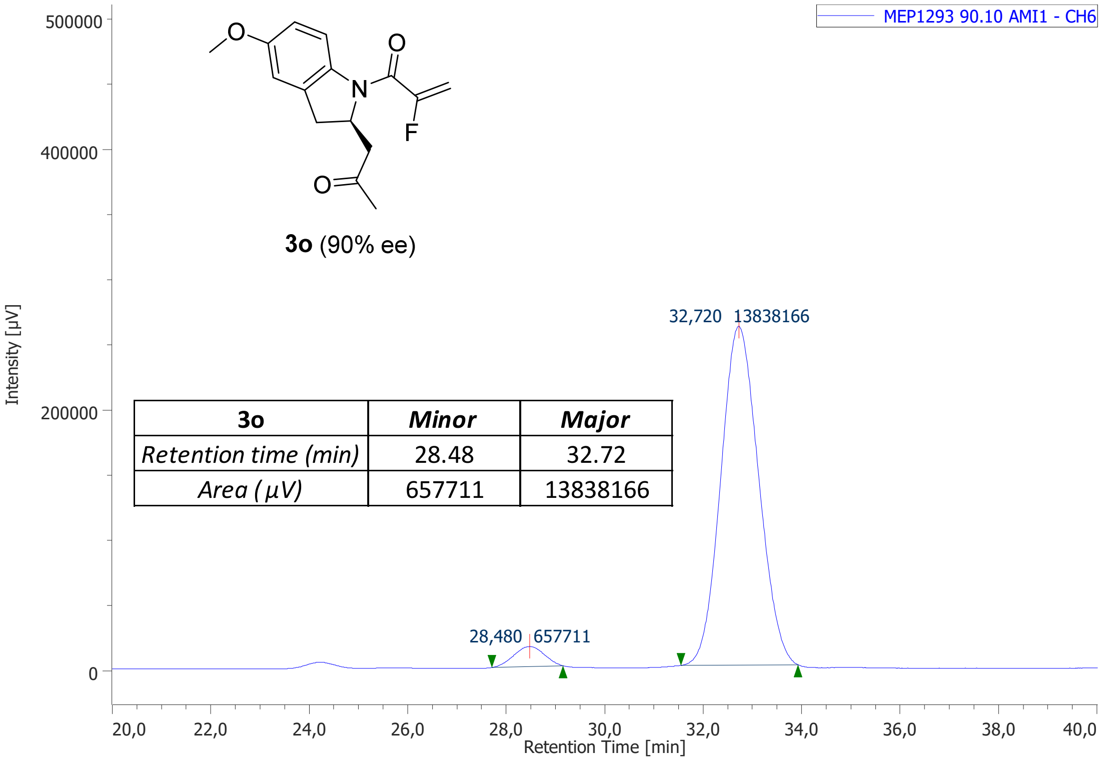

3o	*Minor*	*Major*
Retention time (min)	28.48	32.72
Area (µV)	657711	13838166

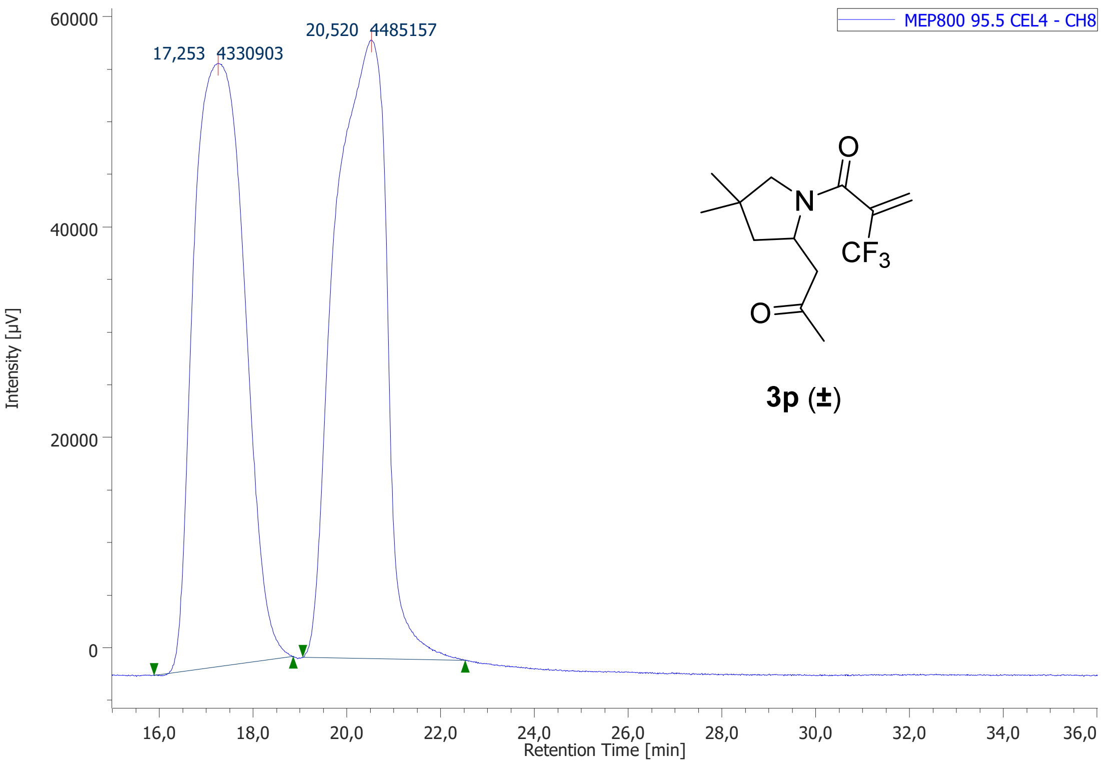

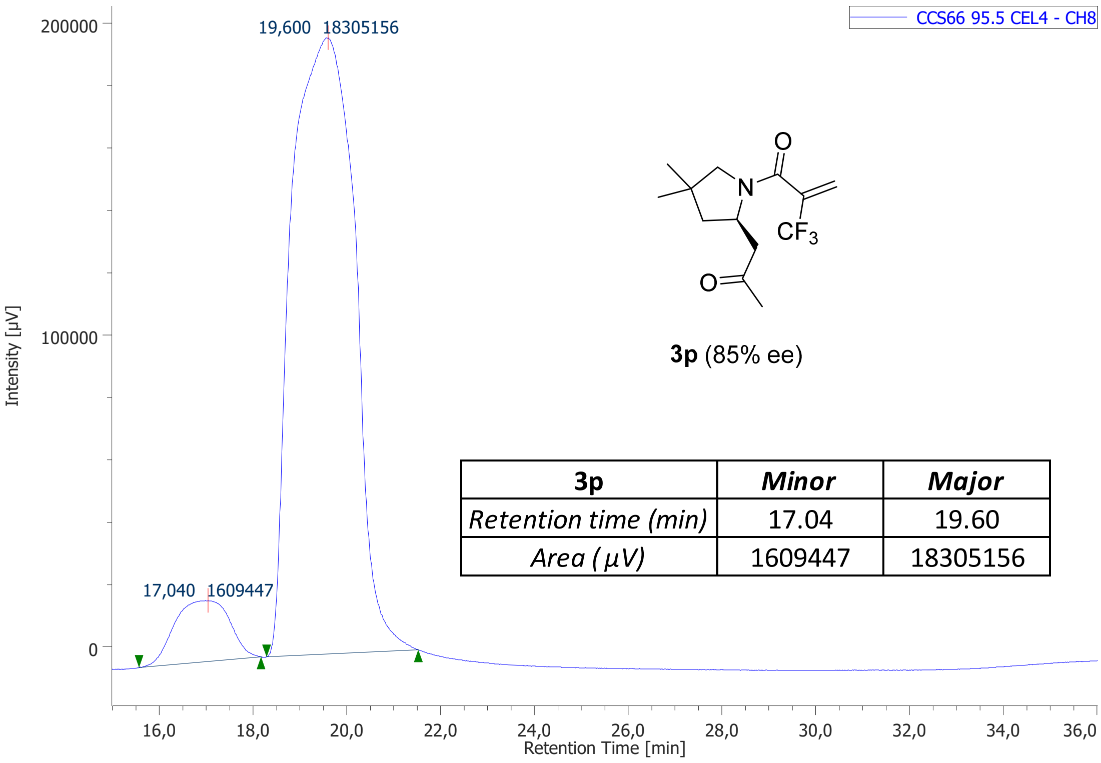

**3p**	***Minor***	***Major***
Retention time (min)	17.04	19.60
Area (µV)	1609447	18305156

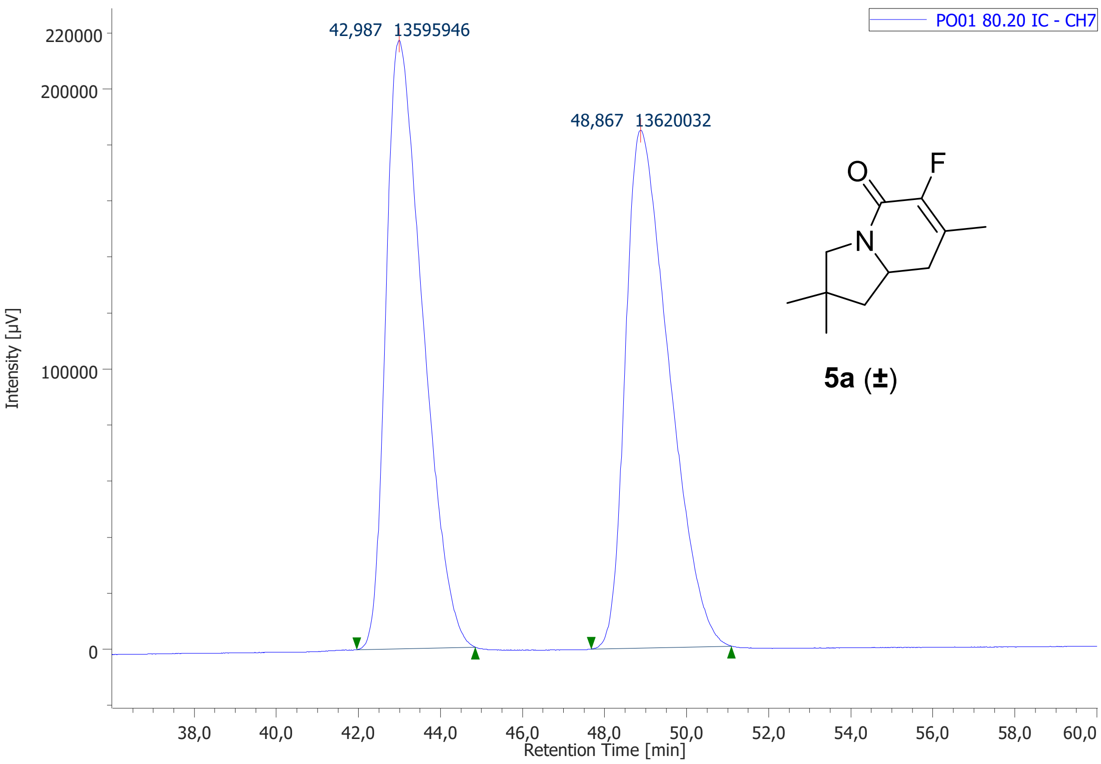

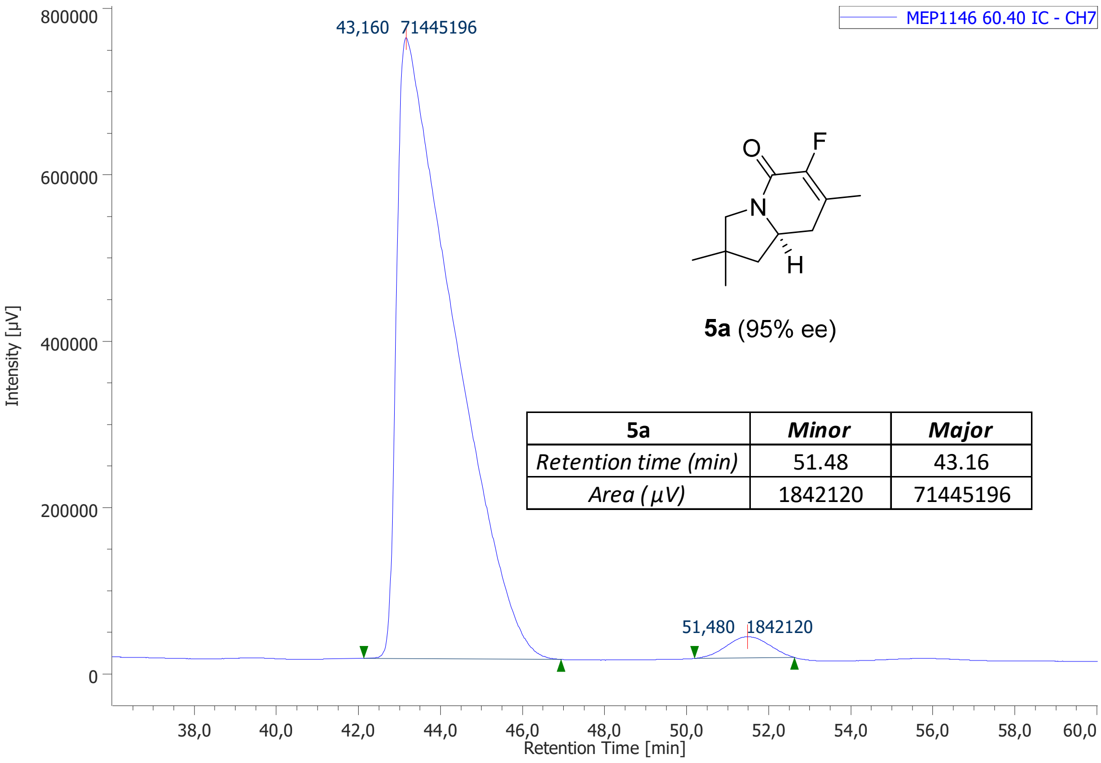

5a	*Minor*	*Major*
Retention time (min)	51.48	43.16
Area (µV)	1842120	71445196

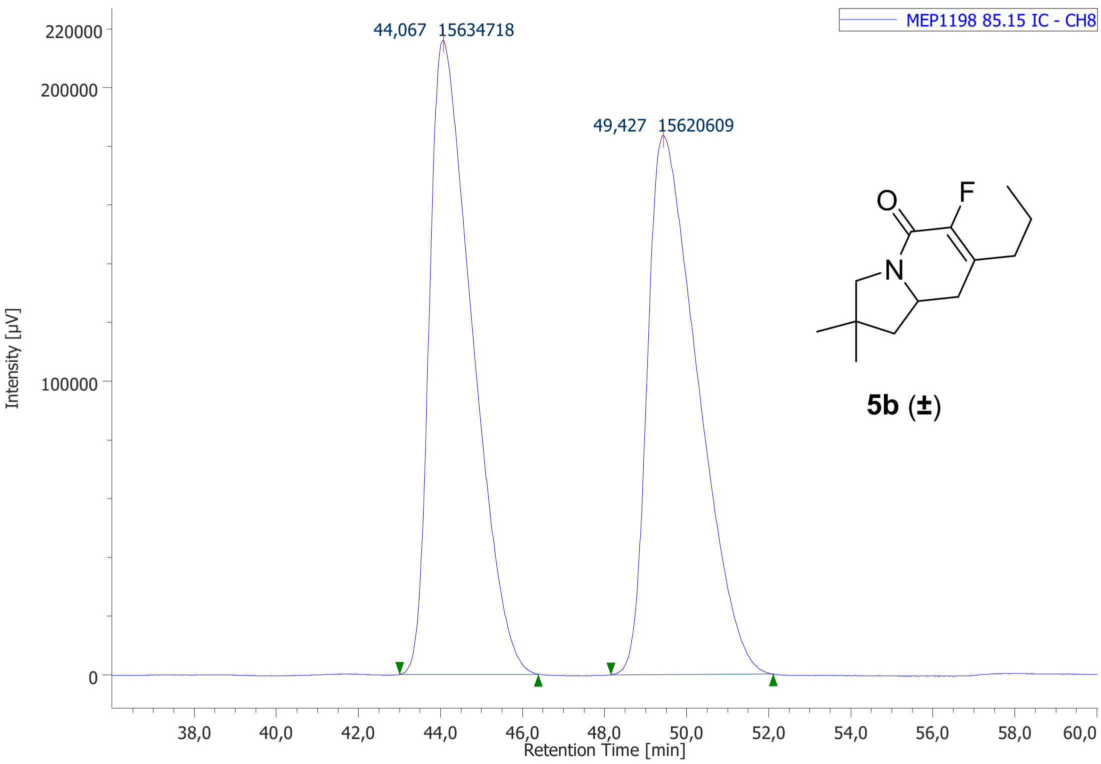

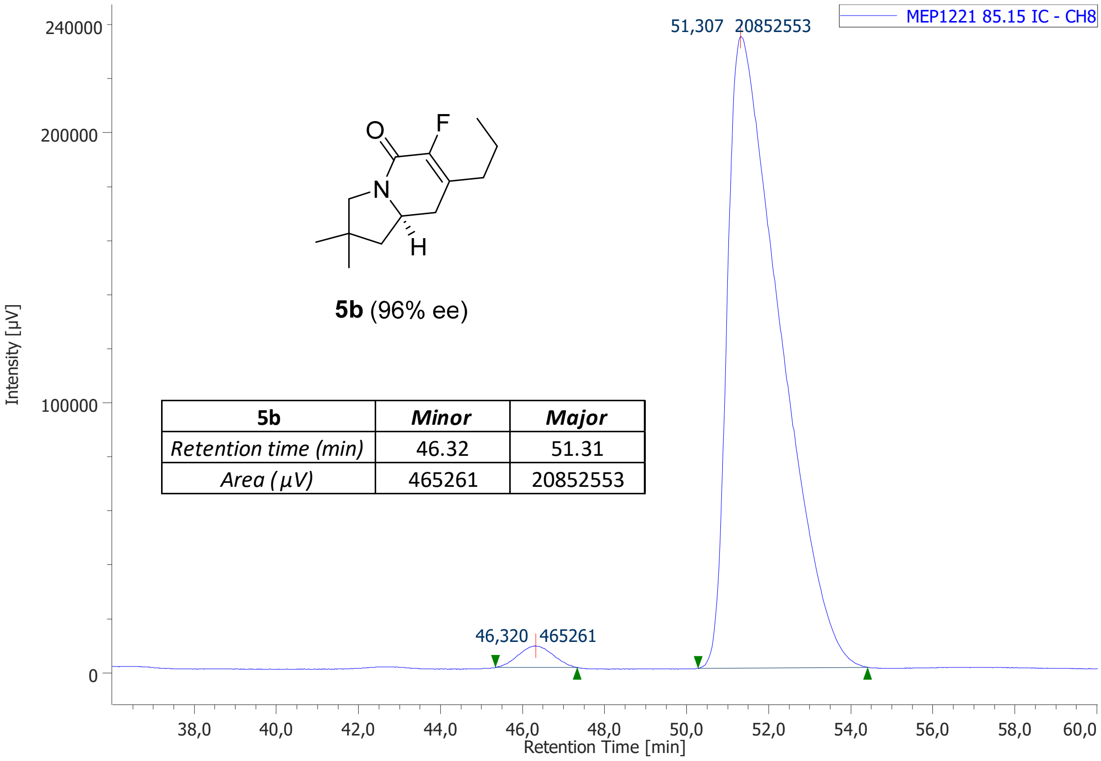

**5b**	***Minor***	***Major***
Retention time (min)	46.32	51.31
Area (µV)	465261	20852553

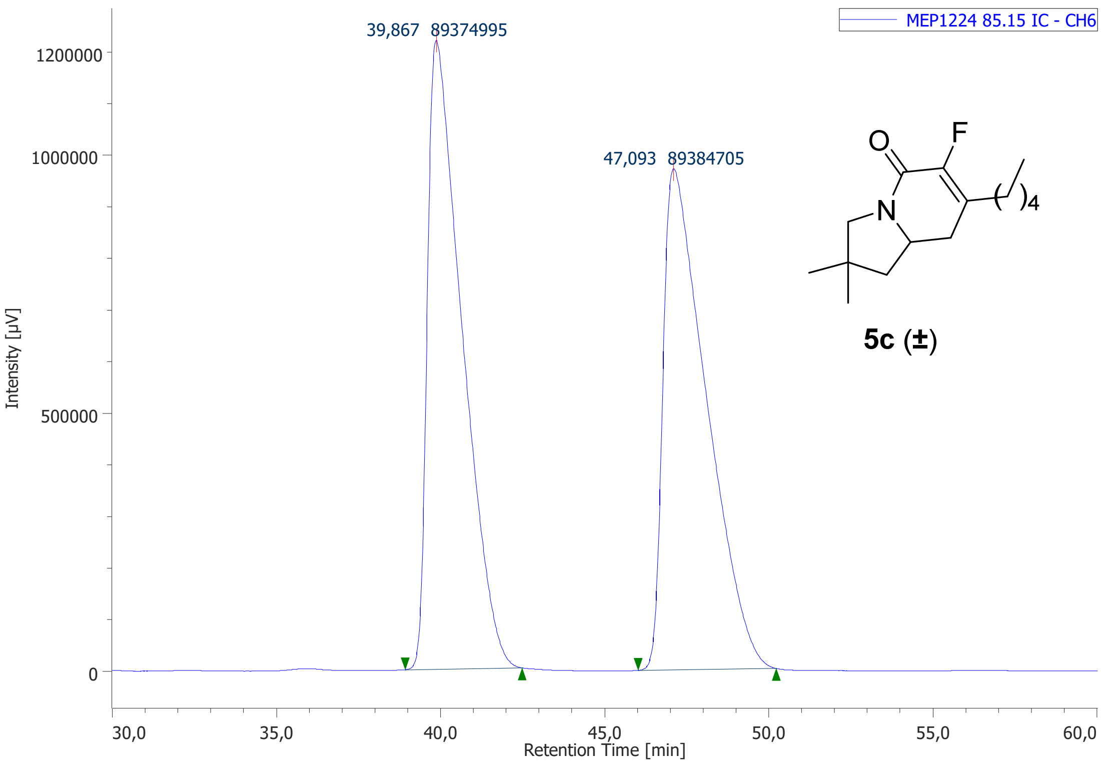

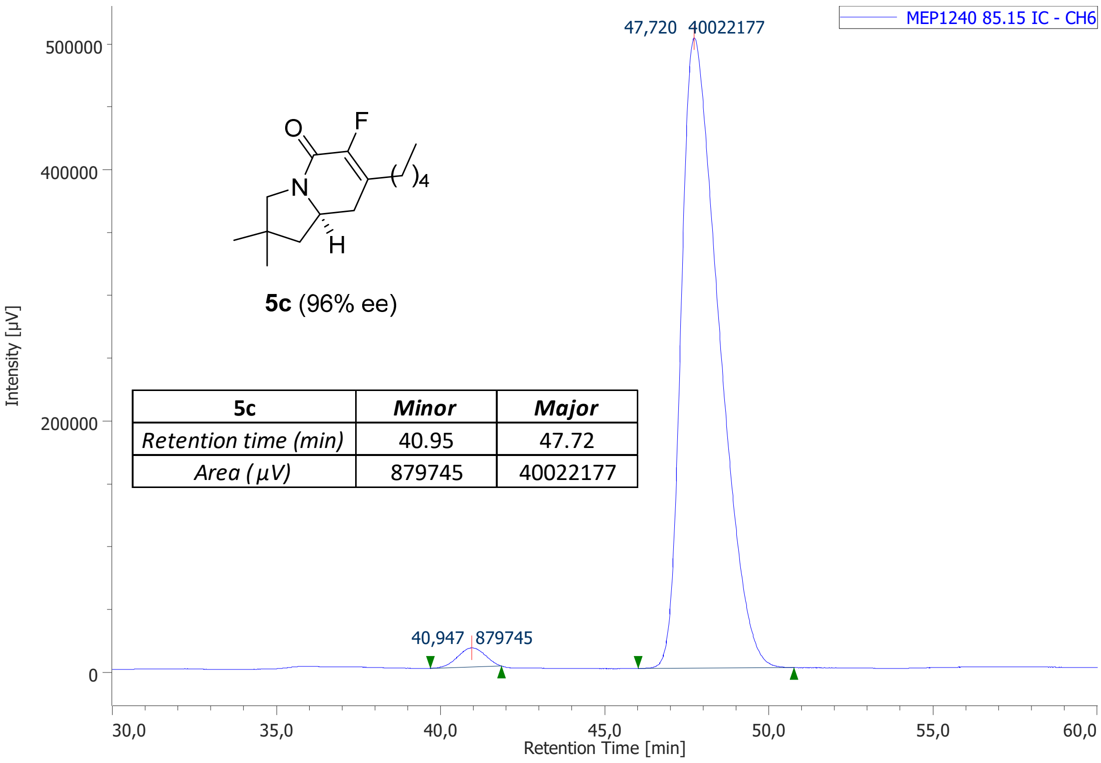

**5c**	***Minor***	***Major***
Retention time (min)	40.95	47.72
Area (µV)	879745	40022177

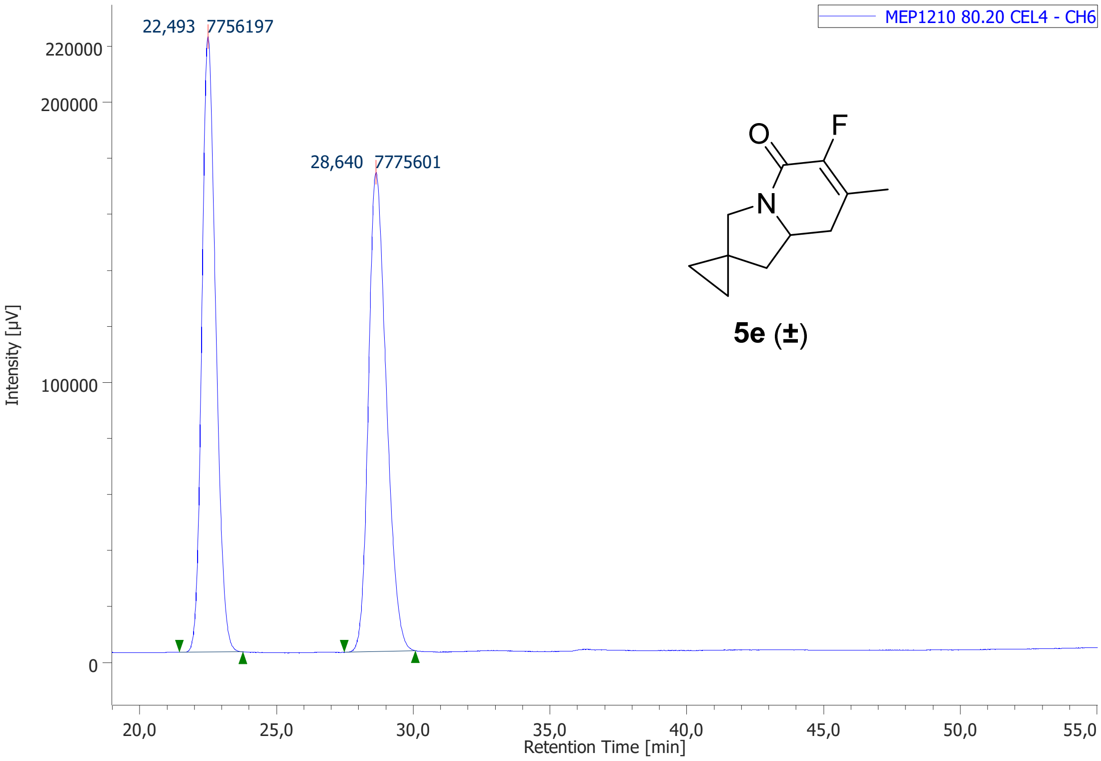

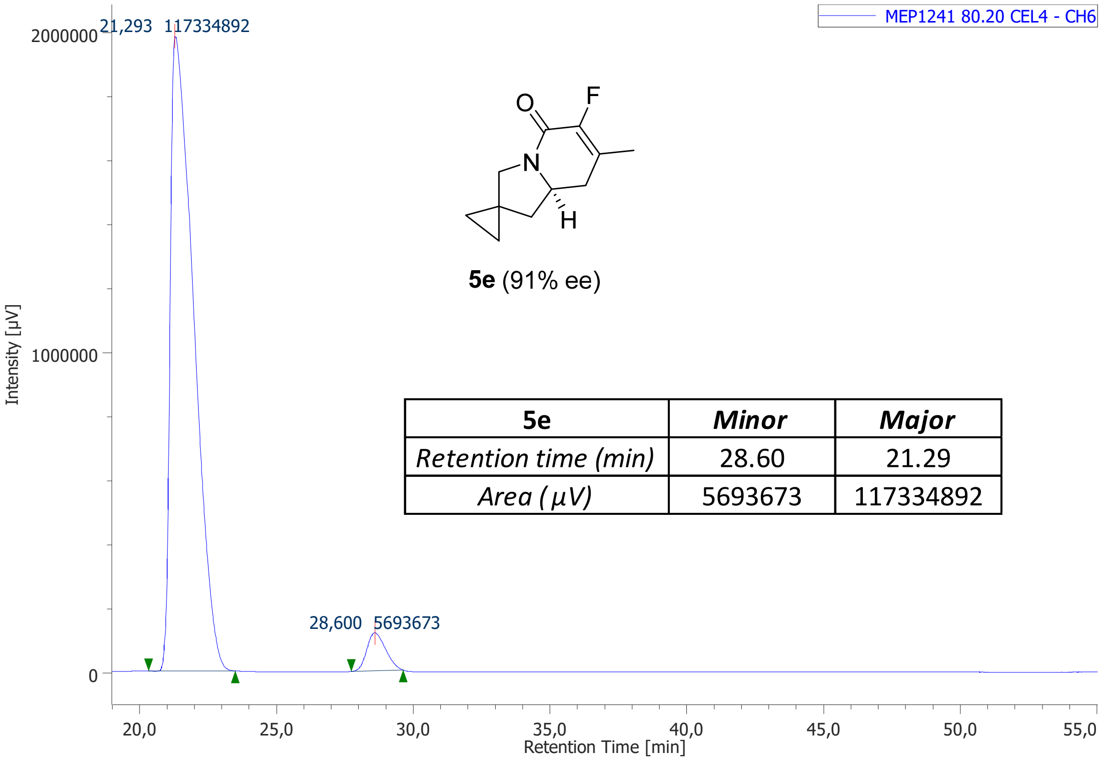

5e	*Minor*	*Major*
Retention time (min)	28.60	21.29
Area (µV)	5693673	117334892

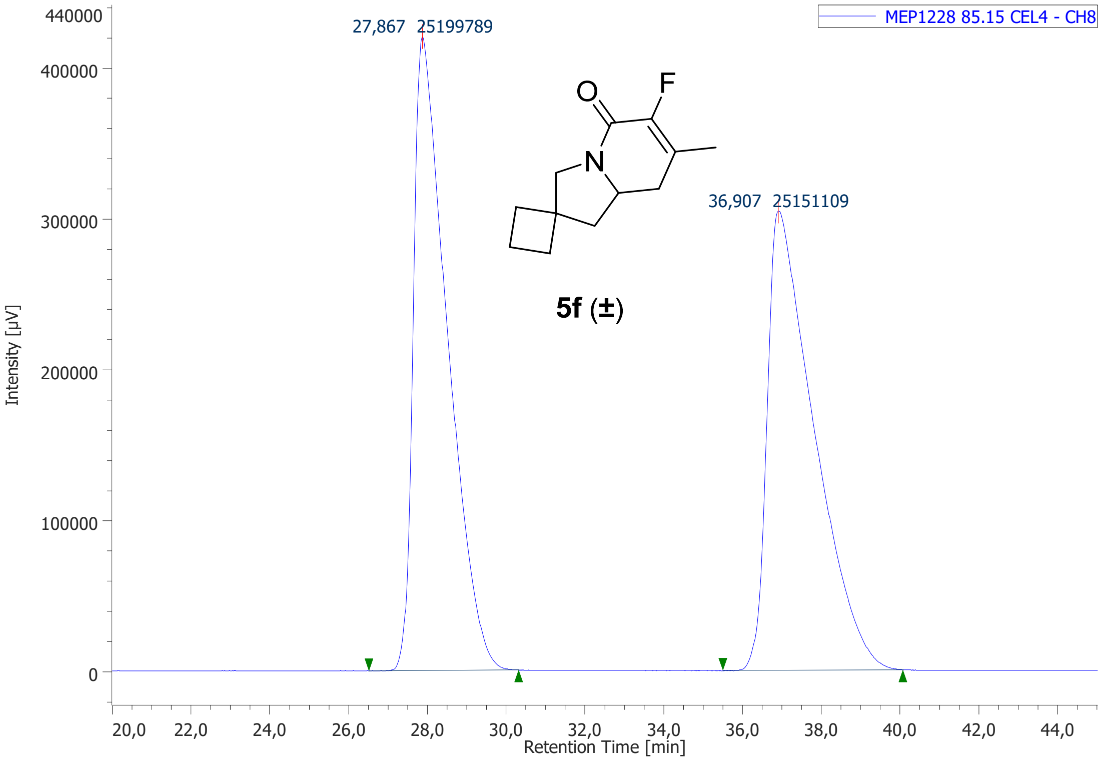

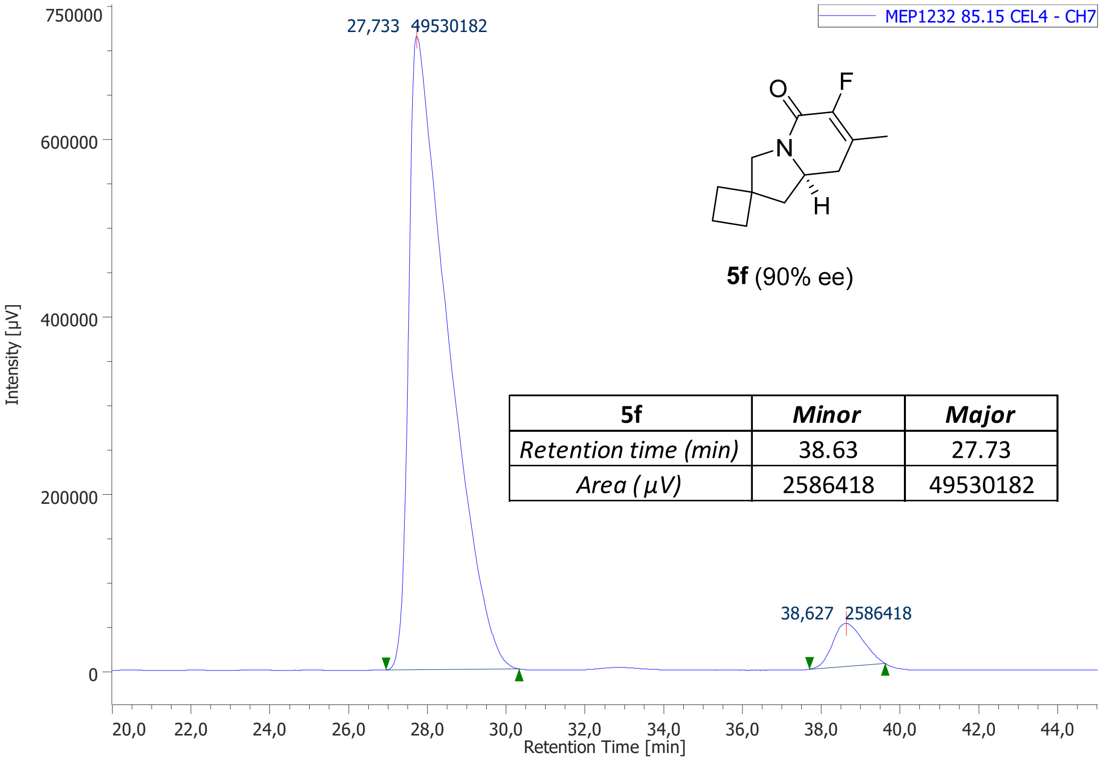

5f	*Minor*	*Major*
Retention time (min)	38.63	27.73
Area (µV)	2586418	49530182

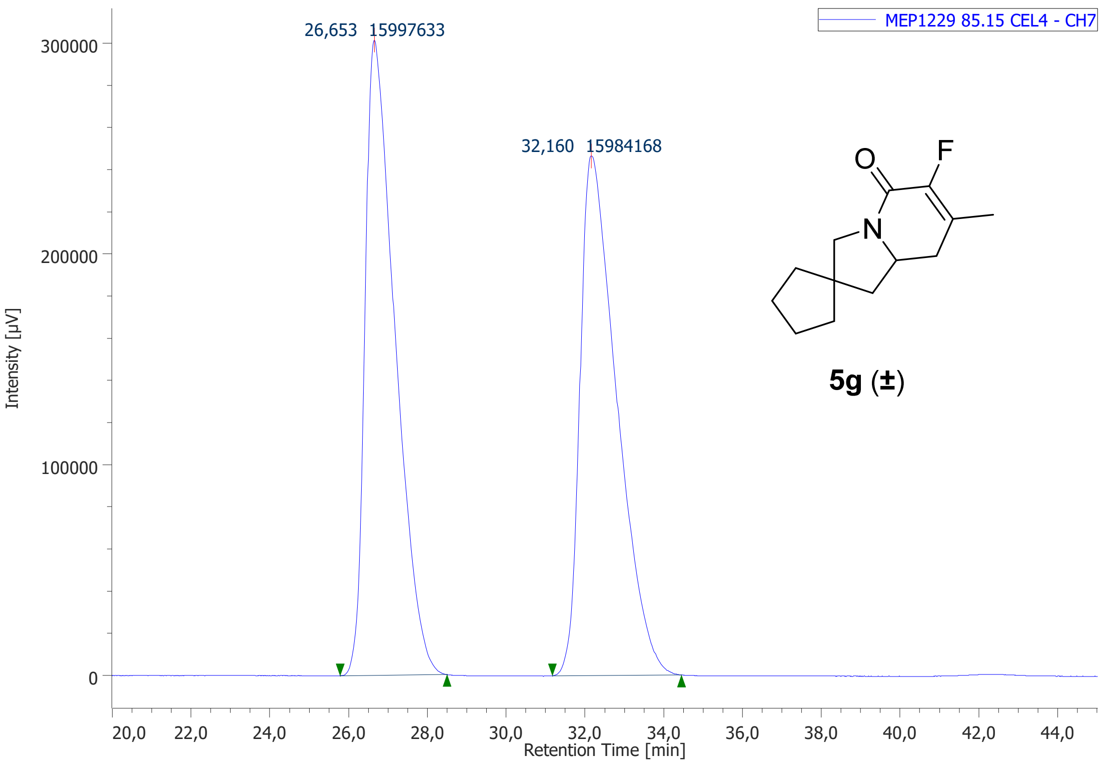

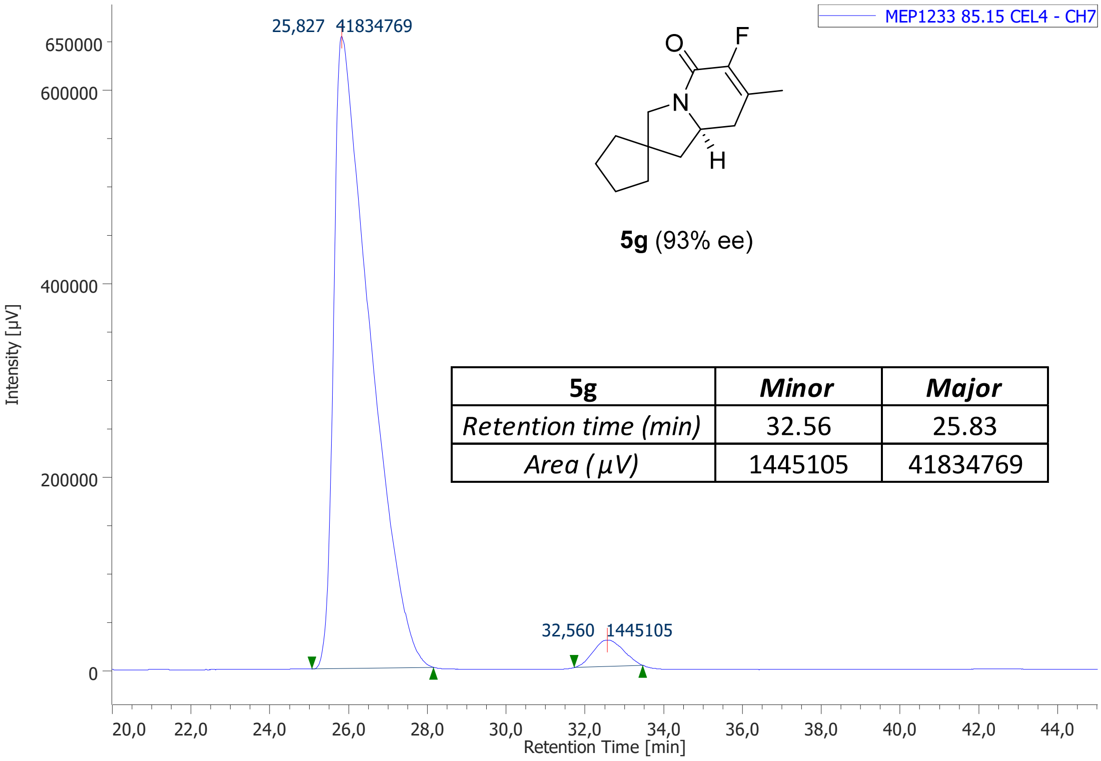

5g	*Minor*	*Major*
Retention time (min)	32.56	25.83
Area (µV)	1445105	41834769

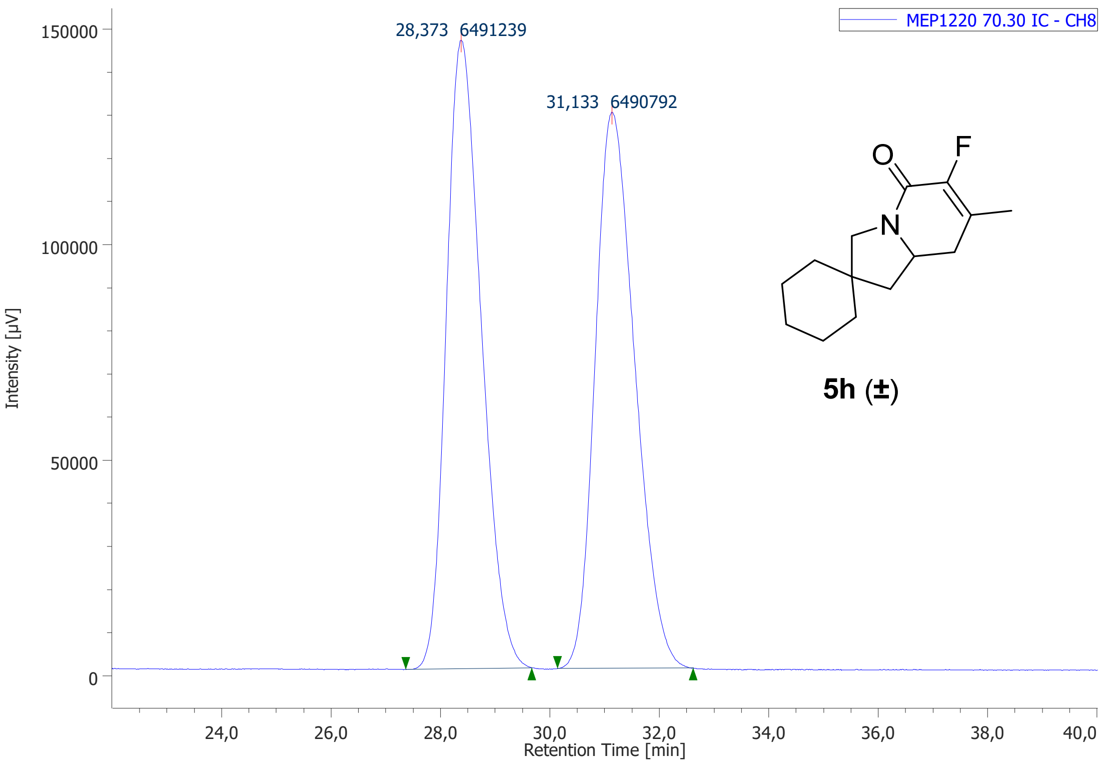

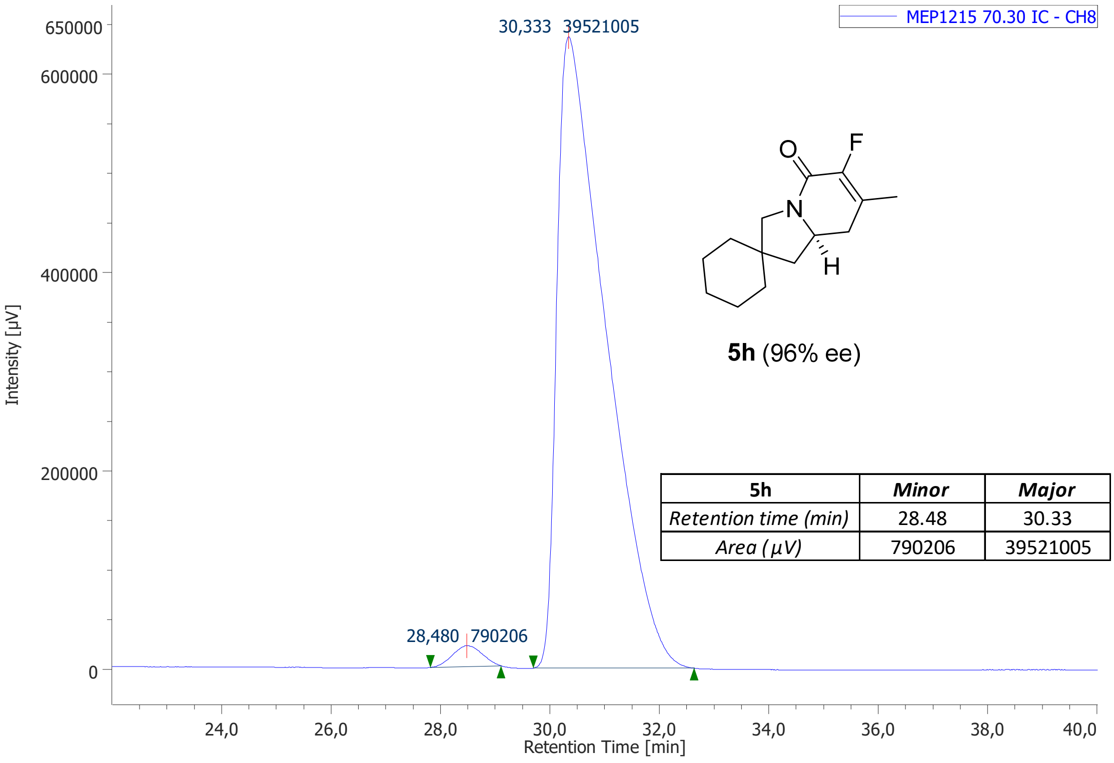

5h	*Minor*	*Major*
Retention time (min)	28.48	30.33
Area (µV)	790206	39521005

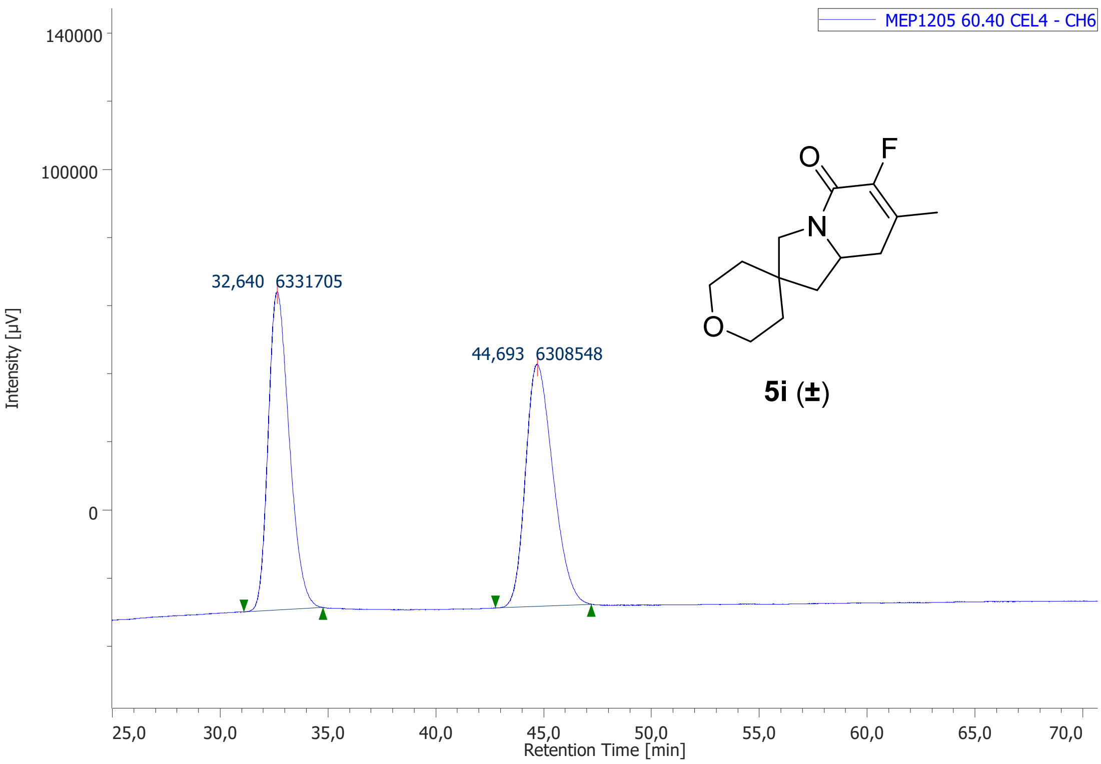

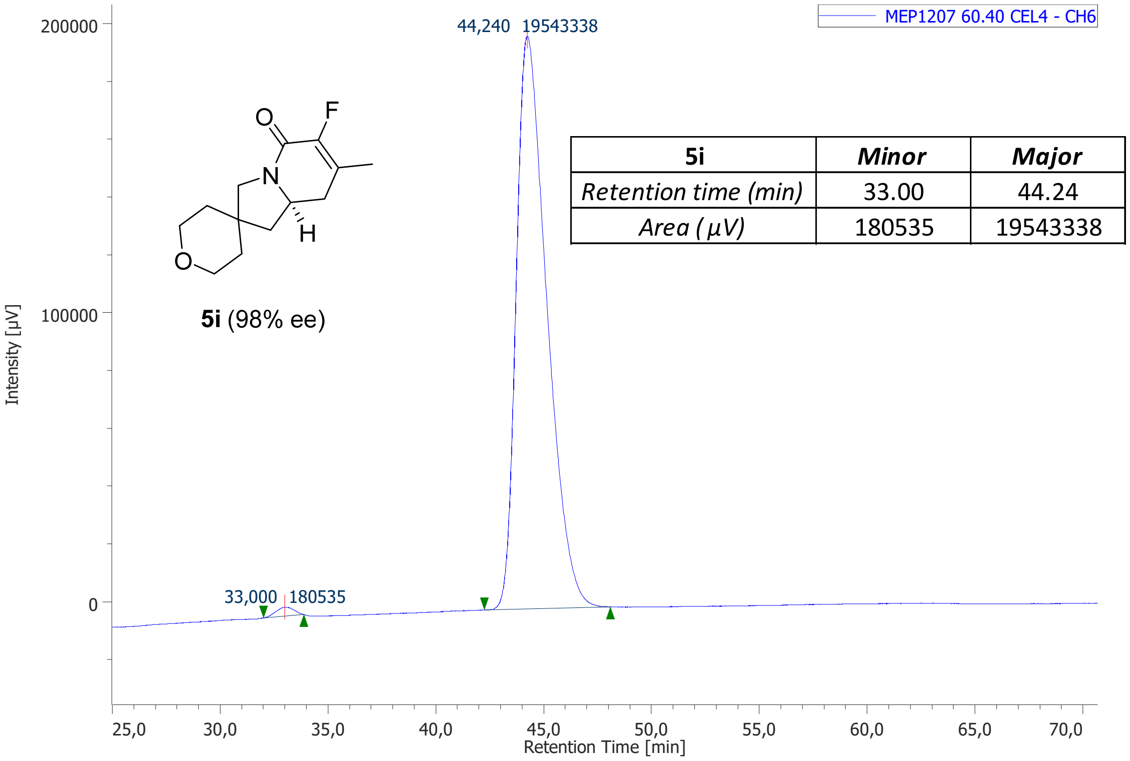

5i	*Minor*	*Major*
Retention time (min)	33.00	44.24
Area (µV)	180535	19543338

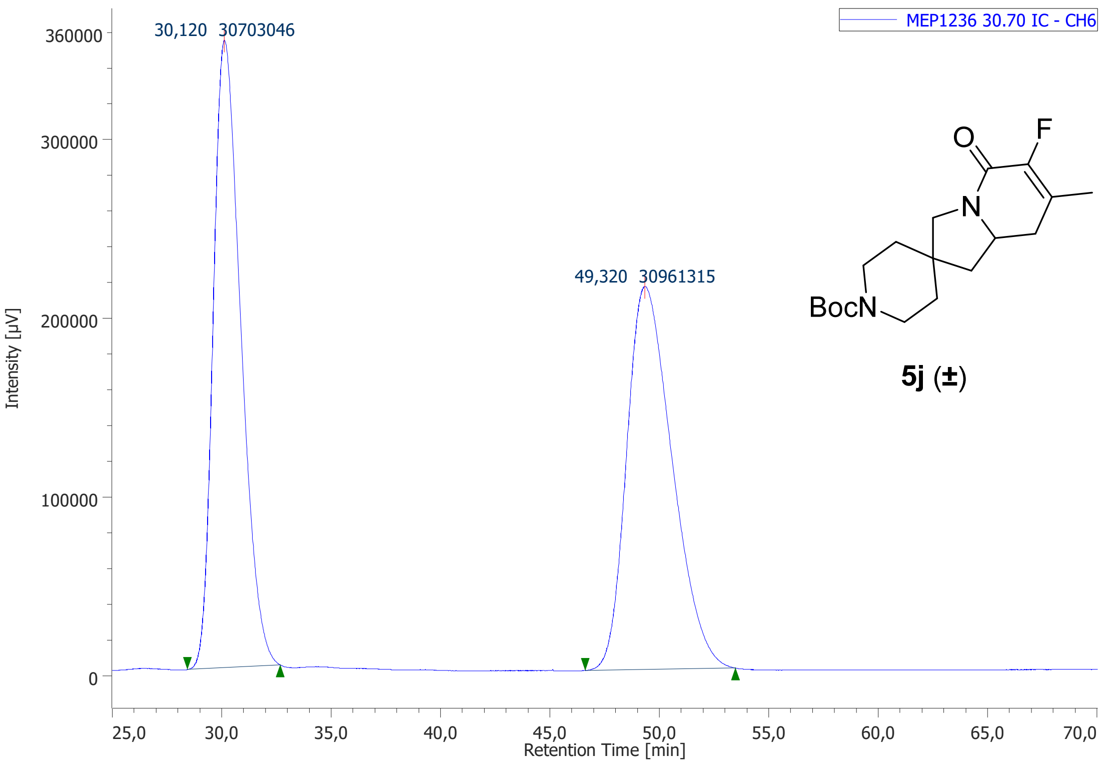

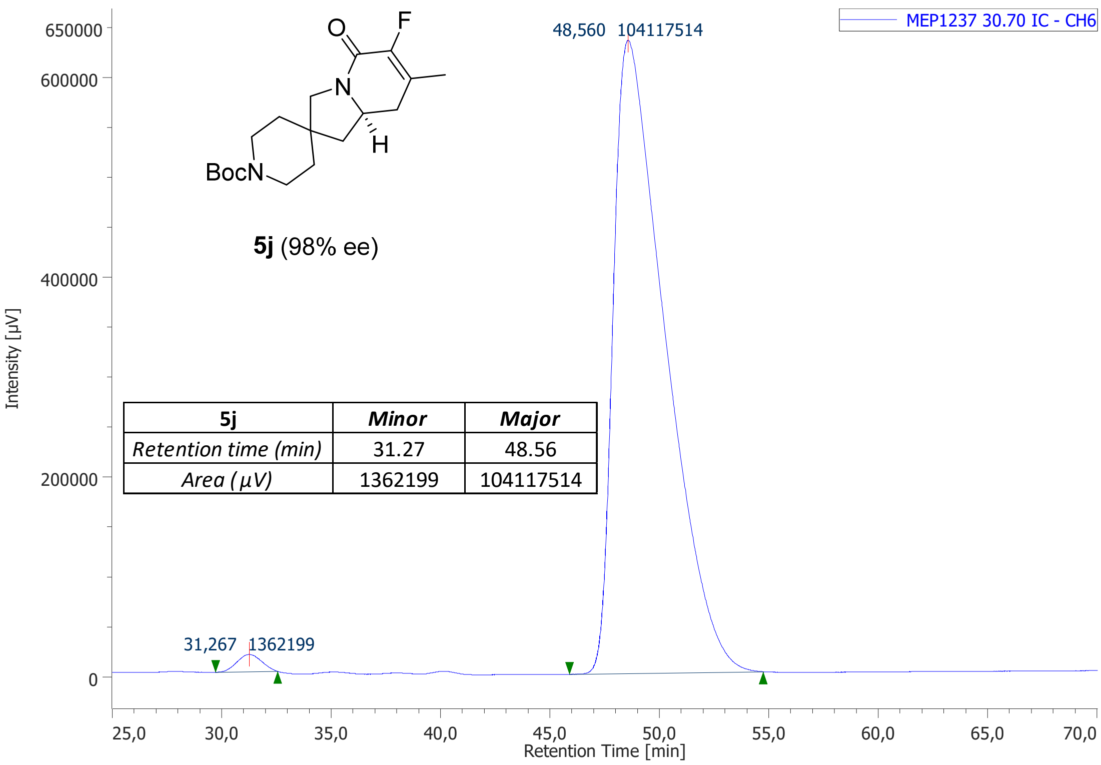

5j	*Minor*	*Major*
Retention time (min)	31.27	48.56
Area (µV)	1362199	104117514

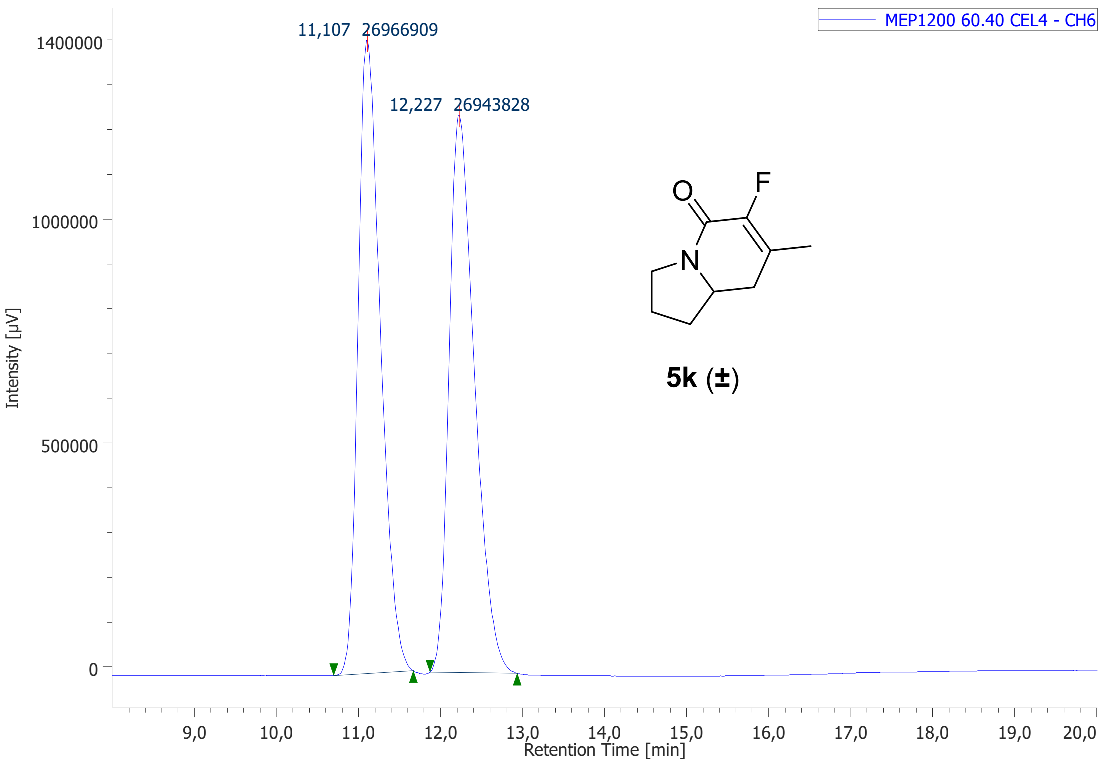

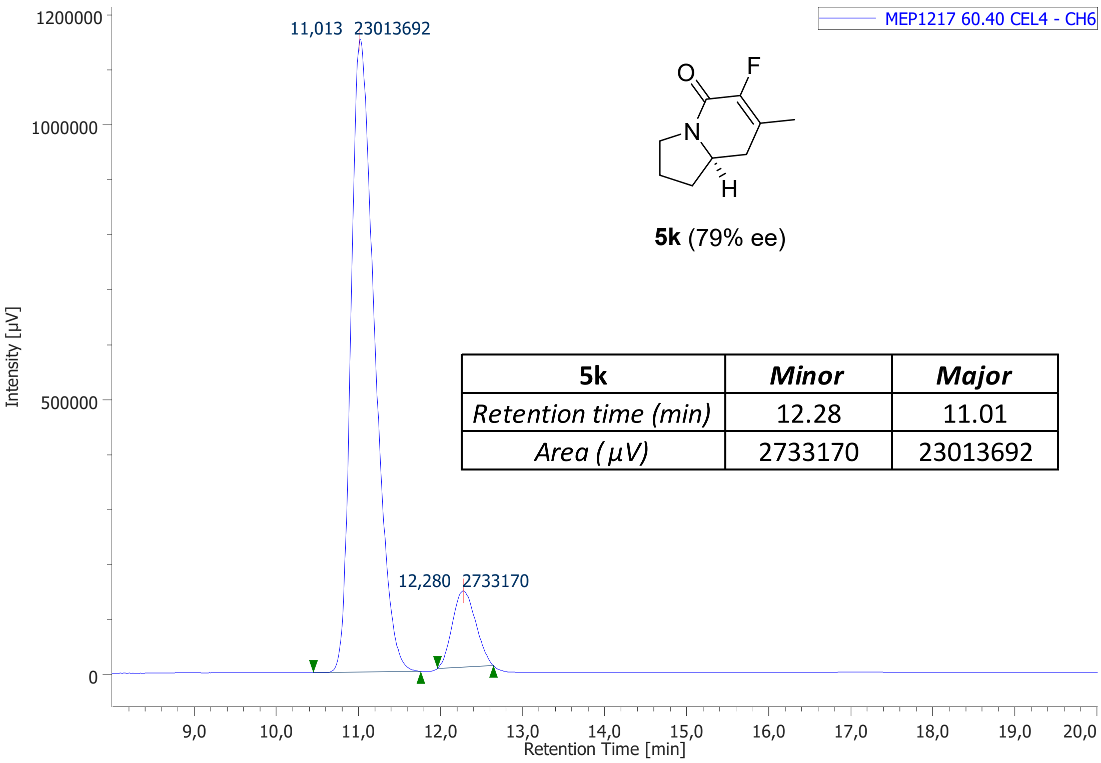

5k	*Minor*	*Major*
Retention time (min)	12.28	11.01
Area (µV)	2733170	23013692

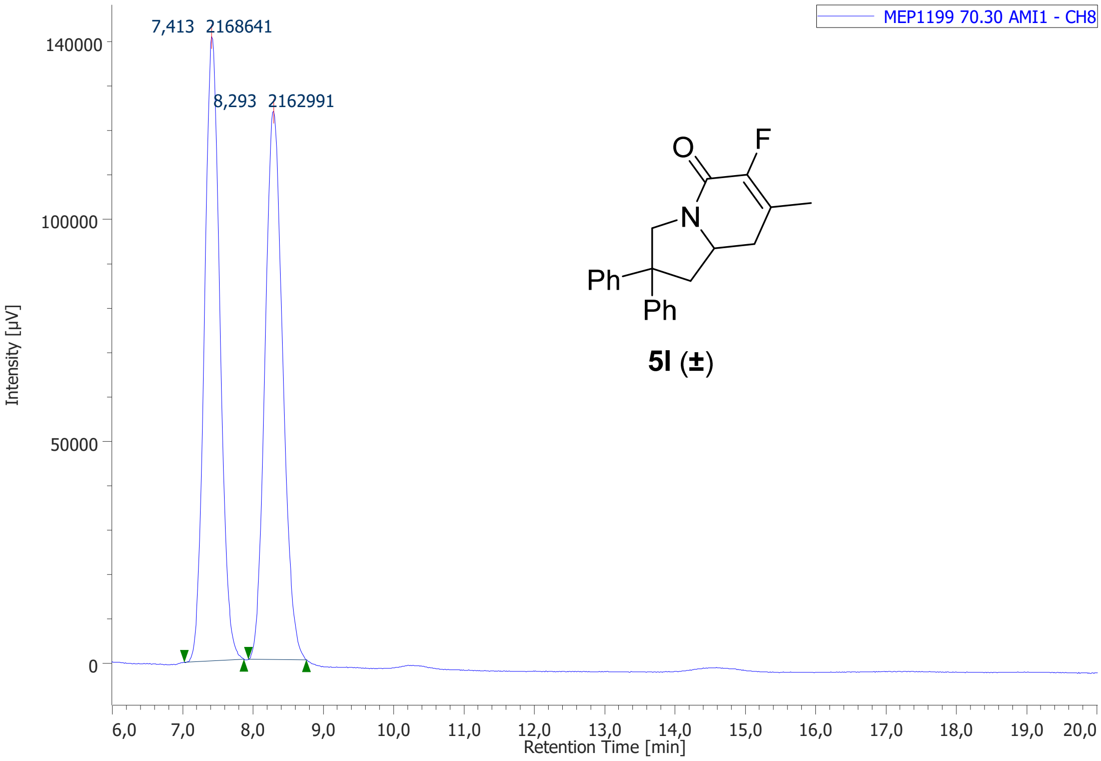

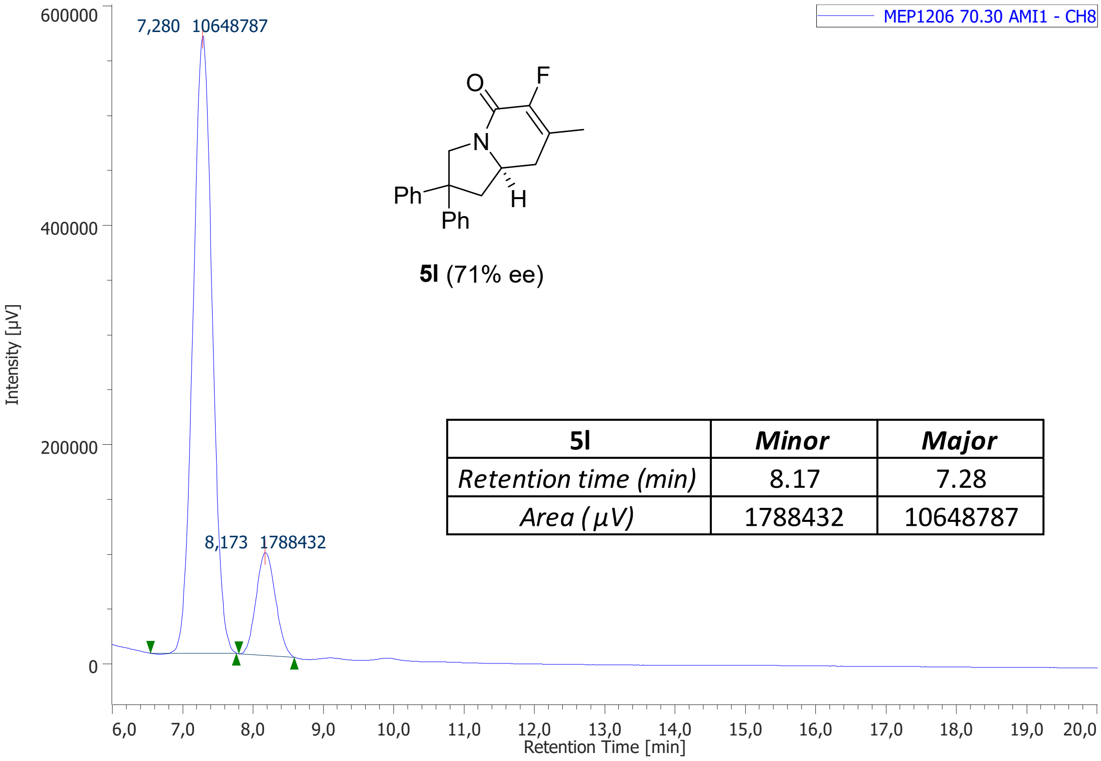

5l	*Minor*	*Major*
Retention time (min)	8.17	7.28
Area (µV)	1788432	10648787

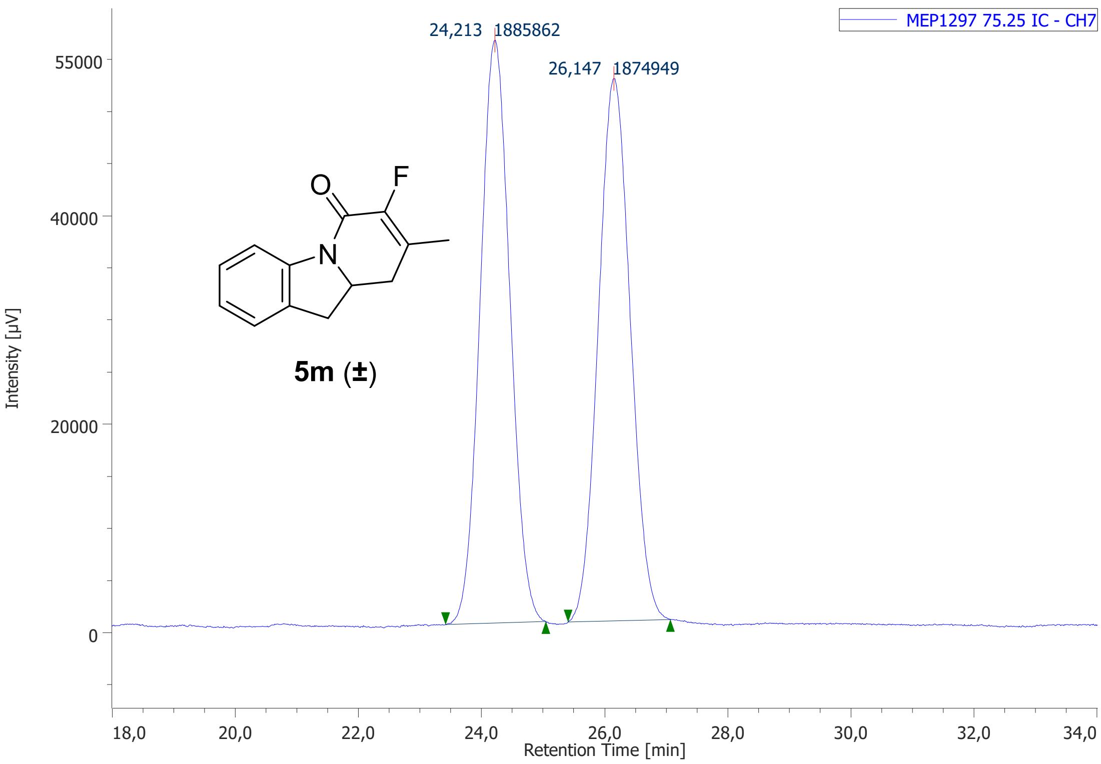

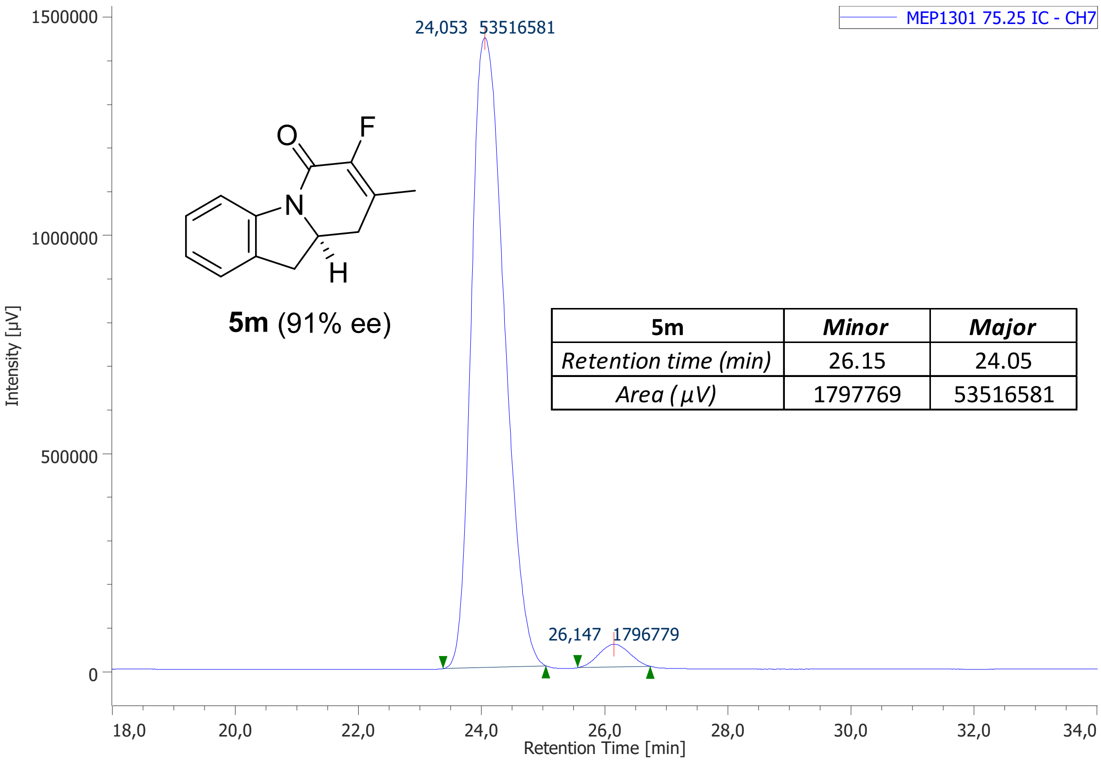

**5m**	***Minor***	***Major***
Retention time (min)	26.15	24.05
Area (µV)	1797769	53516581

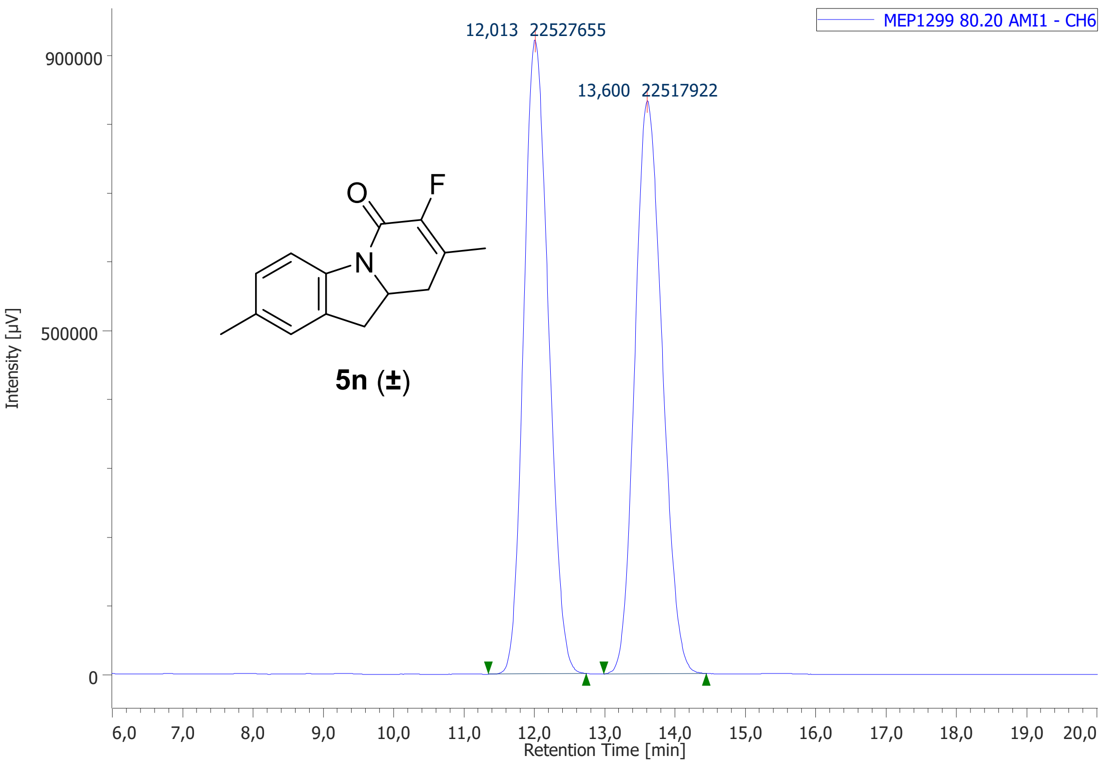

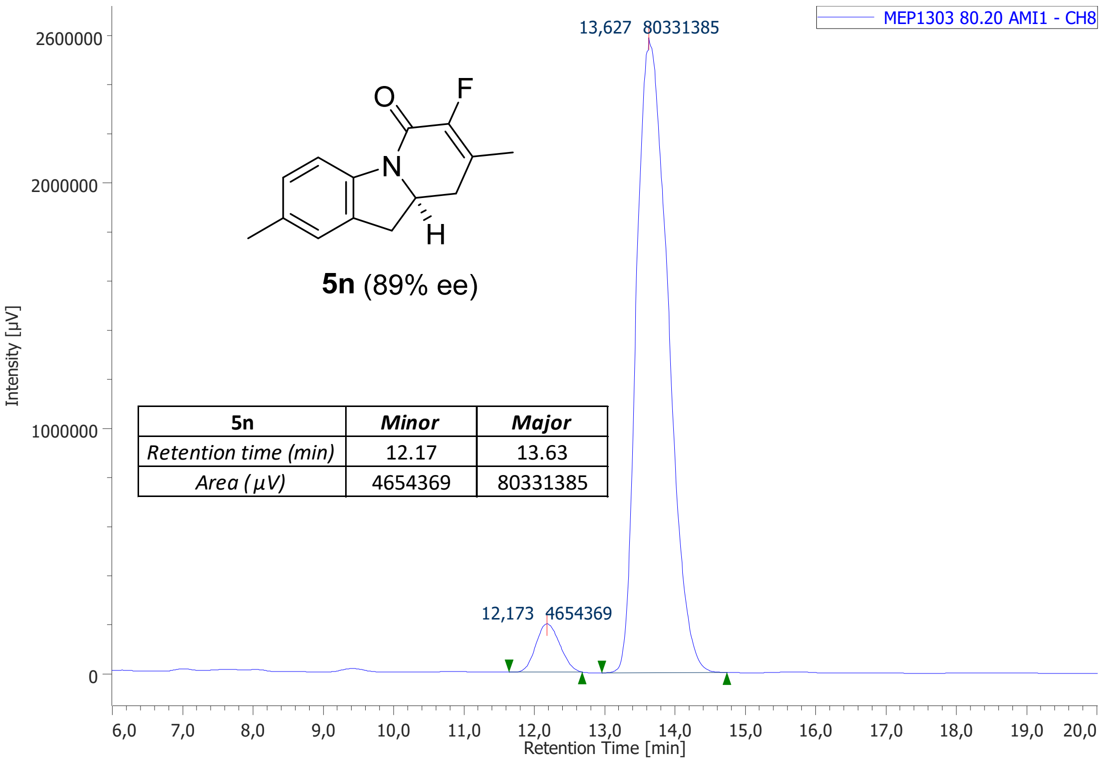

**5n**	***Minor***	***Major***
Retention time (min)	12.17	13.63
Area (µV)	4654369	80331385

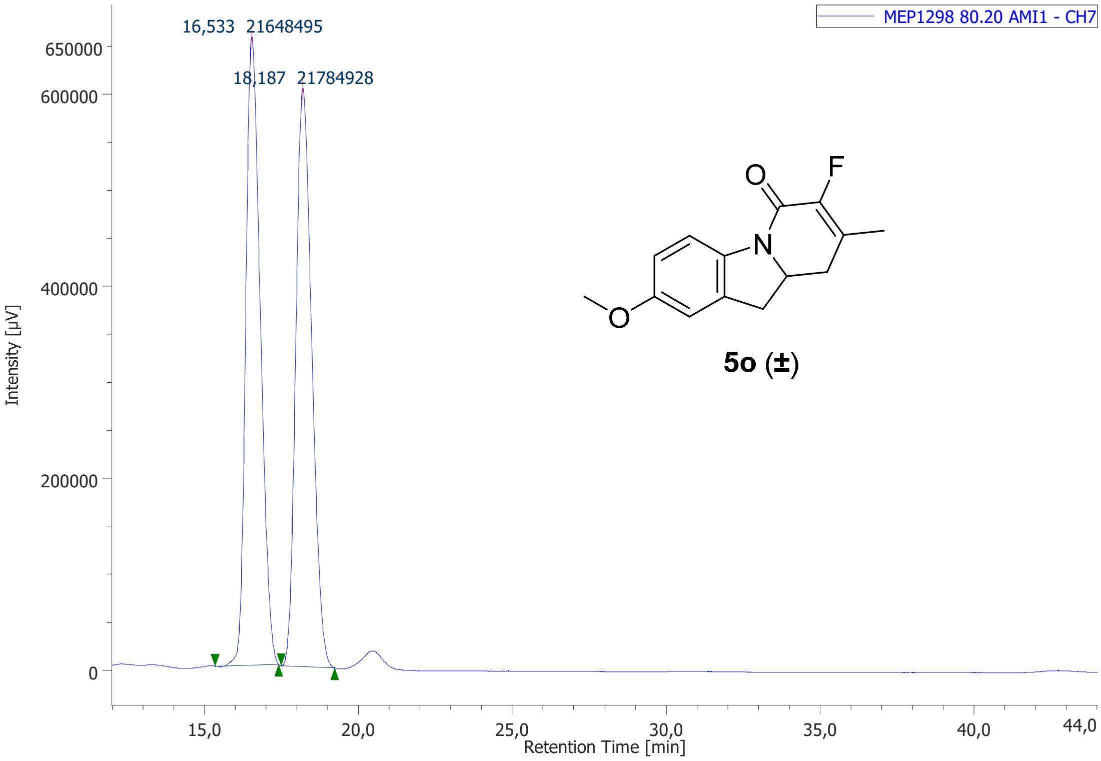

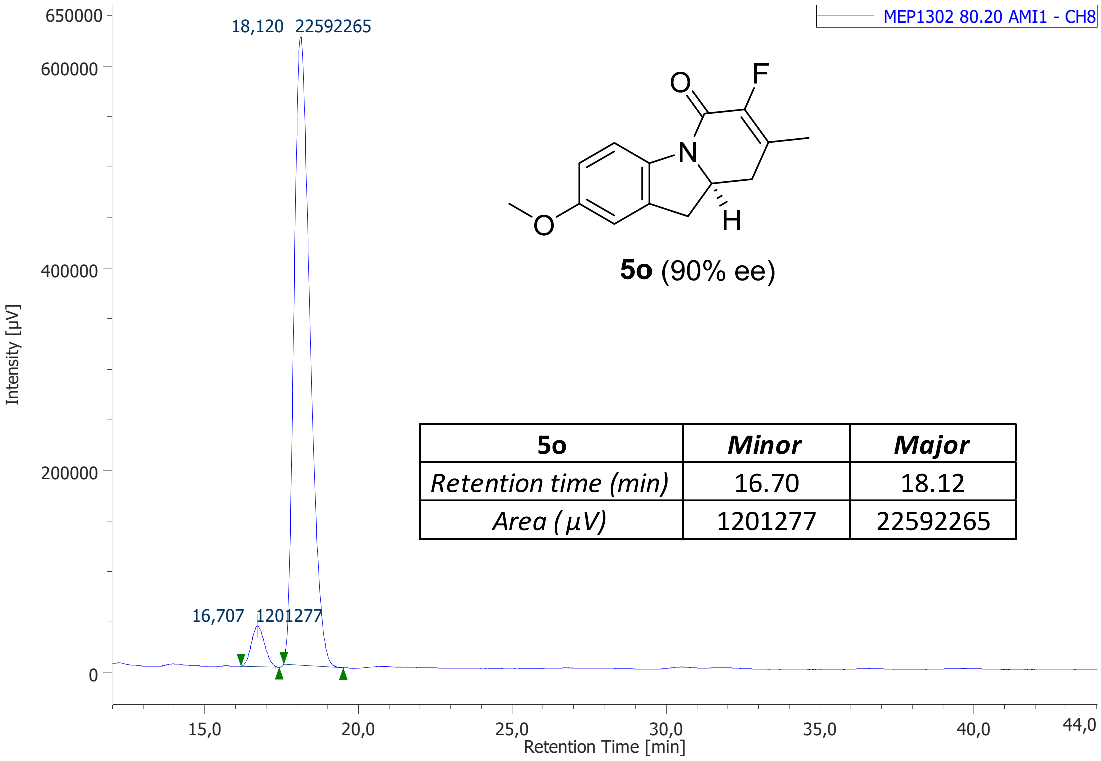

5o	*Minor*	*Major*
Retention time (min)	16.70	18.12
Area (µV)	1201277	22592265

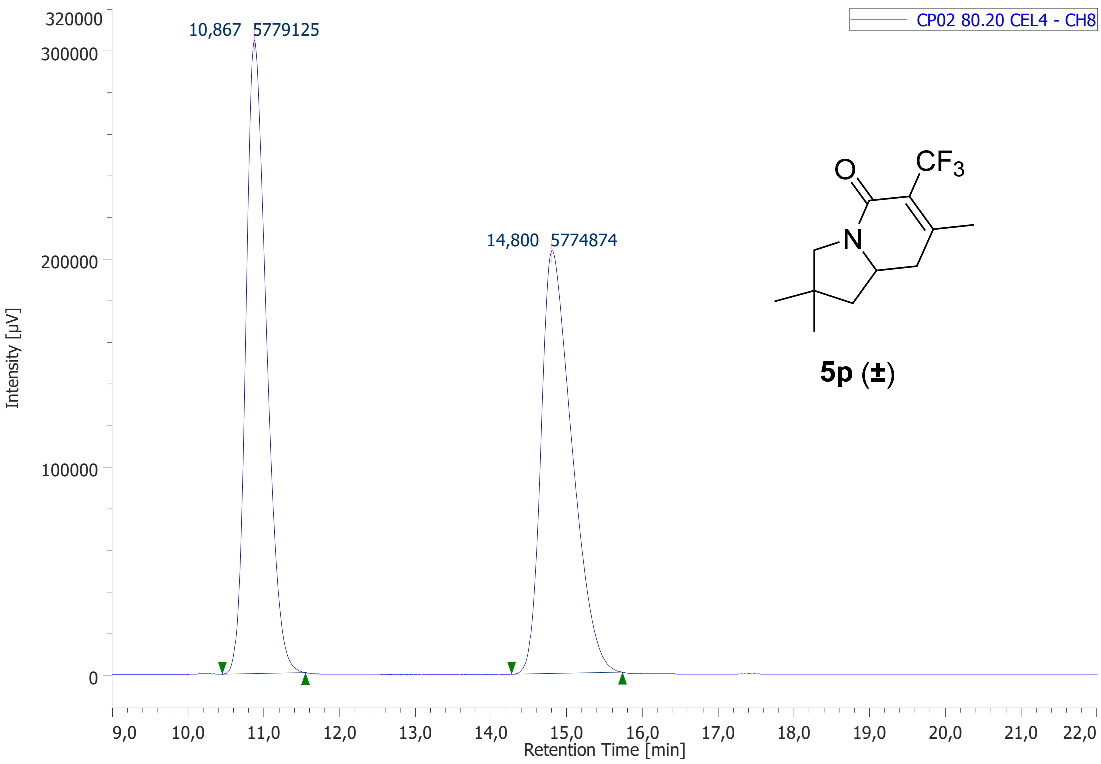

5p (±)

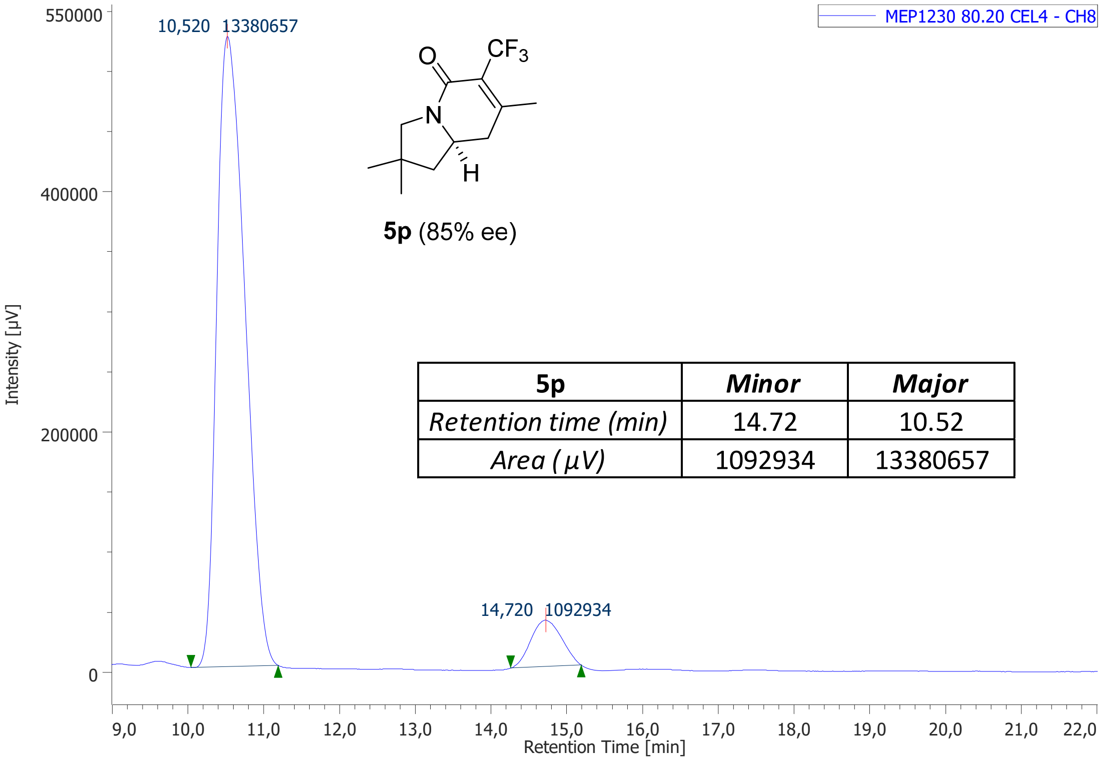

5p (85% ee)

**5p**	***Minor***	***Major***
Retention time (min)	14.72	10.52
Area (µV)	1092934	13380657

NMR SPECTRA OF NEW COMPOUNDS:

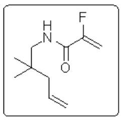

1a

^1H NMR (500 MHz, $CDCl_3$)

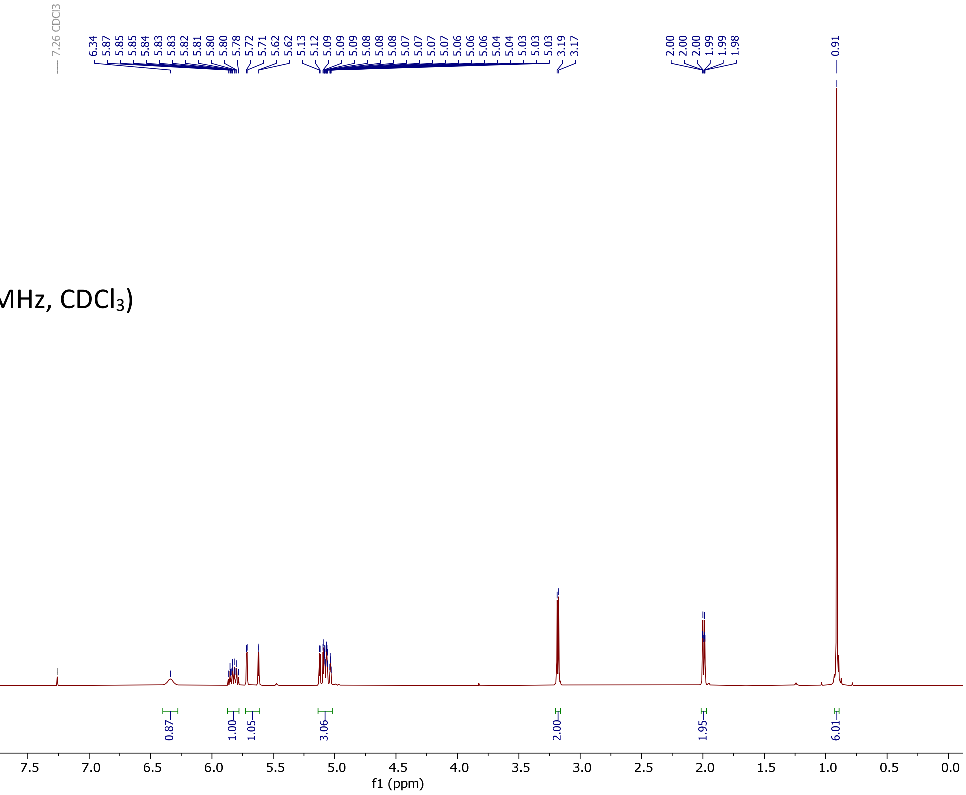

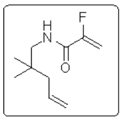

1a

^{19}F NMR (471 MHz, $CDCl_3$)

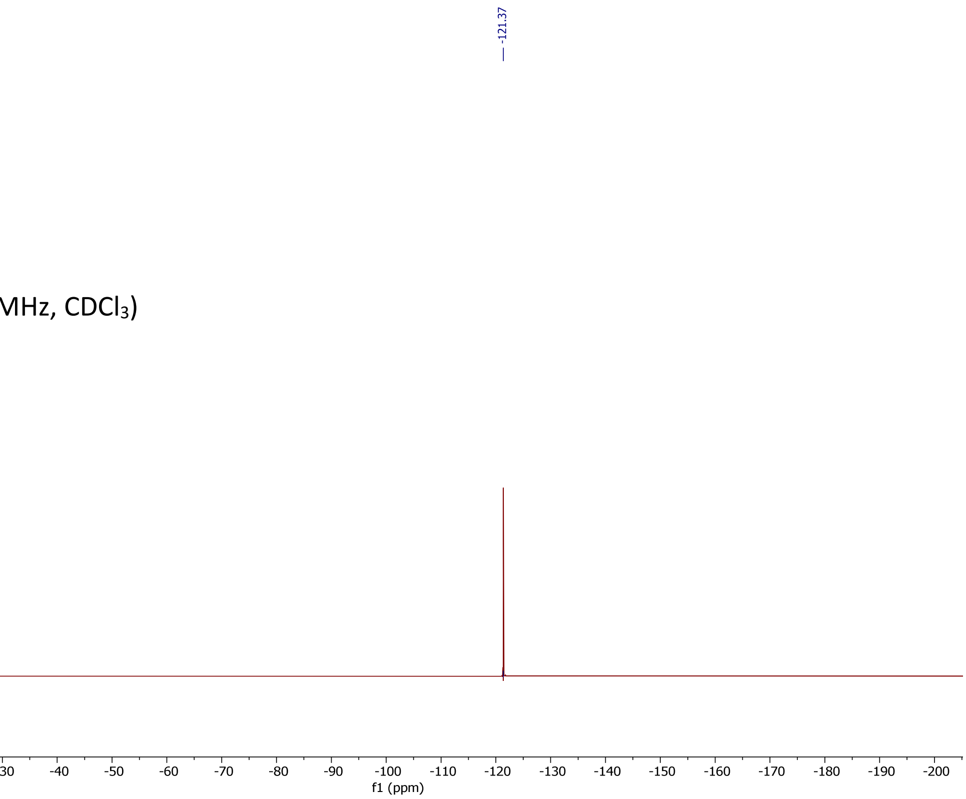

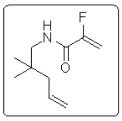

1a

^{13}C NMR (126 MHz, $CDCl_3$)

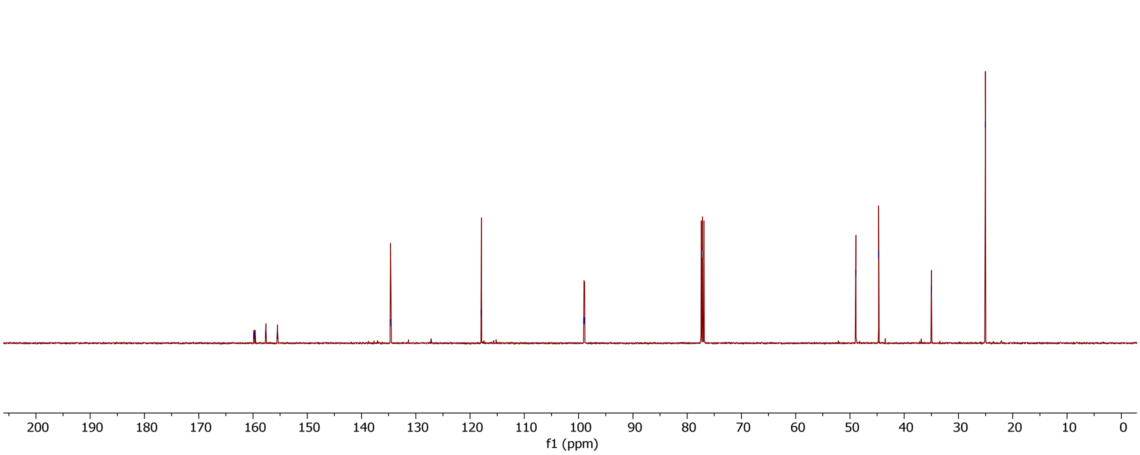

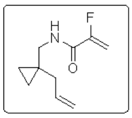

1b

^{1}H NMR (300 MHz, $CDCl_3$)

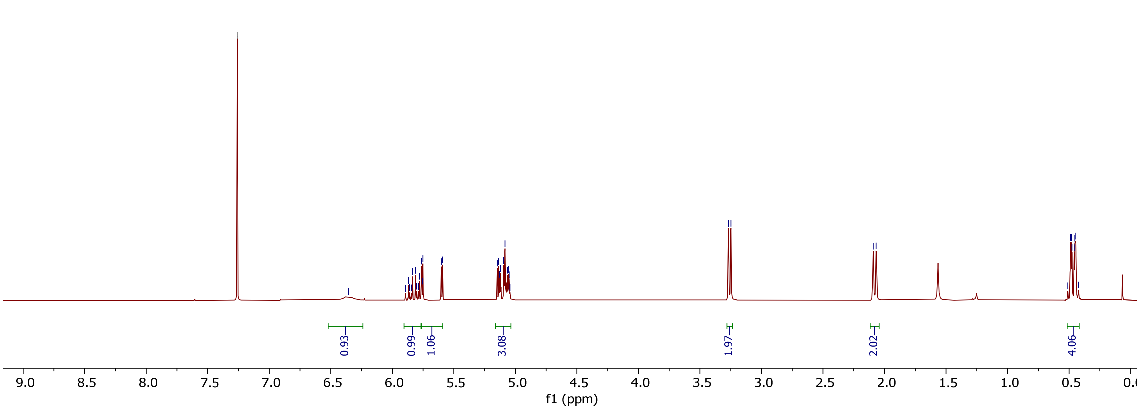

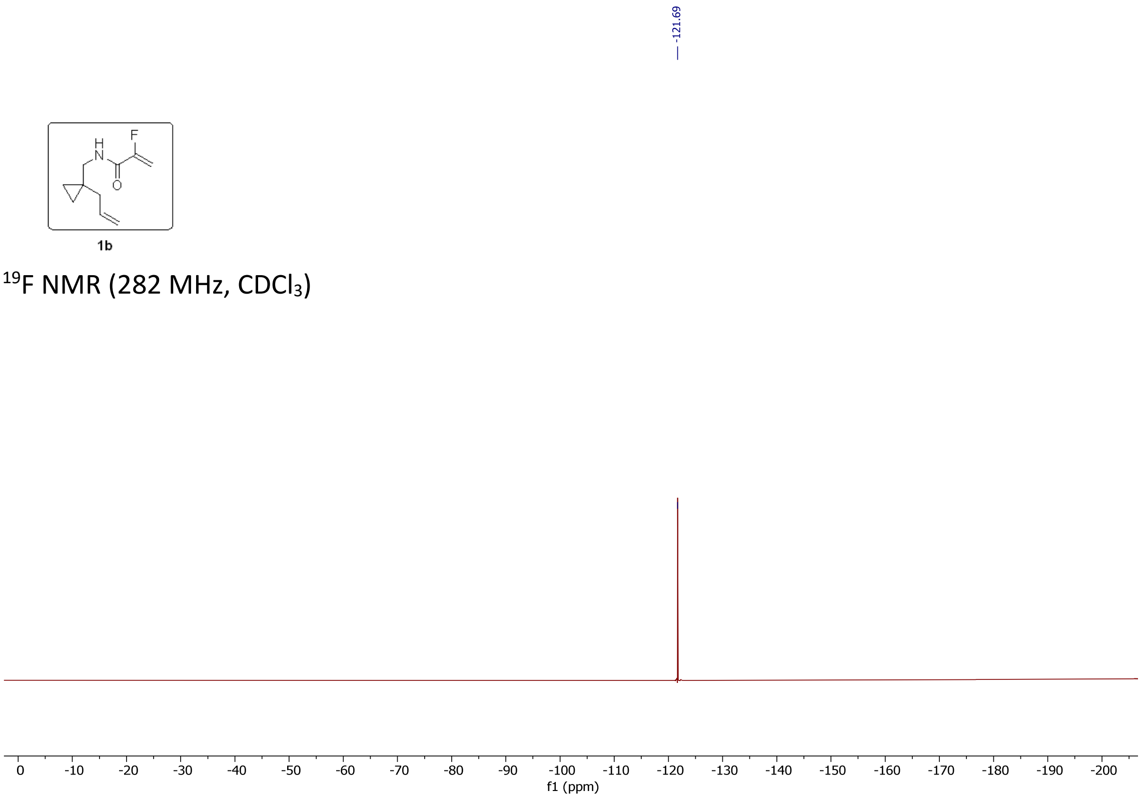

^{19}F NMR (282 MHz, $CDCl_3$)

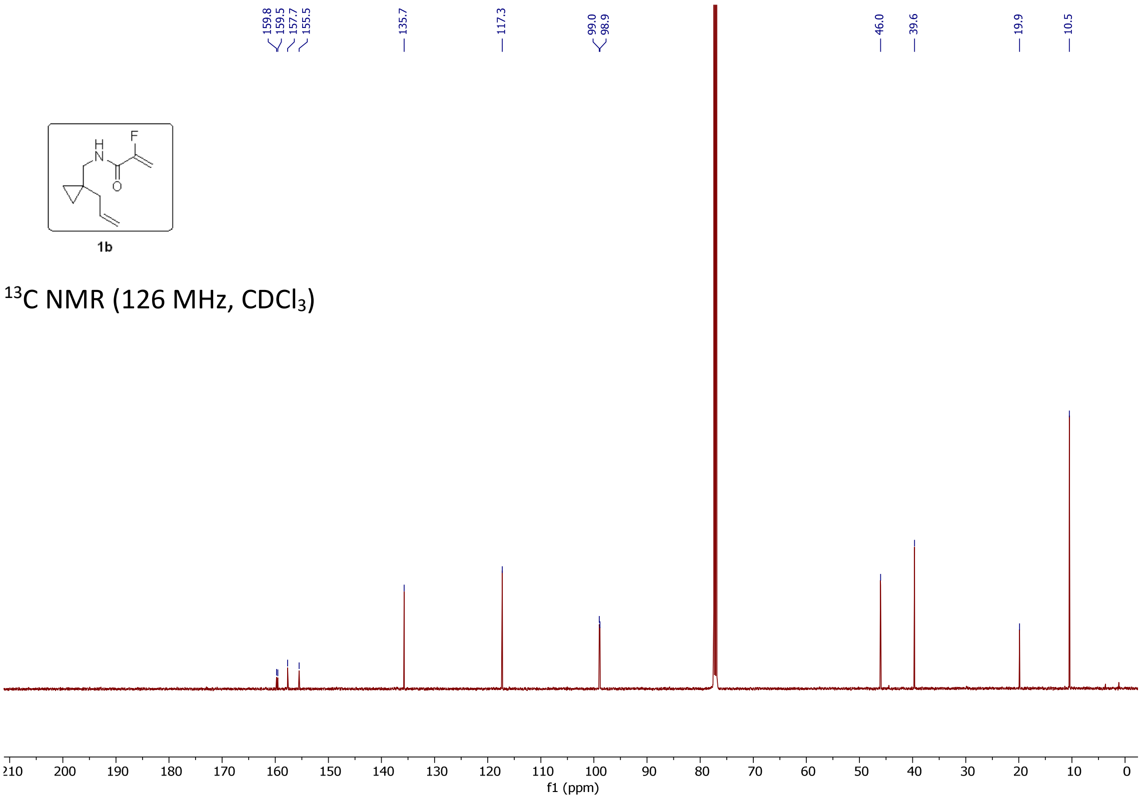

^{13}C NMR (126 MHz, $CDCl_3$)

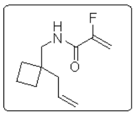

1c

^{1}H NMR (300 MHz, CDCl$_3$)

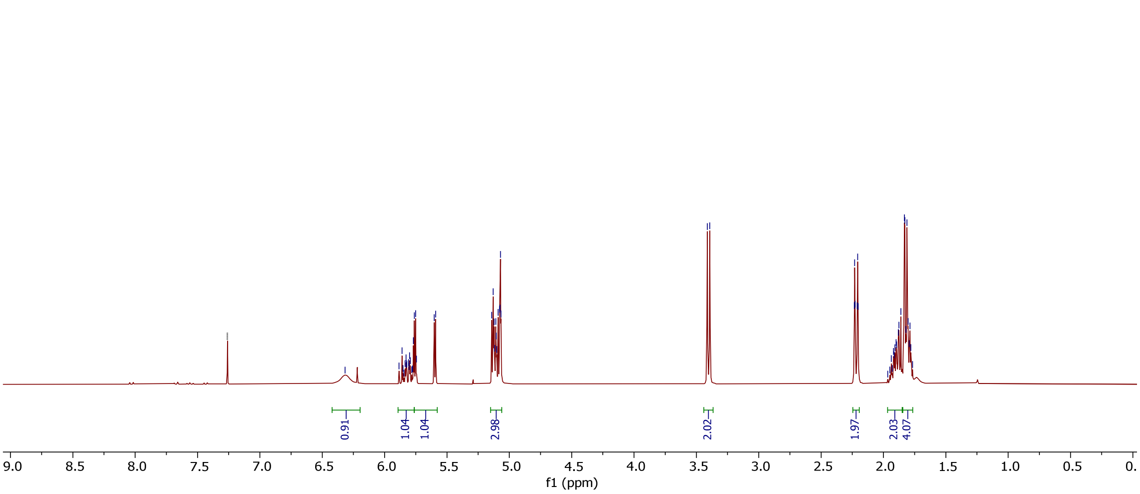

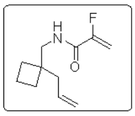

1c

^{19}F NMR (282 MHz, CDCl$_3$)

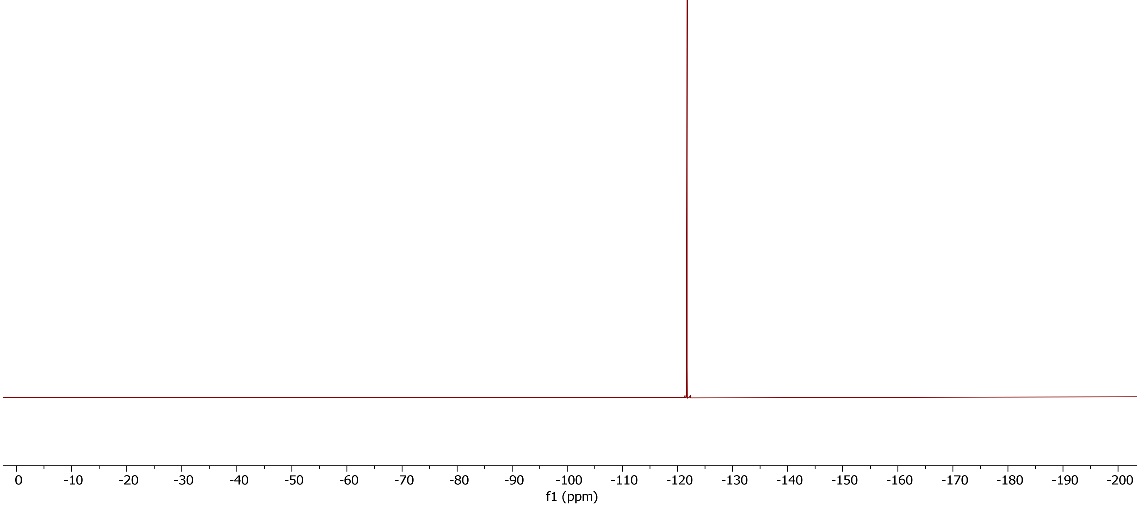

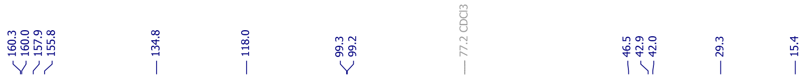

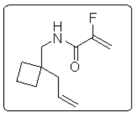

1c

^{13}C NMR (126 MHz, $CDCl_3$)

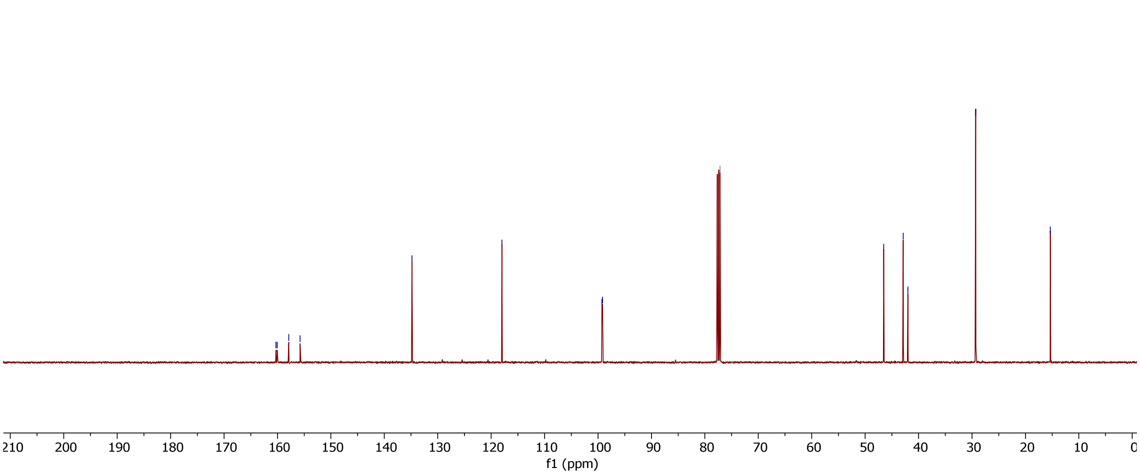

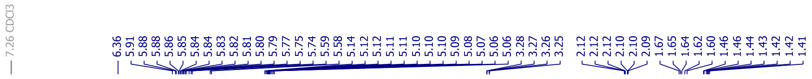

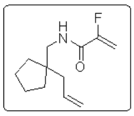

1d

^{1}H NMR (300 MHz, $CDCl_3$)

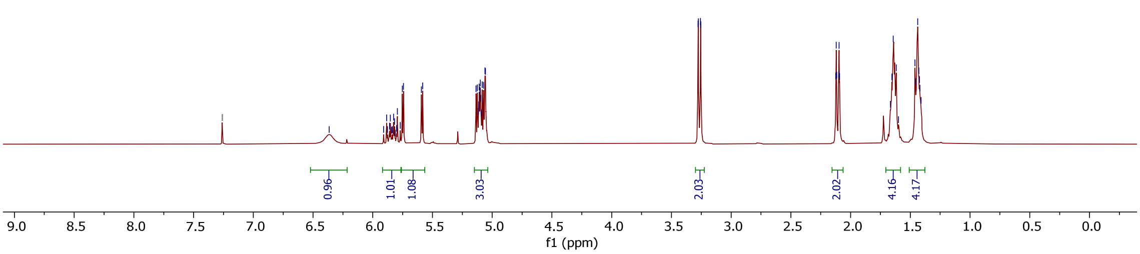

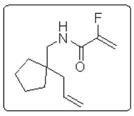

1d

^{19}F NMR (282 MHz, $CDCl_3$)

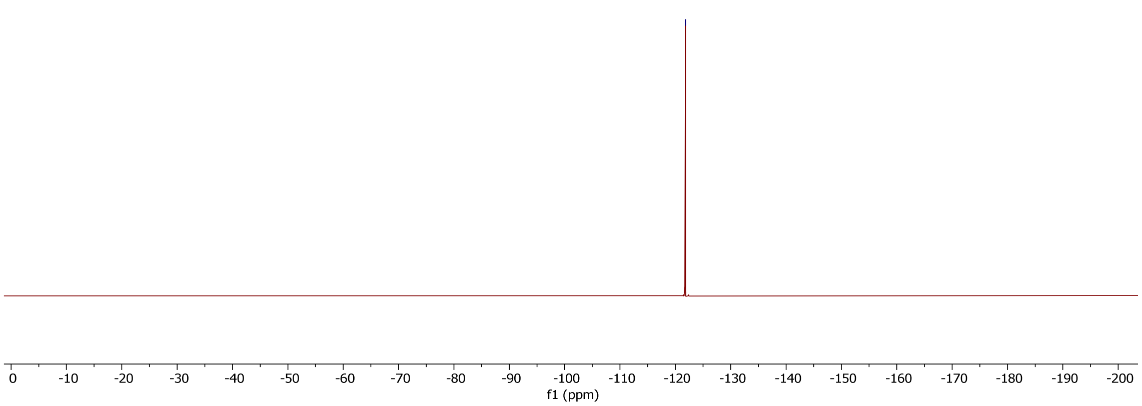

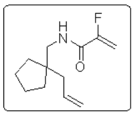

1d

^{13}C NMR (126 MHz, $CDCl_3$)

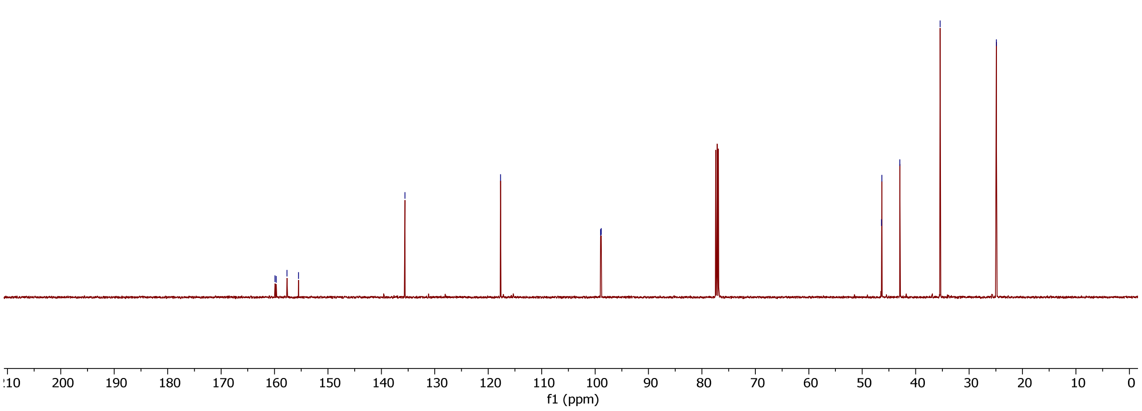

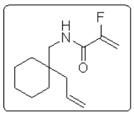

^1H NMR (300 MHz, $CDCl_3$)

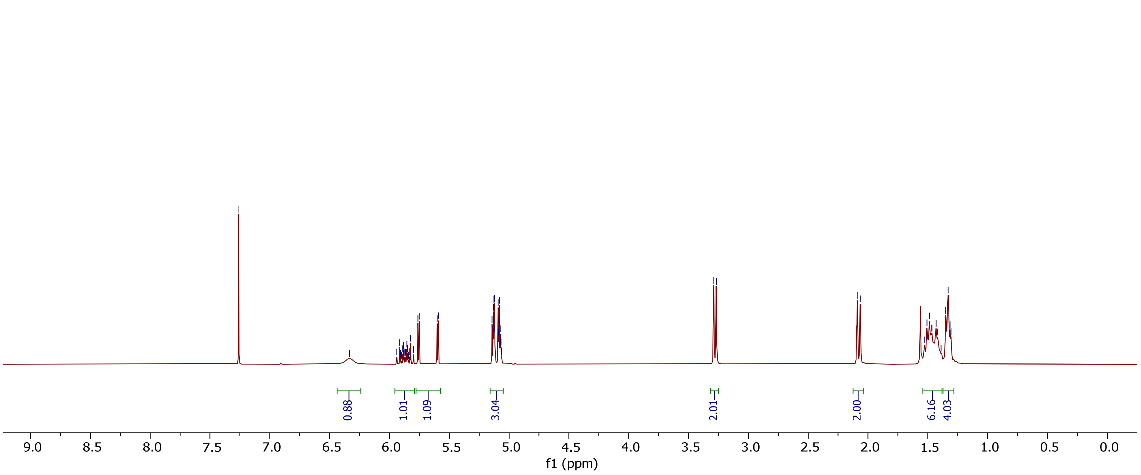

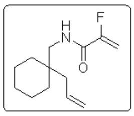

^{19}F NMR (282 MHz, $CDCl_3$)

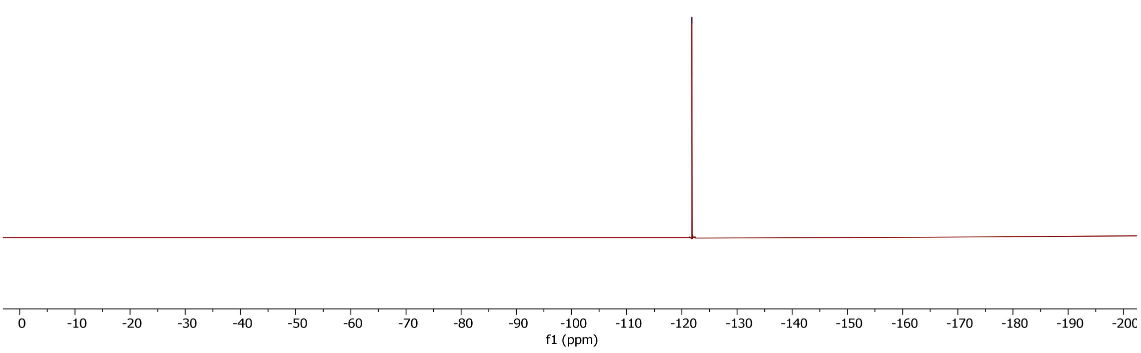

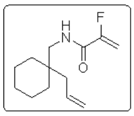

1e

^{13}C NMR (75 MHz, $CDCl_3$)

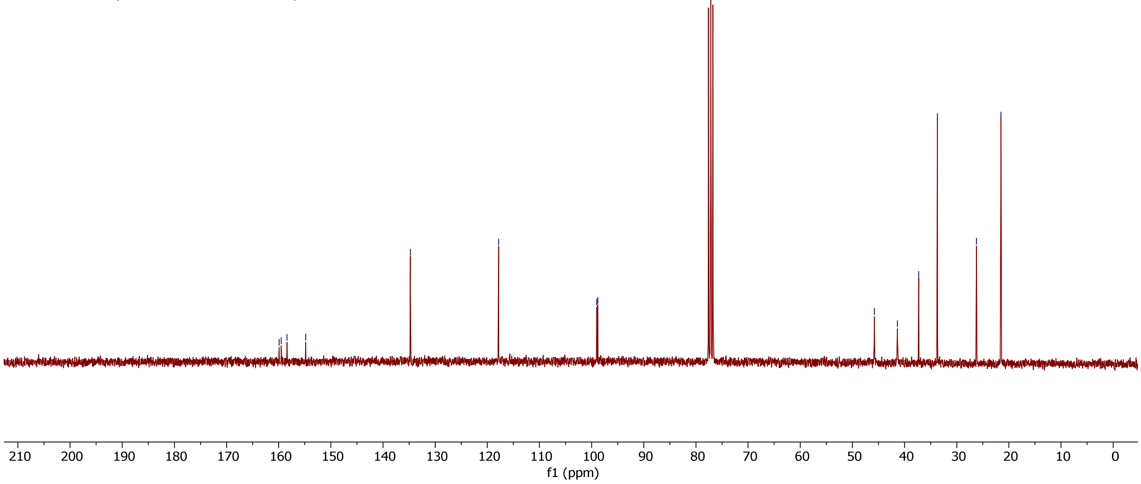

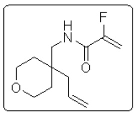

1f

^{1}H NMR (300 MHz, $CDCl_3$)

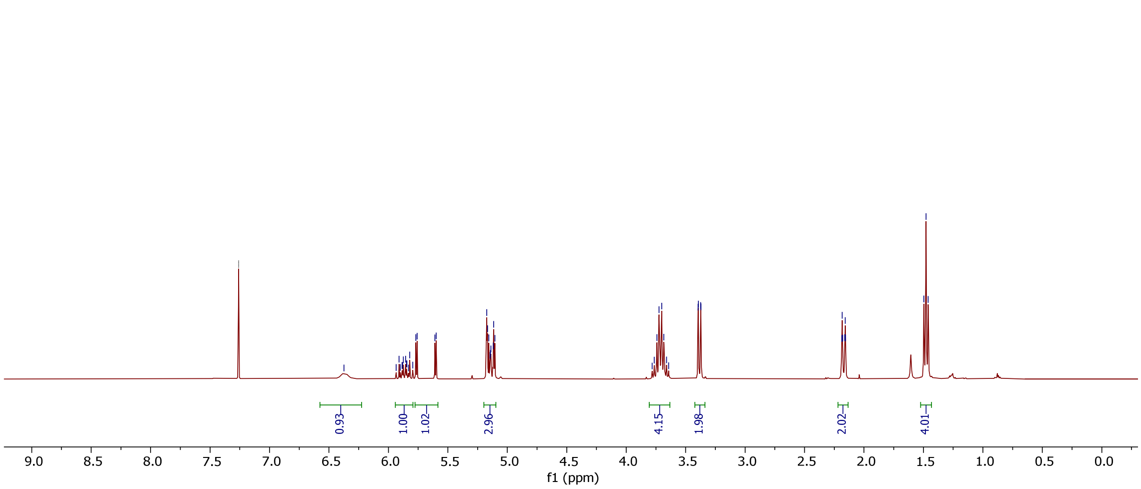

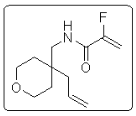

1f

^{19}F NMR (282 MHz, $CDCl_3$)

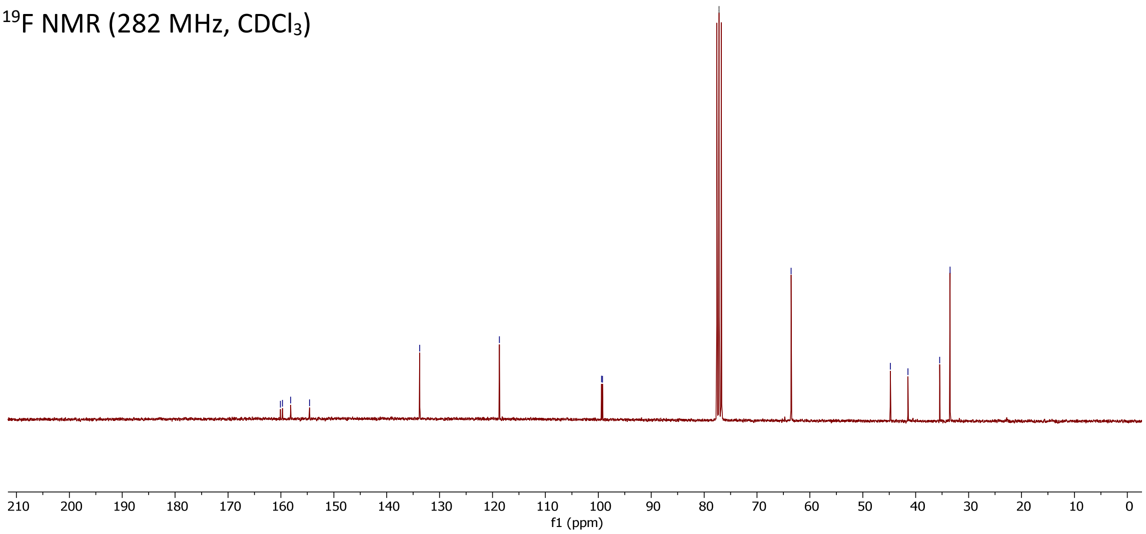

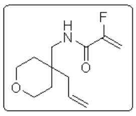

1f

^{13}C NMR (75 MHz, $CDCl_3$)

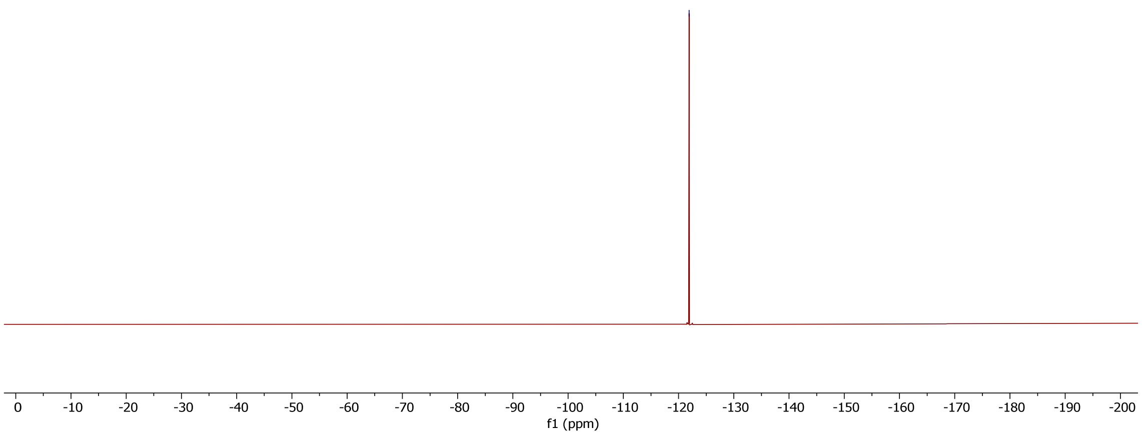

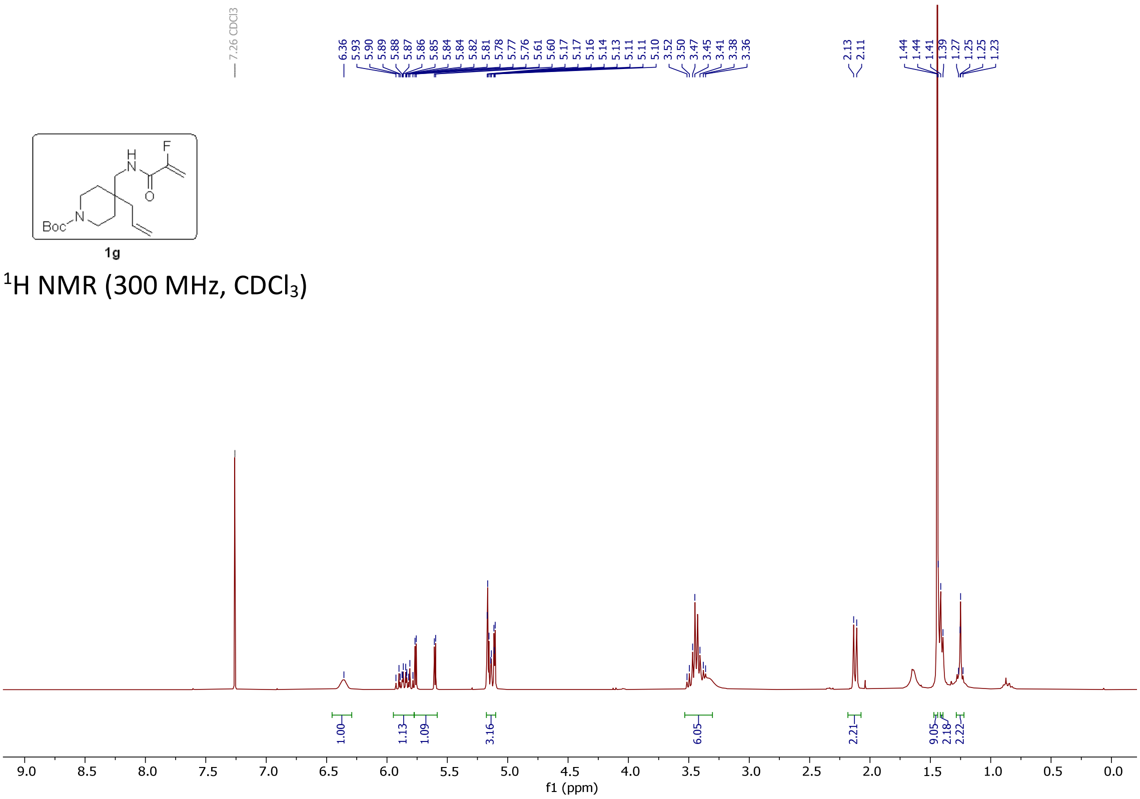

^{1}H NMR (300 MHz, $CDCl_3$)

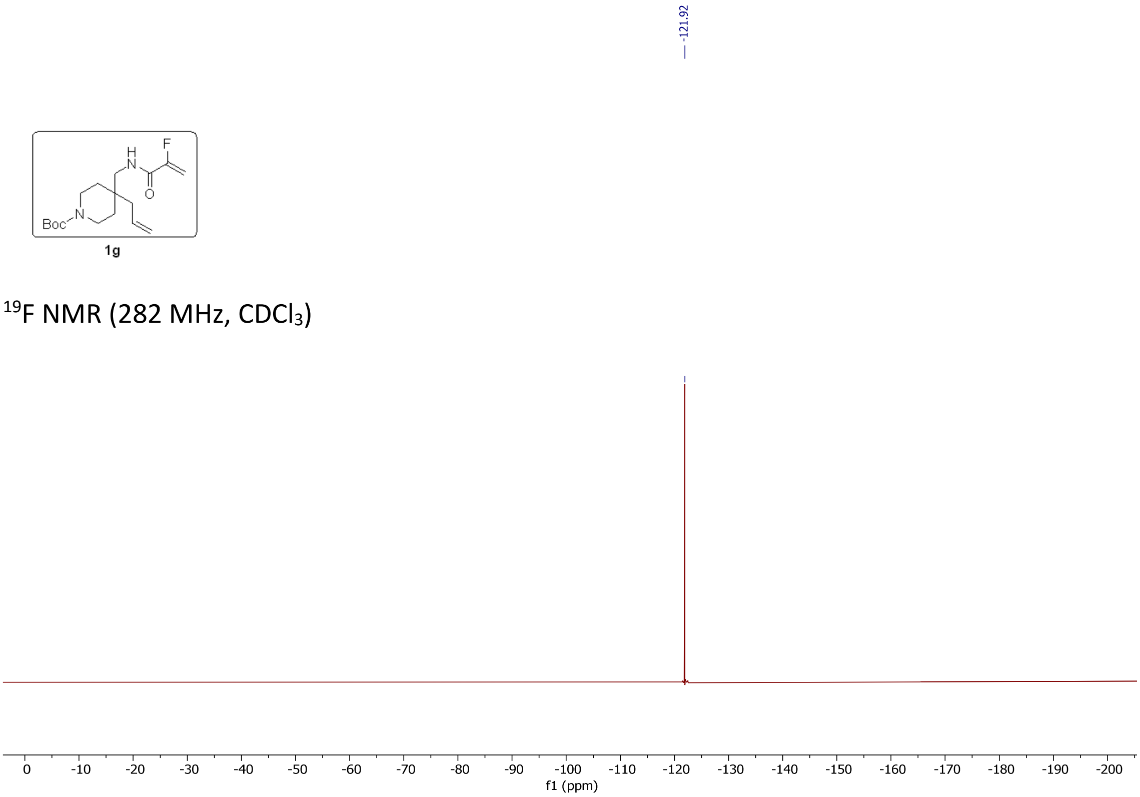

^{19}F NMR (282 MHz, $CDCl_3$)

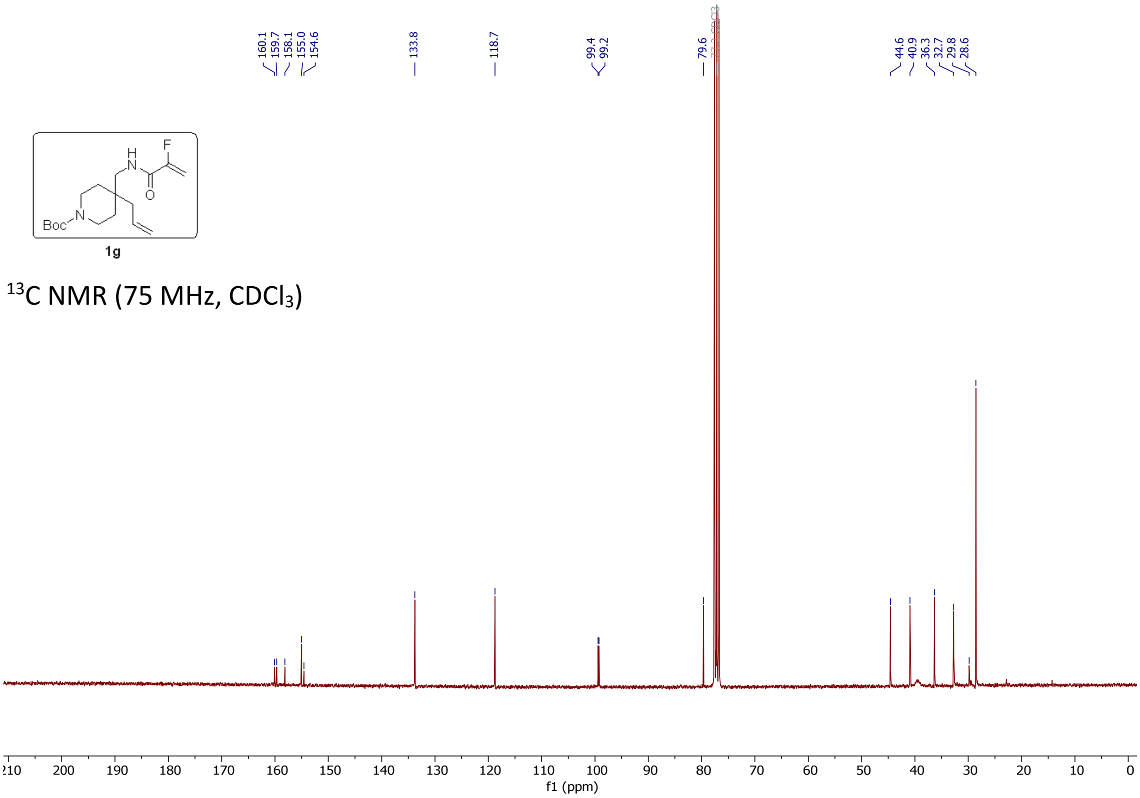

1g

^{13}C NMR (75 MHz, $CDCl_3$)

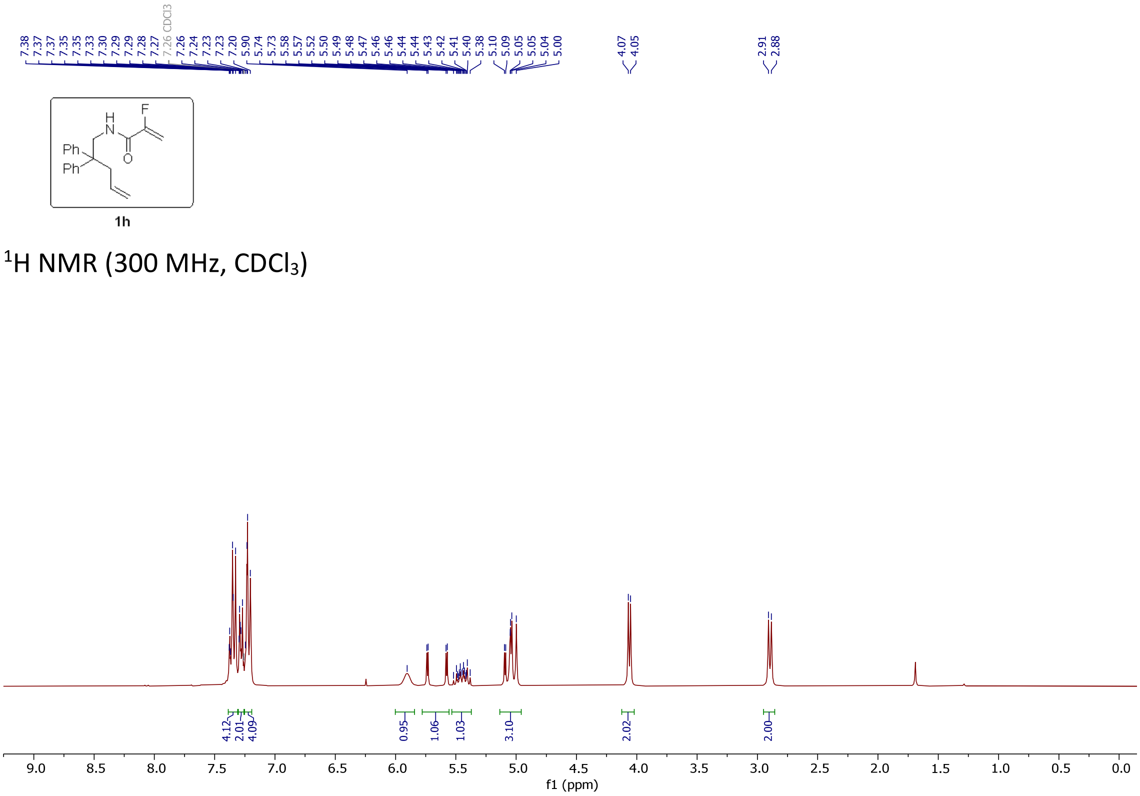

1h

^{1}H NMR (300 MHz, $CDCl_3$)

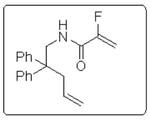

1h

^{19}F NMR (282 MHz, $CDCl_3$)

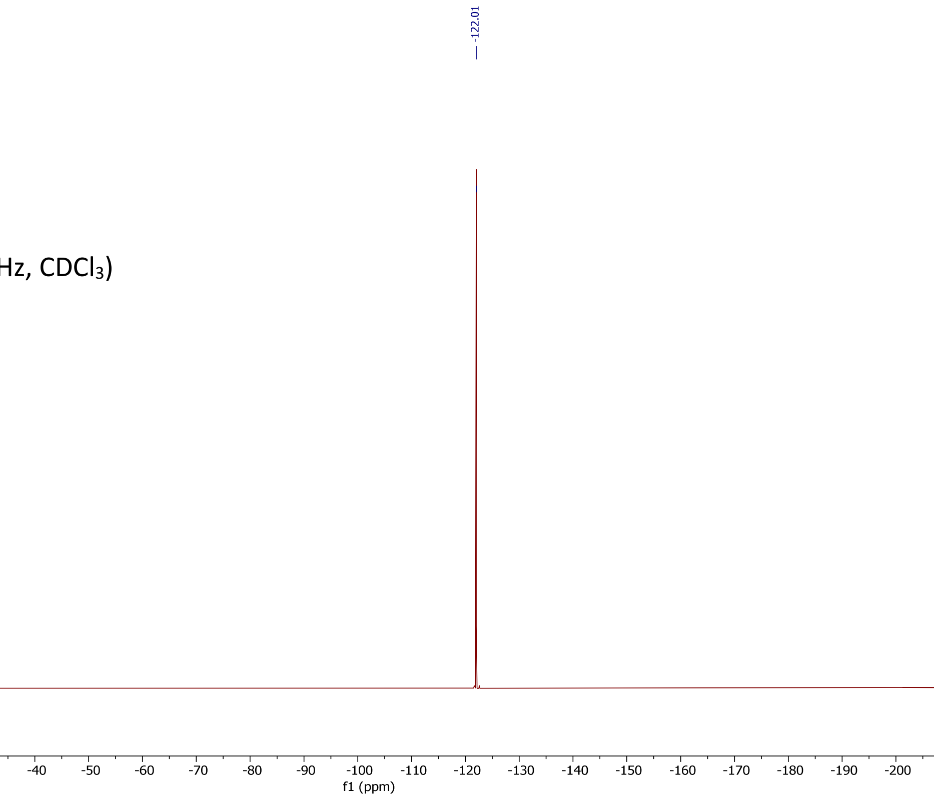

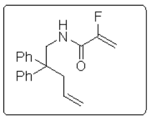

1h

^{13}C NMR (75 MHz, $CDCl_3$)

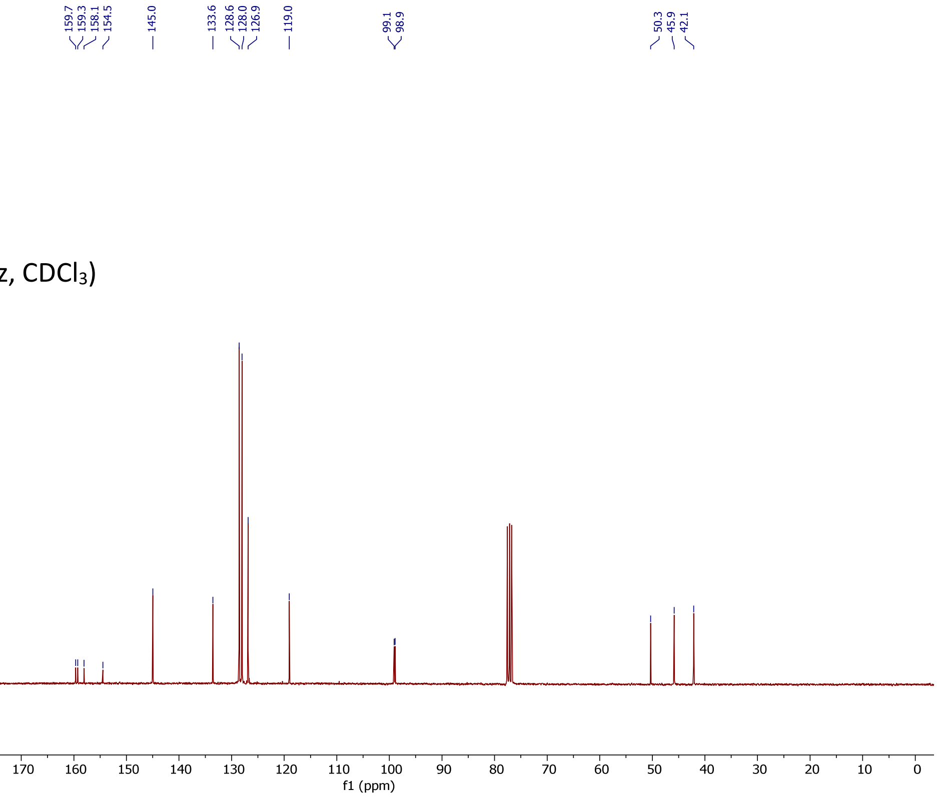

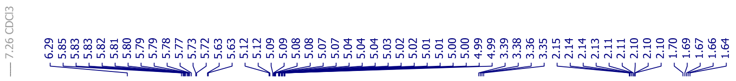

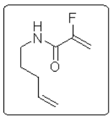

1i

^{1}H NMR (500 MHz, $CDCl_3$)

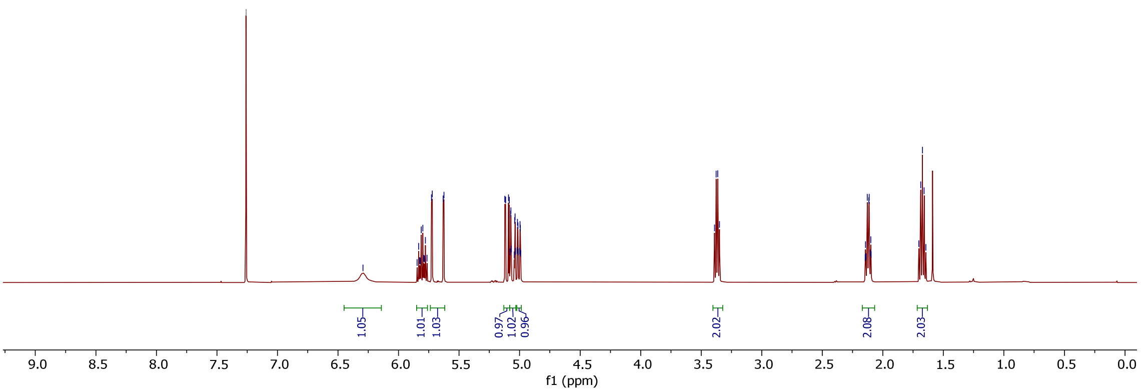

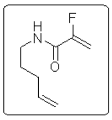

1i

^{19}F NMR (471 MHz, $CDCl_3$)

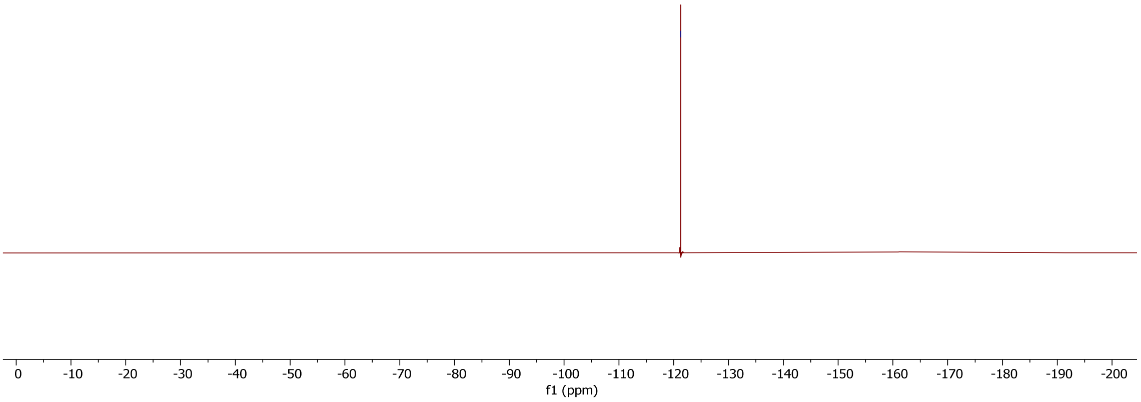

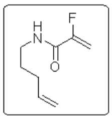

1i

^{13}C NMR (126 MHz, $CDCl_3$)

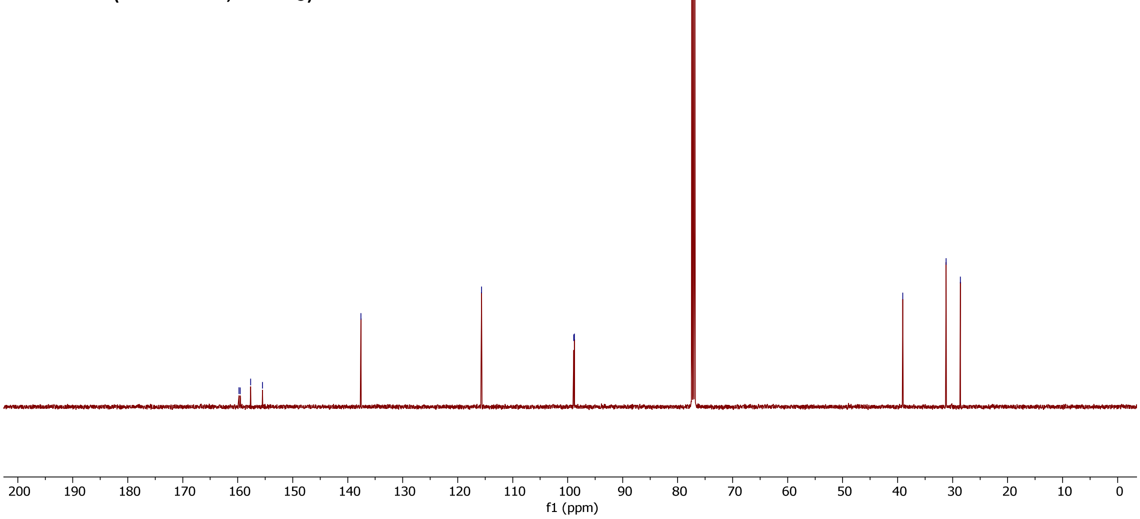

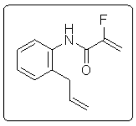

1j

^{1}H NMR (300 MHz, $CDCl_3$)

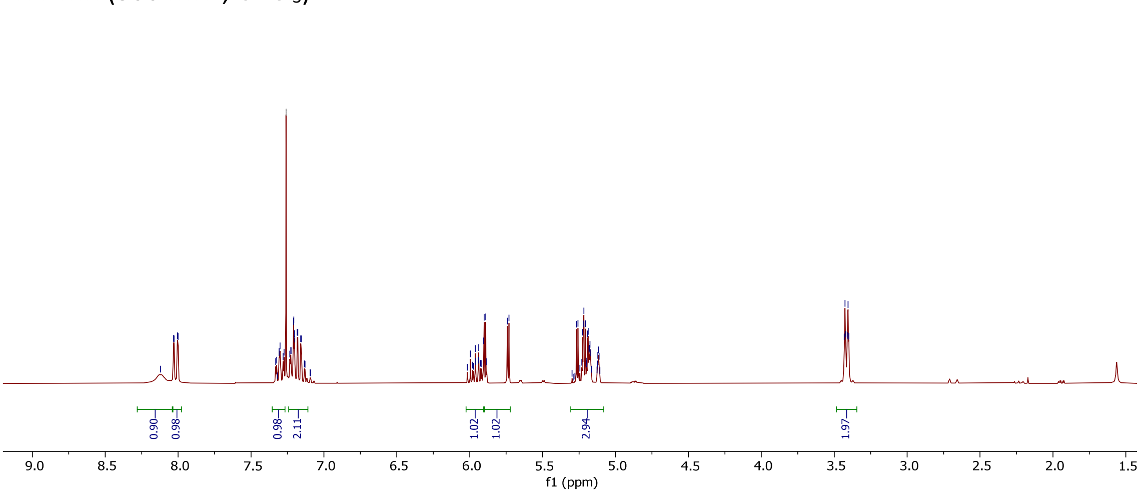

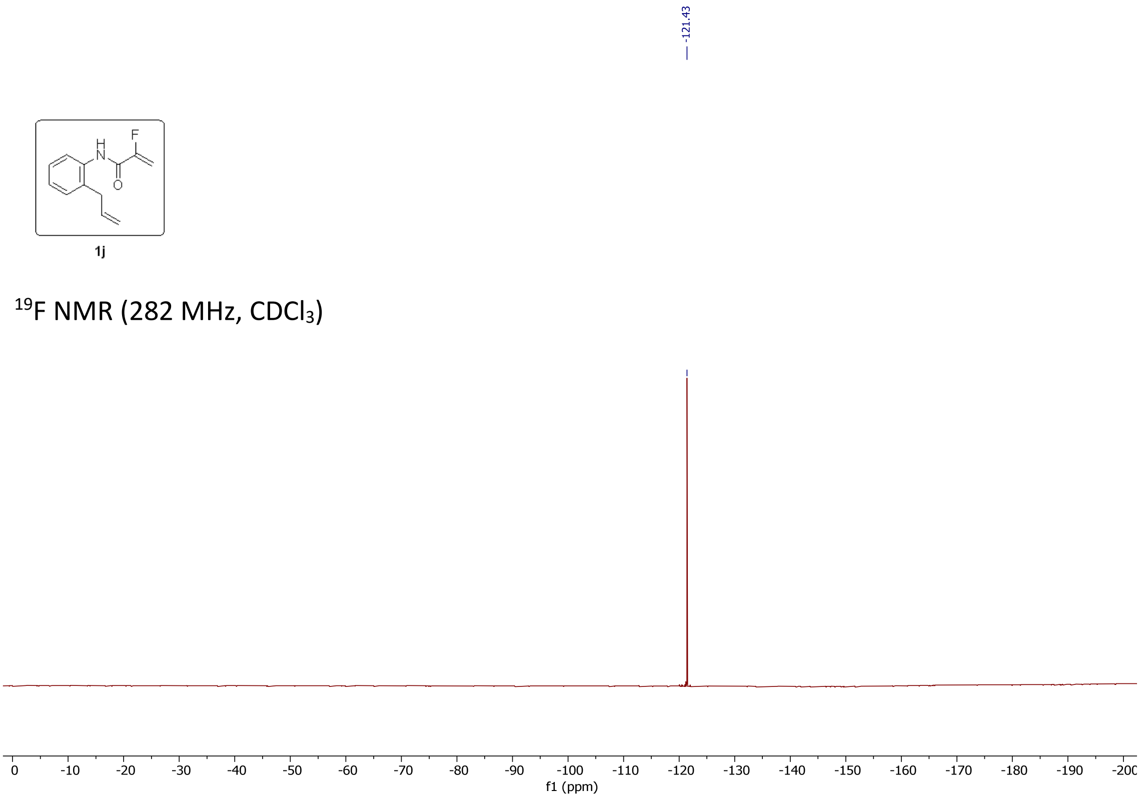

^{19}F NMR (282 MHz, $CDCl_3$)

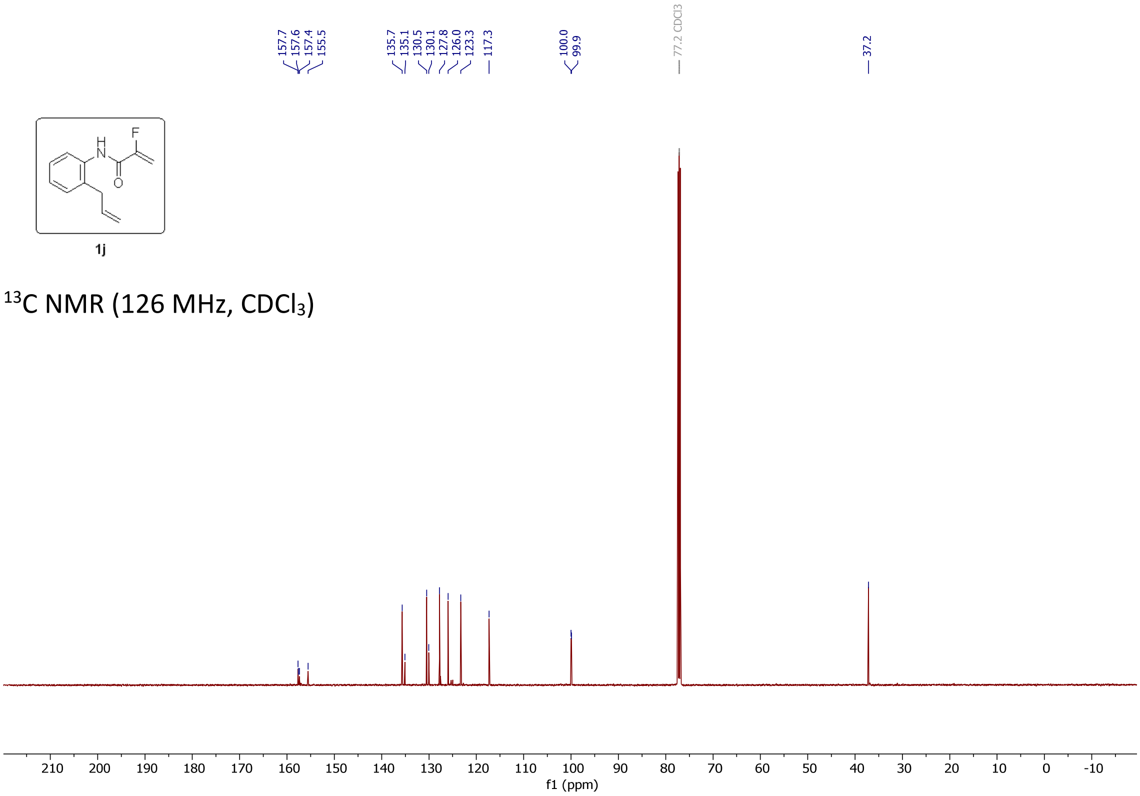

^{13}C NMR (126 MHz, $CDCl_3$)

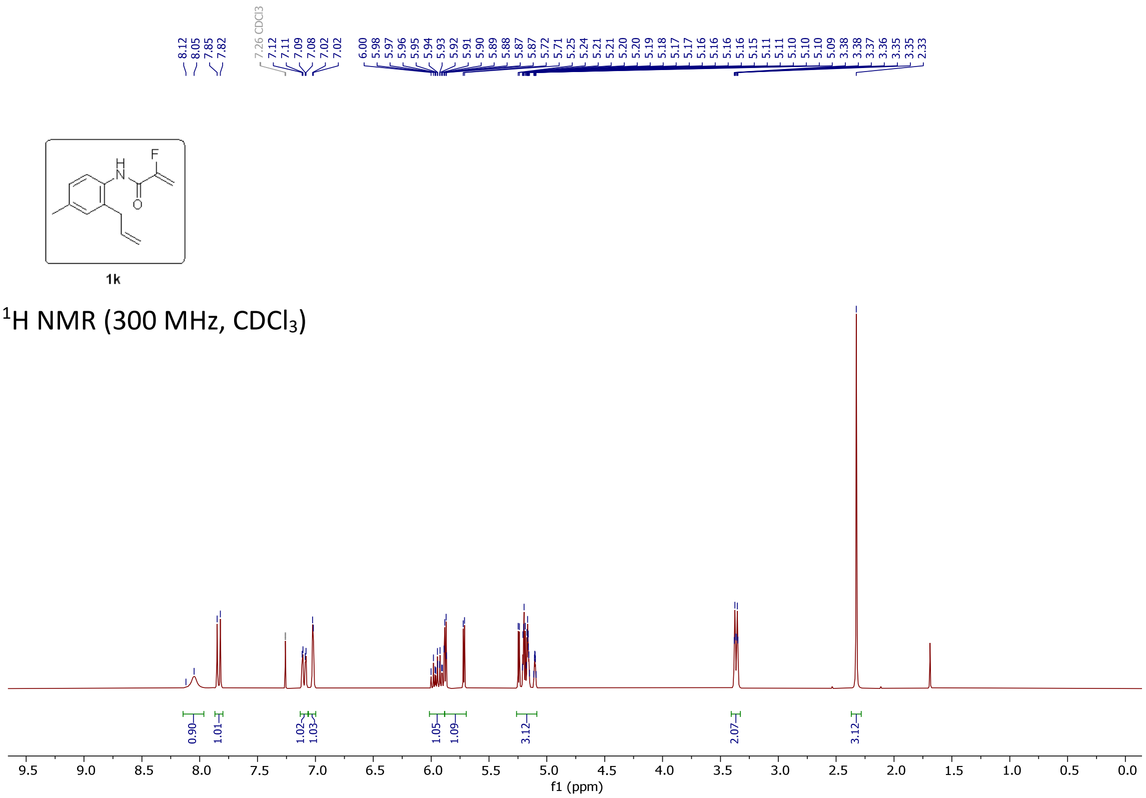

1k

^{1}H NMR (300 MHz, $CDCl_3$)

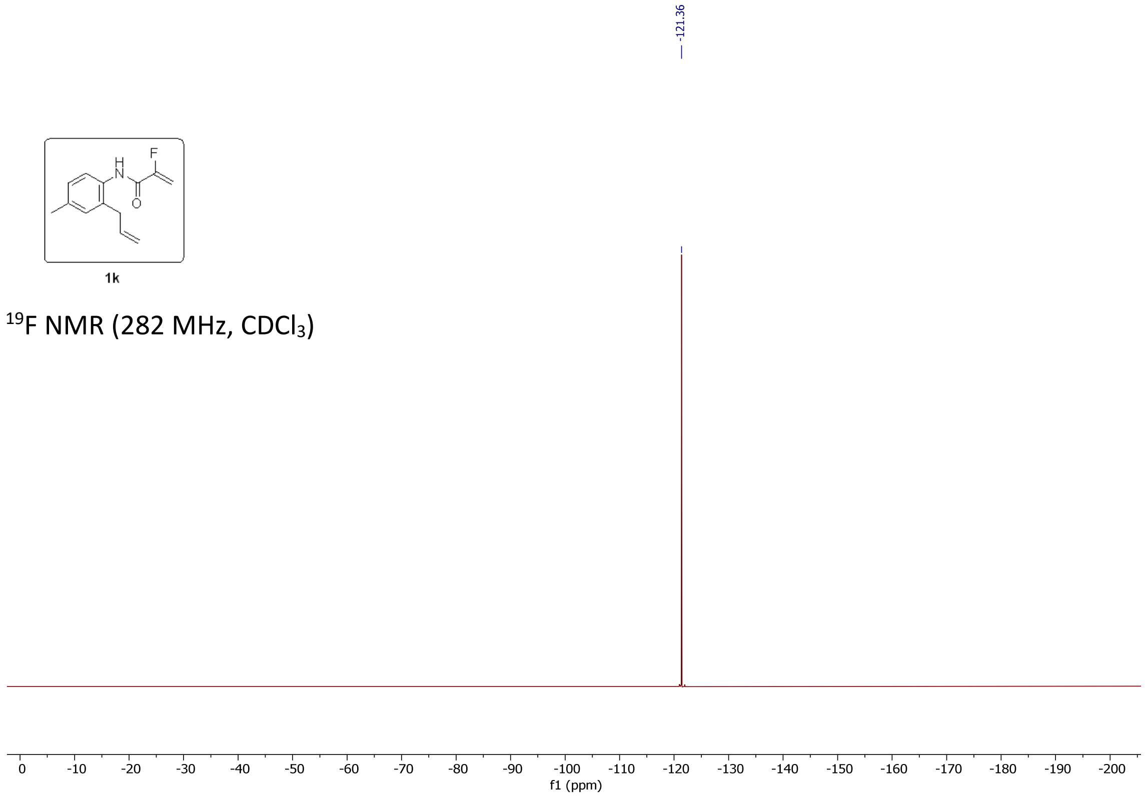

1k

^{19}F NMR (282 MHz, $CDCl_3$)

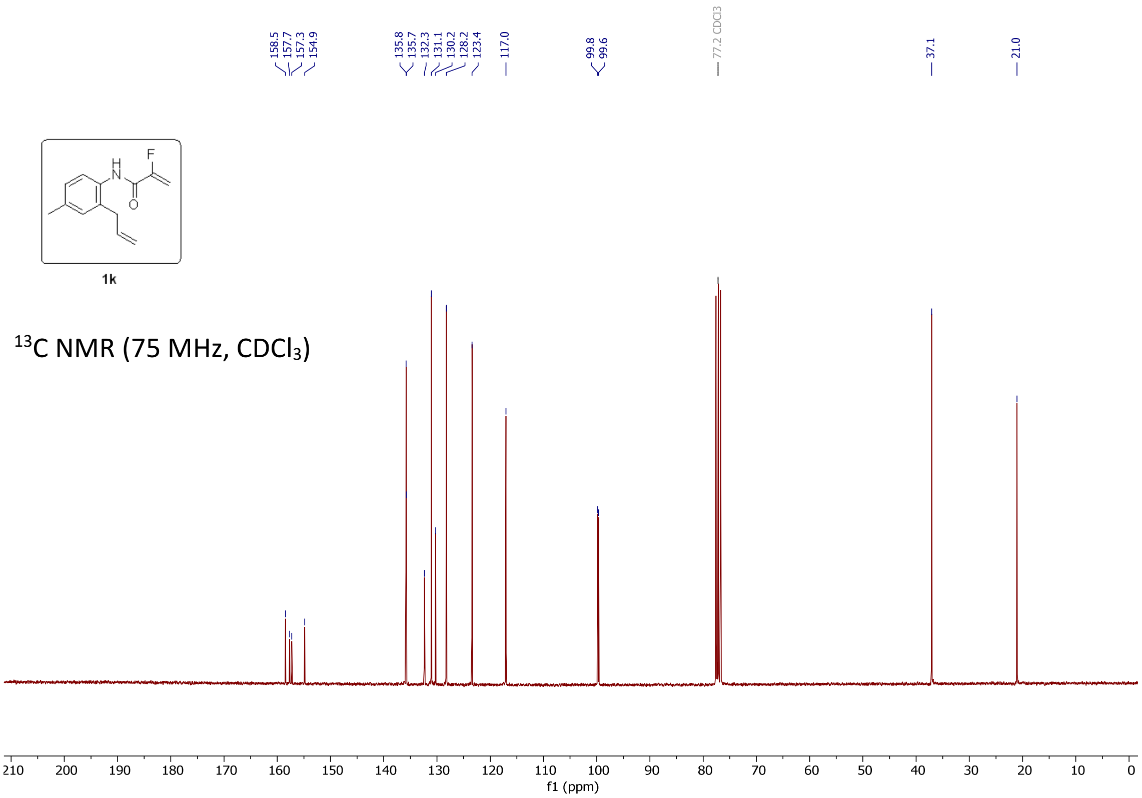
F
H
N
O
1k
13C NMR (75 MHz, CDCl3)
158.5
157.7
157.3
154.9
135.8
135.7
132.3
131.1
130.2
128.2
123.4
117.0
99.8
99.6
77.2 CDCl3
37.1
21.0
f1 (ppm)

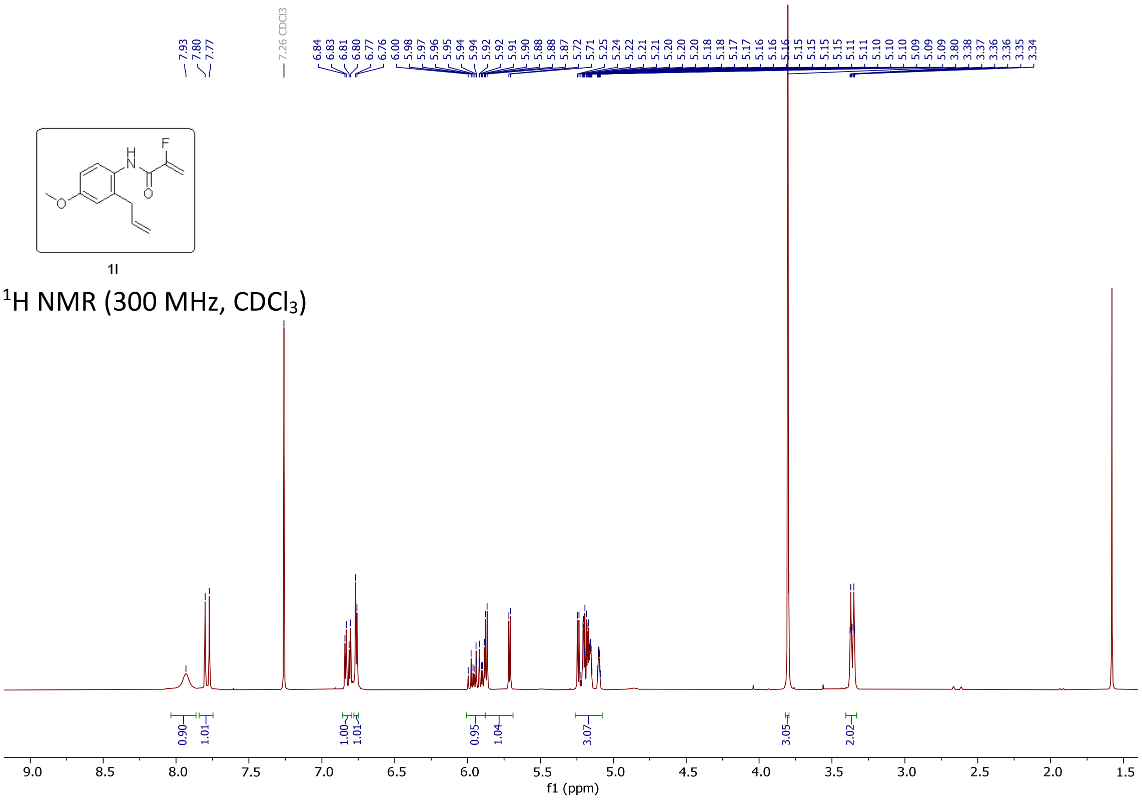
F
H
N
O
O
1l
1H NMR (300 MHz, CDCl3)
7.93
7.80
7.77
7.26 CDCl3
0.90
1.01
1.00
1.01
0.95
1.04
3.07
3.05
2.02
f1 (ppm)

1l

^{19}F NMR (282 MHz, $CDCl_3$)

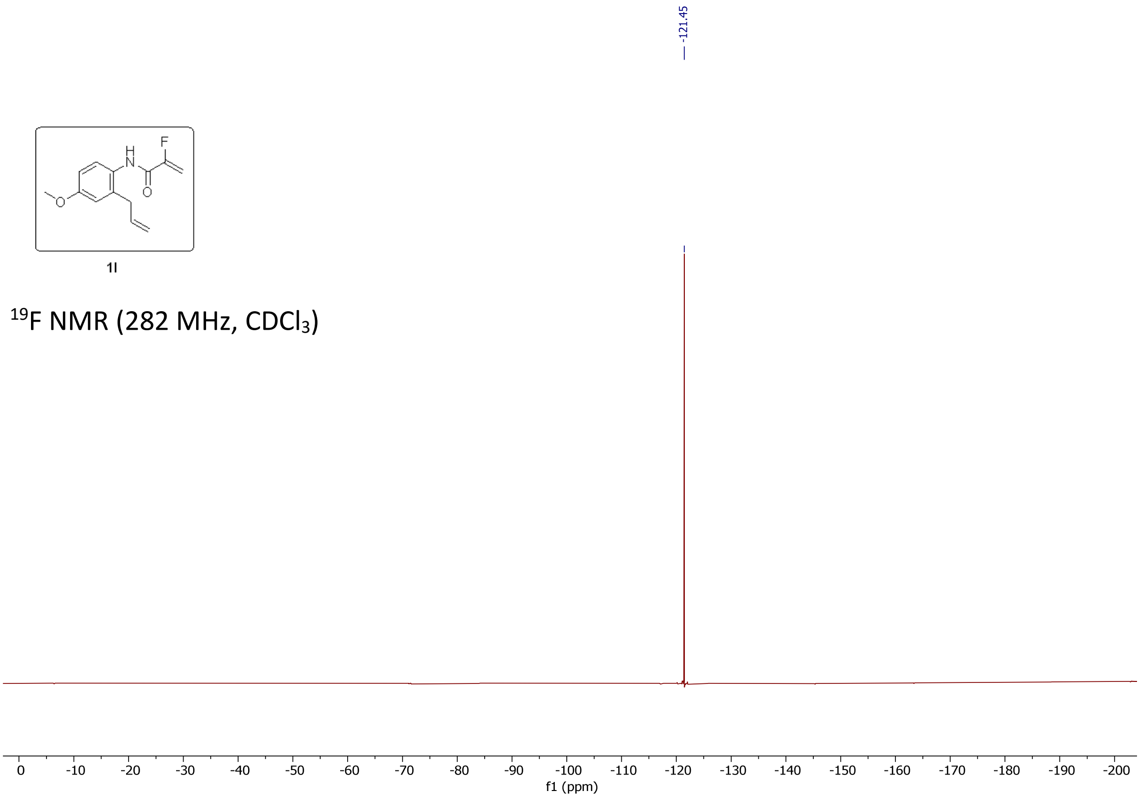

1l

^{13}C NMR (126 MHz, $CDCl_3$)

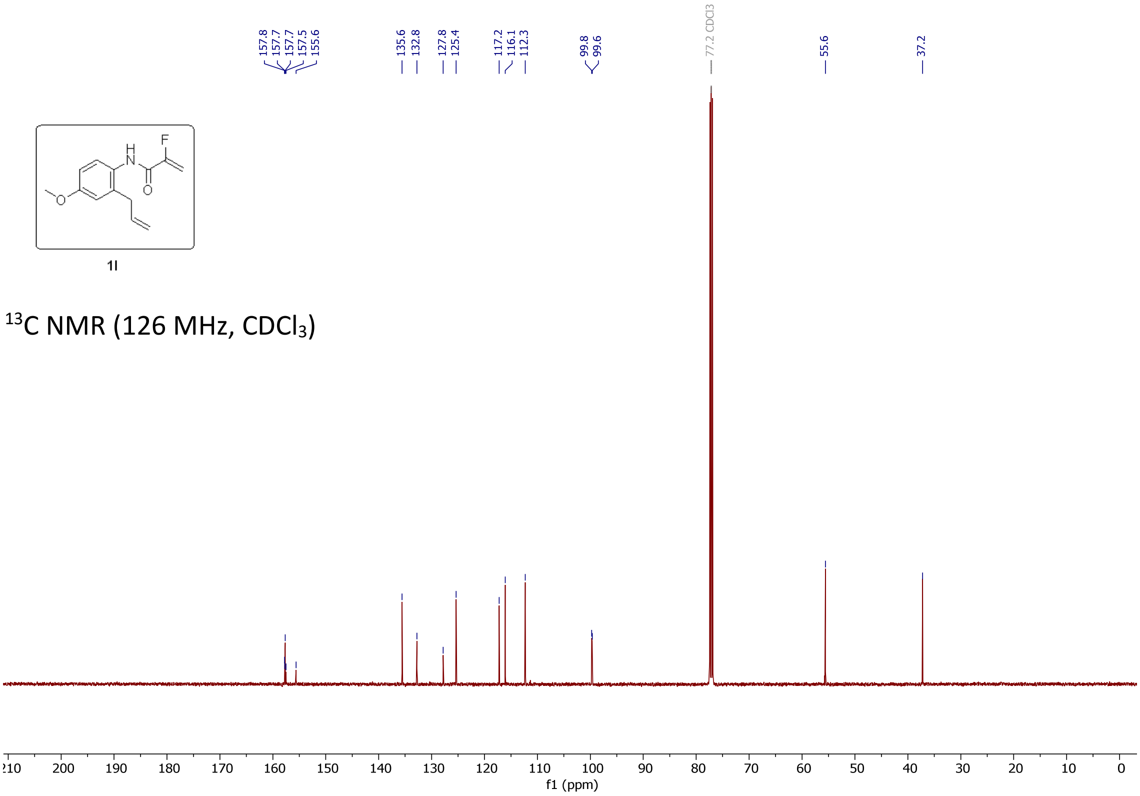

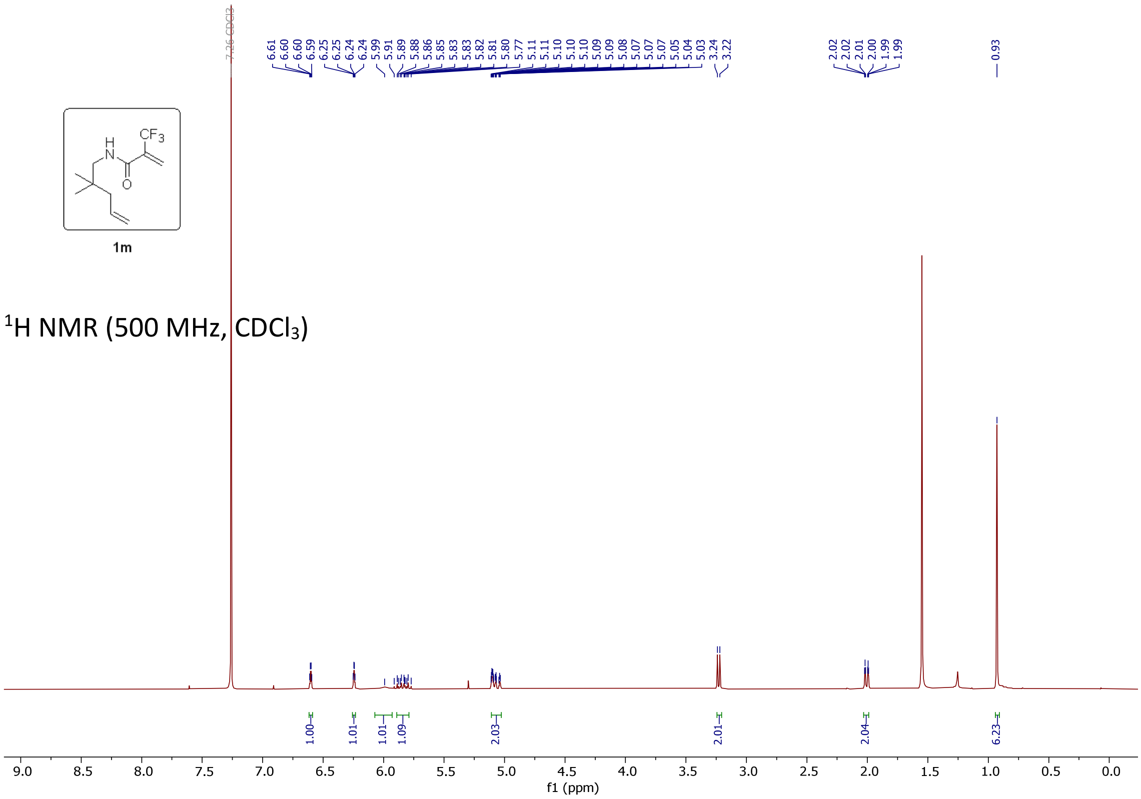
1m
^{1}H NMR (500 MHz, CDCl$_3$)
7.26 CDCl3
6.61 6.60 6.60 6.59 6.25 6.25 6.24 6.24 5.99 5.91 5.89 5.88 5.86 5.85 5.83 5.83 5.82 5.81 5.80 5.77 5.11 5.11 5.10 5.10 5.09 5.09 5.08 5.07 5.07 5.07 5.05 5.04 5.03 3.24 3.22
2.02 2.02 2.01 2.00 1.99 1.99
0.93
1.00 1.01 1.01 1.09 2.03 2.01 2.04 6.23
f1 (ppm)

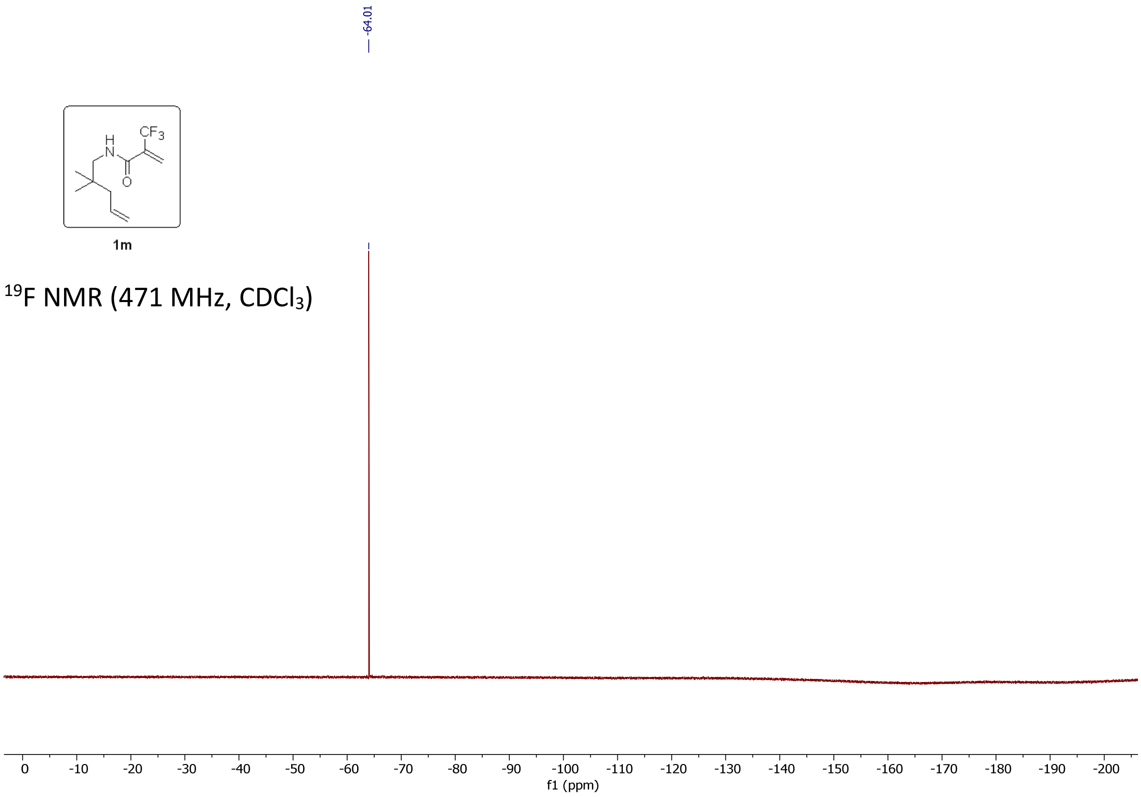
-64.01
1m
^{19}F NMR (471 MHz, CDCl$_3$)
f1 (ppm)

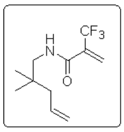

1m

^{13}C NMR (126 MHz, $CDCl_3$)

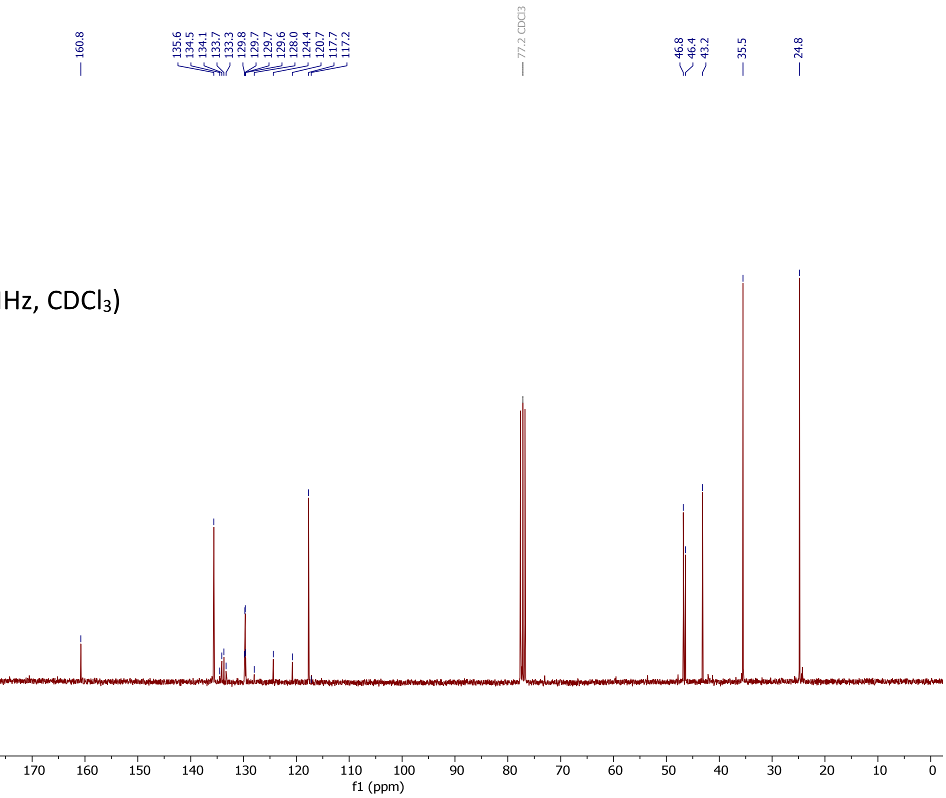

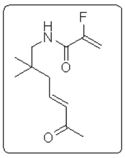

2a

^{1}H NMR (300 MHz, $CDCl_3$)

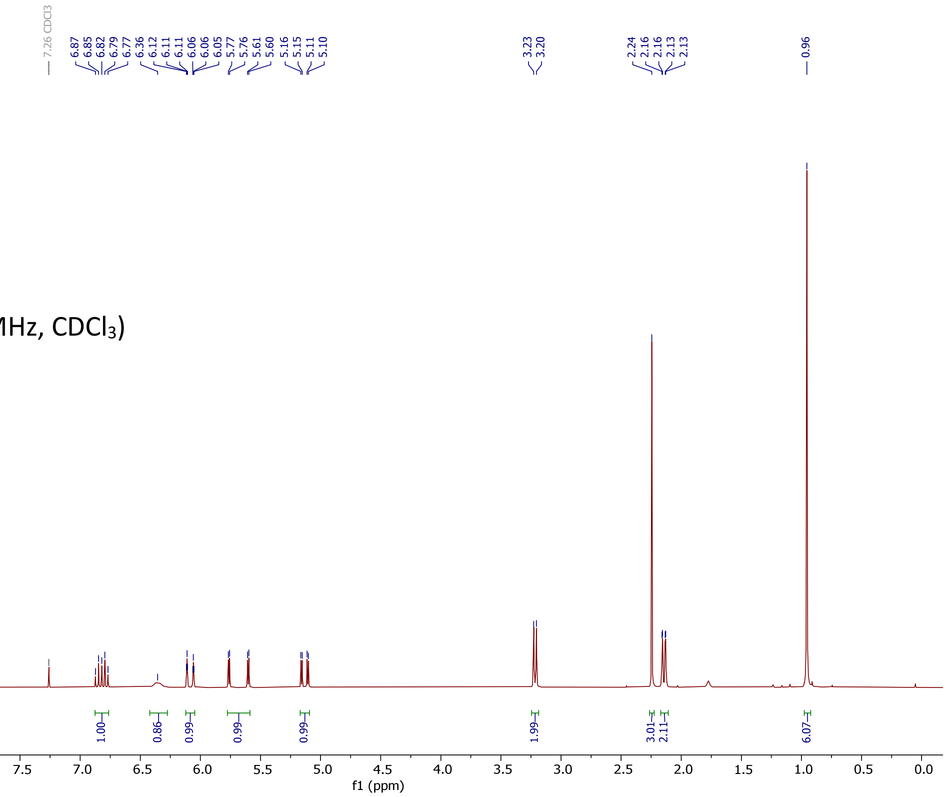

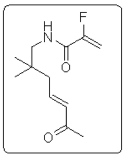

2a

^{19}F NMR (282 MHz, $CDCl_3$)

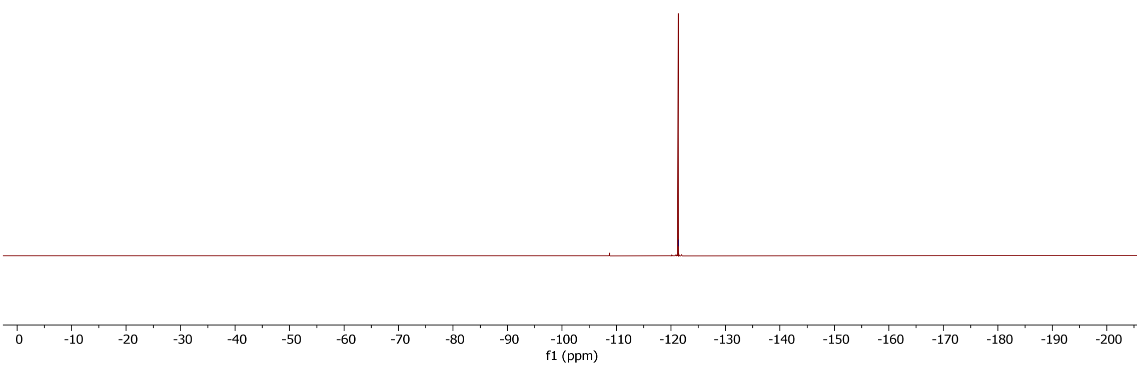

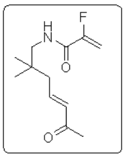

2a

^{13}C NMR (75 MHz, $CDCl_3$)

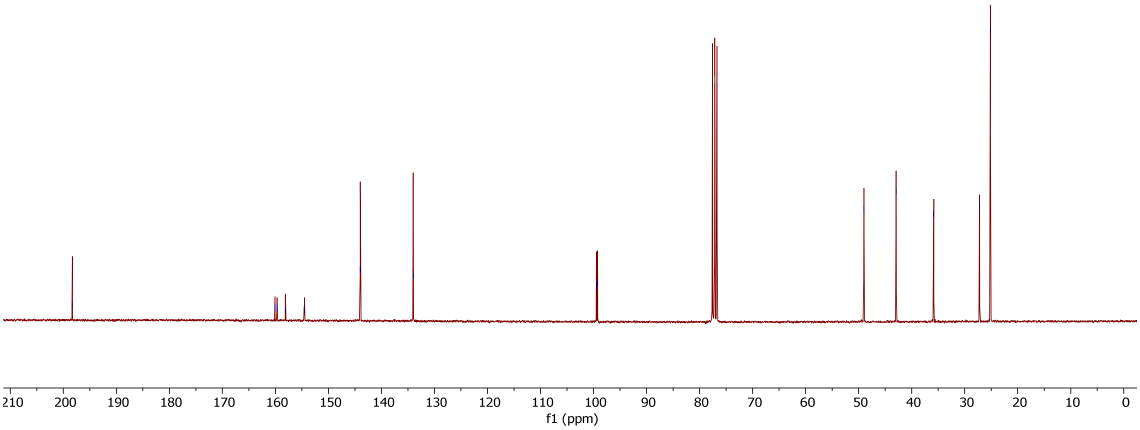

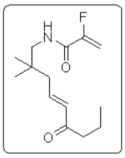

2b

^{1}H NMR (300 MHz, $CDCl_3$)

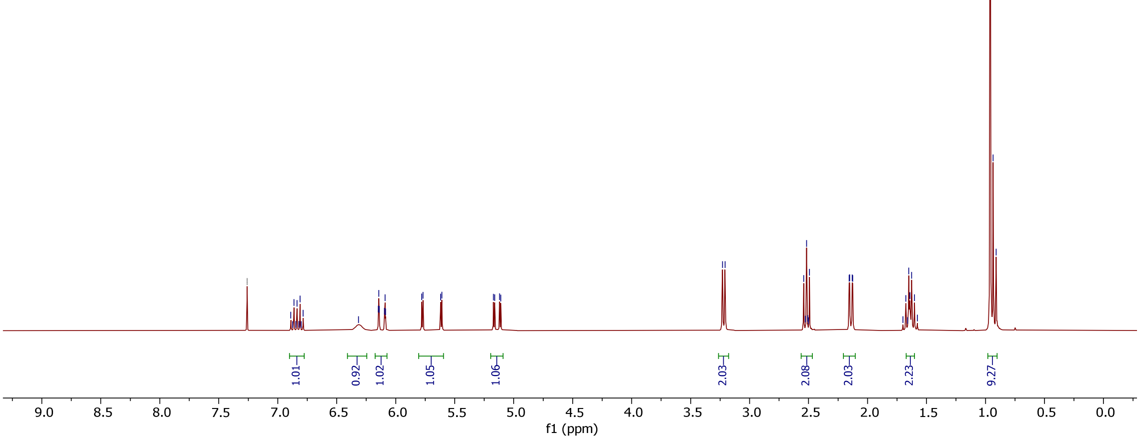

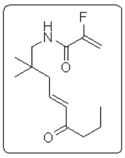

2b

^{19}F NMR (282 MHz, $CDCl_3$)

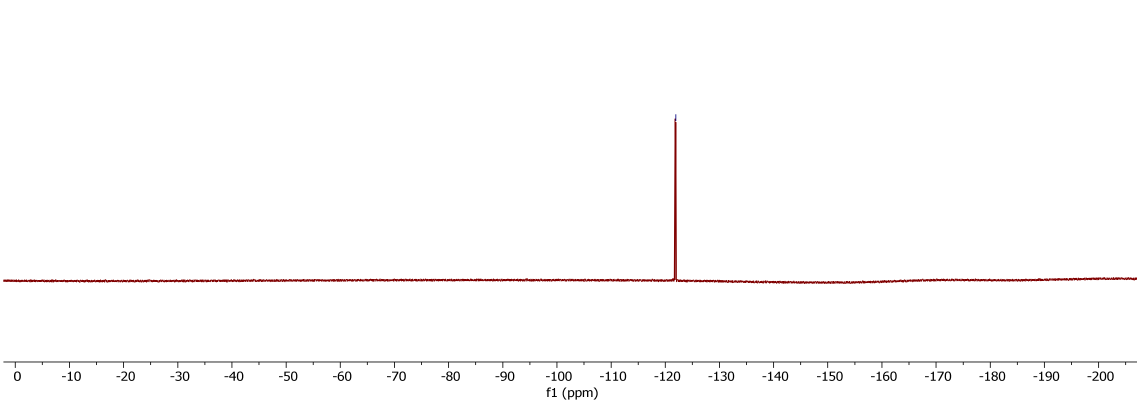

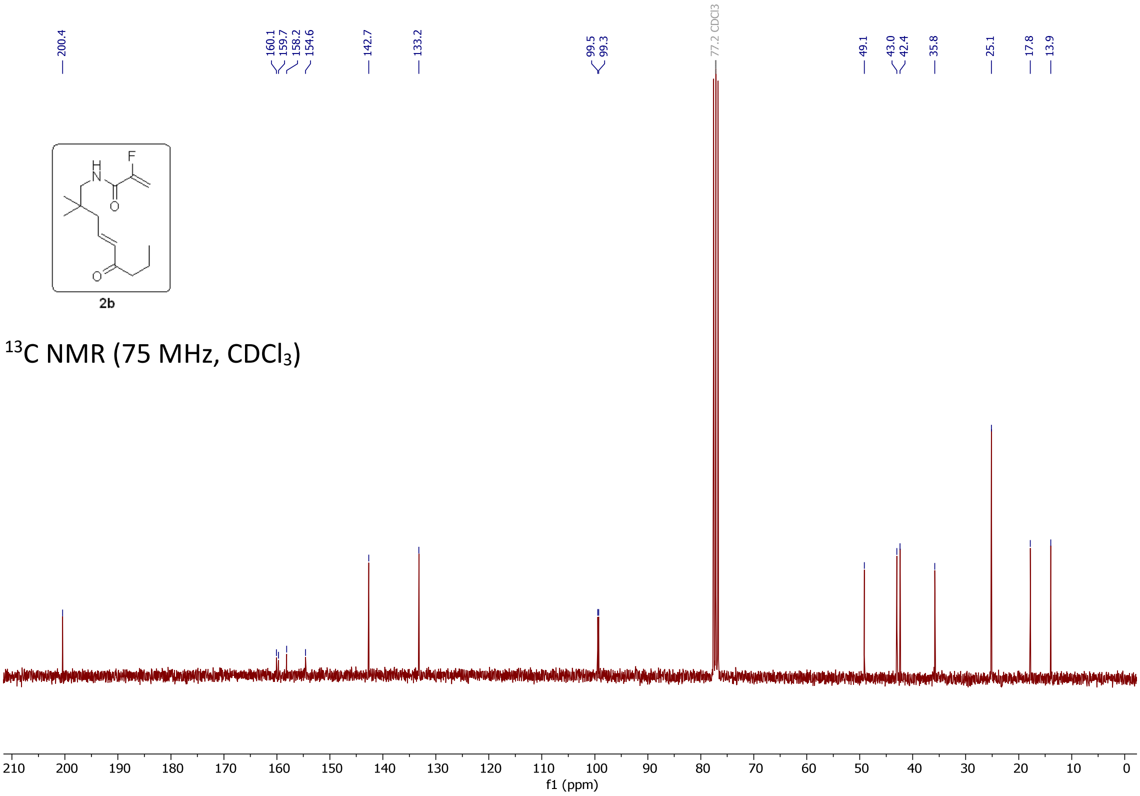

2b

^{13}C NMR (75 MHz, $CDCl_3$)

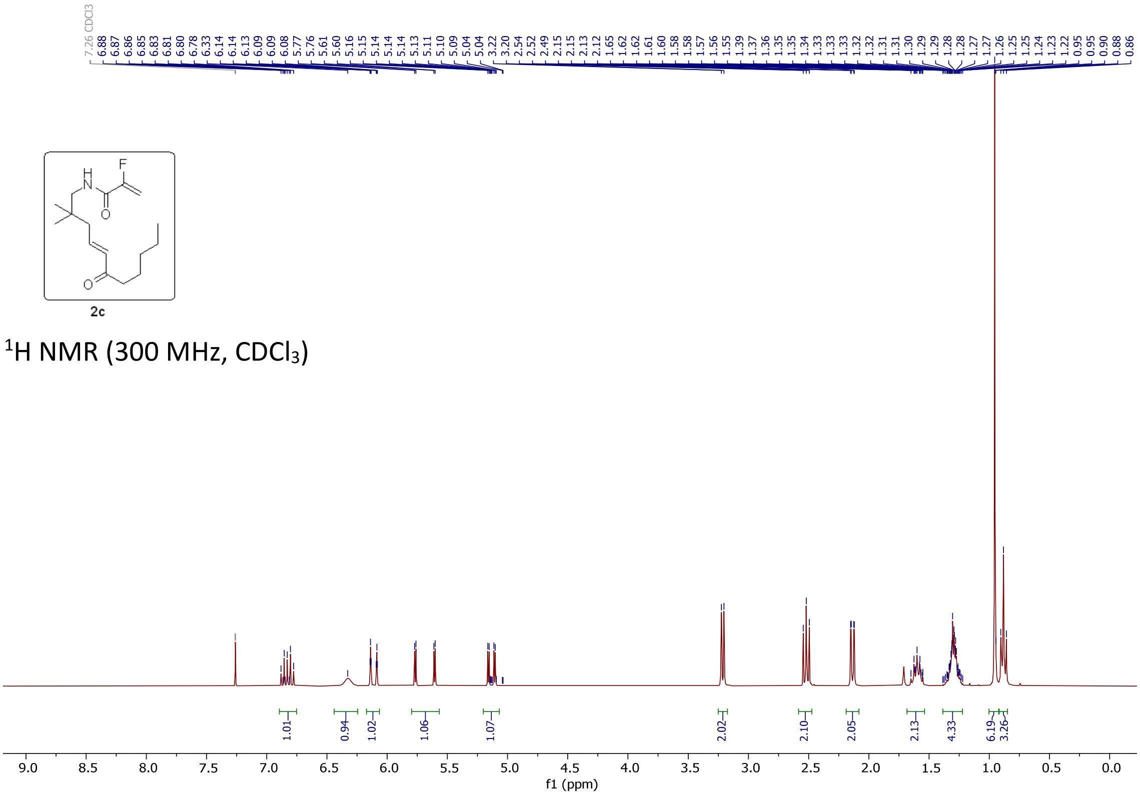

2c

^{1}H NMR (300 MHz, $CDCl_3$)

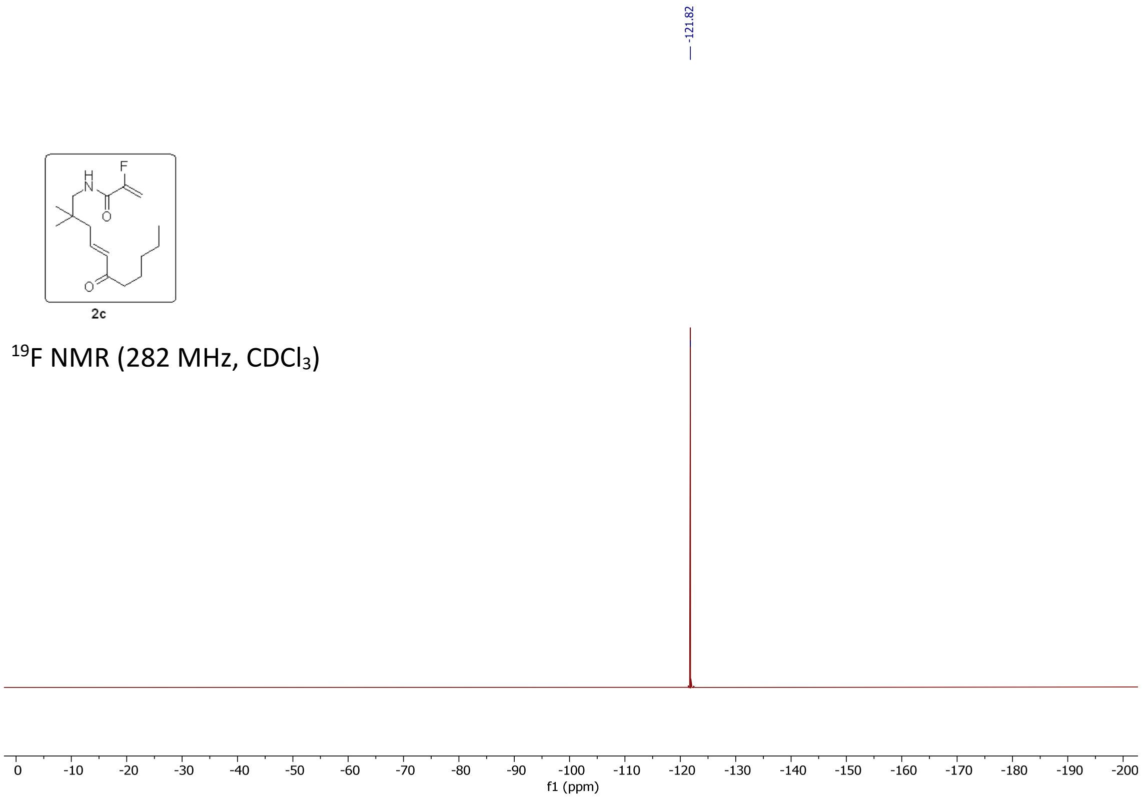

-121.82
F
N
H
O
O
2c
19F NMR (282 MHz, CDCl3)
0 -10 -20 -30 -40 -50 -60 -70 -80 -90 -100 -110 -120 -130 -140 -150 -160 -170 -180 -190 -200
f1 (ppm)

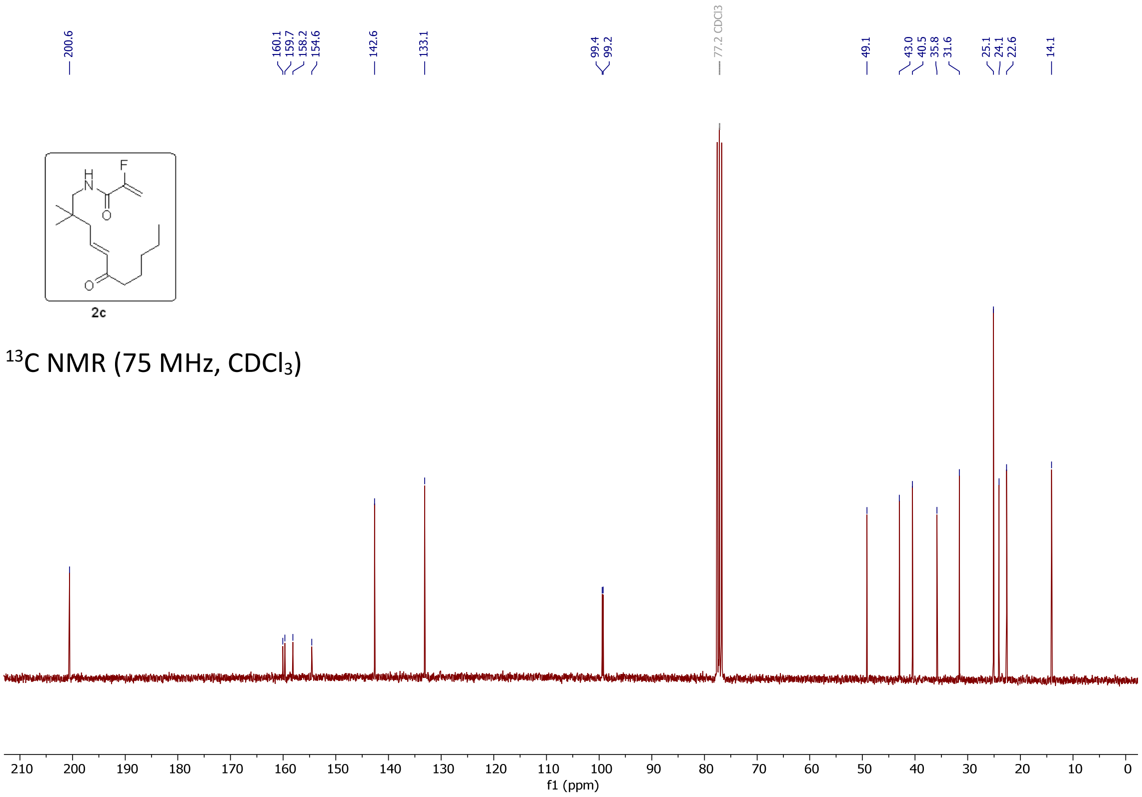

200.6
160.1
159.7
158.2
154.6
142.6
133.1
99.4
99.2
77.2 CDCl3
49.1
43.0
40.5
35.8
31.6
25.1
24.1
22.6
14.1
F
N
H
O
O
2c
13C NMR (75 MHz, CDCl3)
210 200 190 180 170 160 150 140 130 120 110 100 90 80 70 60 50 40 30 20 10 0
f1 (ppm)

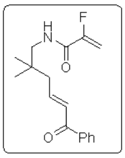

2d

^{1}H NMR (300 MHz, $CDCl_3$)

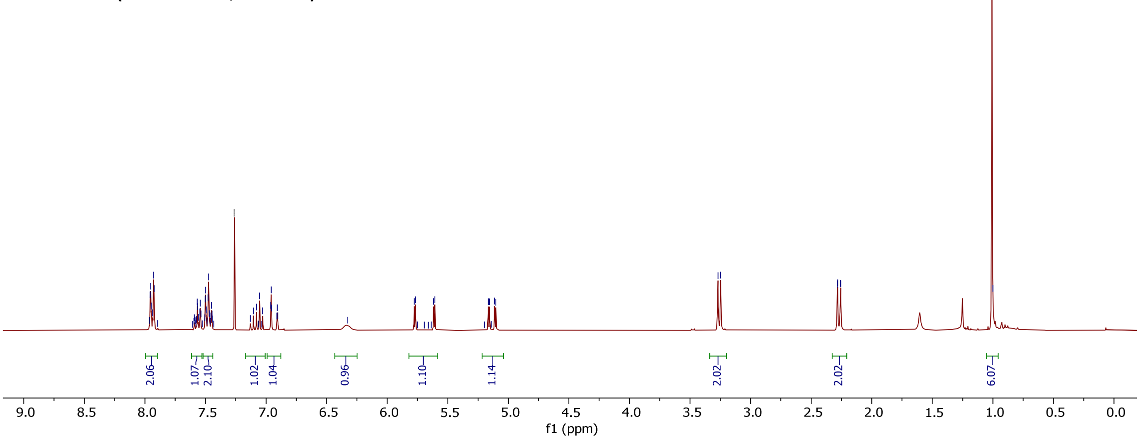

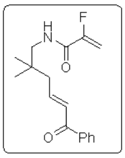

2d

^{19}F NMR (282 MHz, $CDCl_3$)

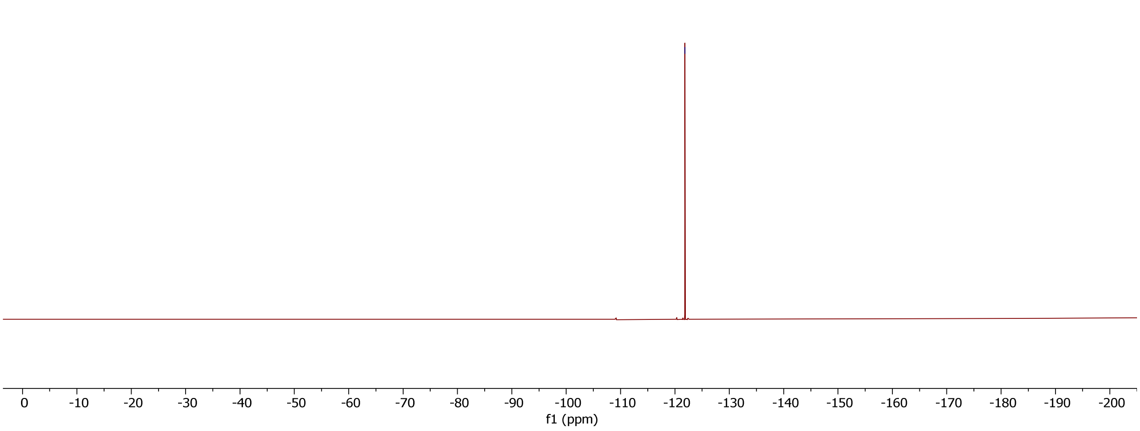

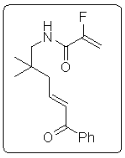

2d

^{13}C NMR (75 MHz, $CDCl_3$)

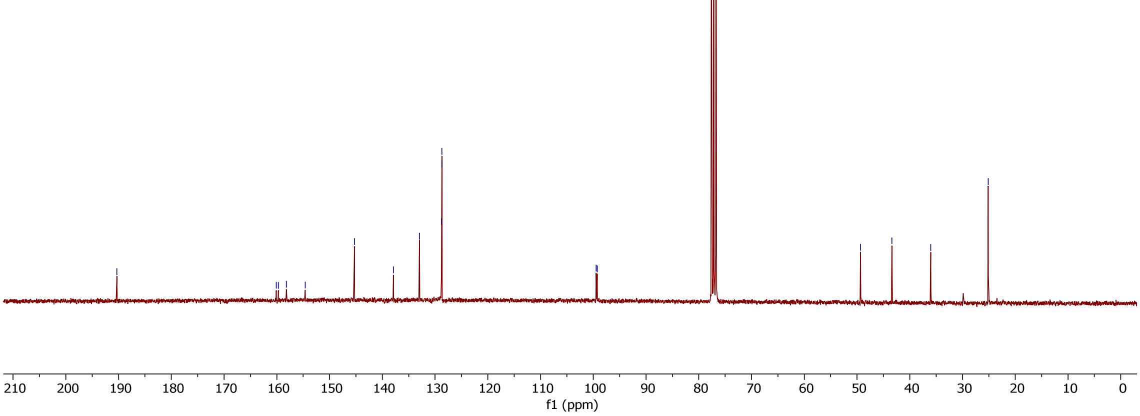

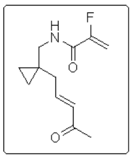

2e

^{1}H NMR (300 MHz, $CDCl_3$)

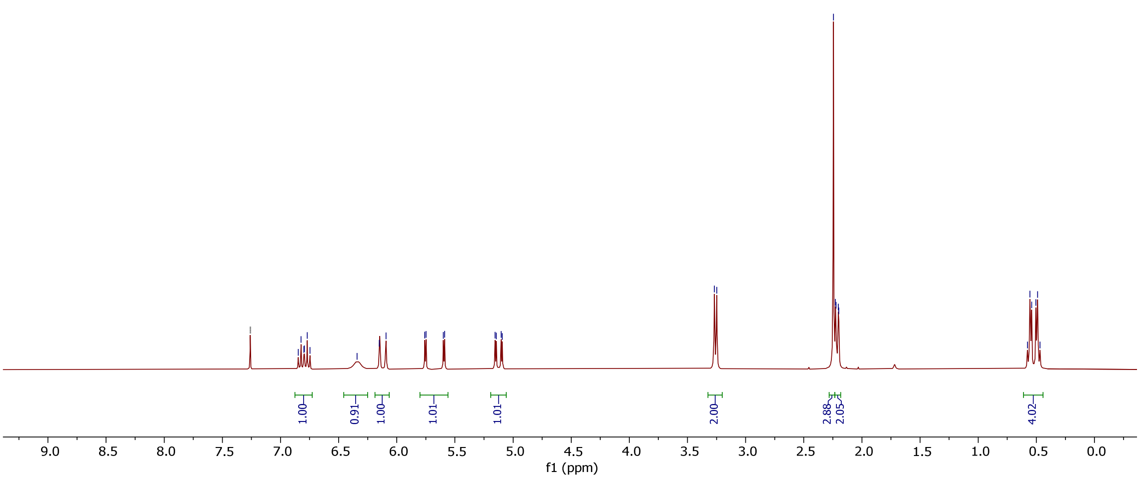

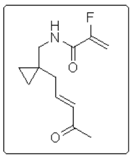

2e

^{19}F NMR (282 MHz, $CDCl_3$)

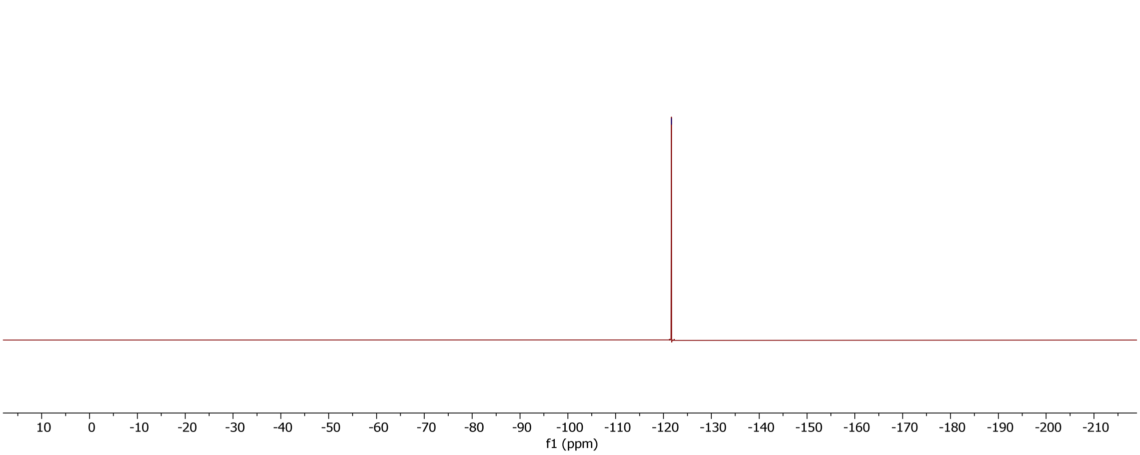

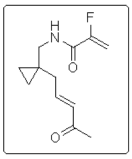

2e

^{13}C NMR (75 MHz, $CDCl_3$)

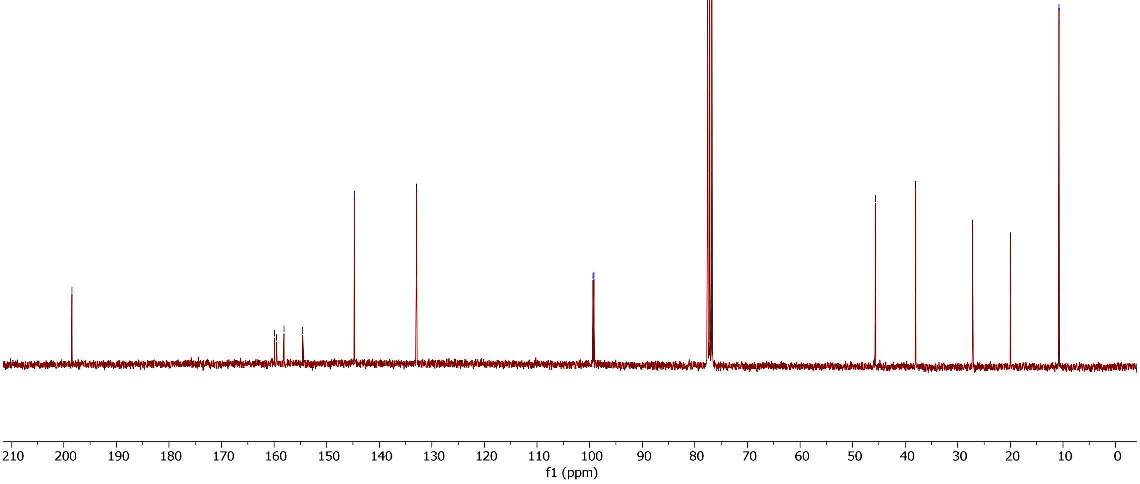

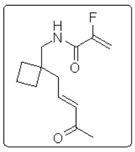

2f

^{1}H NMR (500 MHz, $CDCl_3$)

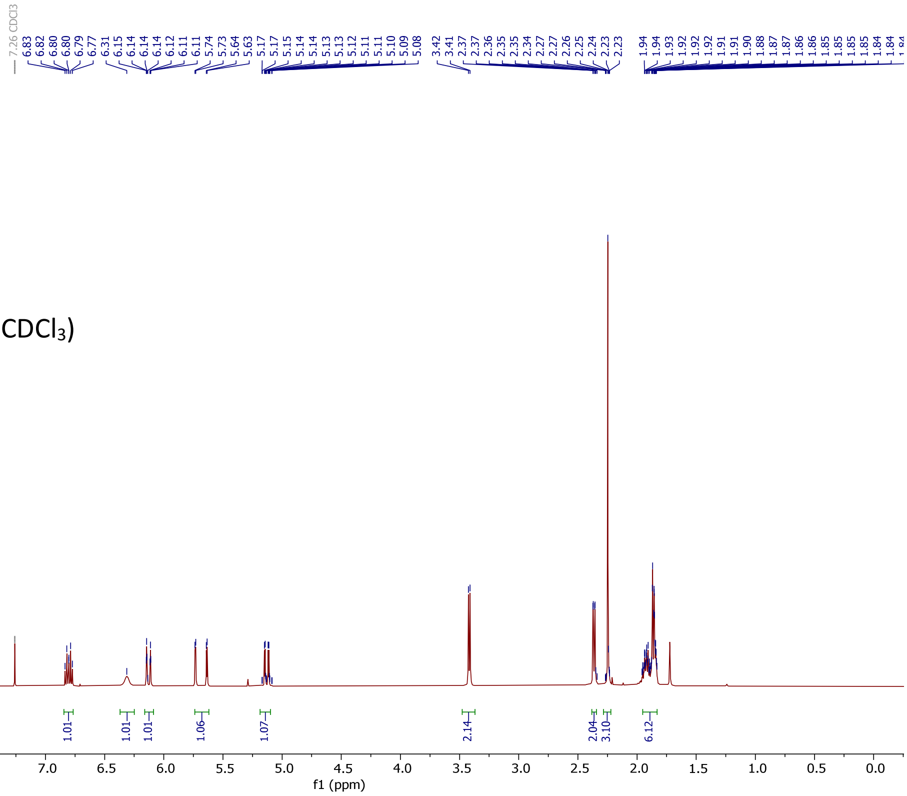

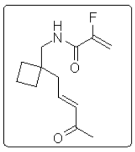

2f

^{19}F NMR (471 MHz, $CDCl_3$)

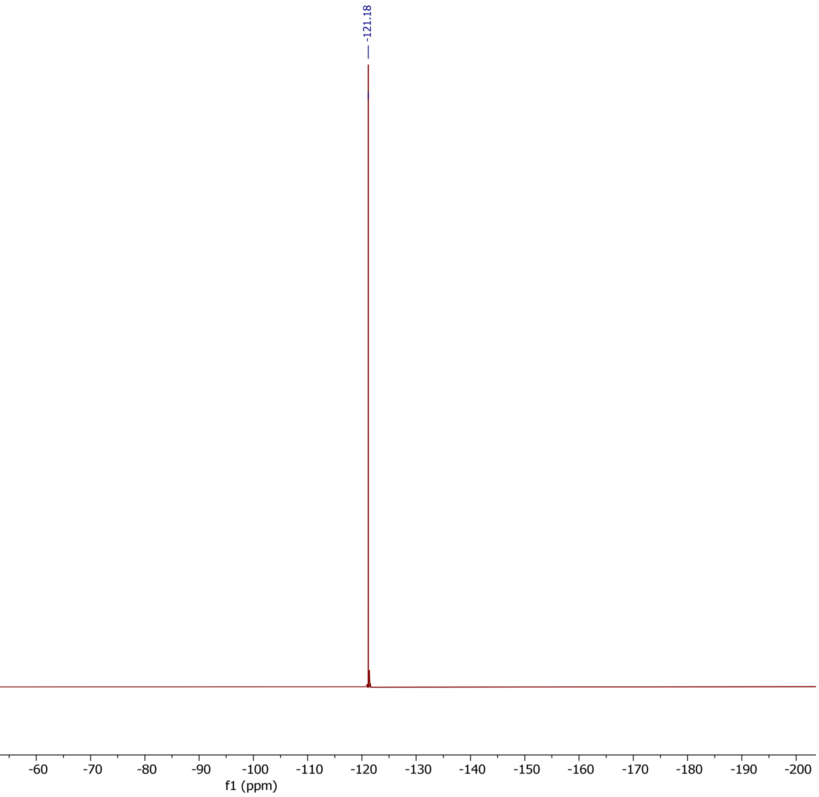

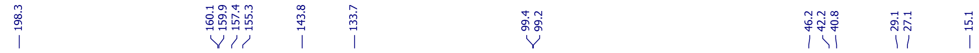

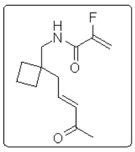

2f

^{13}C NMR (126 MHz, $CDCl_3$)

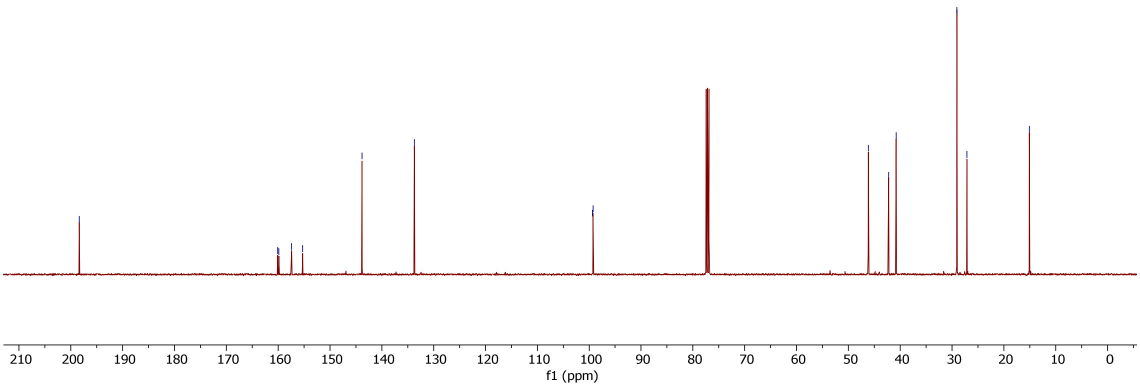

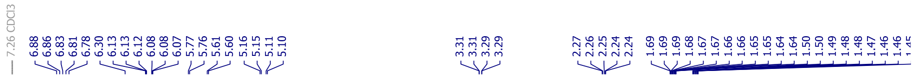

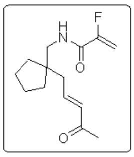

2g

^{1}H NMR (300 MHz, $CDCl_3$)

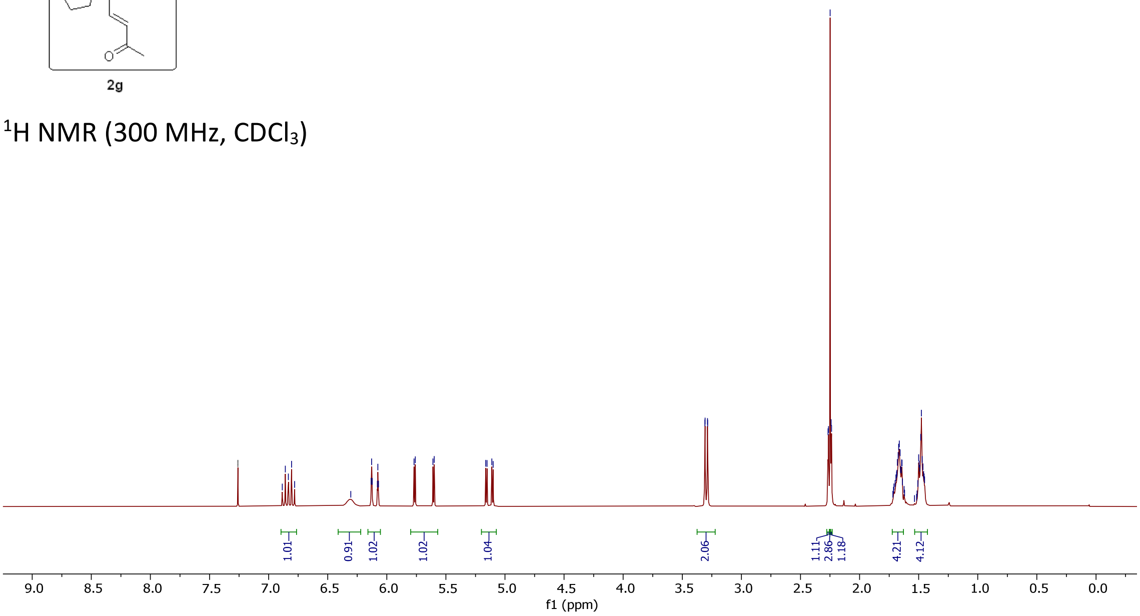

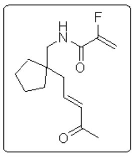

2g

^{19}F NMR (282 MHz, $CDCl_3$)

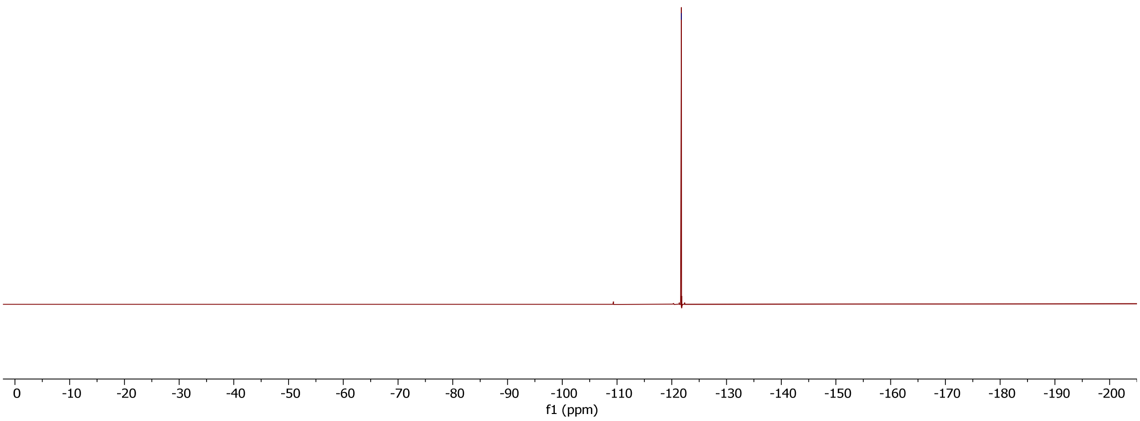

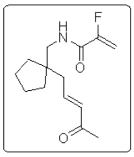

2g

^{13}C NMR (126 MHz, $CDCl_3$)

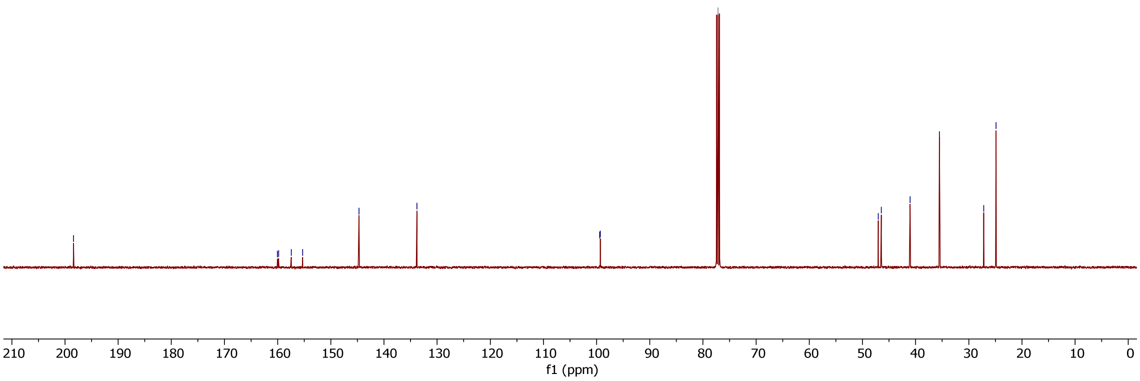

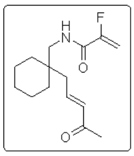

2h

^{1}H NMR (300 MHz, $CDCl_3$)

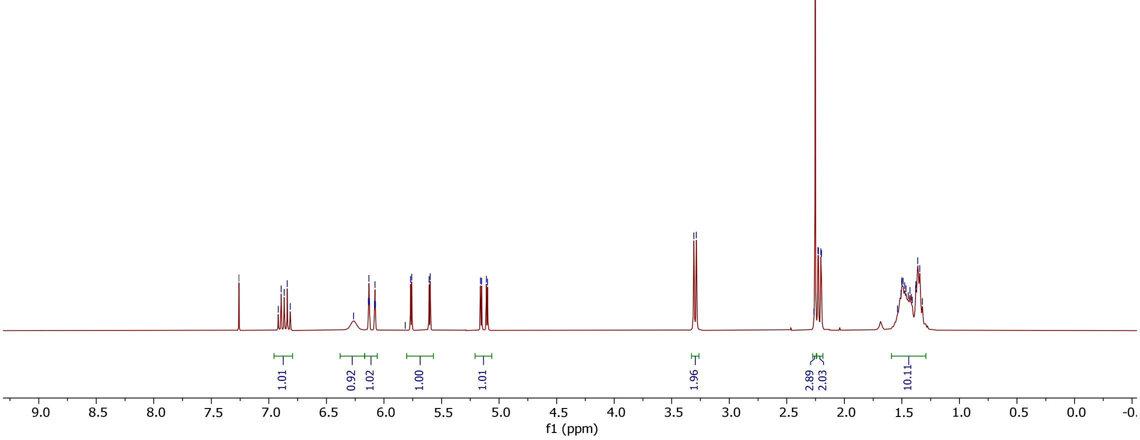

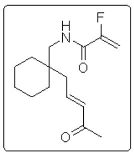

2h

^{19}F NMR (282 MHz, $CDCl_3$)

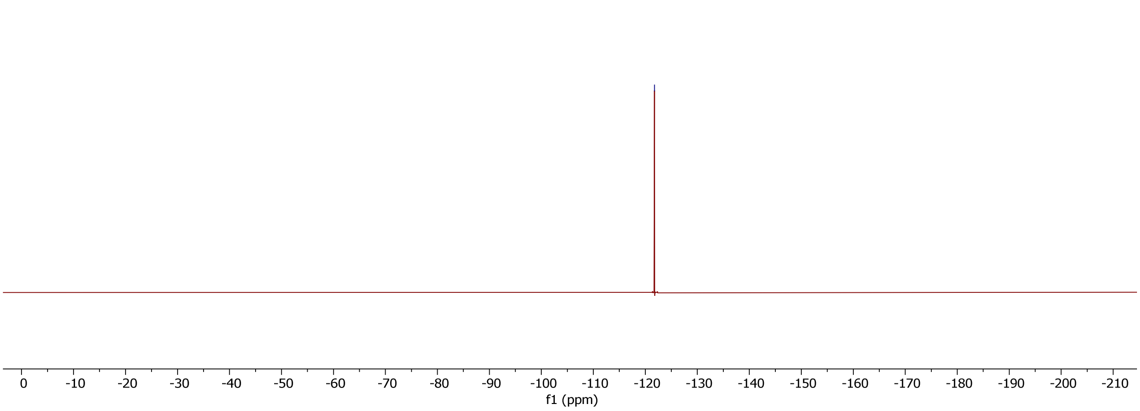

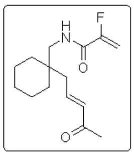

2h

^{13}C NMR (75 MHz, $CDCl_3$)

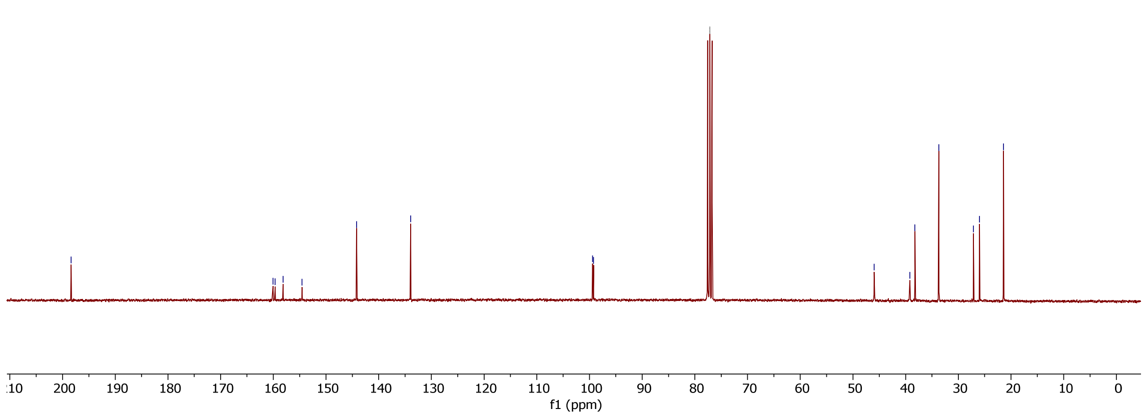

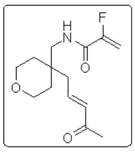

2i

^{1}H NMR (300 MHz, $CDCl_3$)

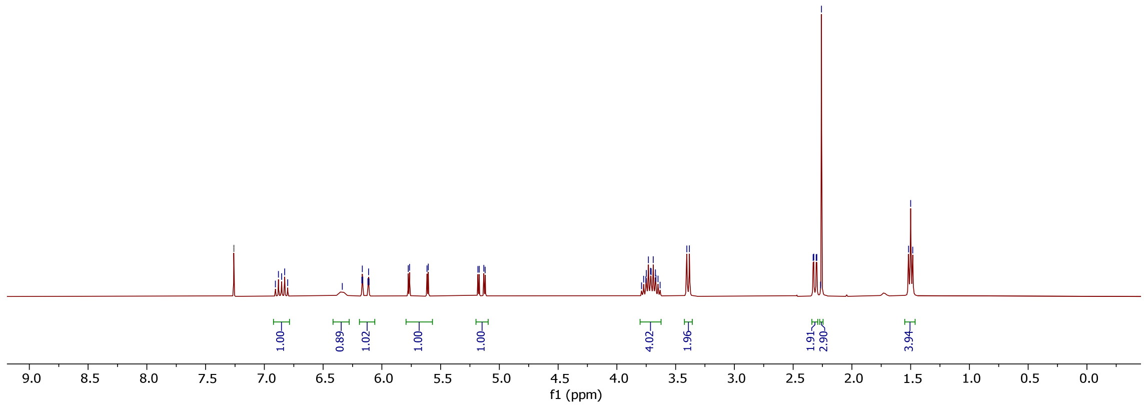

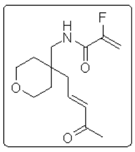

2i

^{19}F NMR (282 MHz, $CDCl_3$)

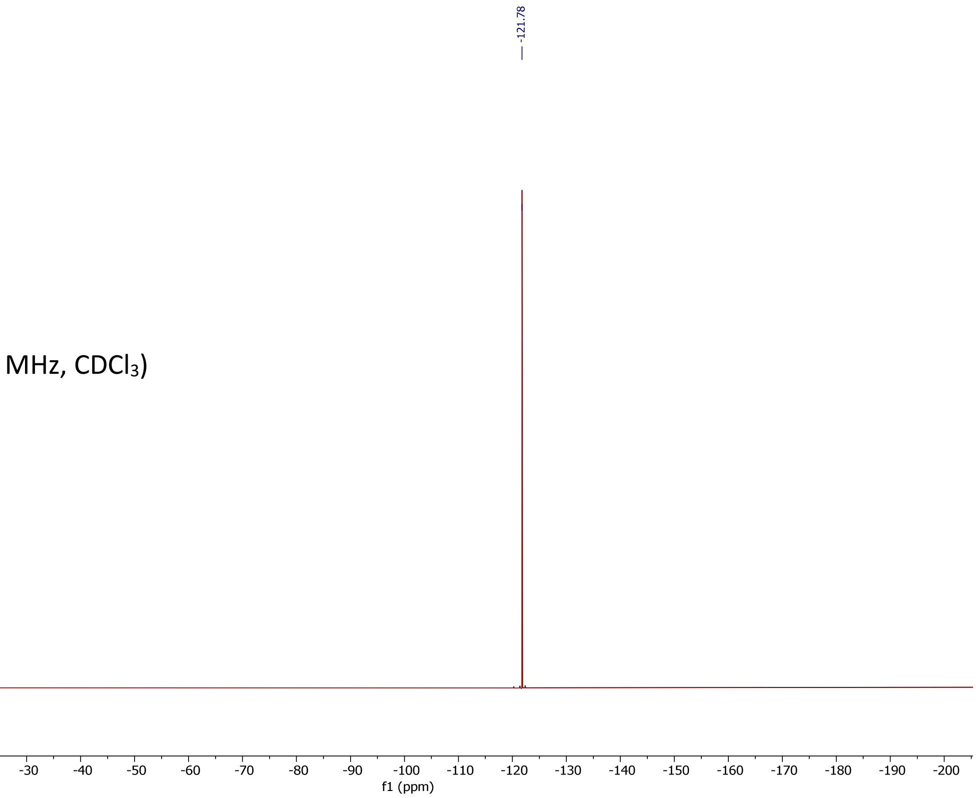

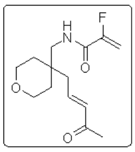

2i

^{13}C NMR (126 MHz, $CDCl_3$)

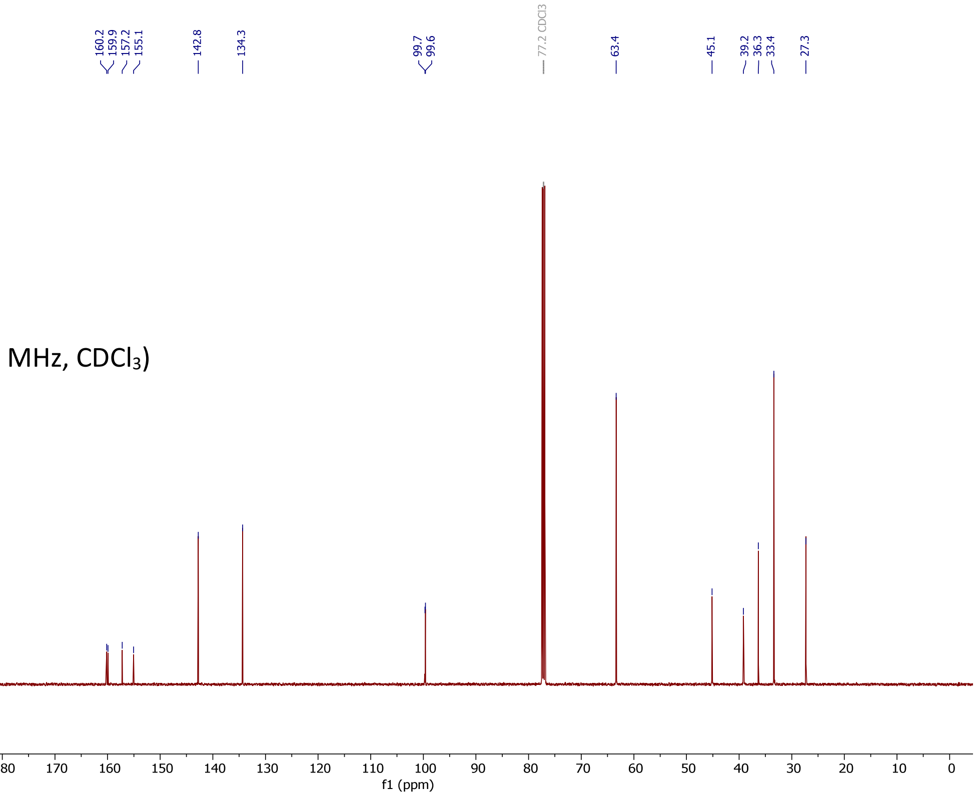

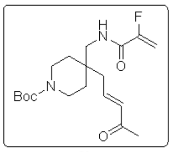

2j

^{1}H NMR (500 MHz, $CDCl_3$)

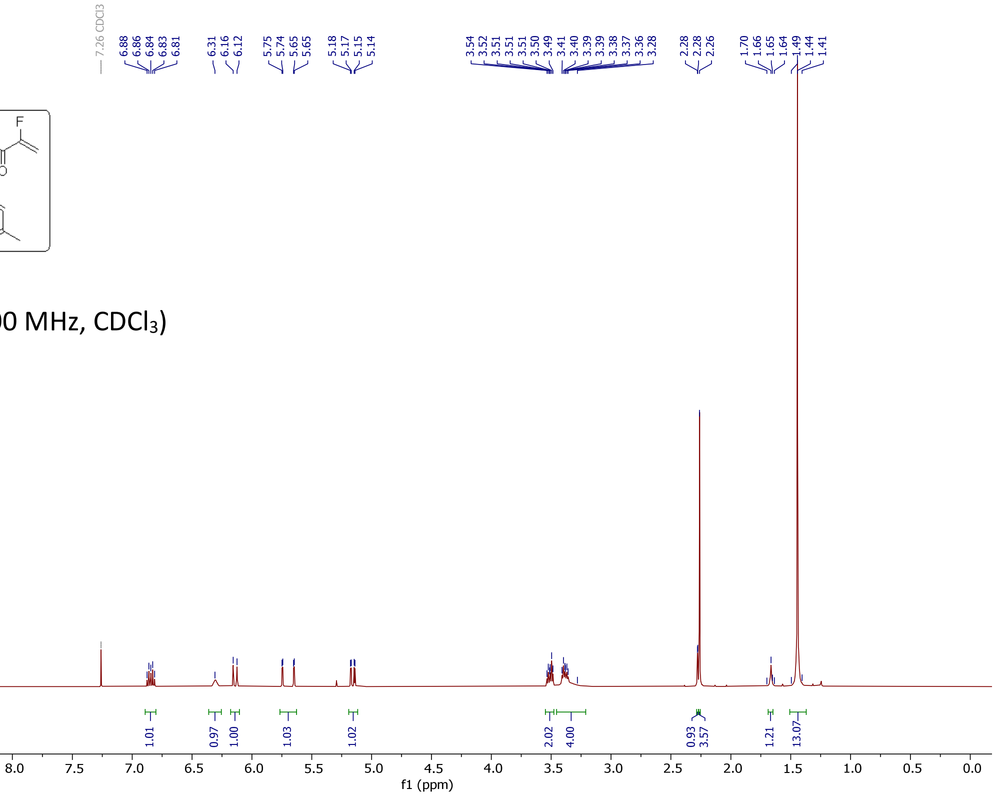

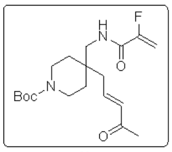

2j

^{19}F NMR (471 MHz, $CDCl_3$)

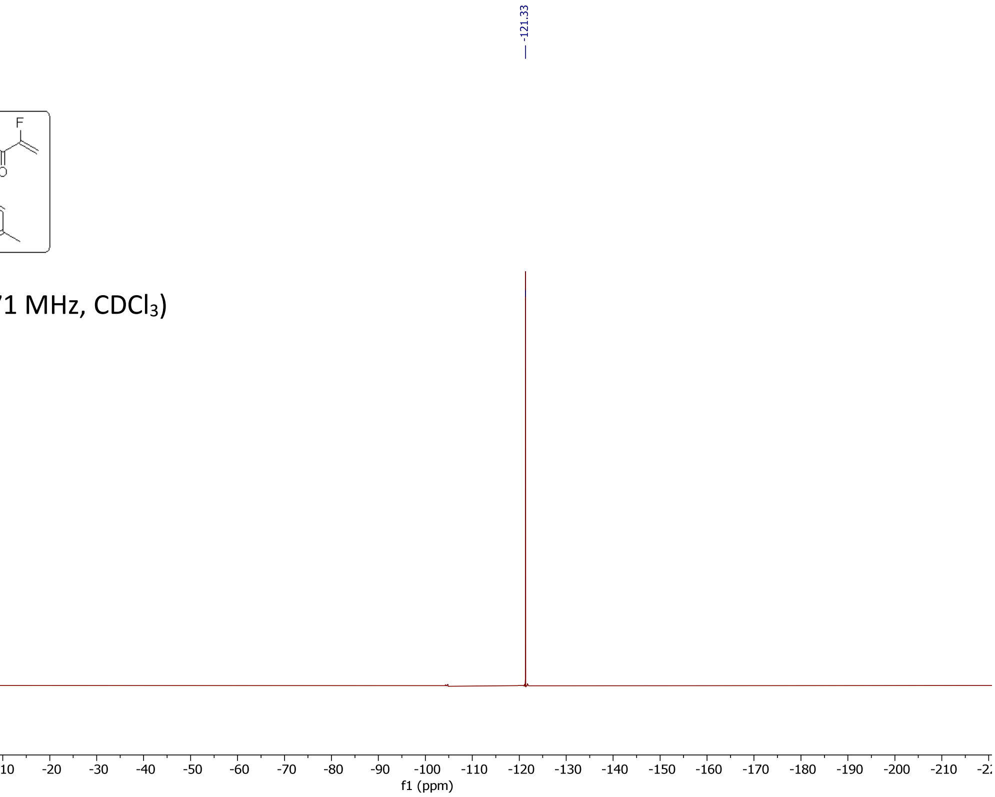

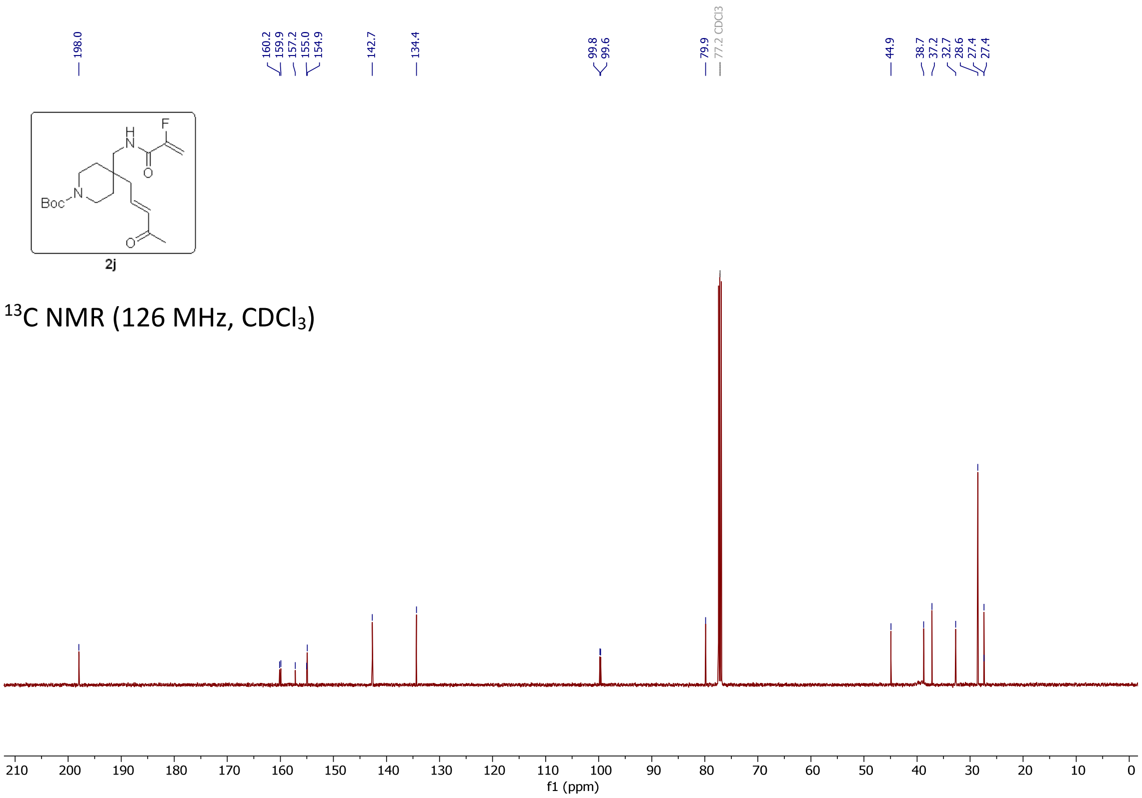

^{13}C NMR (126 MHz, $CDCl_3$)

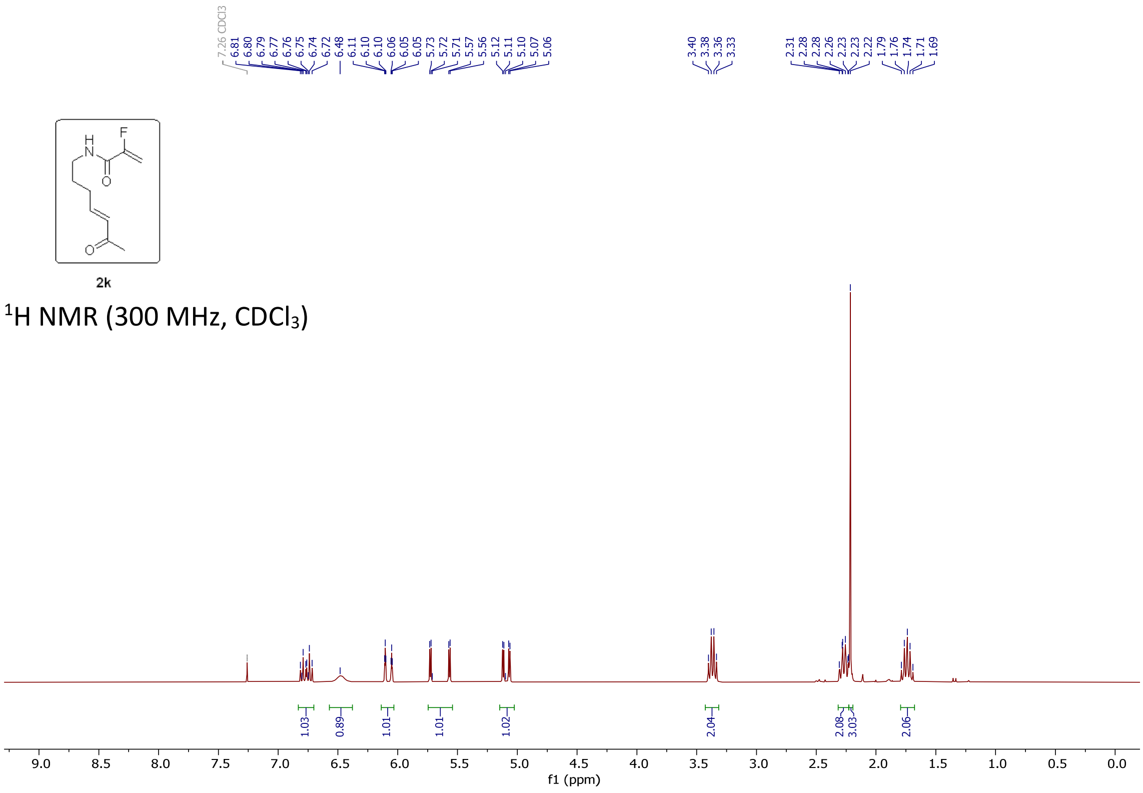

^{1}H NMR (300 MHz, $CDCl_3$)

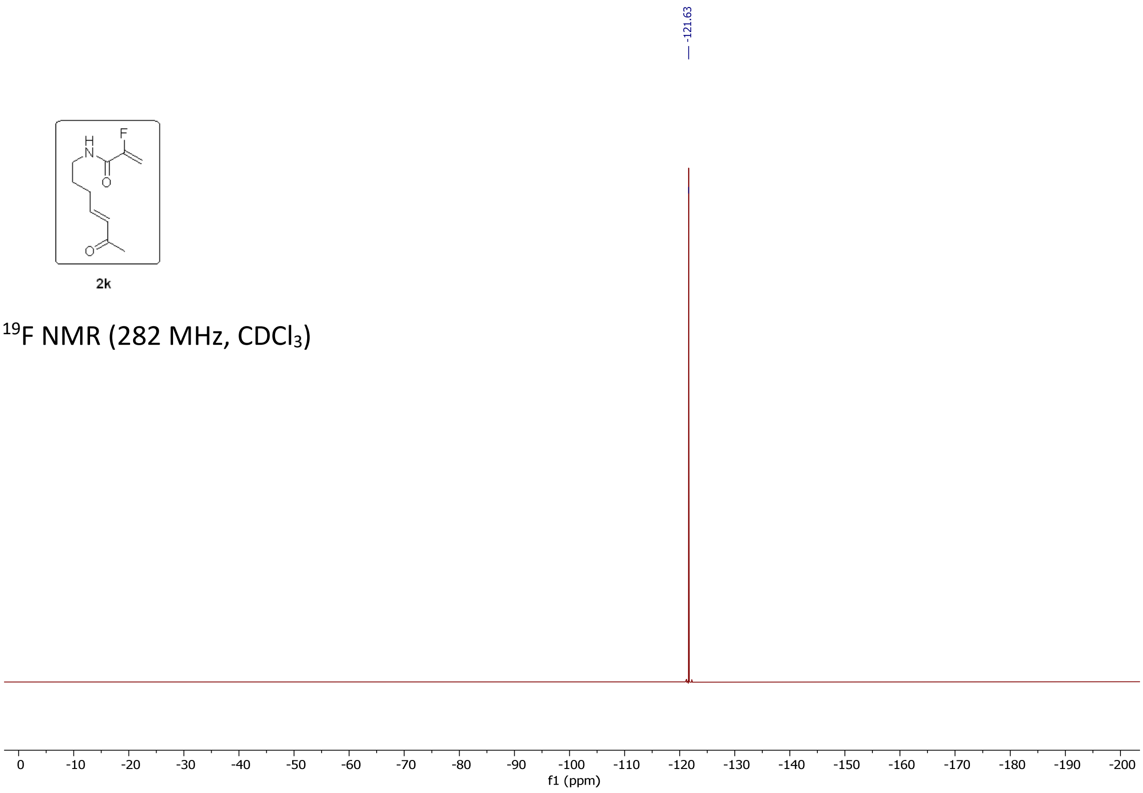

^{19}F NMR (282 MHz, $CDCl_3$)

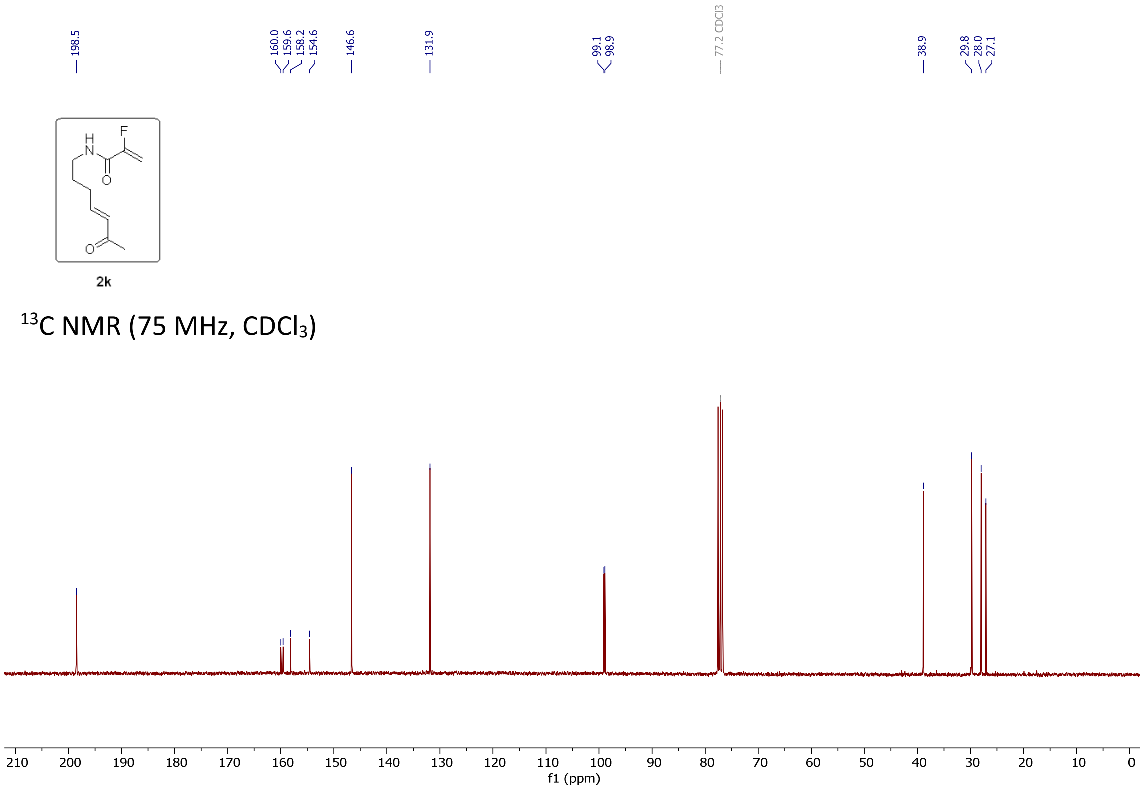

^{13}C NMR (75 MHz, $CDCl_3$)

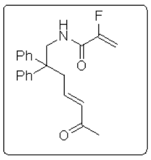

2l

^{1}H NMR (300 MHz, $CDCl_3$)

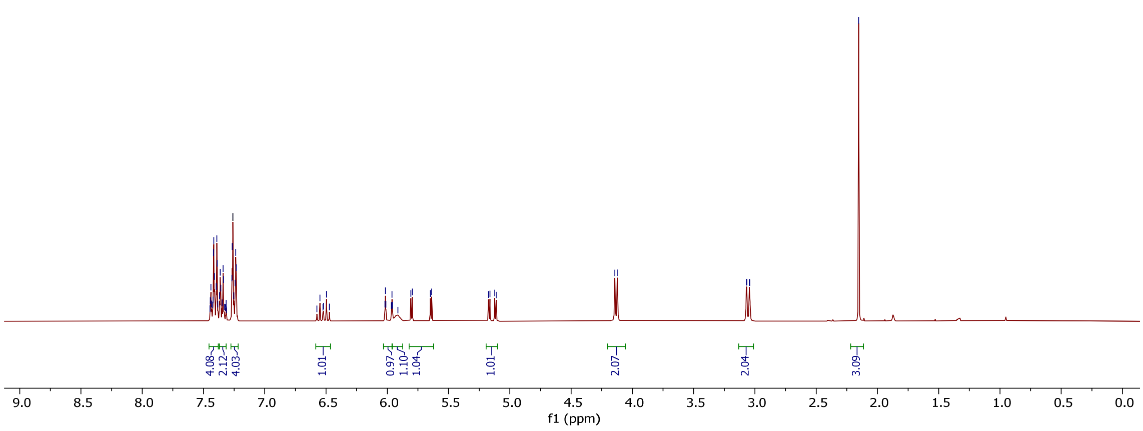

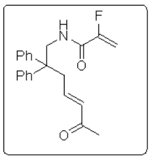

2l

^{19}F NMR (282 MHz, $CDCl_3$)

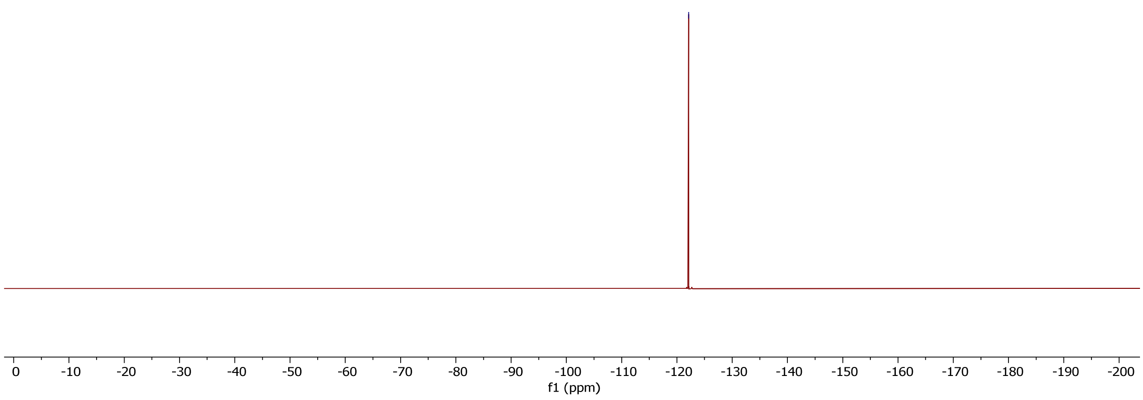

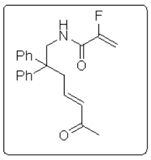

2l

^{13}C NMR (75 MHz, $CDCl_3$)

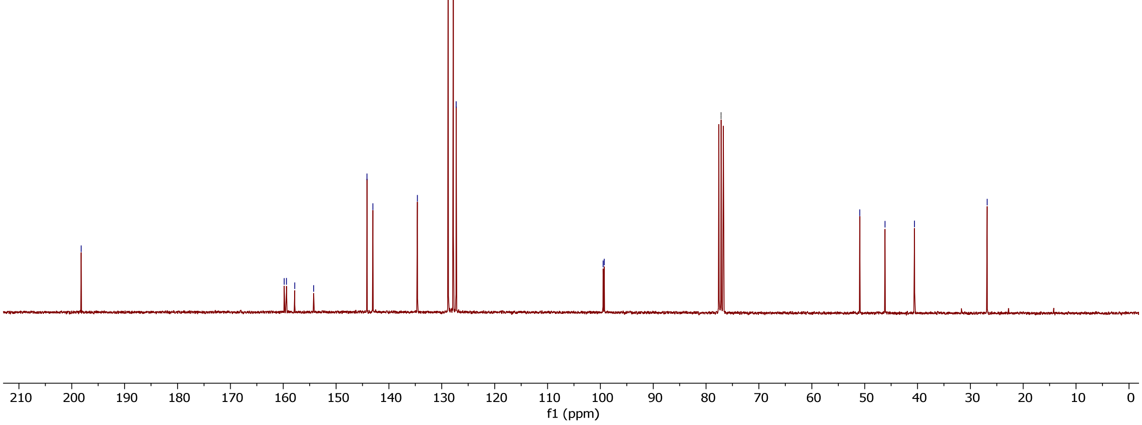

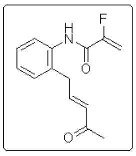

2m

^{1}H NMR (500 MHz, $CDCl_3$)

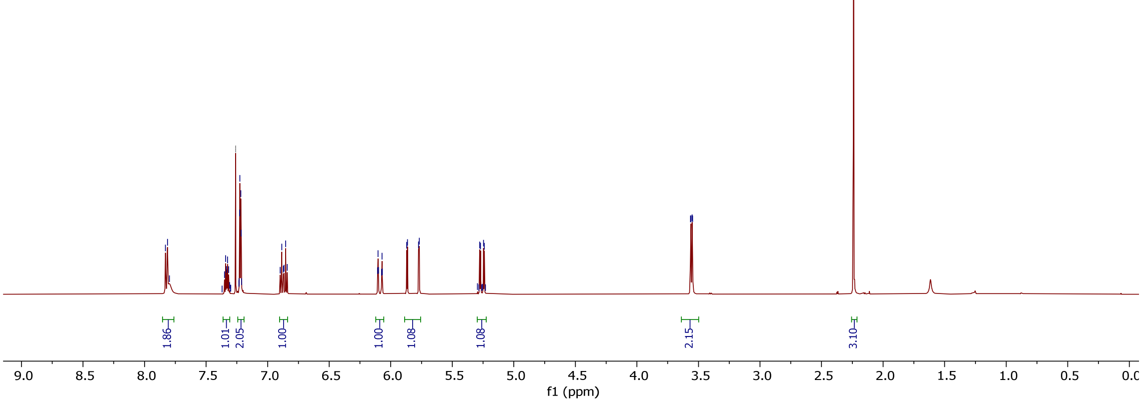

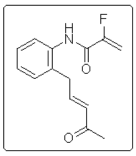

2m

^{19}F NMR (471 MHz, $CDCl_3$)

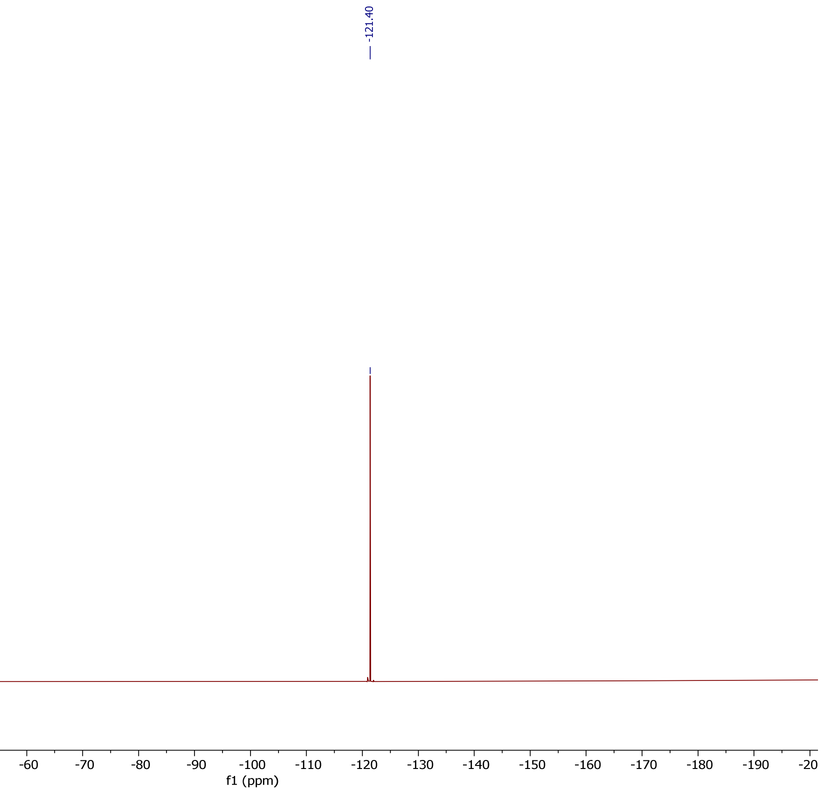

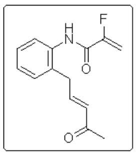

2m

^{13}C NMR (126 MHz, $CDCl_3$)

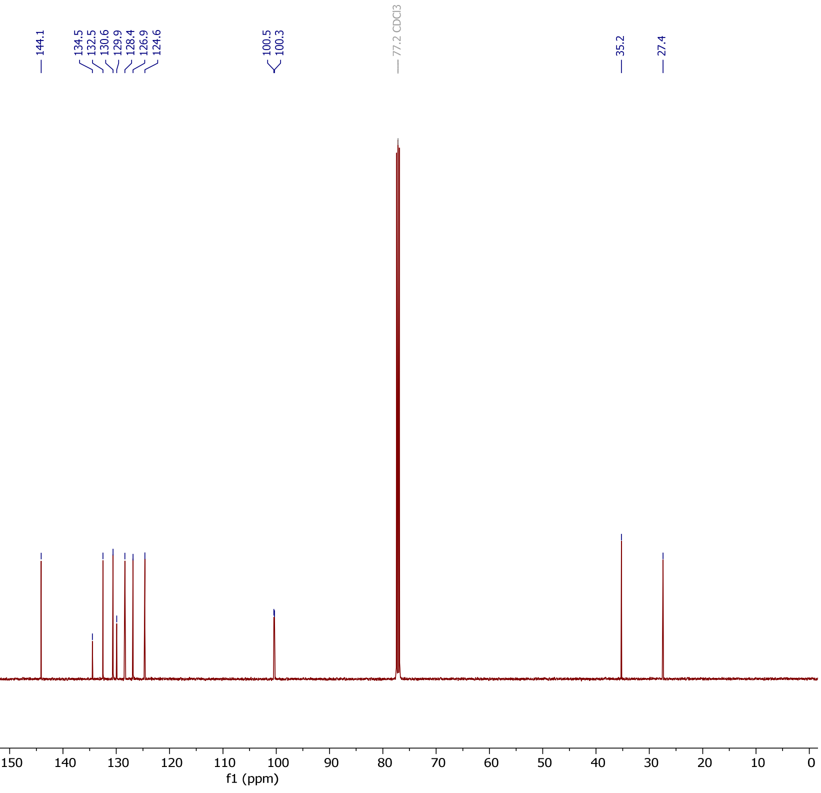

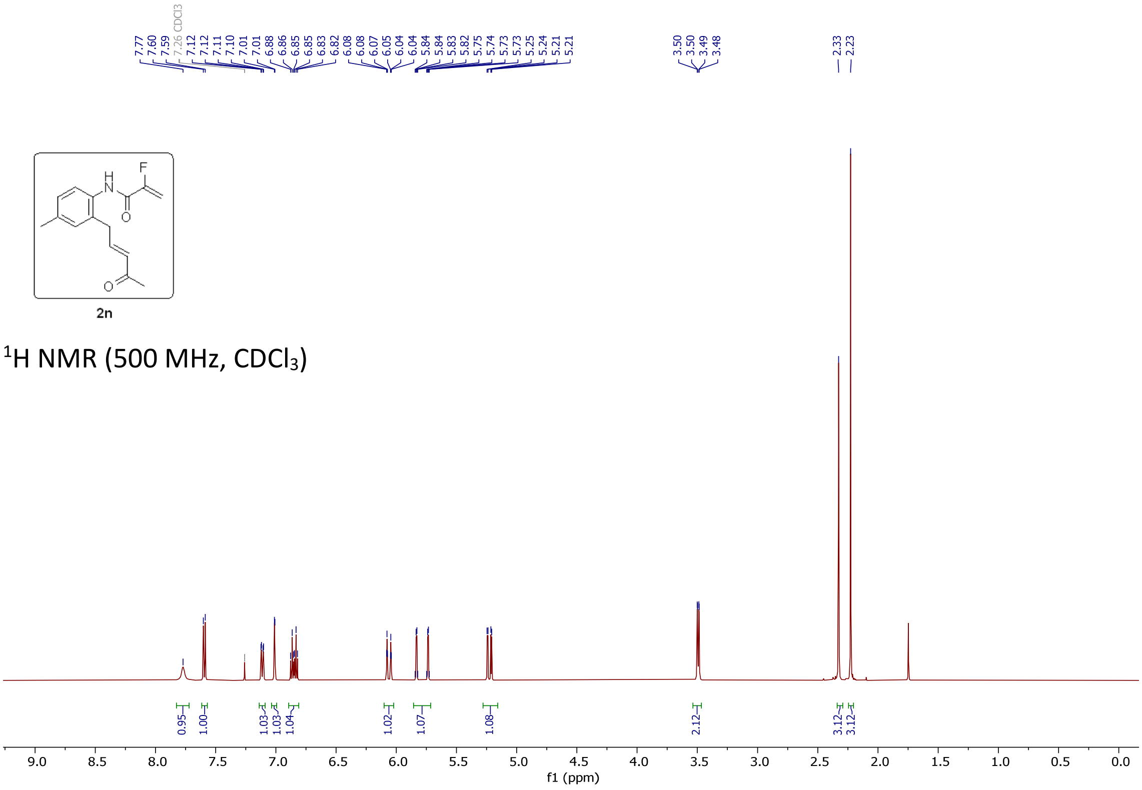

2n

^{1}H NMR (500 MHz, $CDCl_3$)

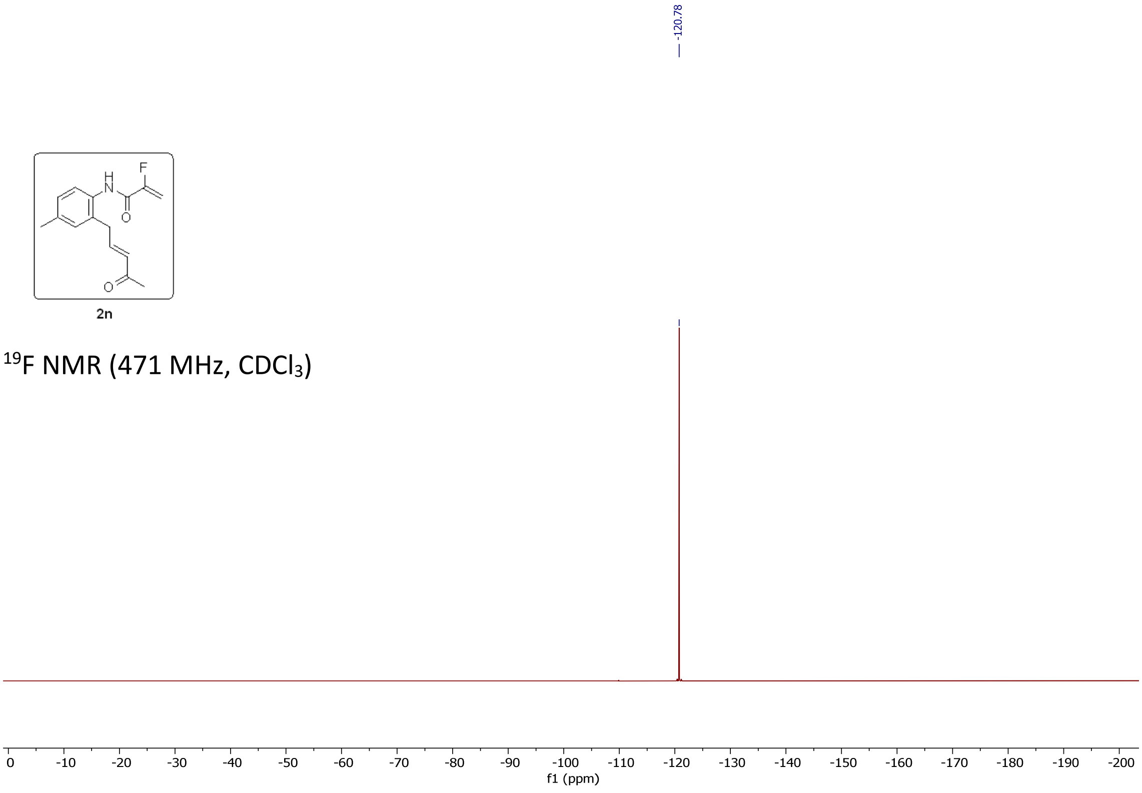

2n

^{19}F NMR (471 MHz, $CDCl_3$)

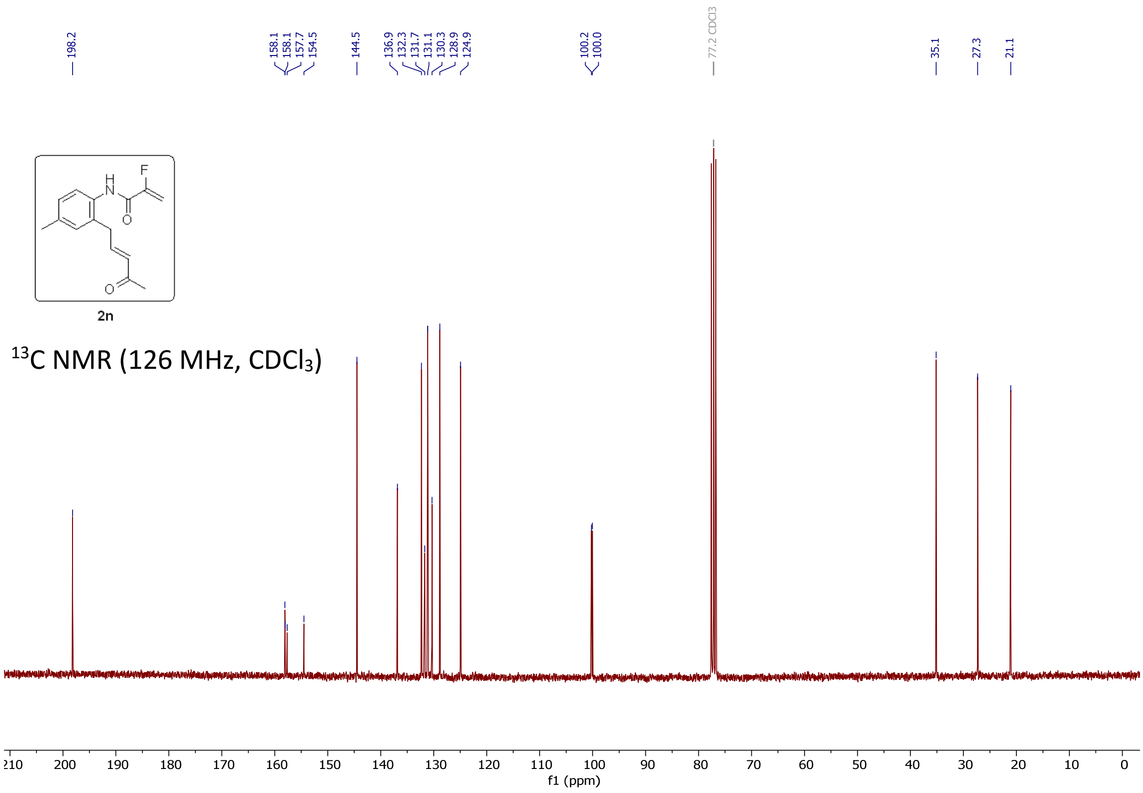

2n

^{13}C NMR (126 MHz, $CDCl_3$)

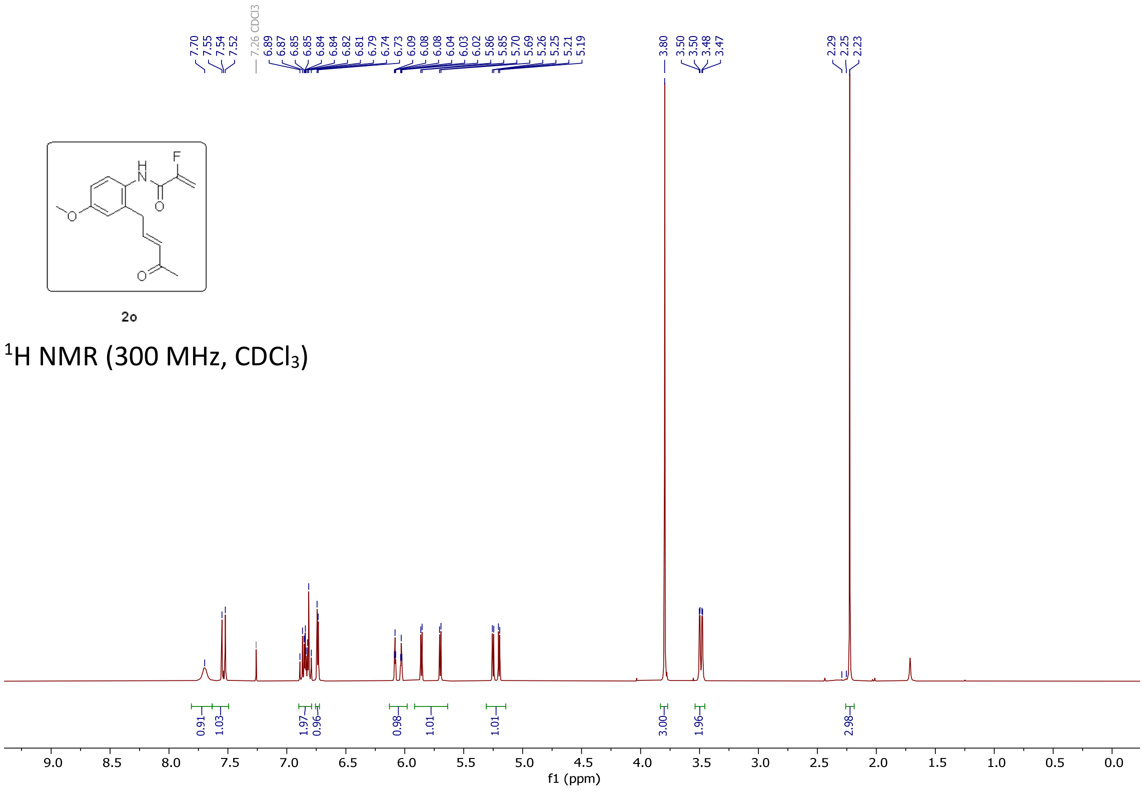

2o

^{1}H NMR (300 MHz, $CDCl_3$)

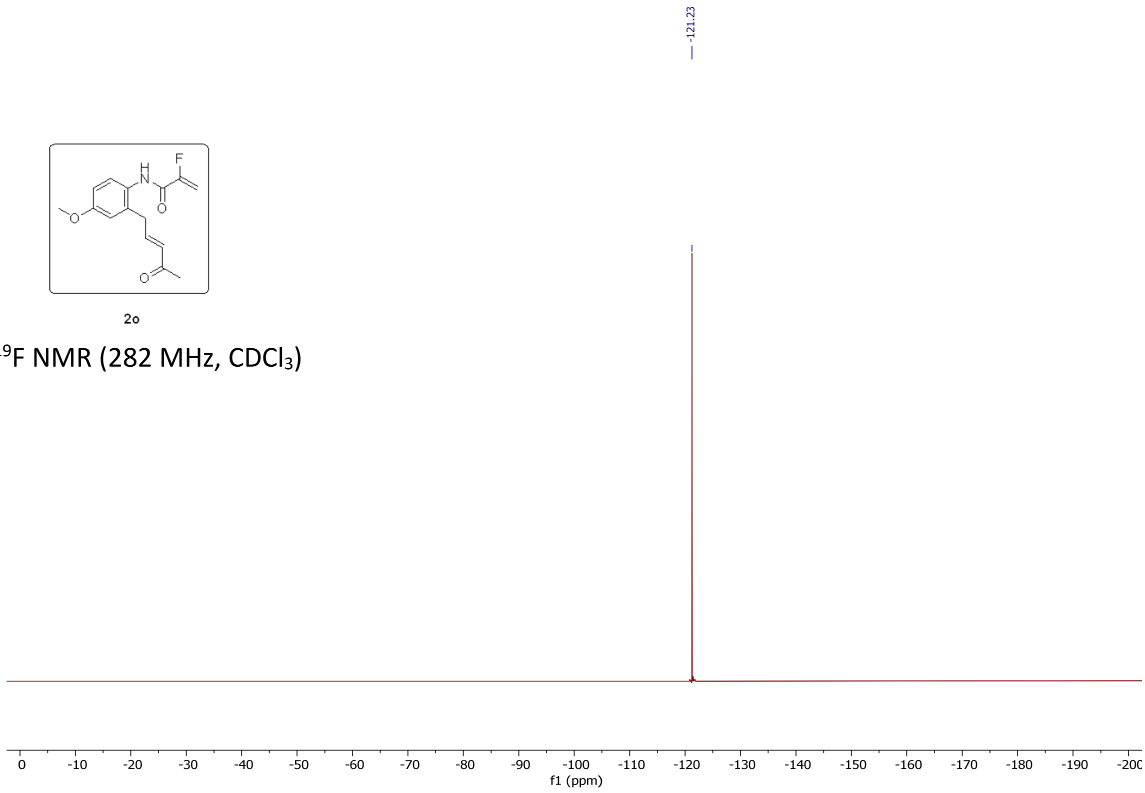

^{19}F NMR (282 MHz, $CDCl_3$)

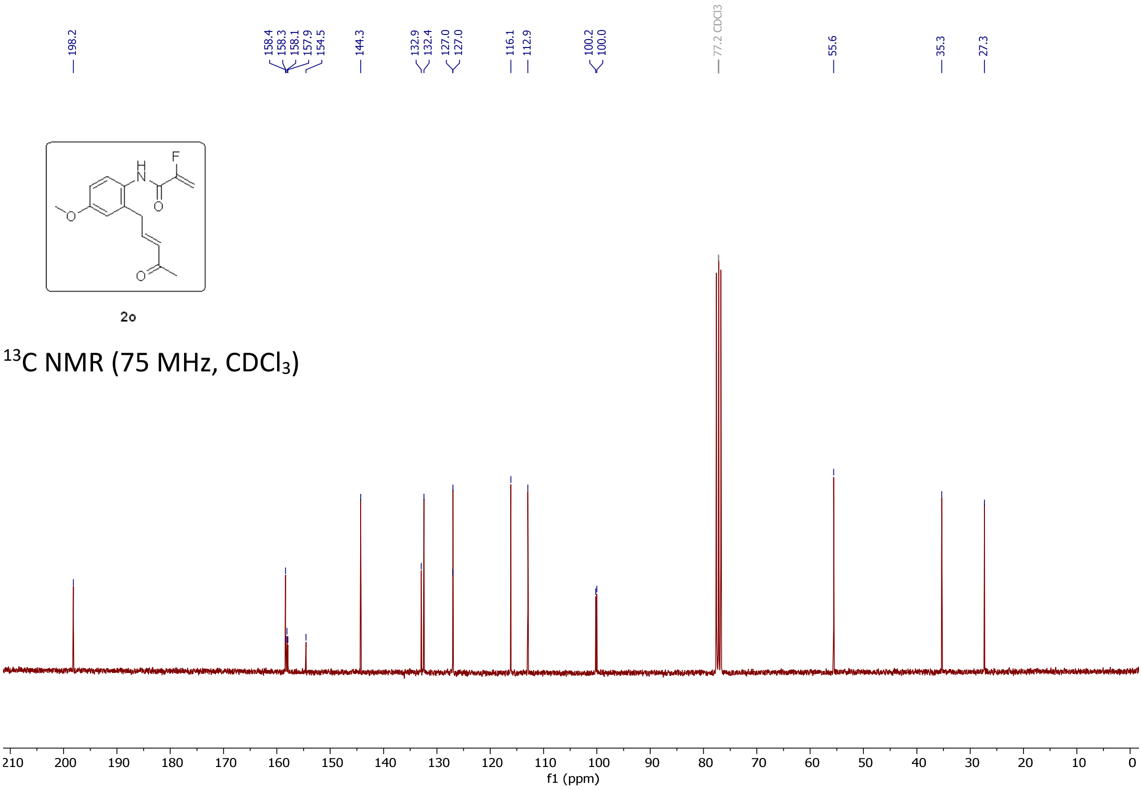

^{13}C NMR (75 MHz, $CDCl_3$)

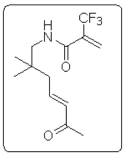
2p

^{1}H NMR (300 MHz, $CDCl_3$)

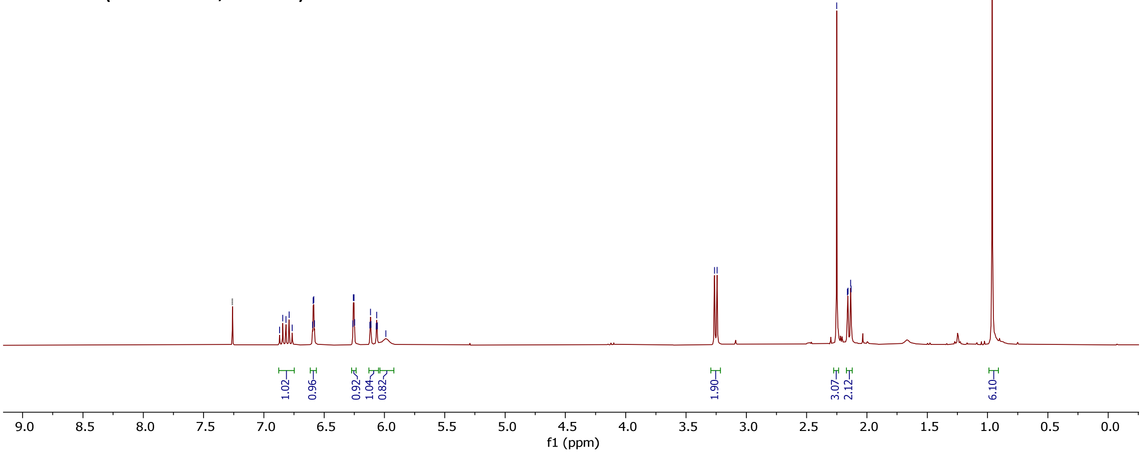

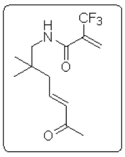
2p

^{19}F NMR (282 MHz, $CDCl_3$)

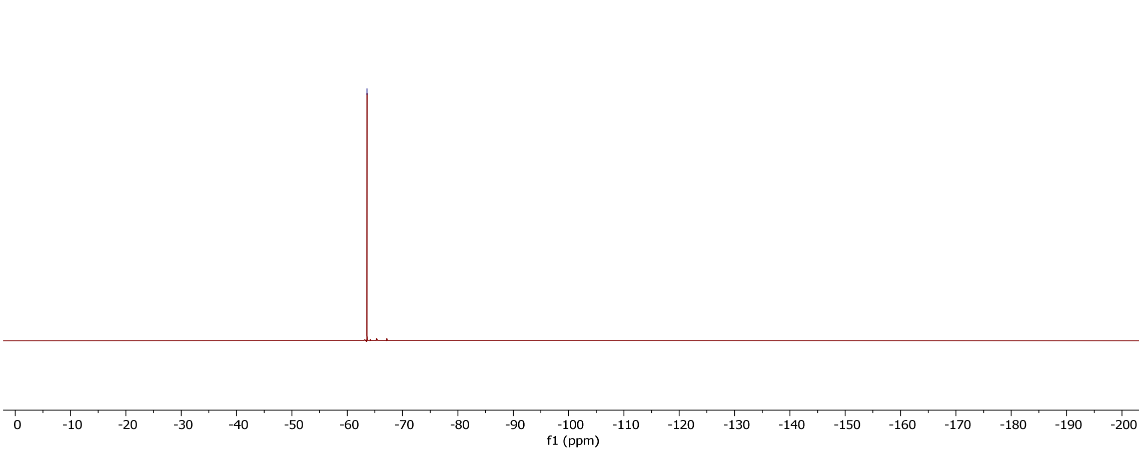

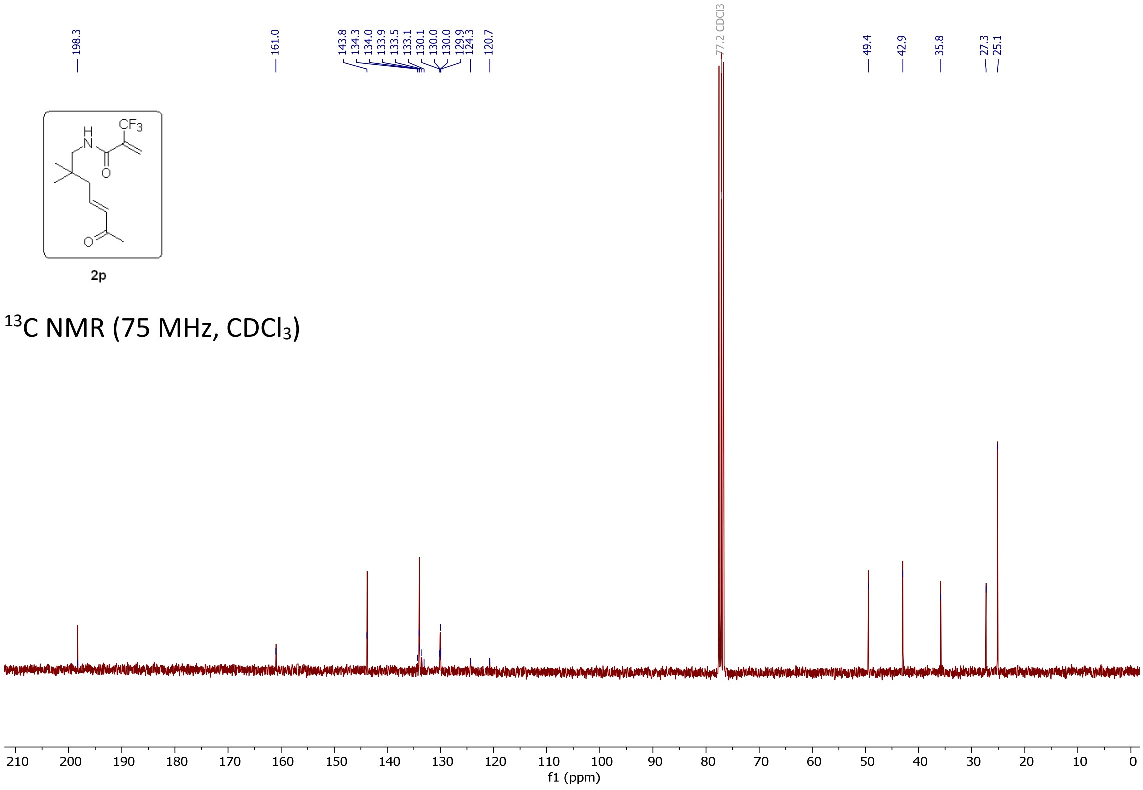

2p

^{13}C NMR (75 MHz, $CDCl_3$)

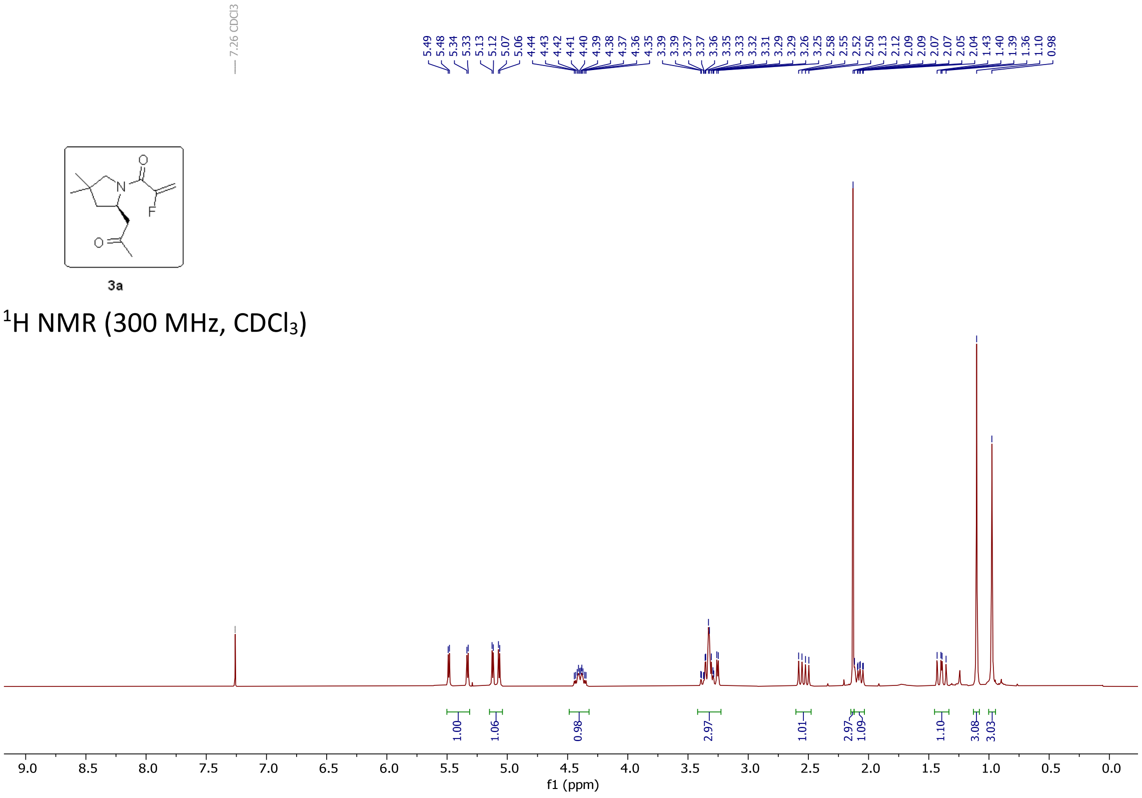

3a

^{1}H NMR (300 MHz, $CDCl_3$)

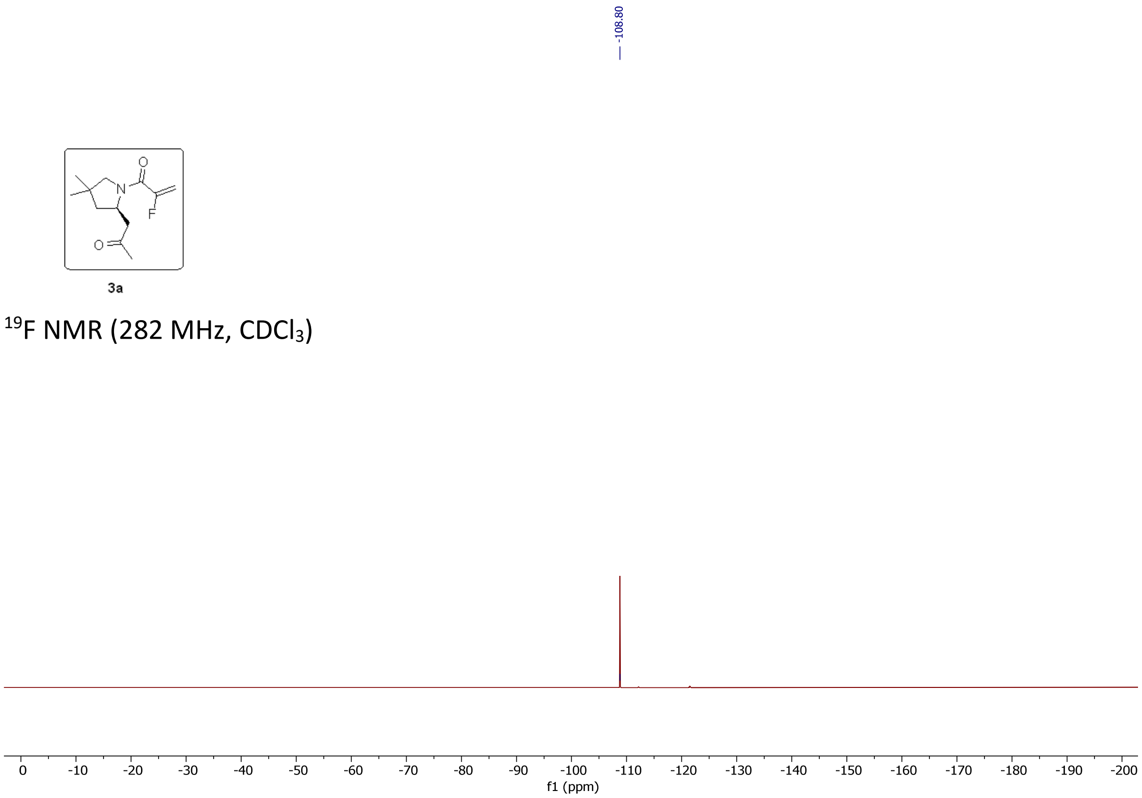

^{19}F NMR (282 MHz, $CDCl_3$)

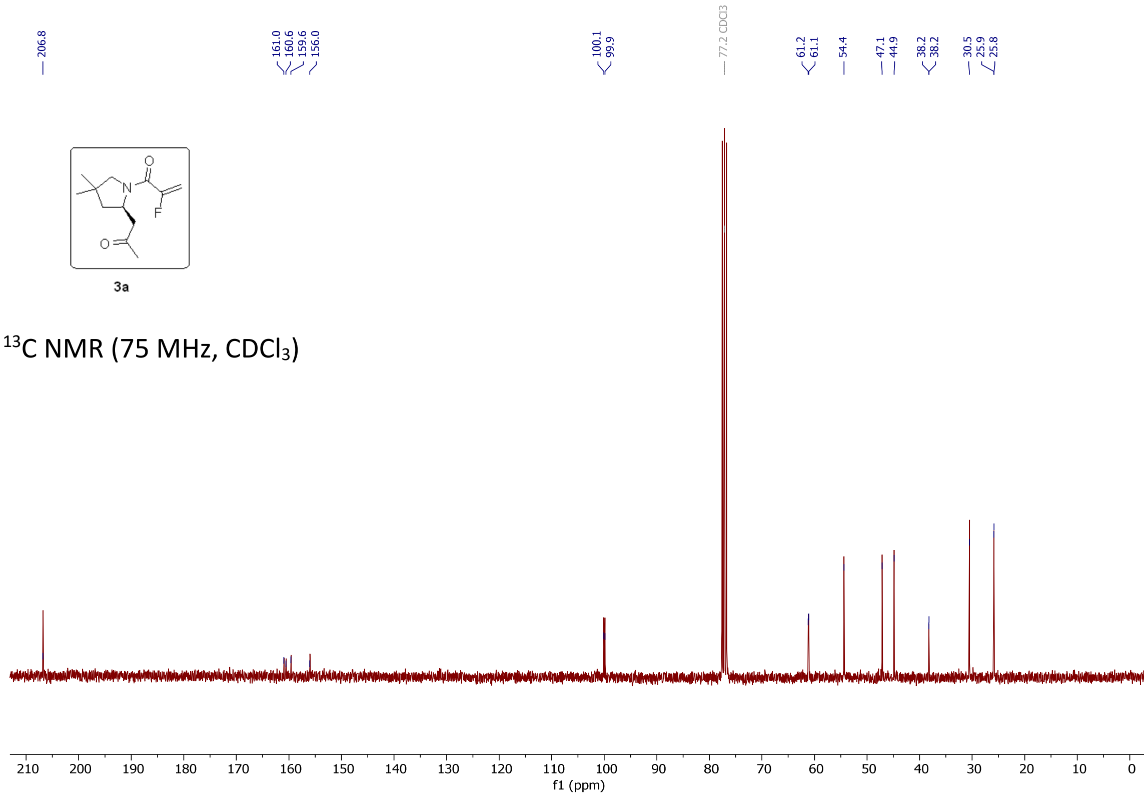

^{13}C NMR (75 MHz, $CDCl_3$)

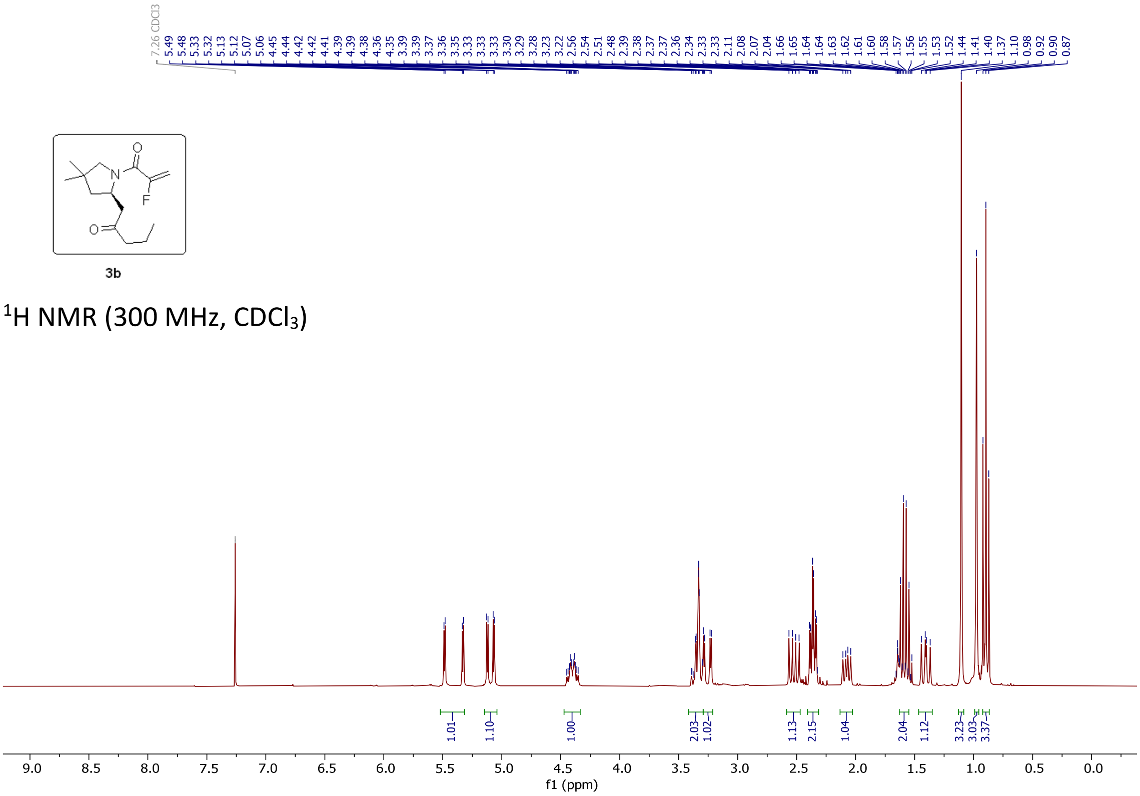

3b

^{1}H NMR (300 MHz, $CDCl_3$)

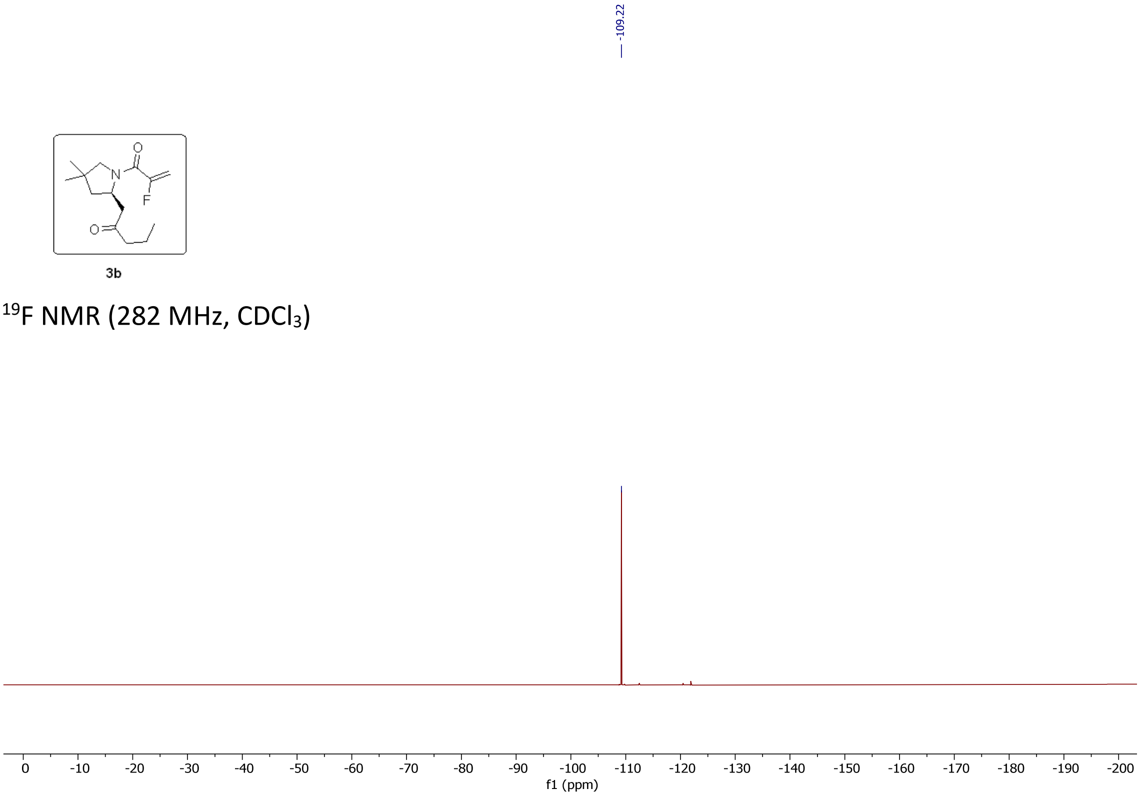

3b

^{19}F NMR (282 MHz, $CDCl_3$)

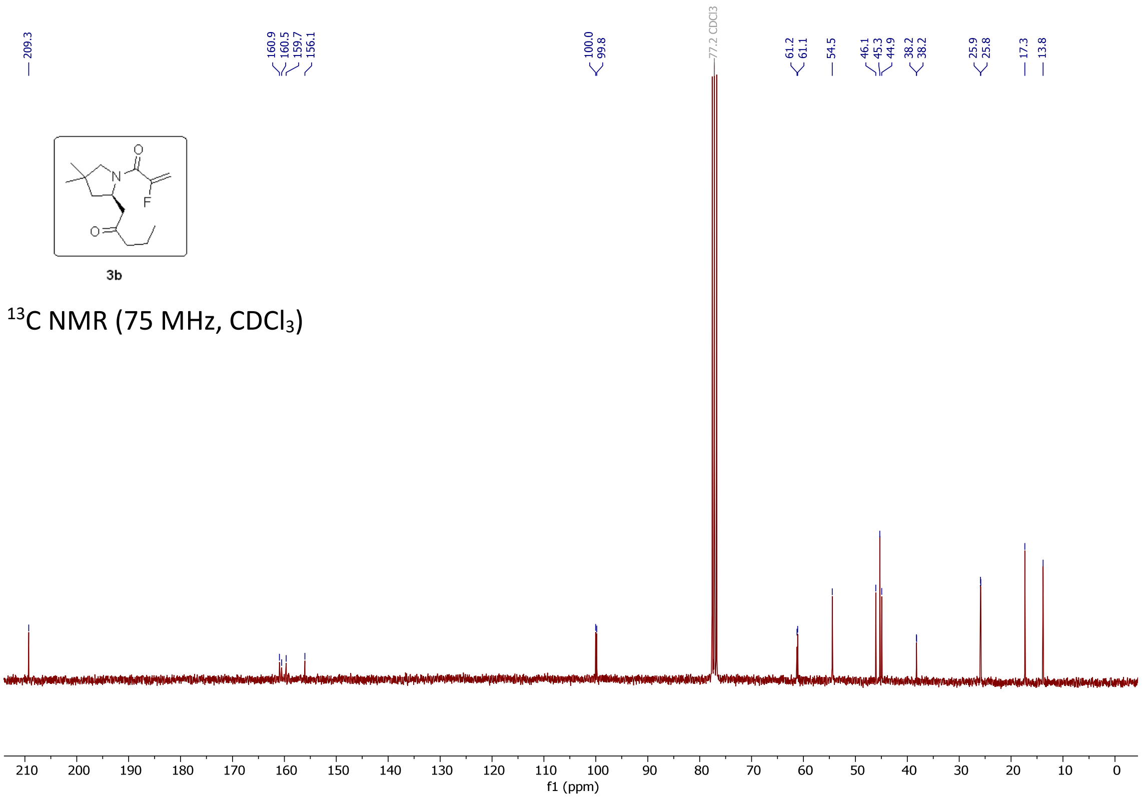

^{13}C NMR (75 MHz, $CDCl_3$)

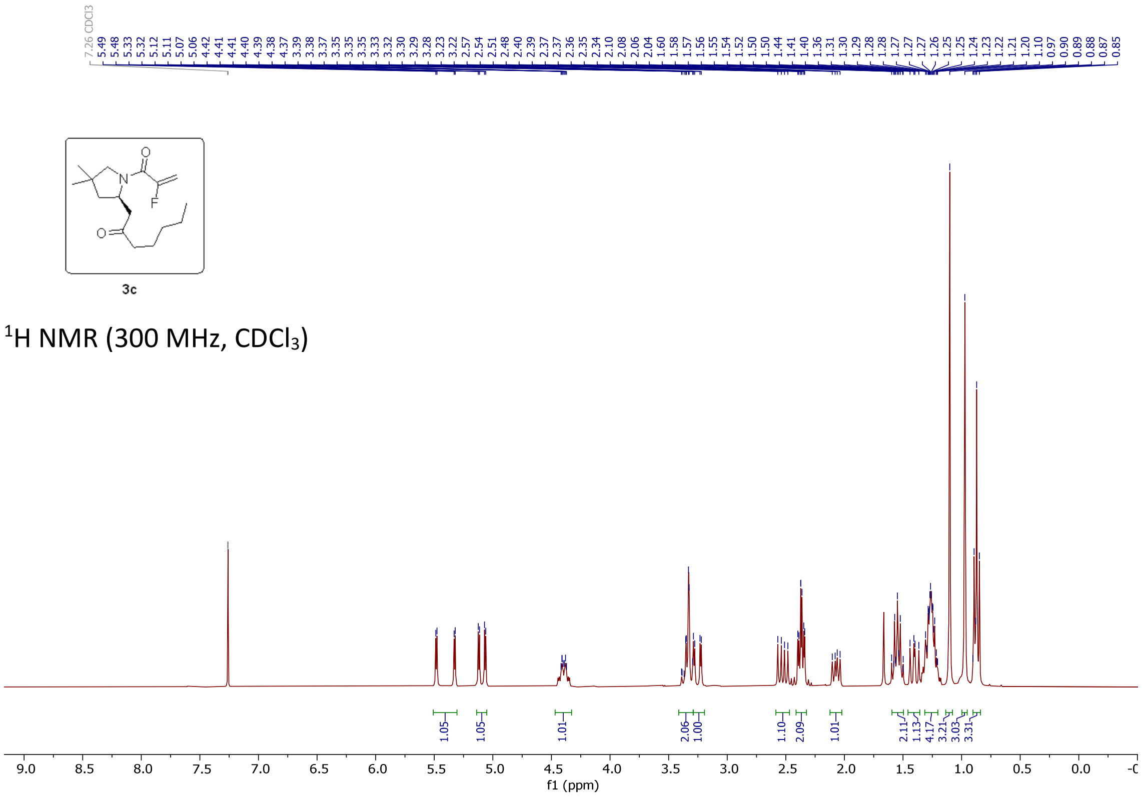

^{1}H NMR (300 MHz, $CDCl_3$)

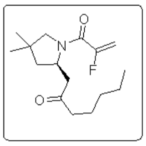

3c

^{19}F NMR (282 MHz, $CDCl_3$)

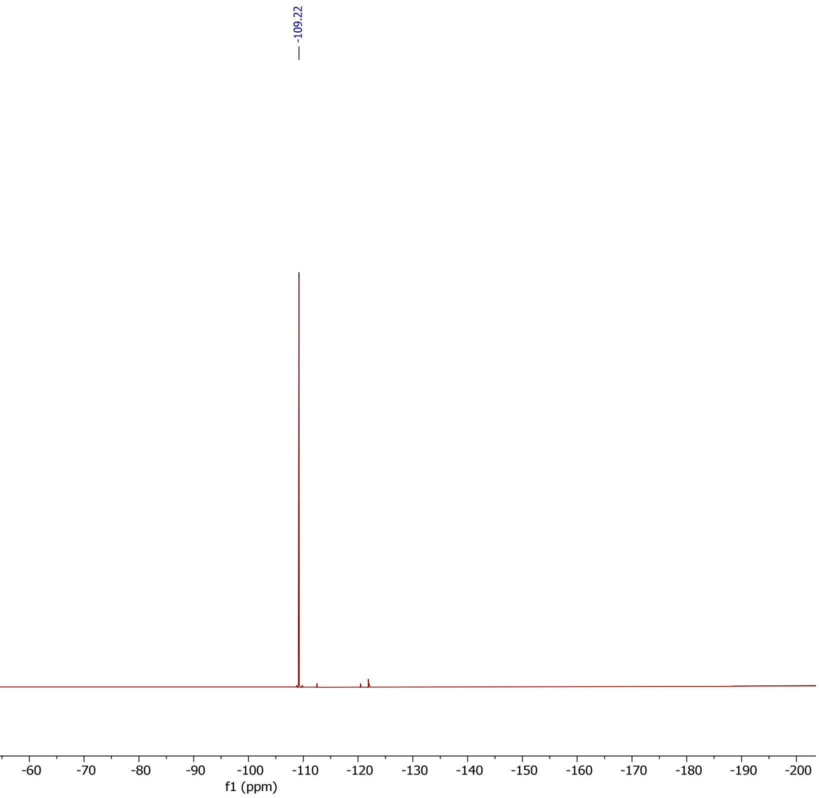

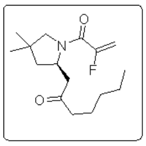

3c

^{13}C NMR (75 MHz, $CDCl_3$)

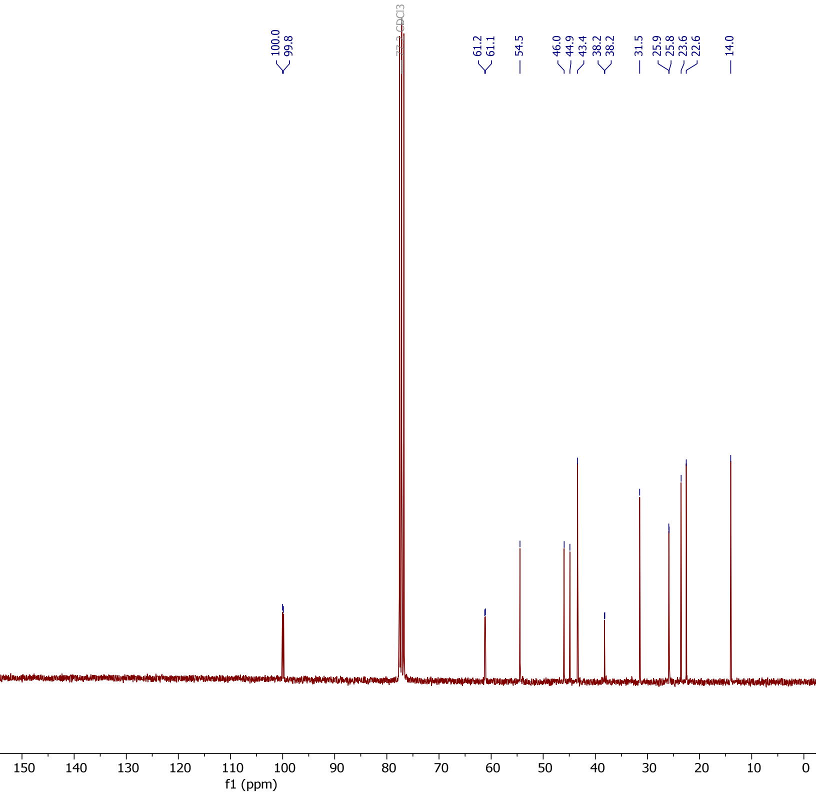

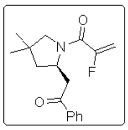

3d

^{1}H NMR (300 MHz, $CDCl_3$)

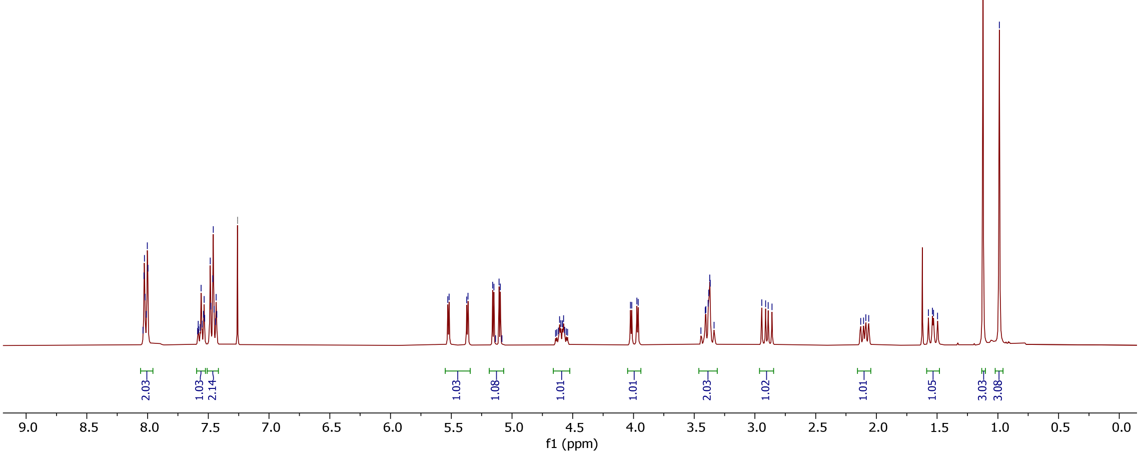

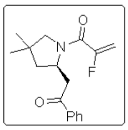

3d

^{19}F NMR (282 MHz, $CDCl_3$)

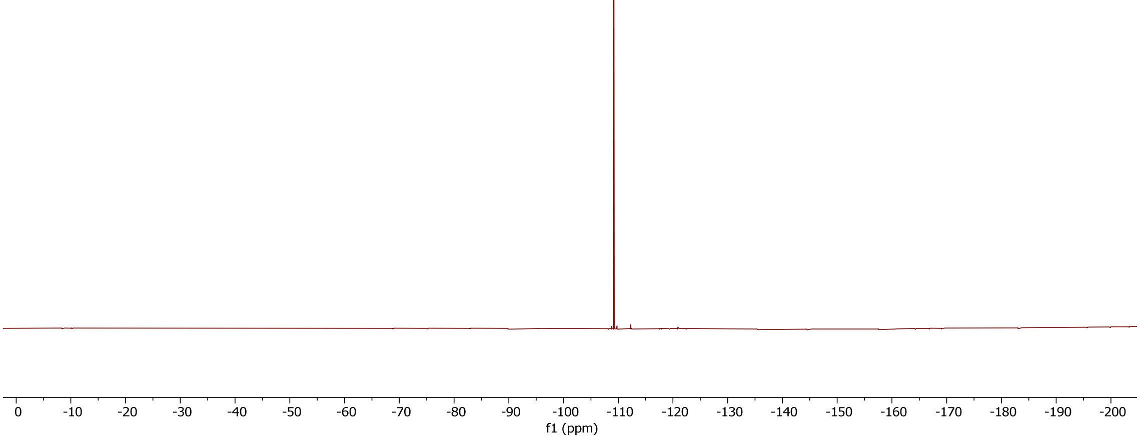

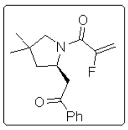

3d

^{13}C NMR (75 MHz, $CDCl_3$)

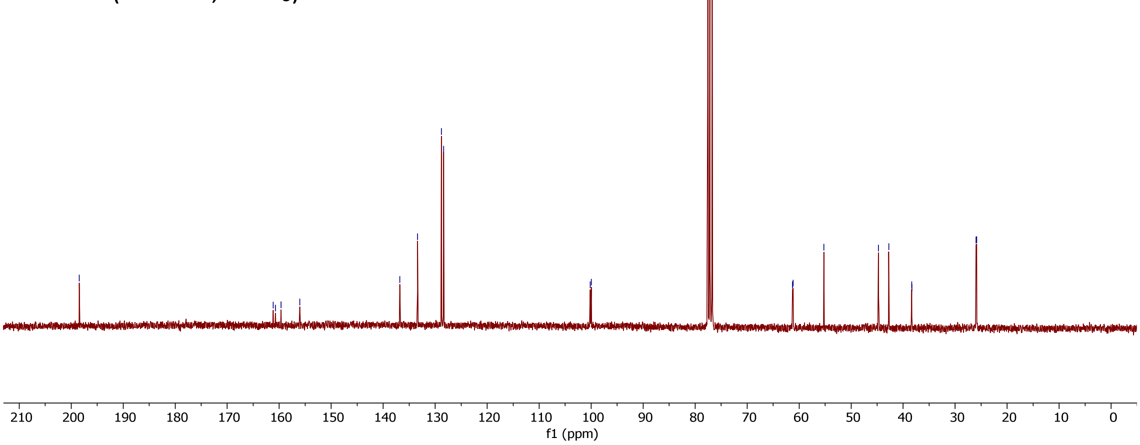

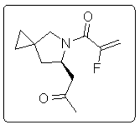

3e

^{1}H NMR (300 MHz, $CDCl_3$)

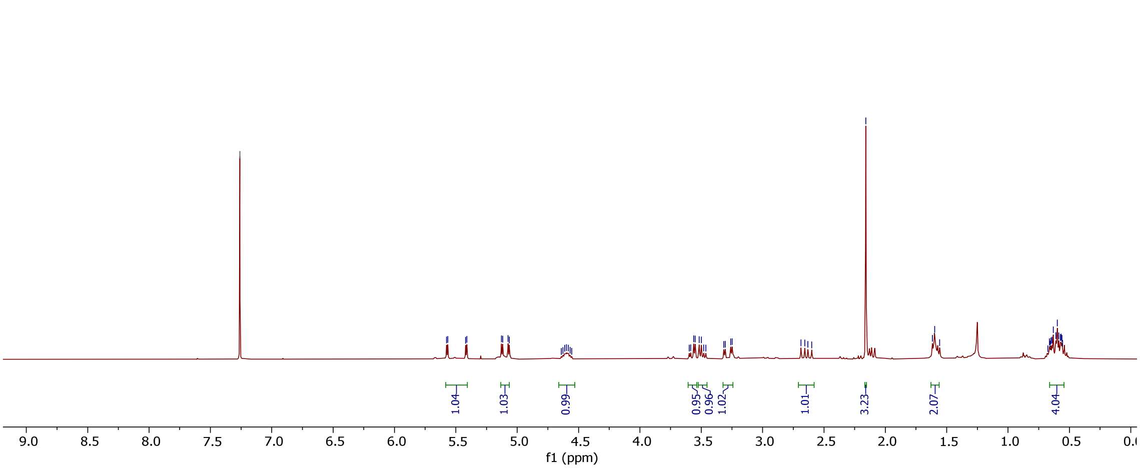

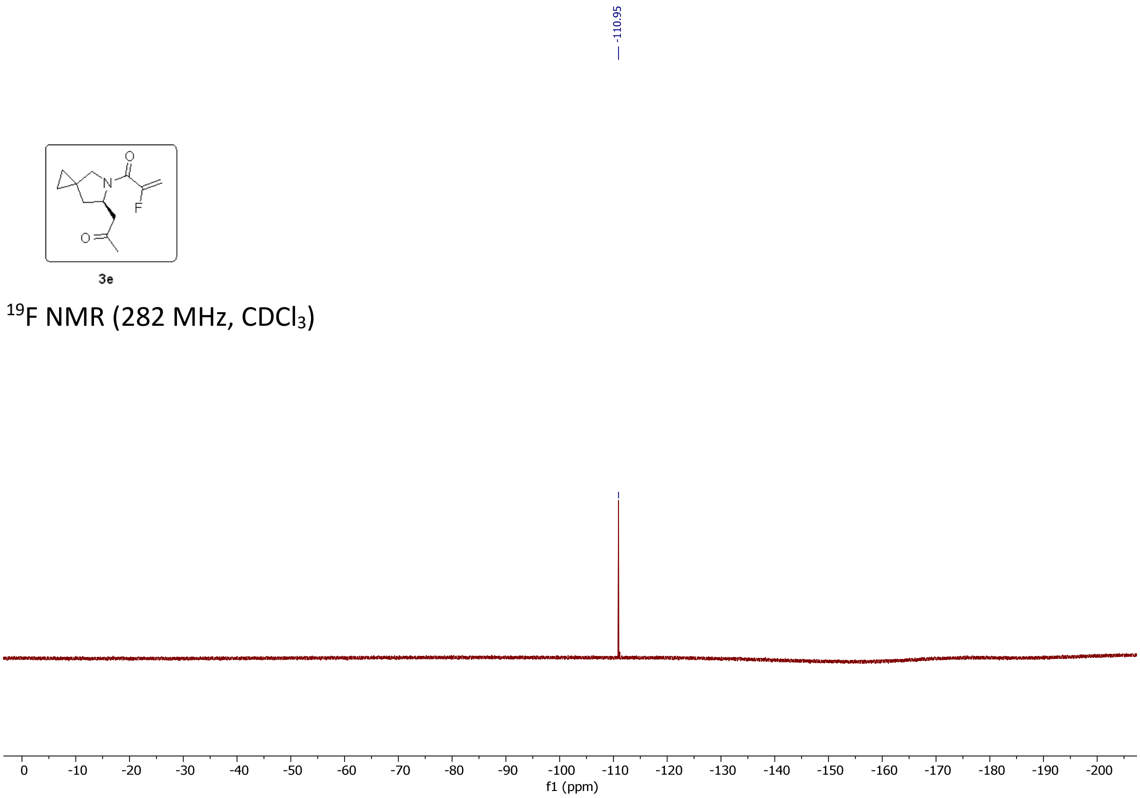

3e

^{19}F NMR (282 MHz, $CDCl_3$)

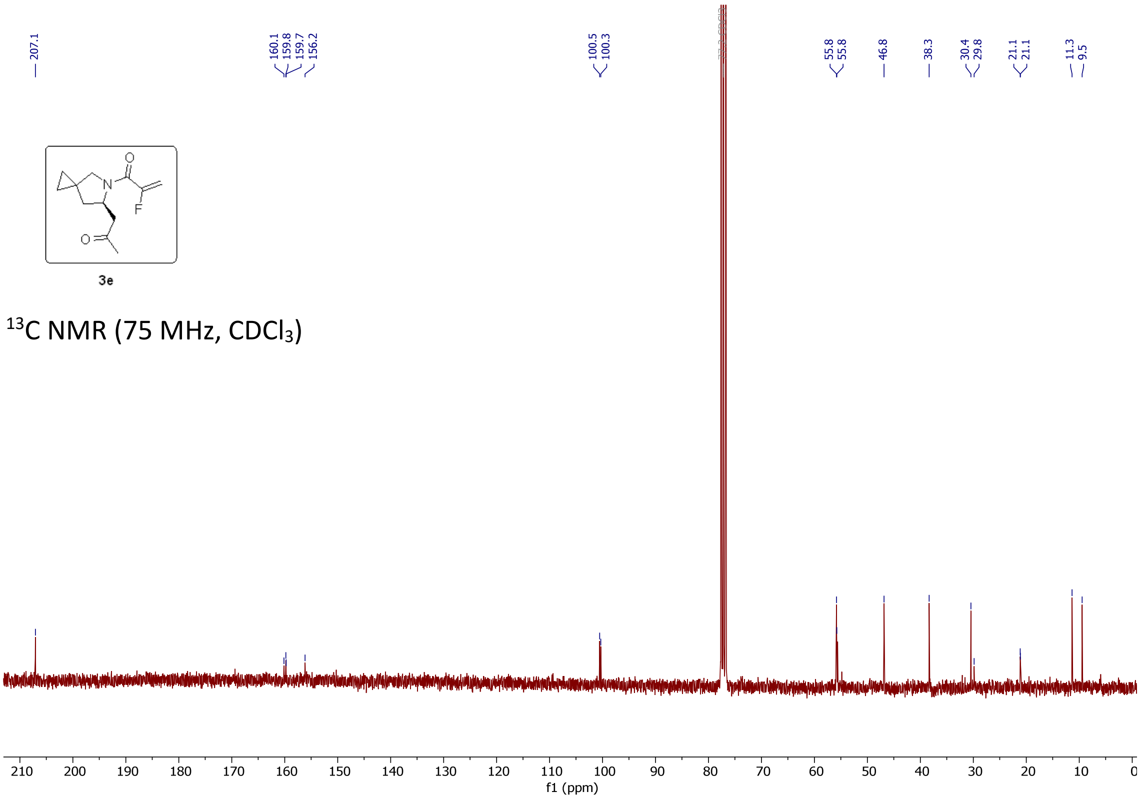

3e

^{13}C NMR (75 MHz, $CDCl_3$)

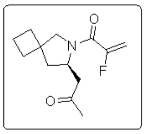

3f

^{1}H NMR (300 MHz, $CDCl_3$)

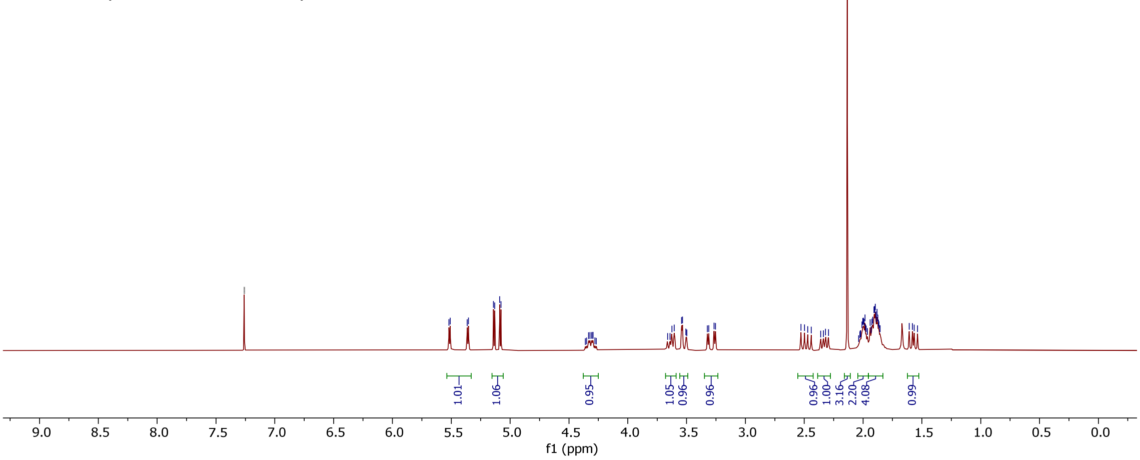

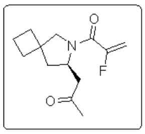

3f

^{19}F NMR (282 MHz, $CDCl_3$)

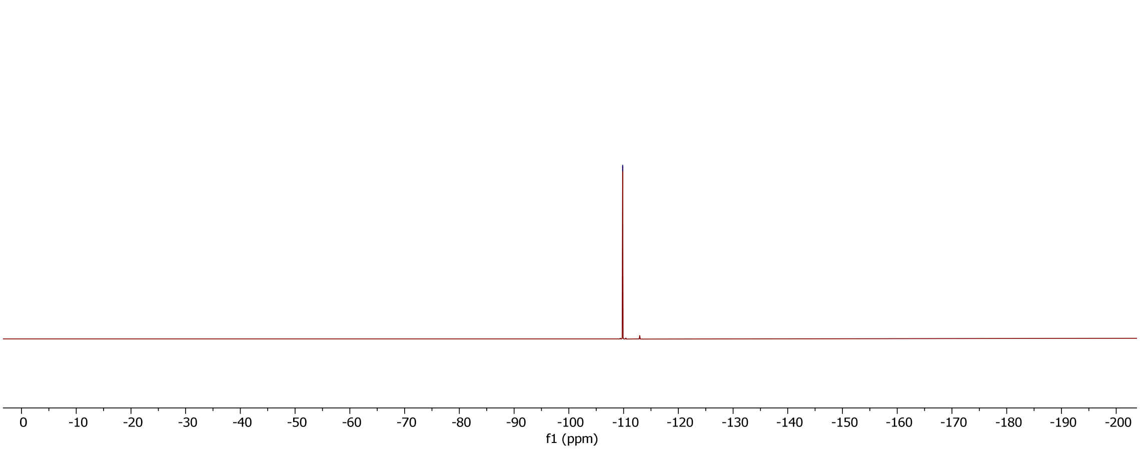

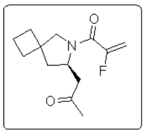

3f

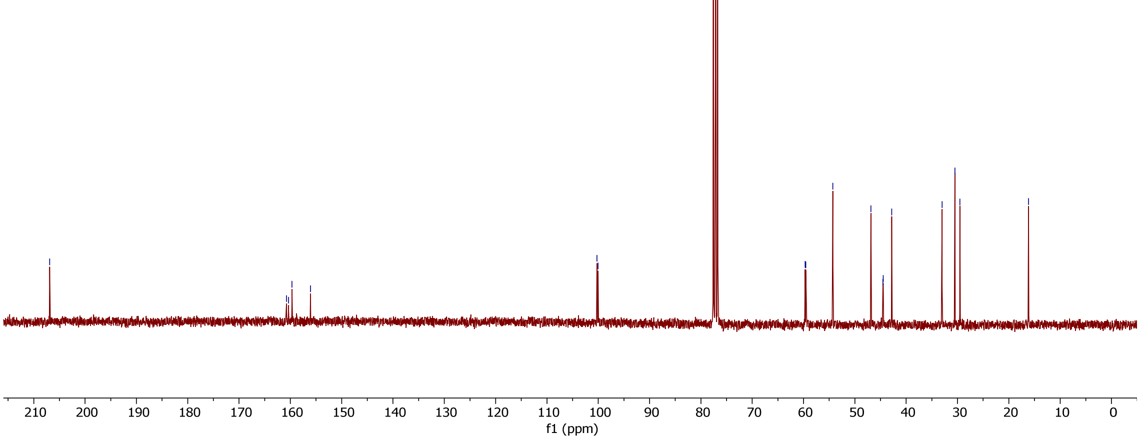

^{13}C NMR (75 MHz, $CDCl_3$)

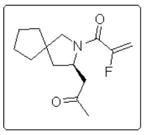

3g

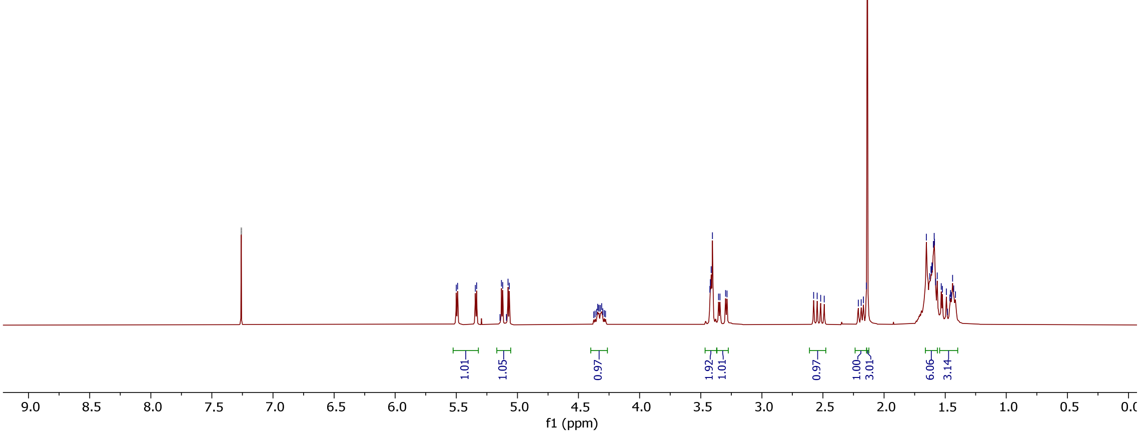

^{1}H NMR (300 MHz, $CDCl_3$)

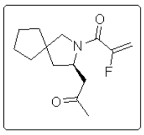

3g

^{19}F NMR (282 MHz, $CDCl_3$)

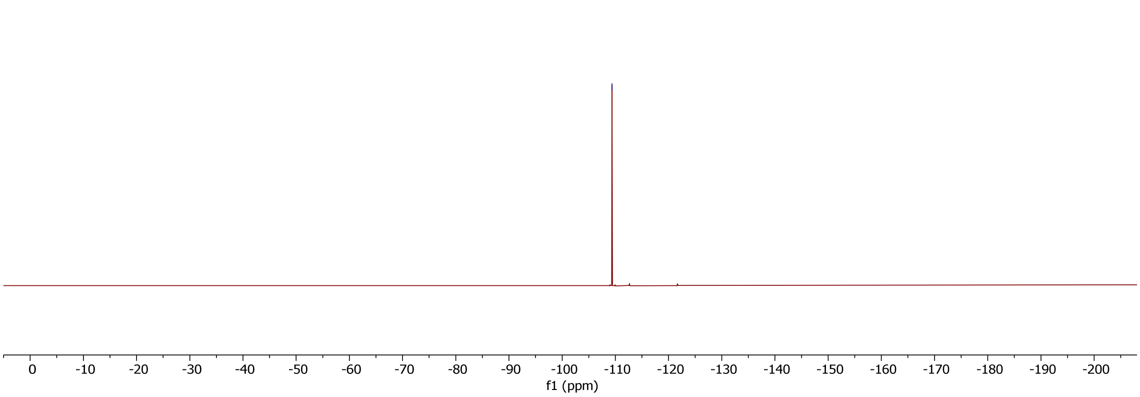

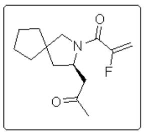

3g

^{13}C NMR (75 MHz, $CDCl_3$)

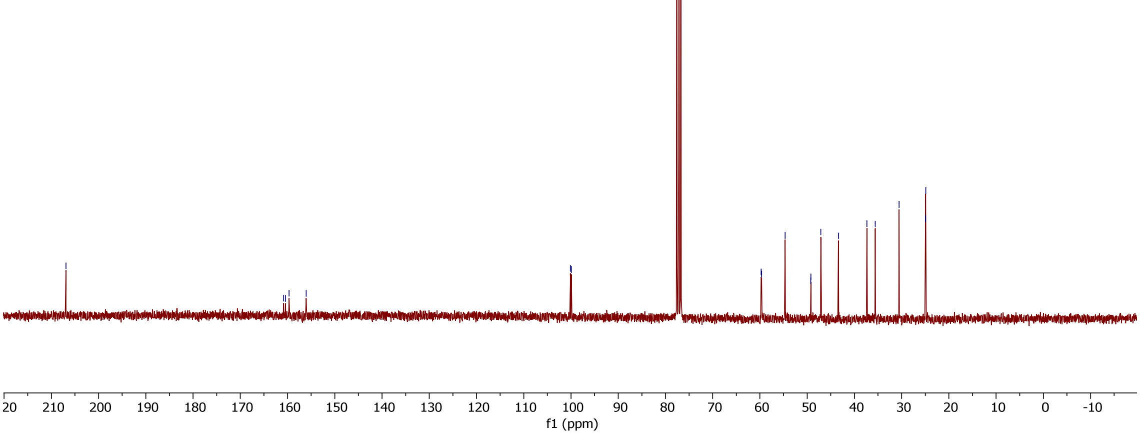

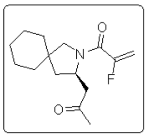

3h

¹H NMR (300 MHz, $CDCl_3$)

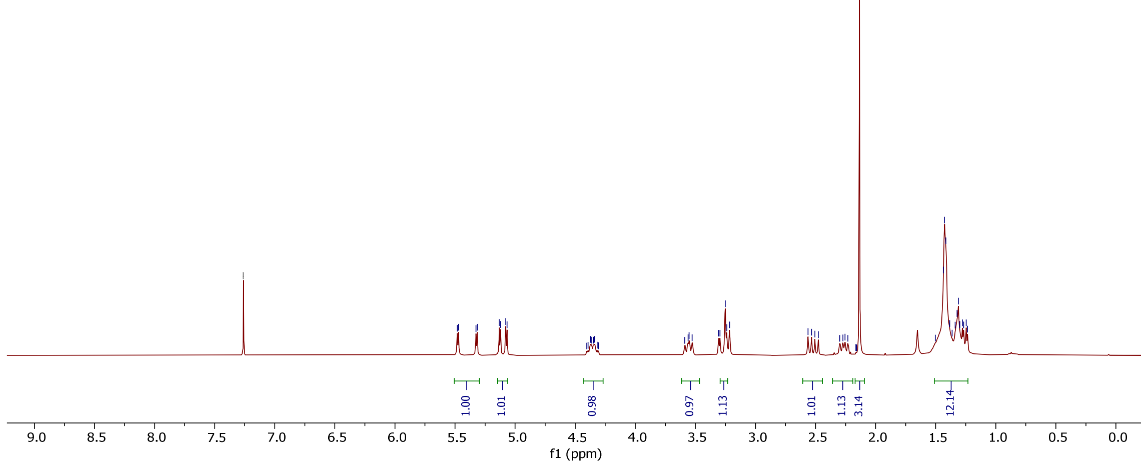

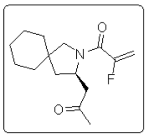

3h

¹⁹F NMR (282 MHz, $CDCl_3$)

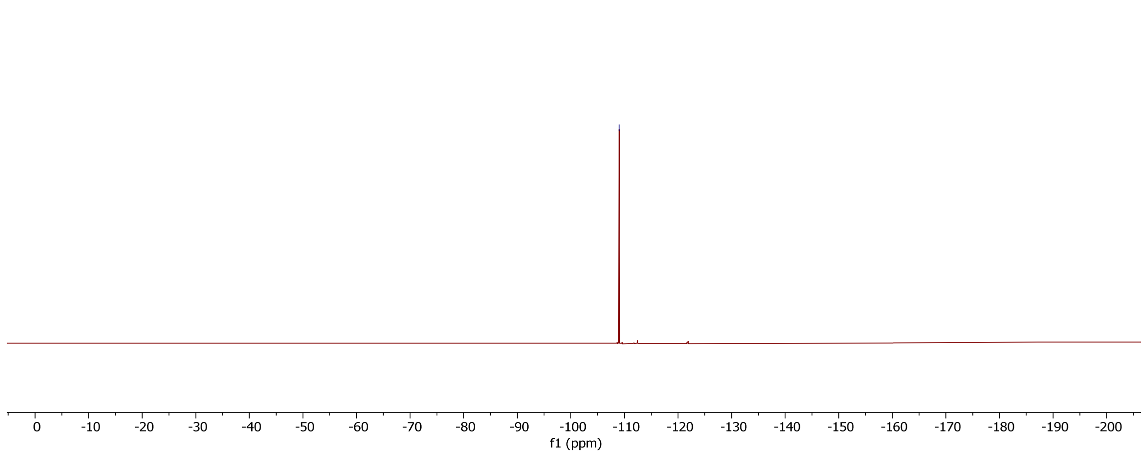

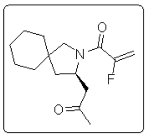

3h

^{13}C NMR (75 MHz, $CDCl_3$)

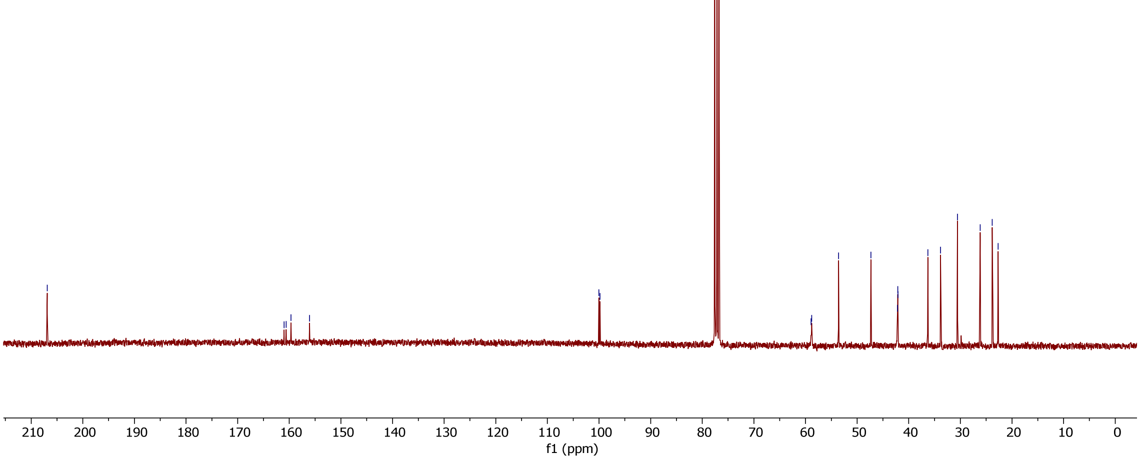

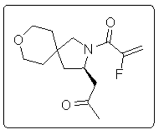

3i

^{1}H NMR (300 MHz, $CDCl_3$)

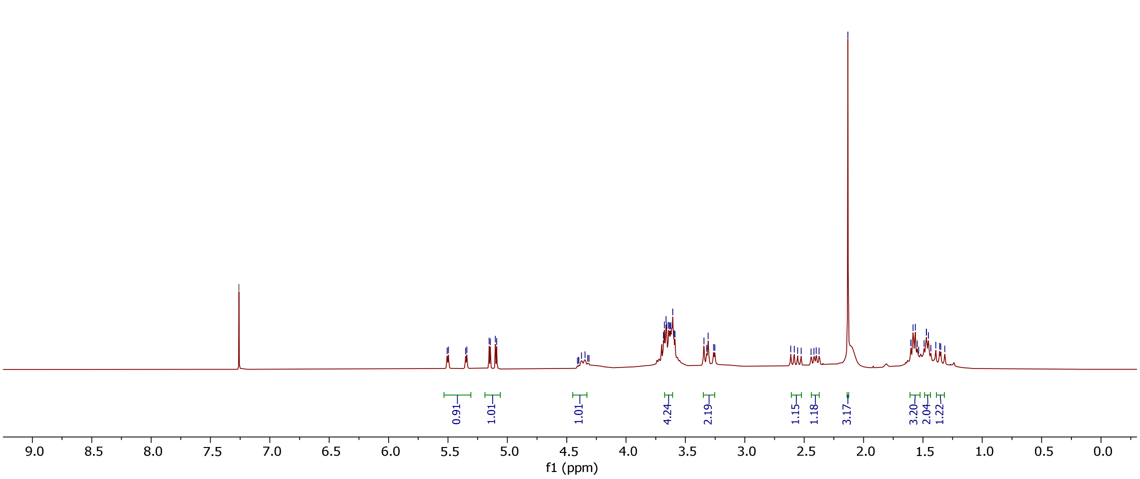

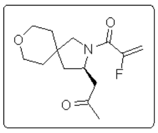

3i

^{19}F NMR (282 MHz, $CDCl_3$)

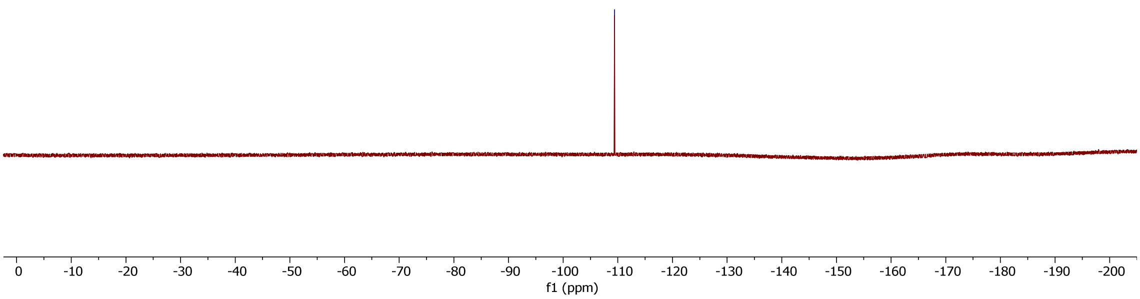

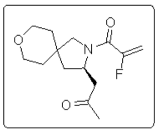

3i

^{13}C NMR (75 MHz, $CDCl_3$)

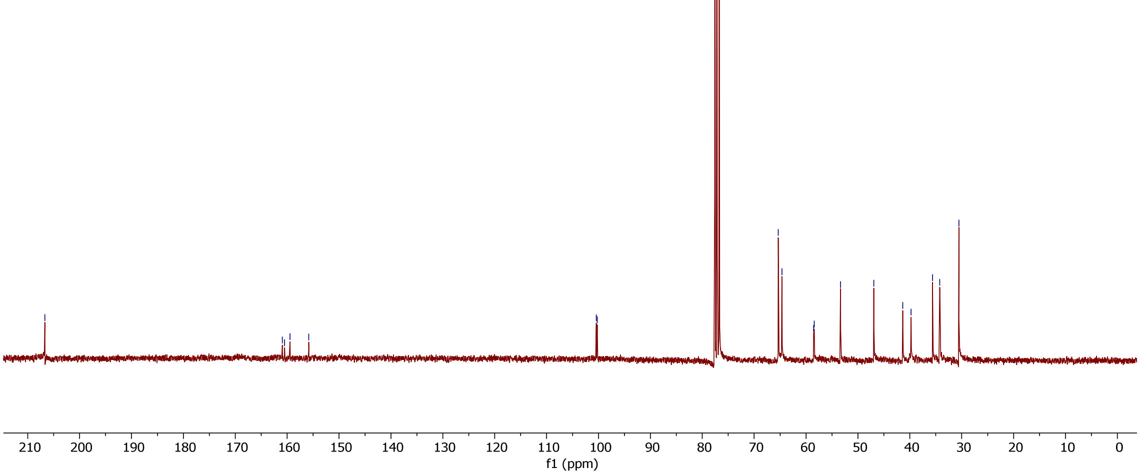

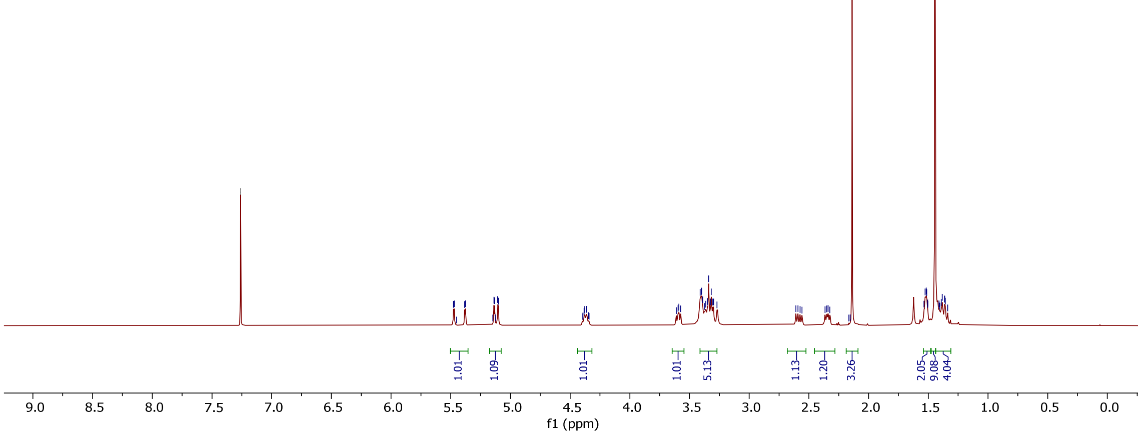

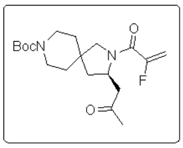

3j

^{1}H NMR (500 MHz, $CDCl_3$)

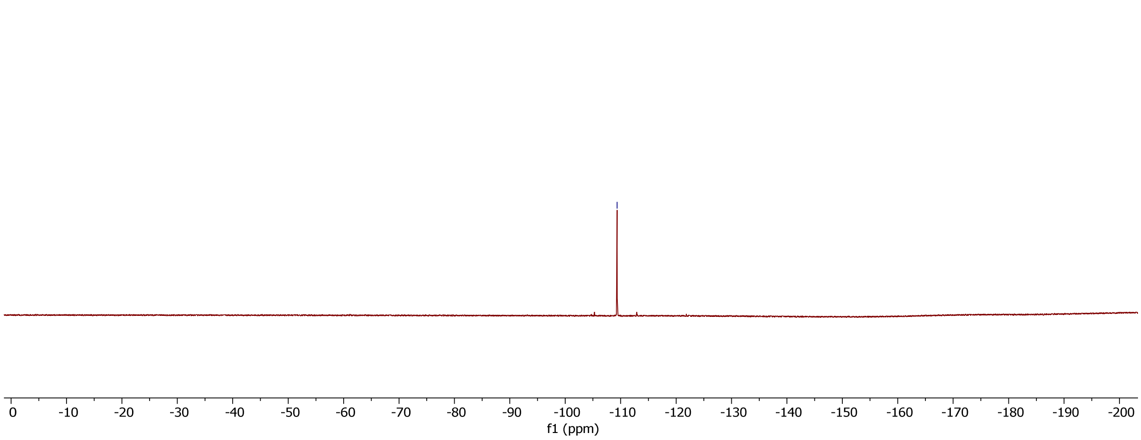

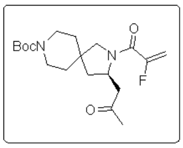

3j

^{19}F NMR (471 MHz, $CDCl_3$)

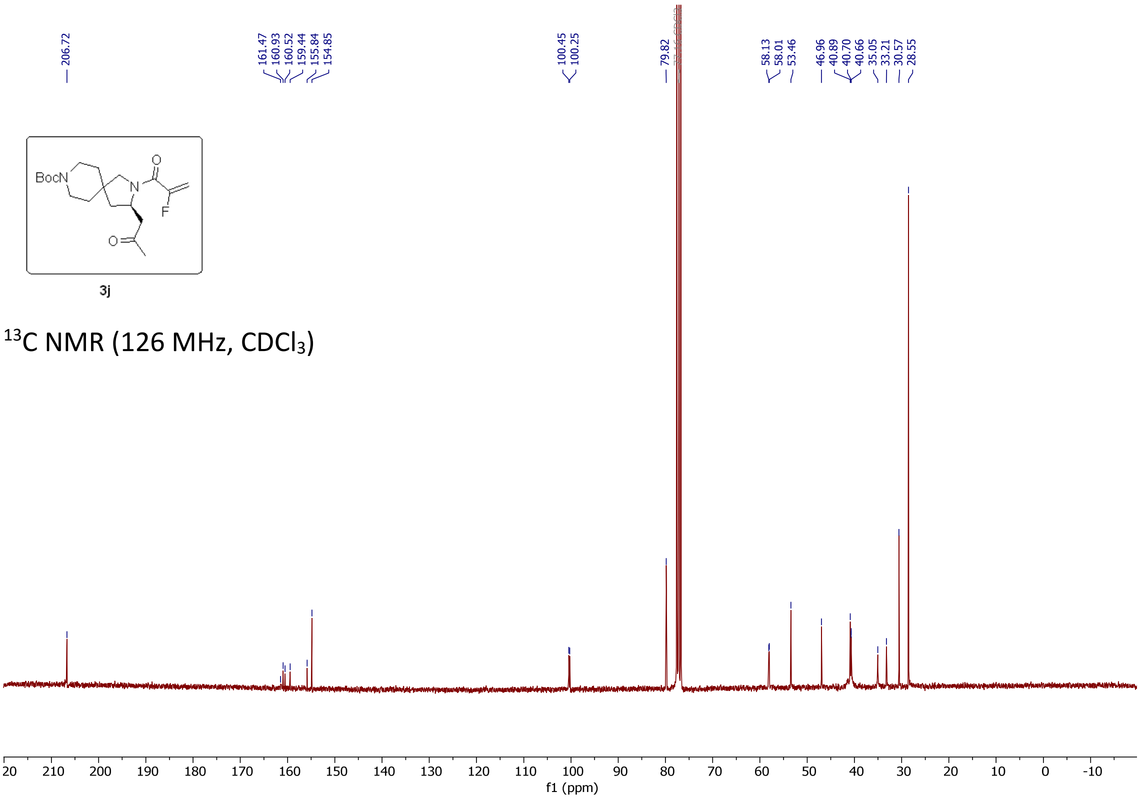

3j

^{13}C NMR (126 MHz, $CDCl_3$)

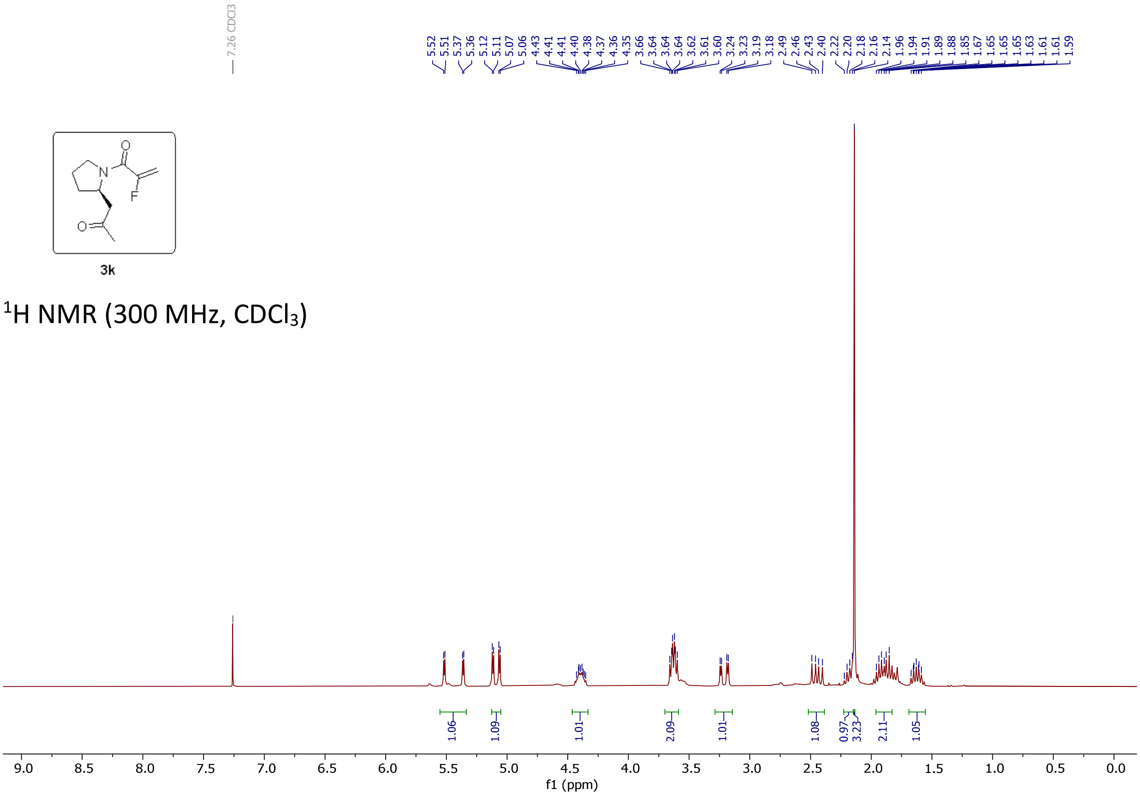

3k

^{1}H NMR (300 MHz, $CDCl_3$)

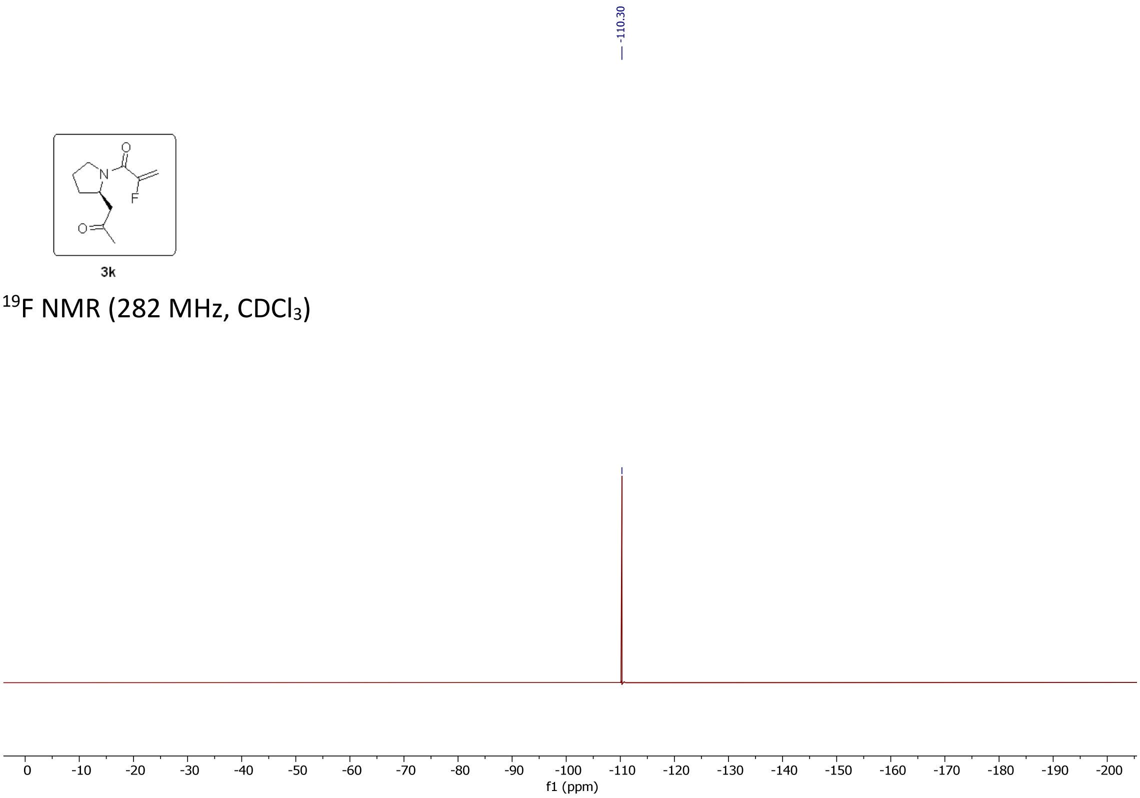

3k

^{19}F NMR (282 MHz, $CDCl_3$)

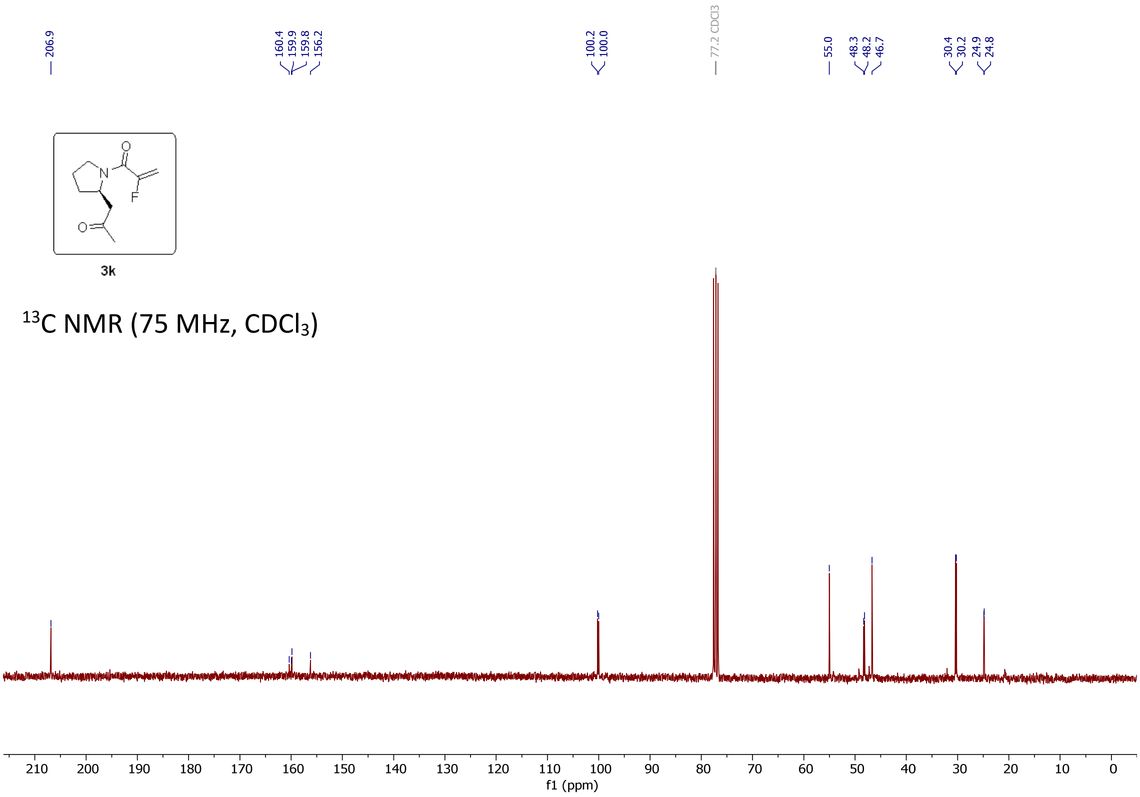

3k

^{13}C NMR (75 MHz, $CDCl_3$)

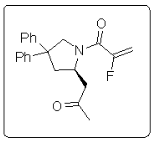

3l

^{1}H NMR (300 MHz, $CDCl_3$)

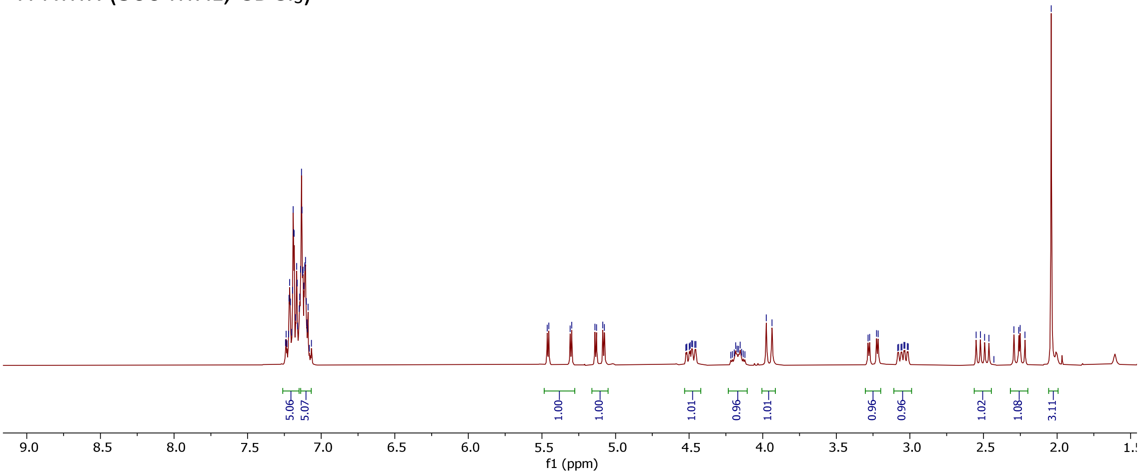

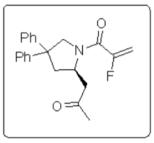

3l

^{19}F NMR (282 MHz, $CDCl_3$)

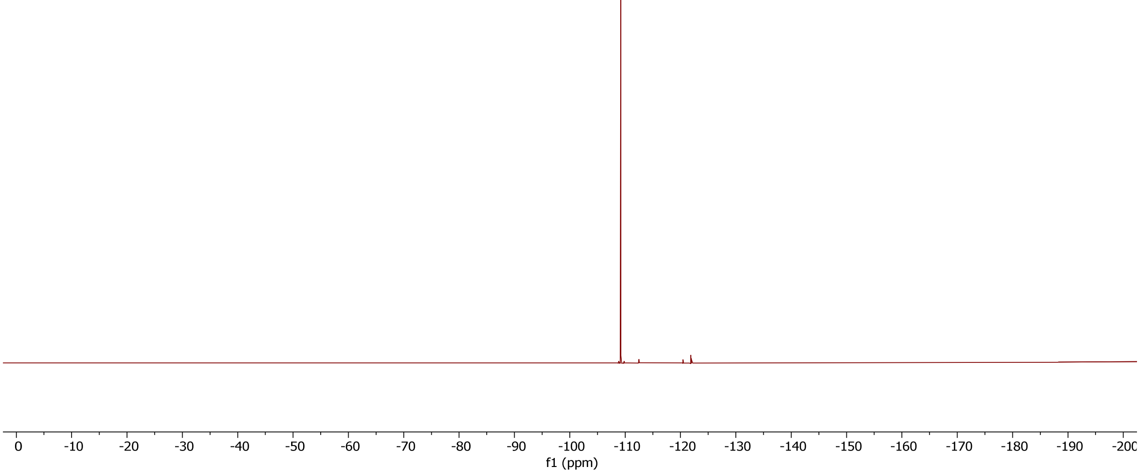

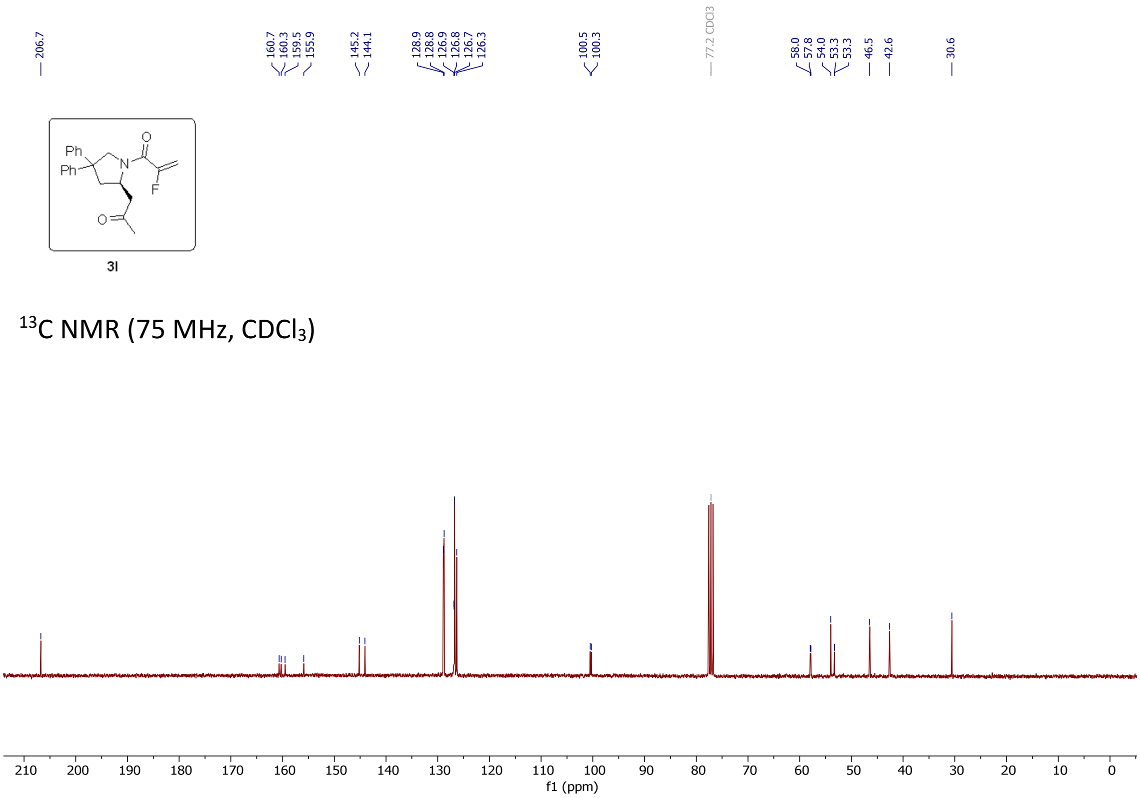

3l

^{13}C NMR (75 MHz, $CDCl_3$)

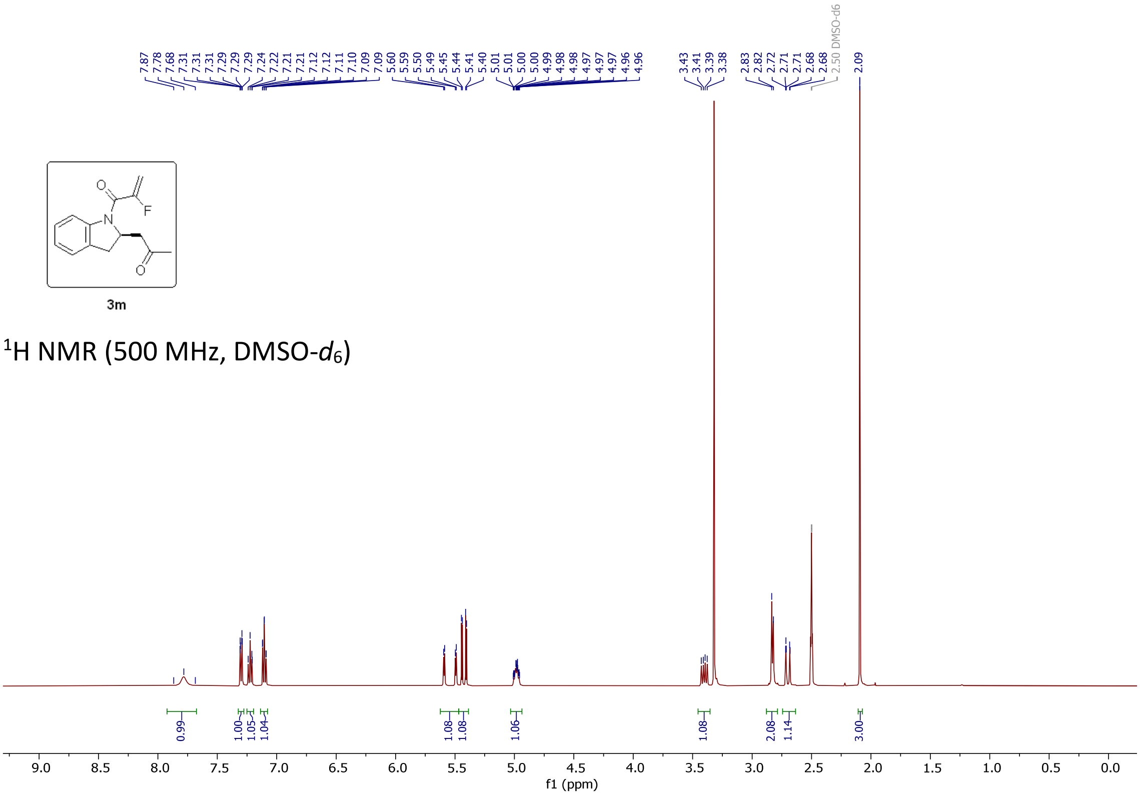

3m

^{1}H NMR (500 MHz, DMSO-d_6)

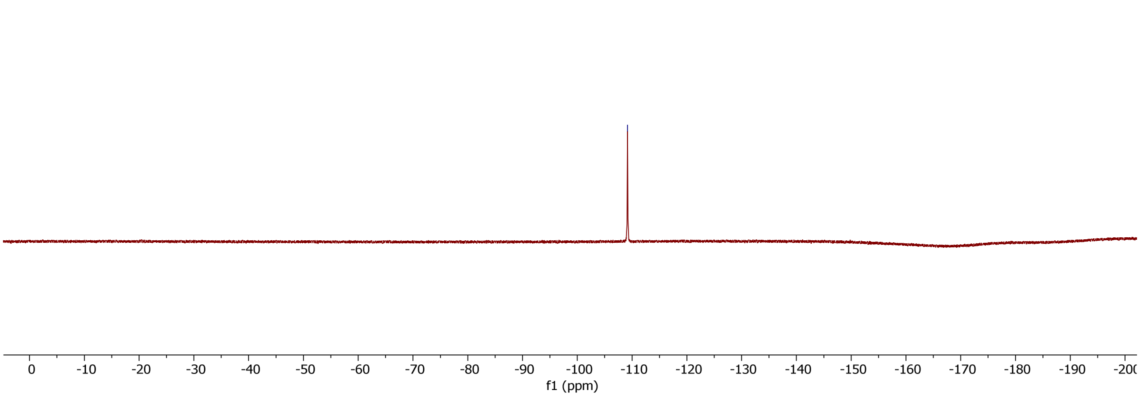

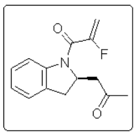

3m

^{19}F NMR (471 MHz, DMSO-d_6)

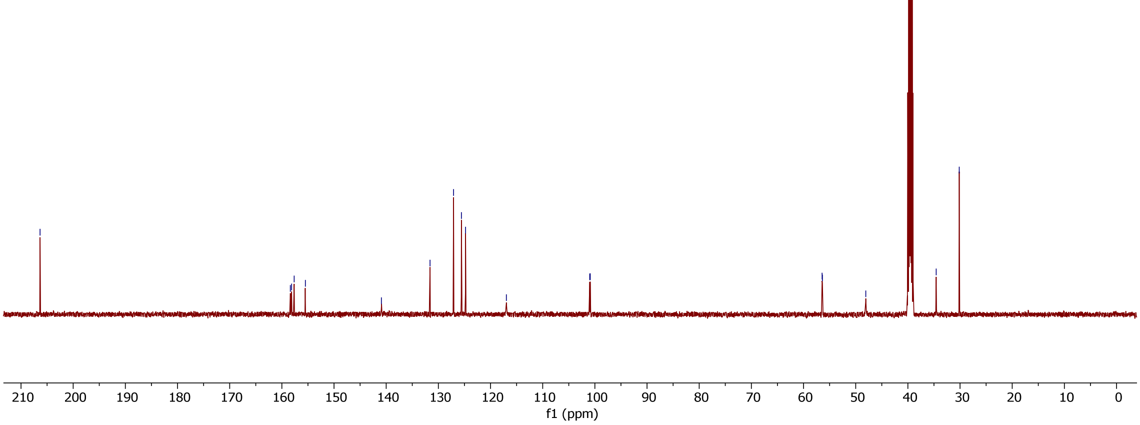

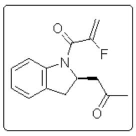

3m

^{13}C NMR (126 MHz, DMSO-d_6)

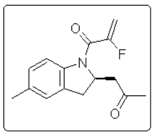

3n

^{1}H NMR (500 MHz, DMSO-d_6)

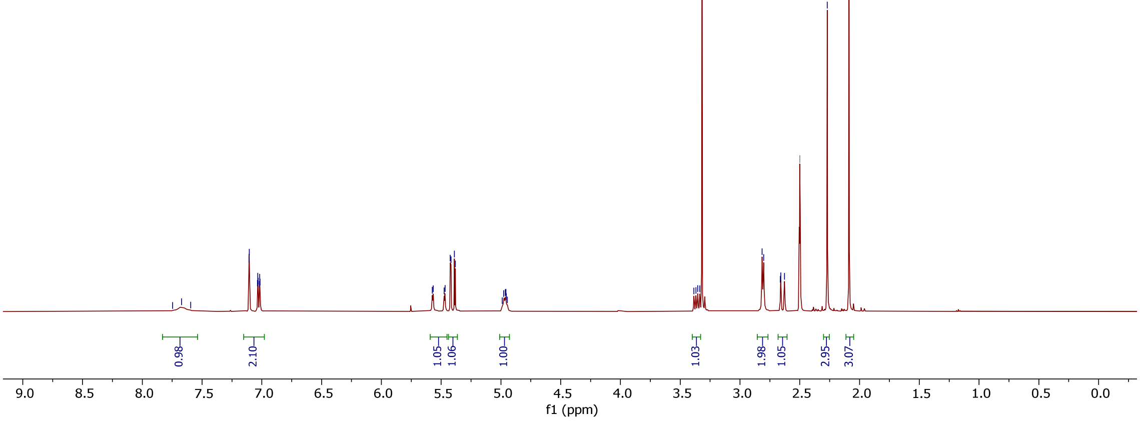

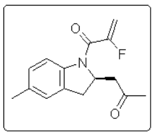

3n

^{19}F NMR (471 MHz, DMSO-d_6)

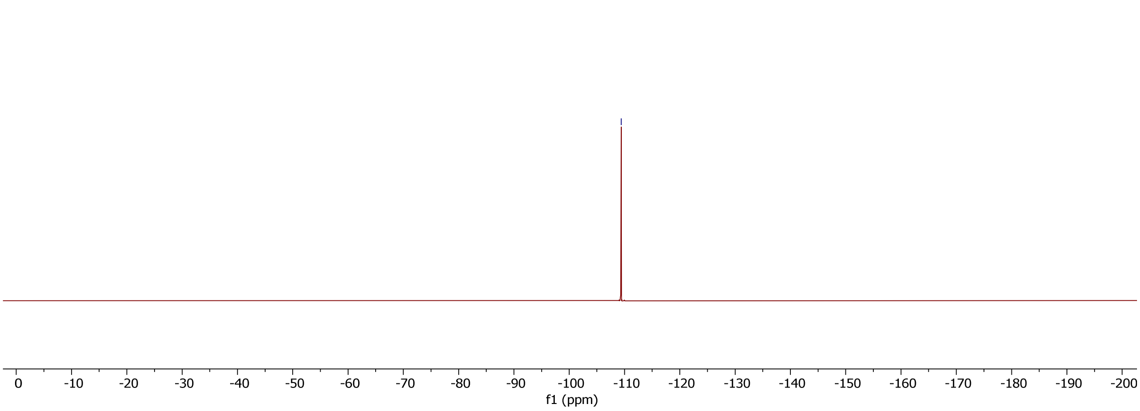

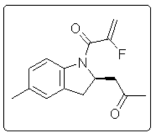

3n

^{13}C NMR (126 MHz, DMSO-d_6)

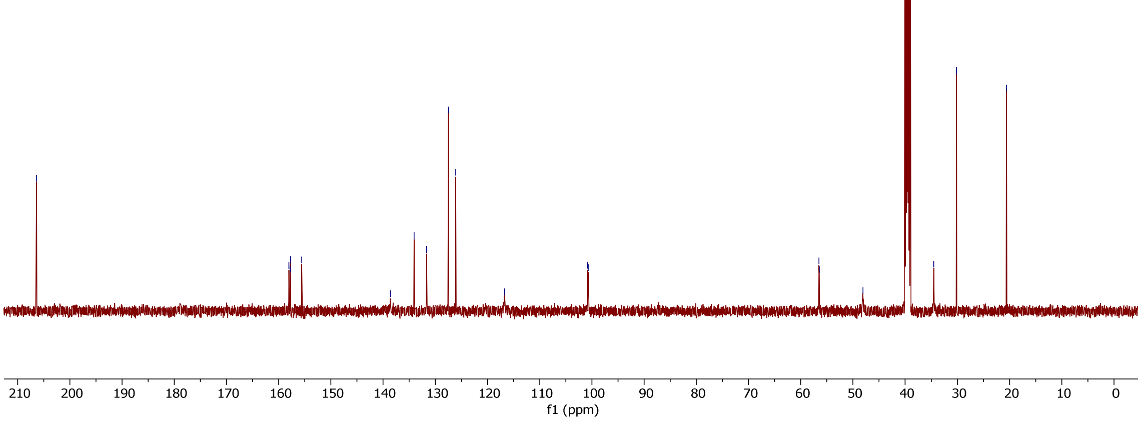

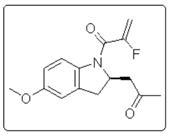

3o

^{1}H NMR (500 MHz, DMSO-d_6)

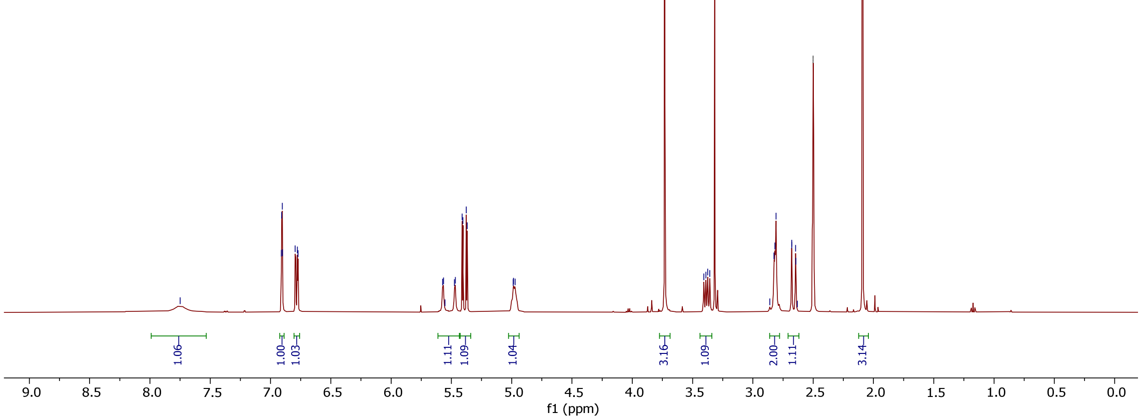

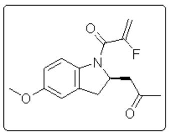

3o

^{19}F NMR (471 MHz, DMSO-d_6)

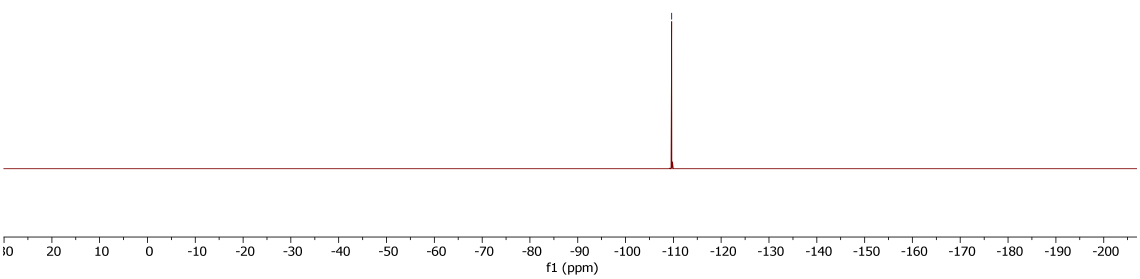

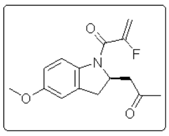

3o

^{13}C NMR (126 MHz, DMSO-d_6)

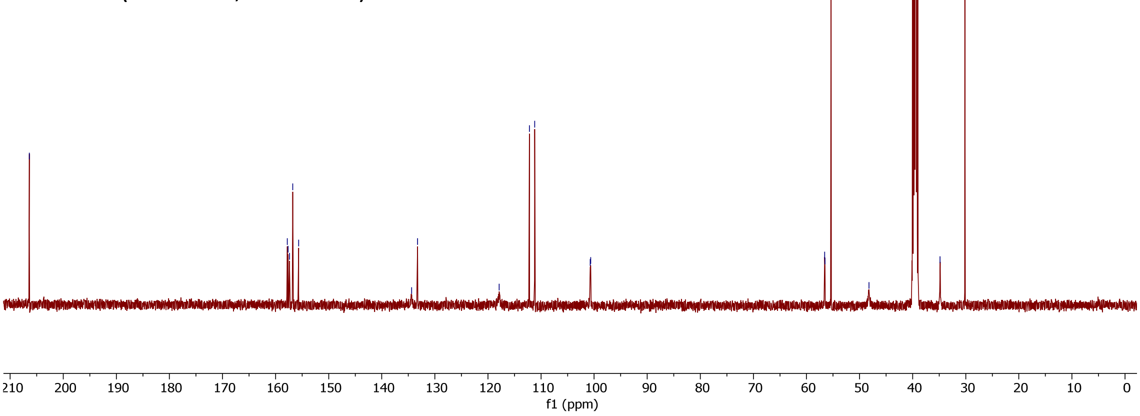

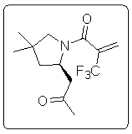

3p

^{1}H NMR (300 MHz, CDCl3)

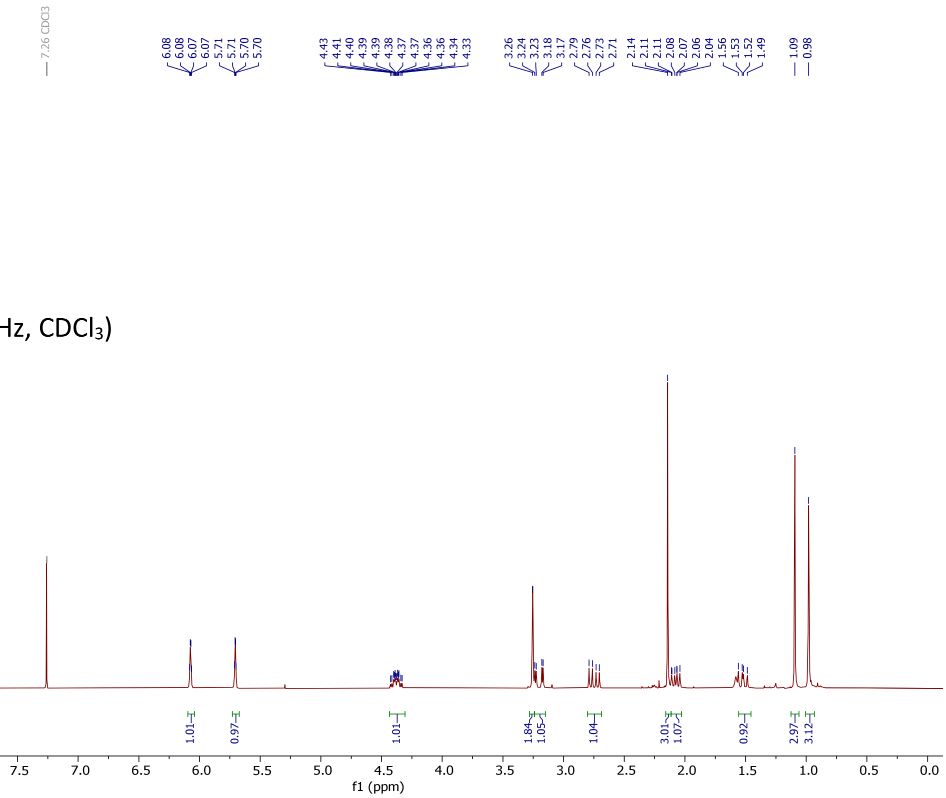

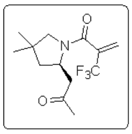

3p

^{19}F NMR (282 MHz, CDCl3)

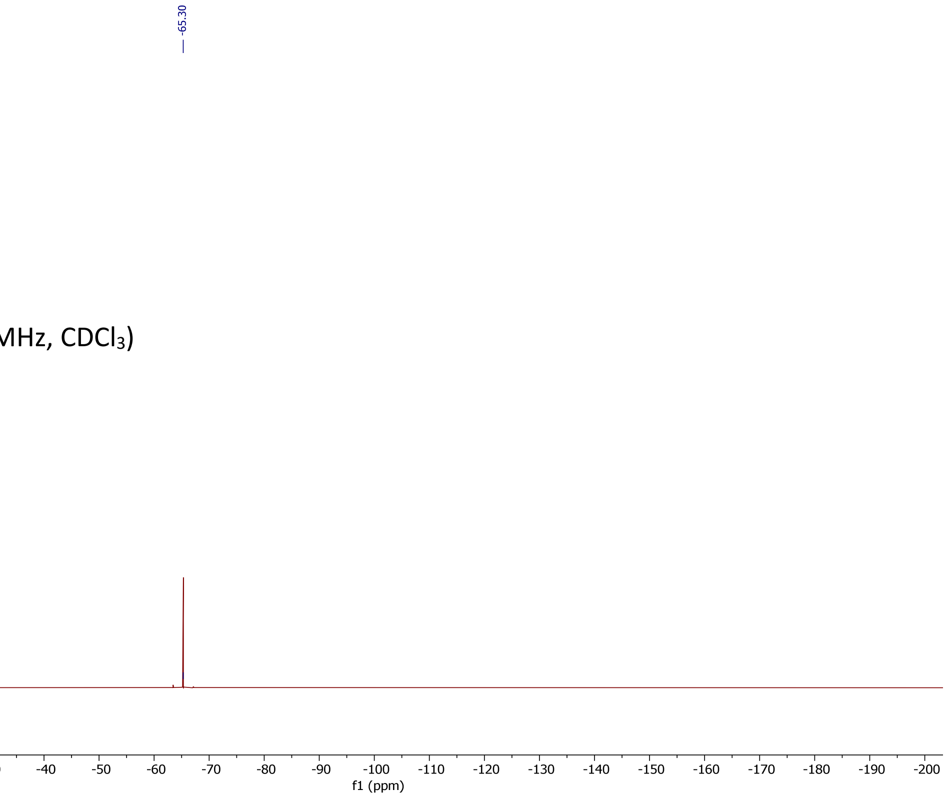

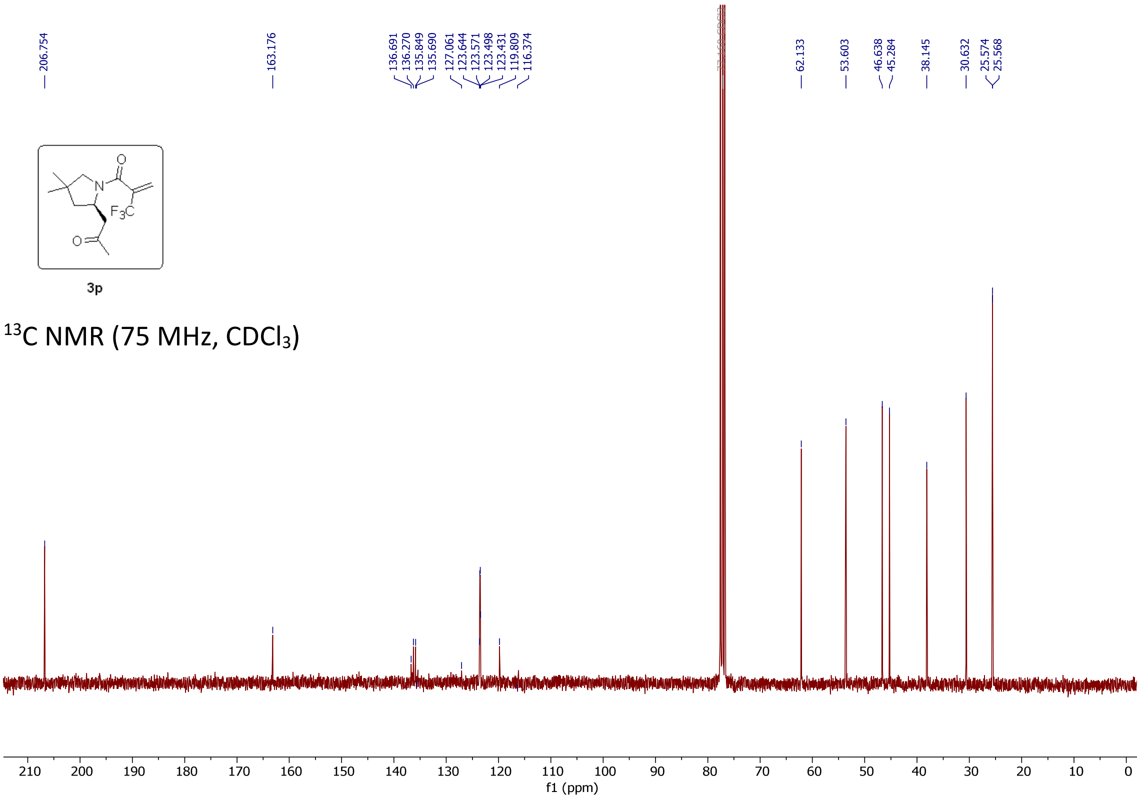

3p

^{13}C NMR (75 MHz, $CDCl_3$)

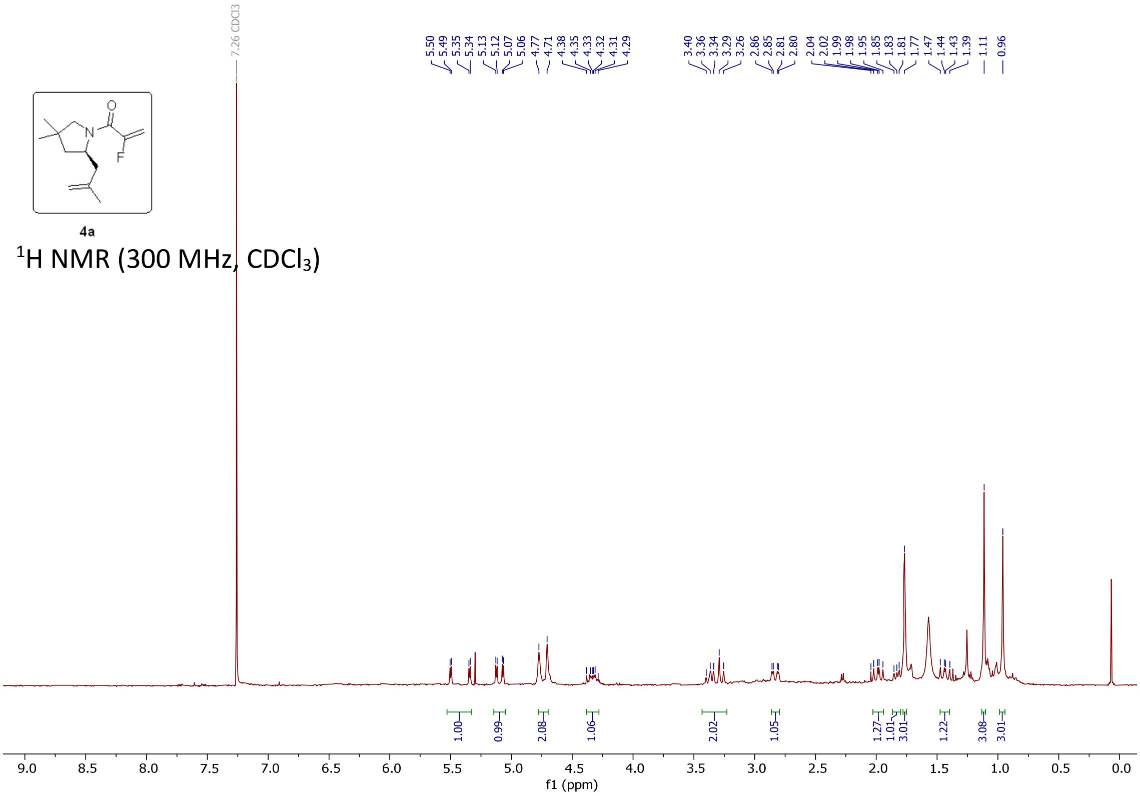

4a

^{1}H NMR (300 MHz, $CDCl_3$)

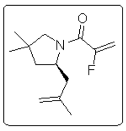

4a

^{19}F NMR (282 MHz, CDCl$_3$)

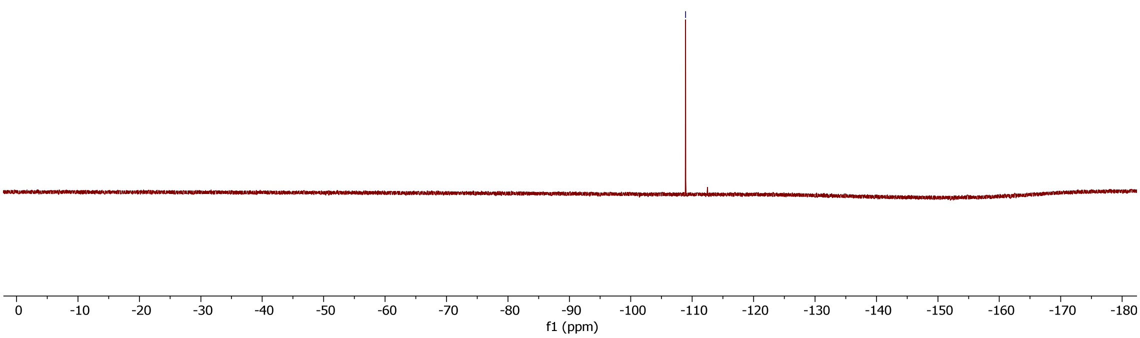

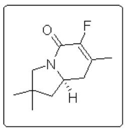

5a

^{1}H NMR (500 MHz, CDCl$_3$)

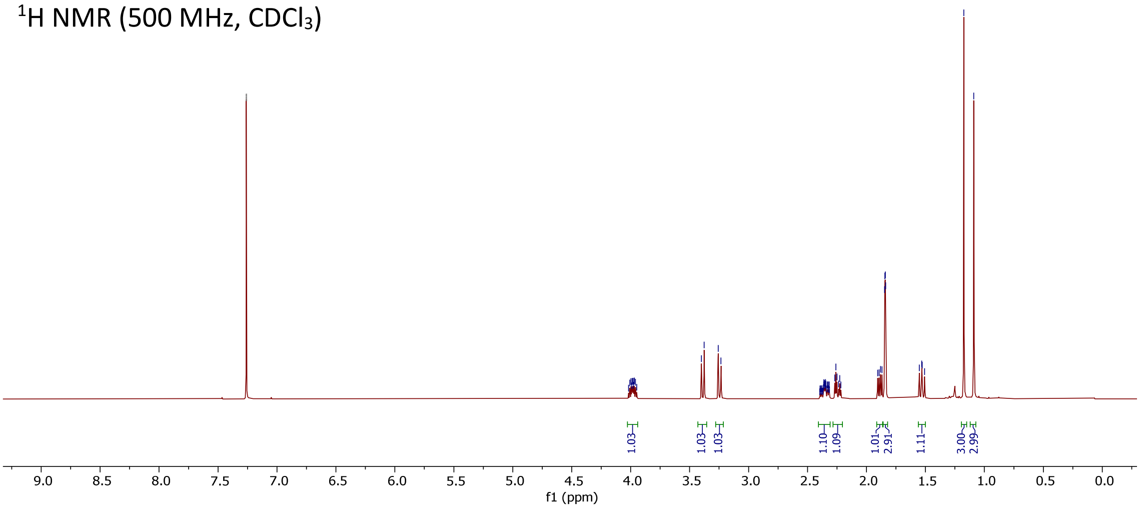

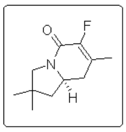

5a

^{19}F NMR (471 MHz, CDCl$_3$)

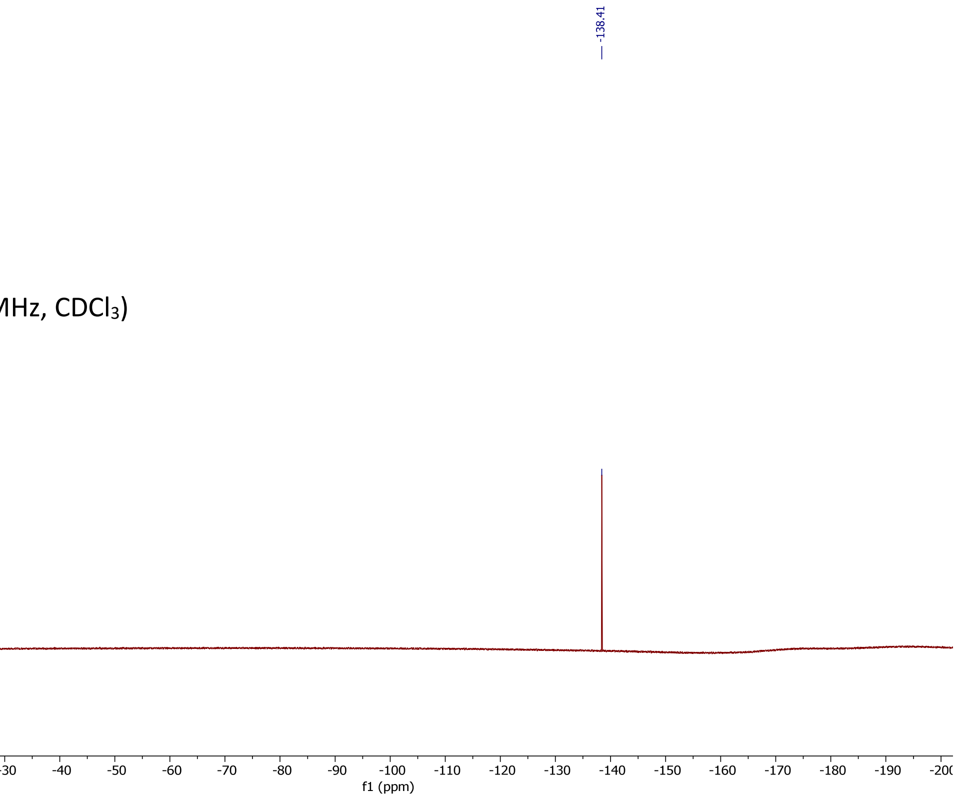

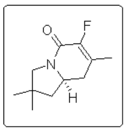

5a

^{13}C NMR (126 MHz, CDCl$_3$)

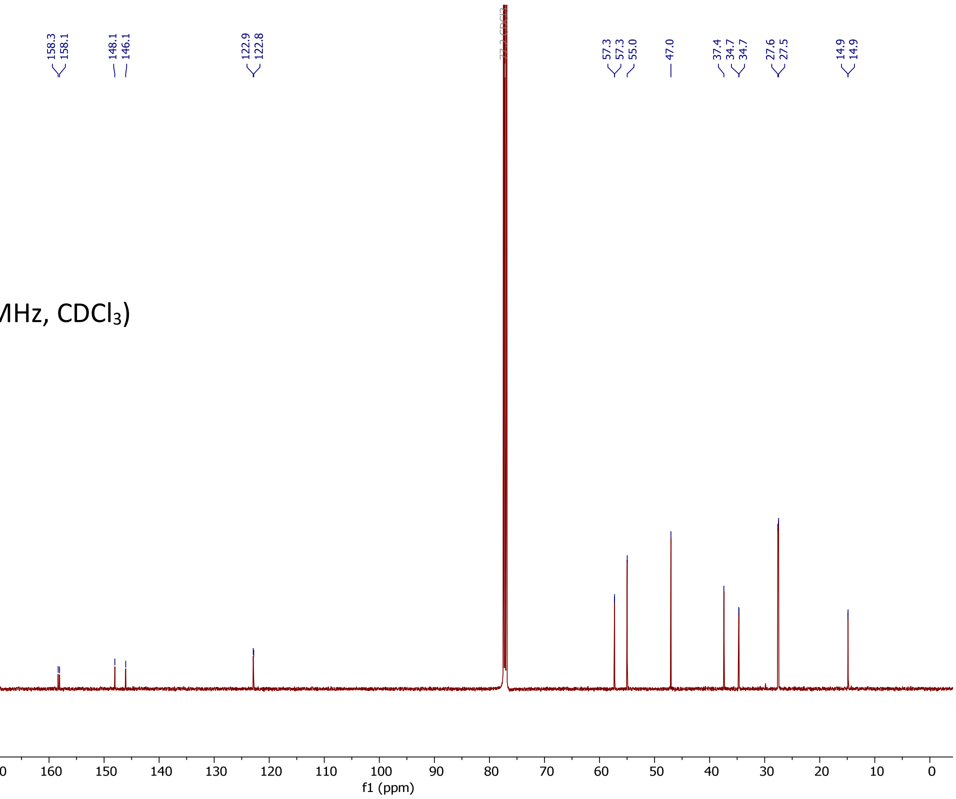

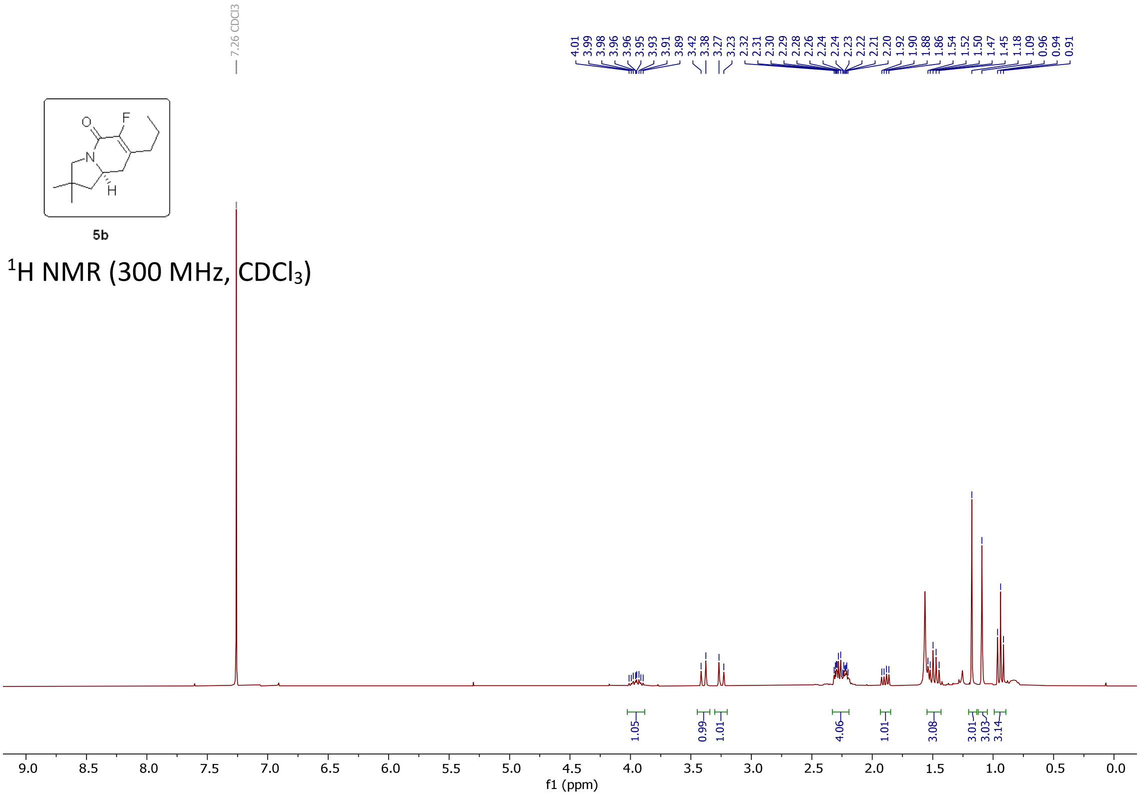

5b

^{1}H NMR (300 MHz, $CDCl_3$)

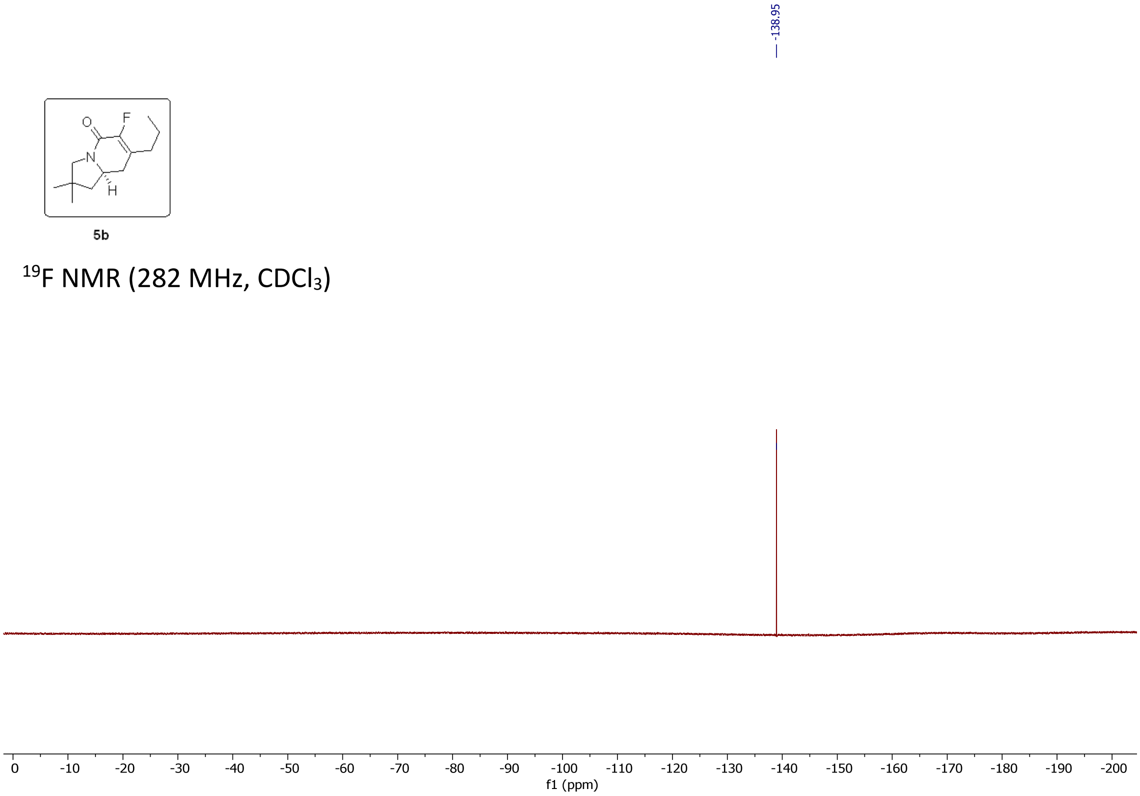

5b

^{19}F NMR (282 MHz, $CDCl_3$)

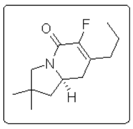

5b

^{13}C NMR (126 MHz, $CDCl_3$)

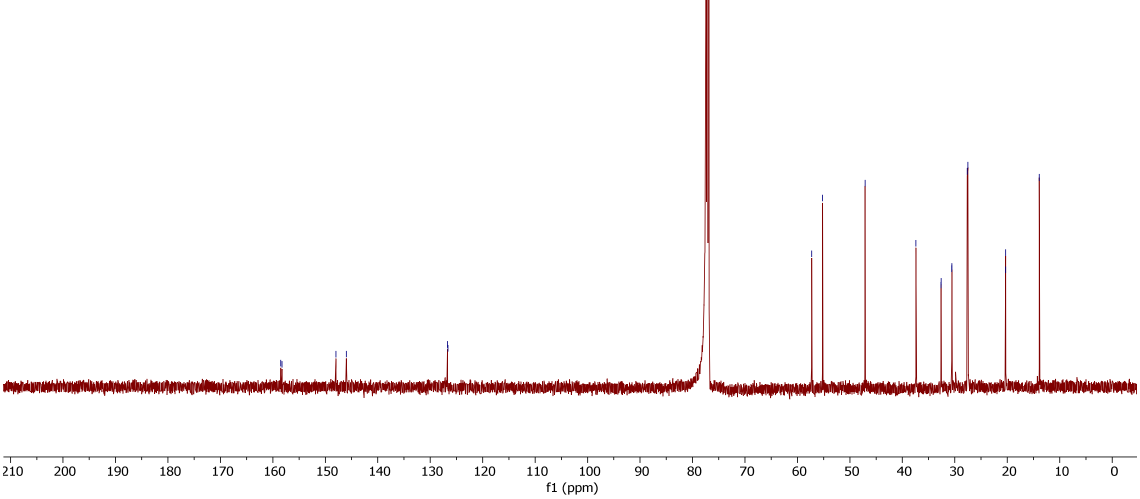

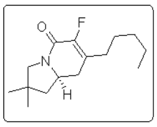

5c

^{1}H NMR (500 MHz, $CDCl_3$)

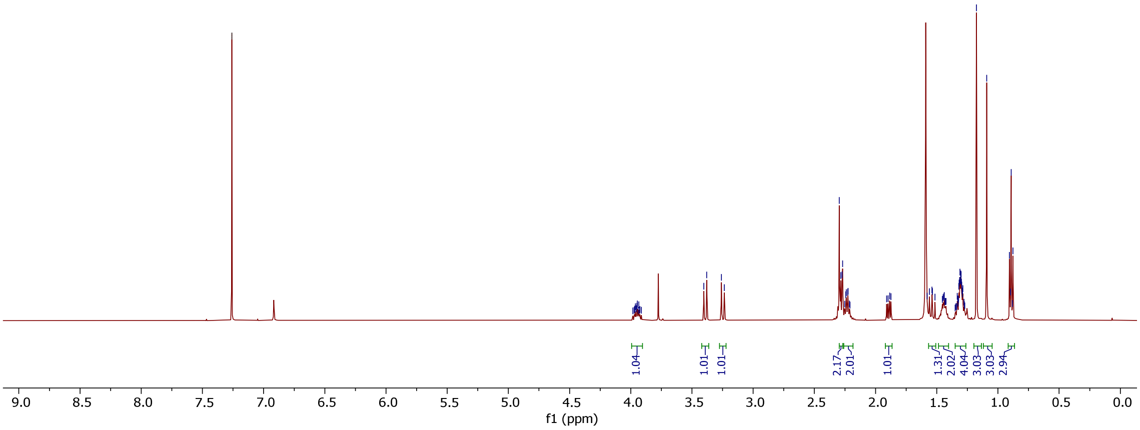

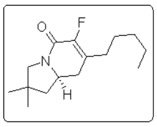

5c

^{19}F NMR (471 MHz, $CDCl_3$)

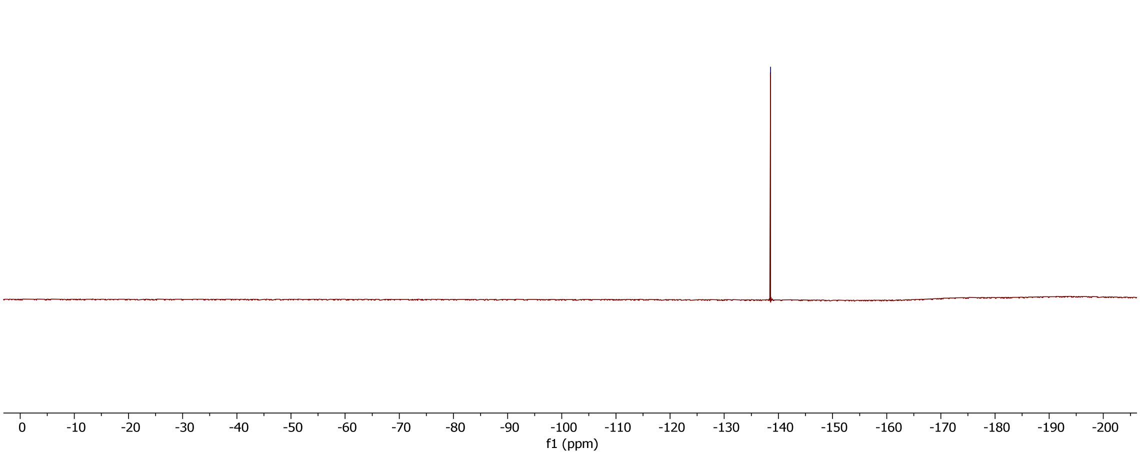

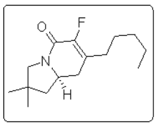

5c

^{13}C NMR (126 MHz, $CDCl_3$)

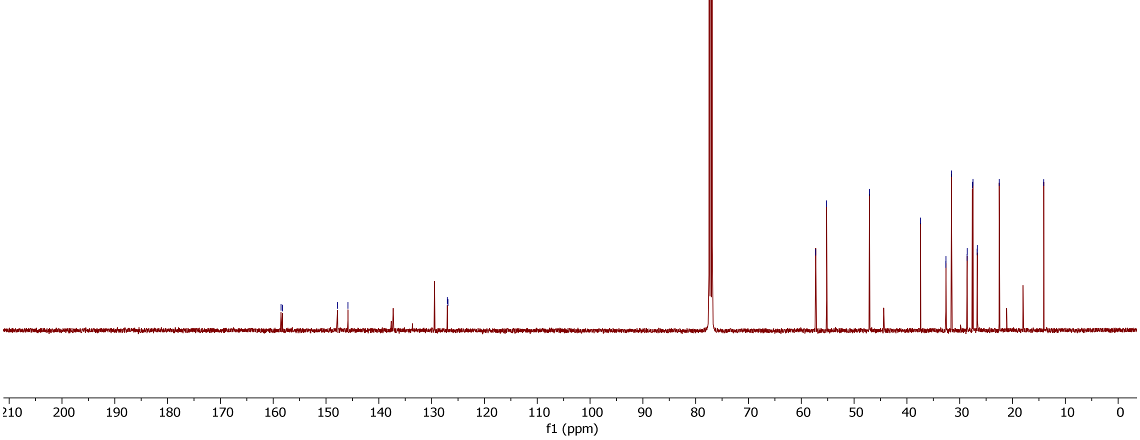

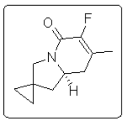

5e

^{1}H NMR (500 MHz, $CDCl_3$)

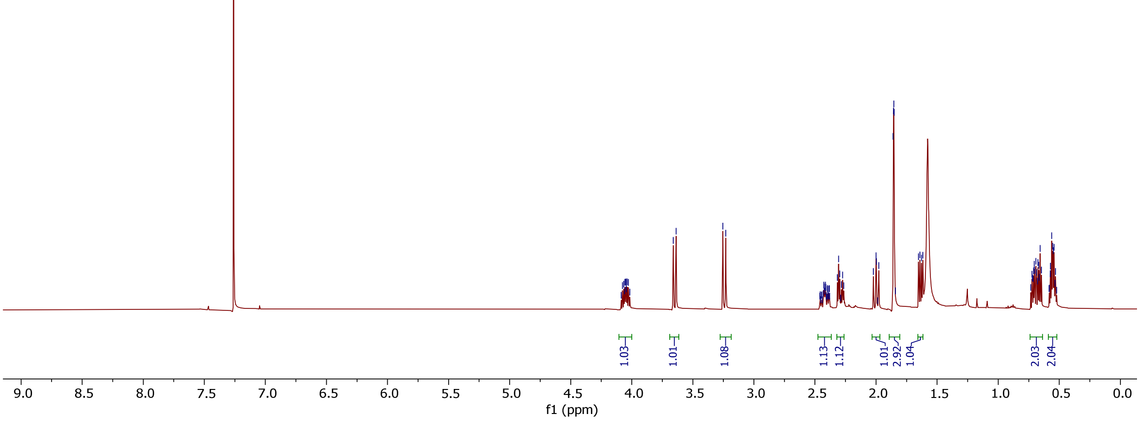

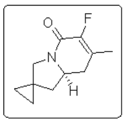

5e

^{19}F NMR (471 MHz, $CDCl_3$)

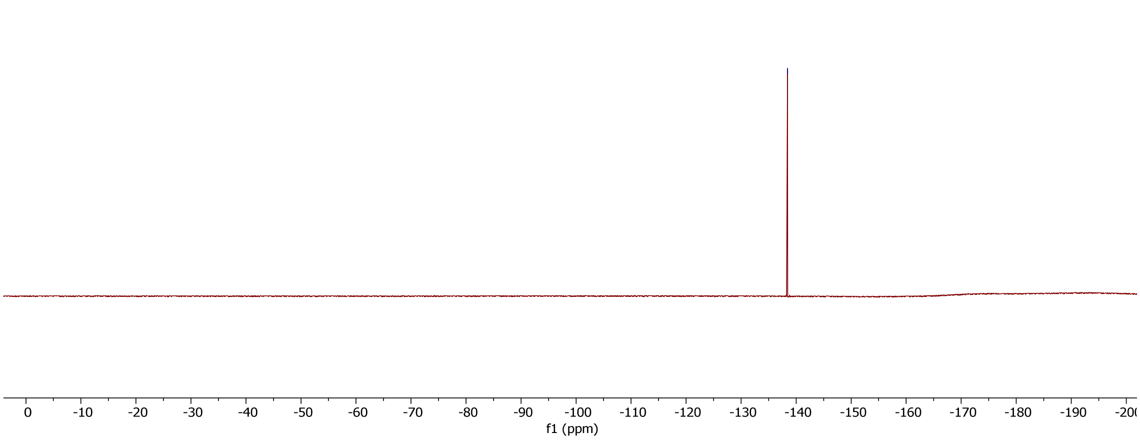

5e

^{13}C NMR (126 MHz, $CDCl_3$)

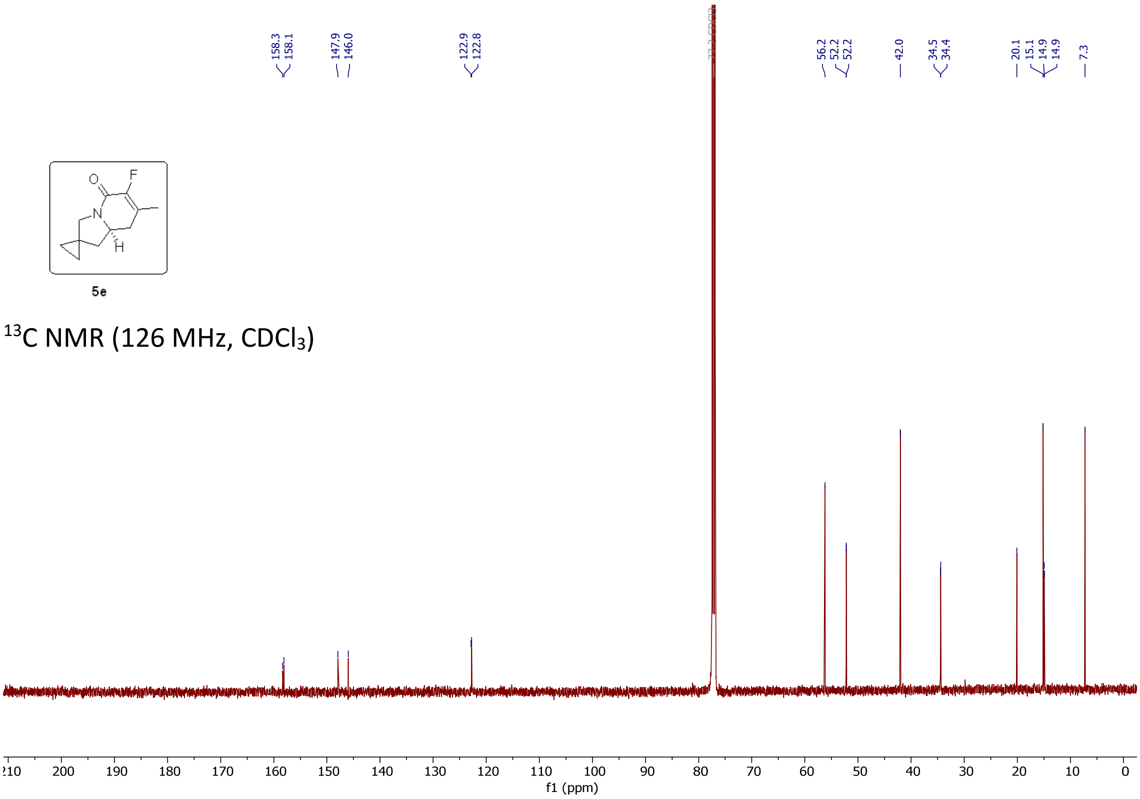

5f

^{1}H NMR (500 MHz, $CDCl_3$)

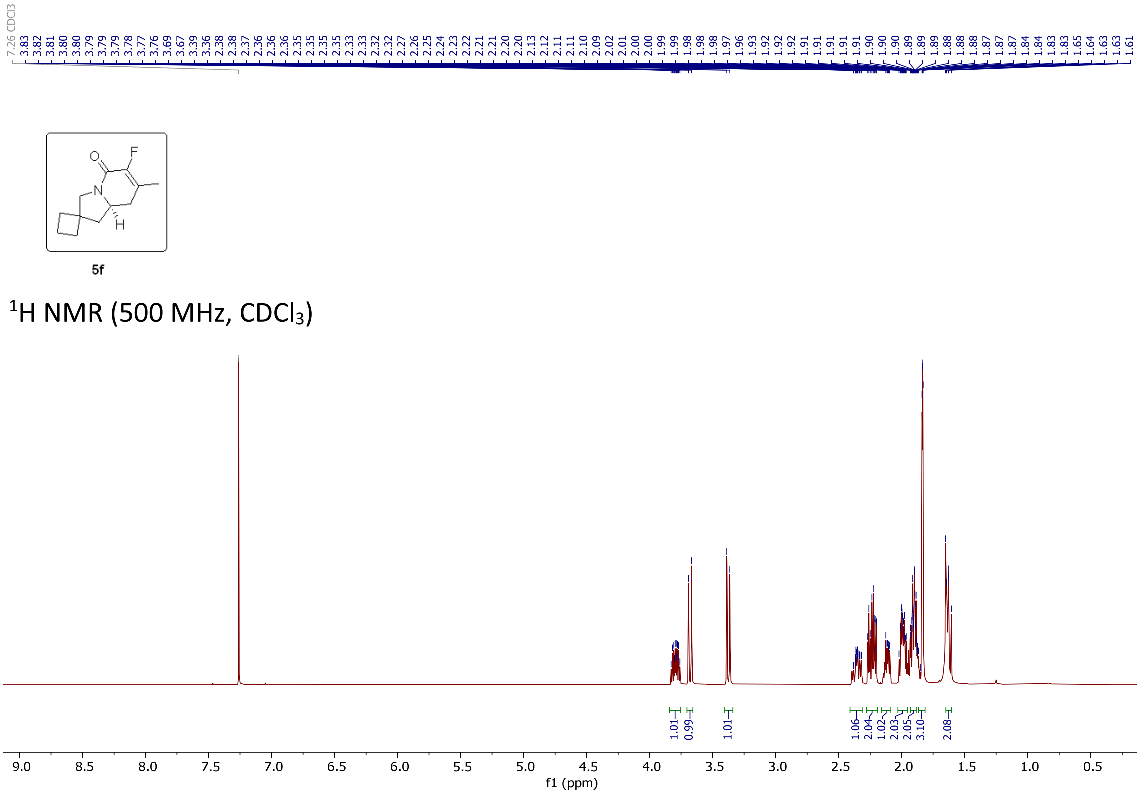

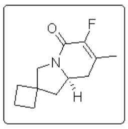

5f

^{19}F NMR (471 MHz, $CDCl_3$)

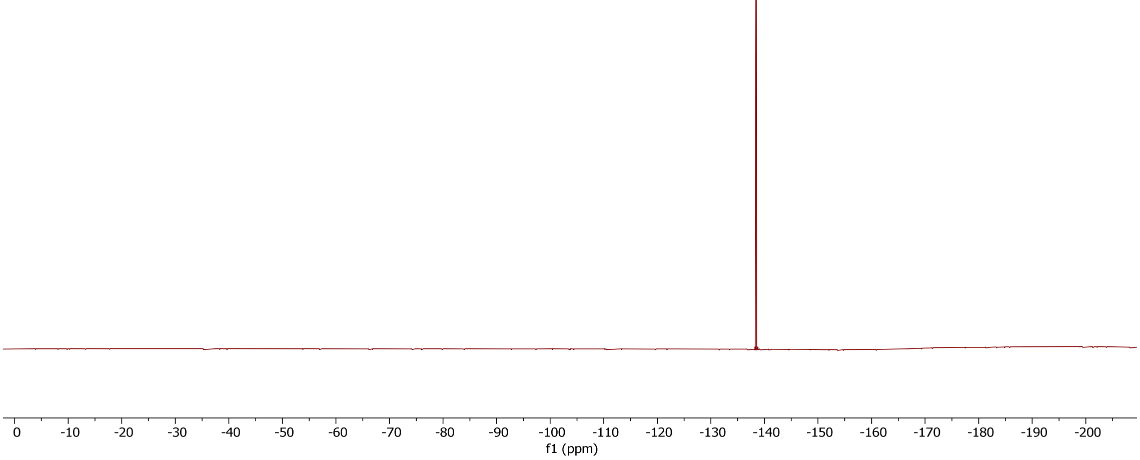

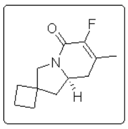

5f

^{13}C NMR (126 MHz, $CDCl_3$)

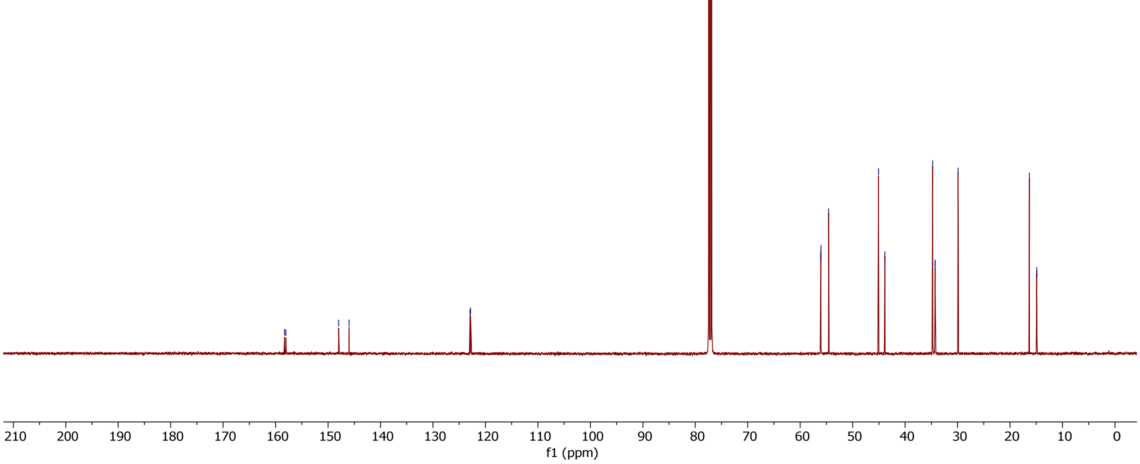

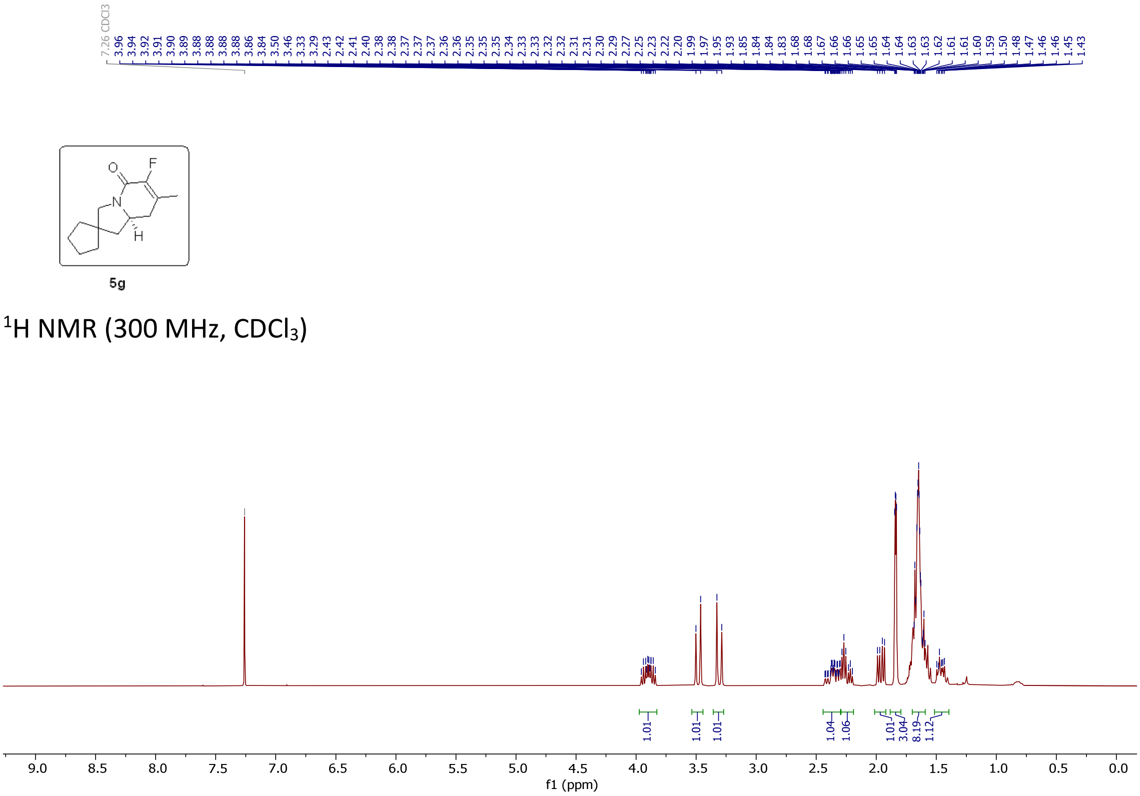

5g

^{1}H NMR (300 MHz, $CDCl_3$)

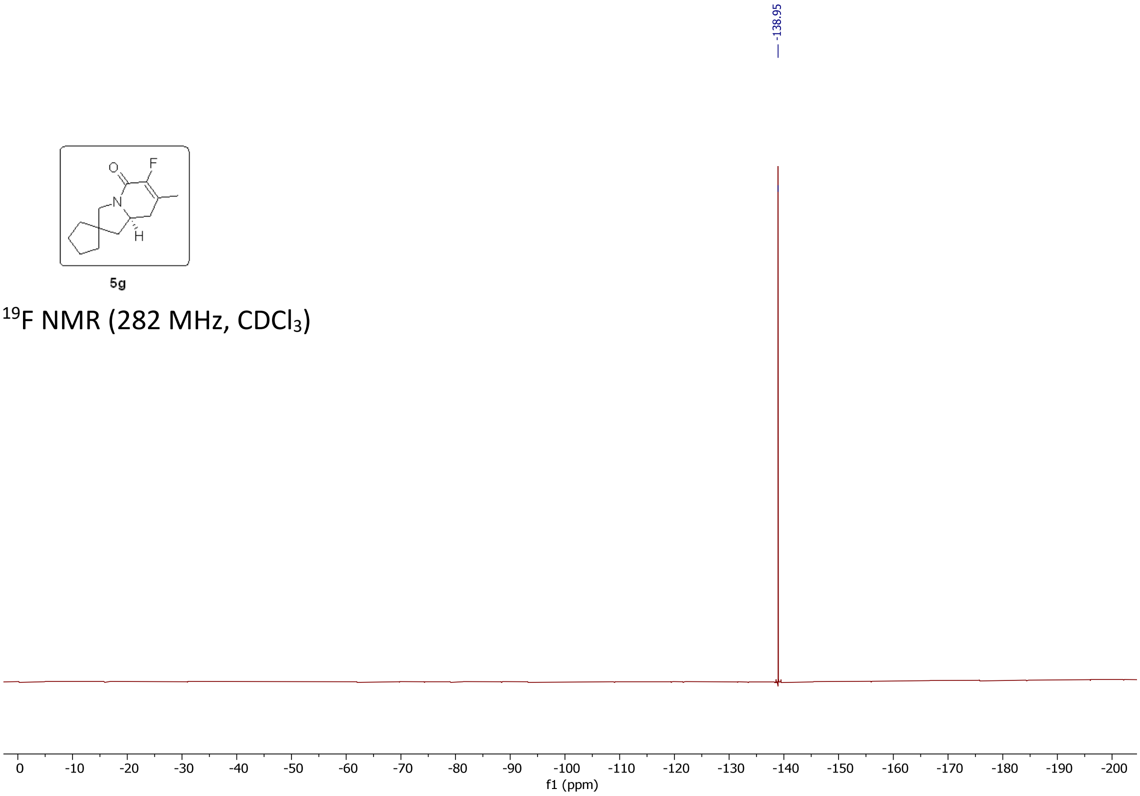

5g

^{19}F NMR (282 MHz, $CDCl_3$)

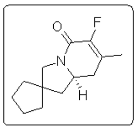

5g

^{13}C NMR (75 MHz, $CDCl_3$)

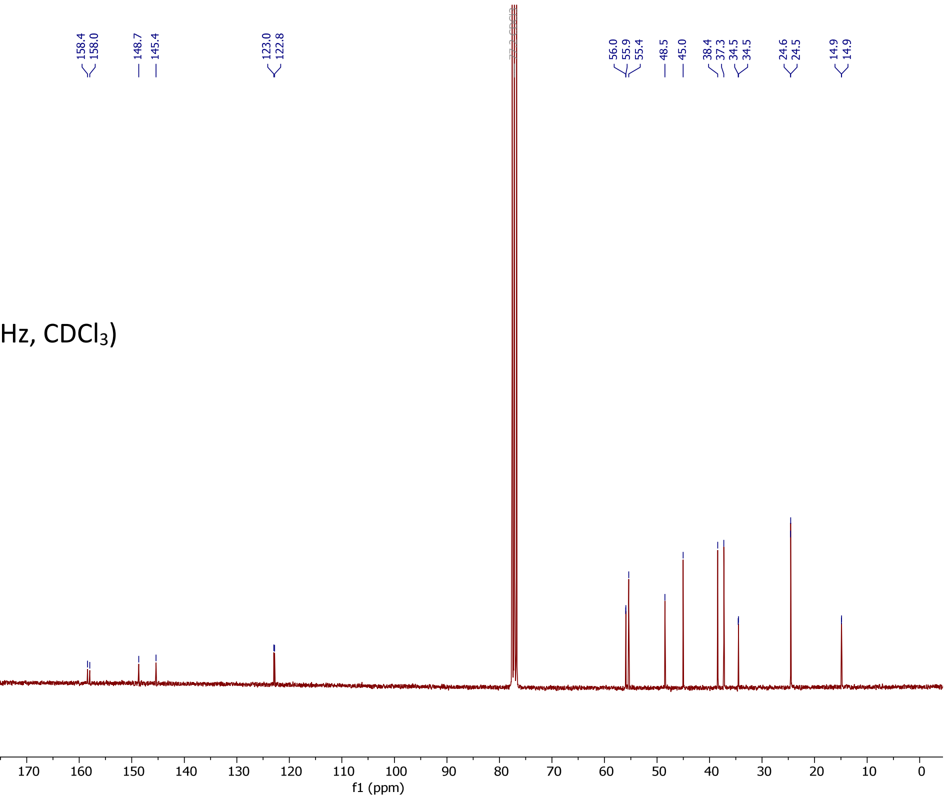

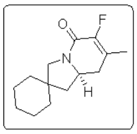

5h

^{1}H NMR (300 MHz, $CDCl_3$)

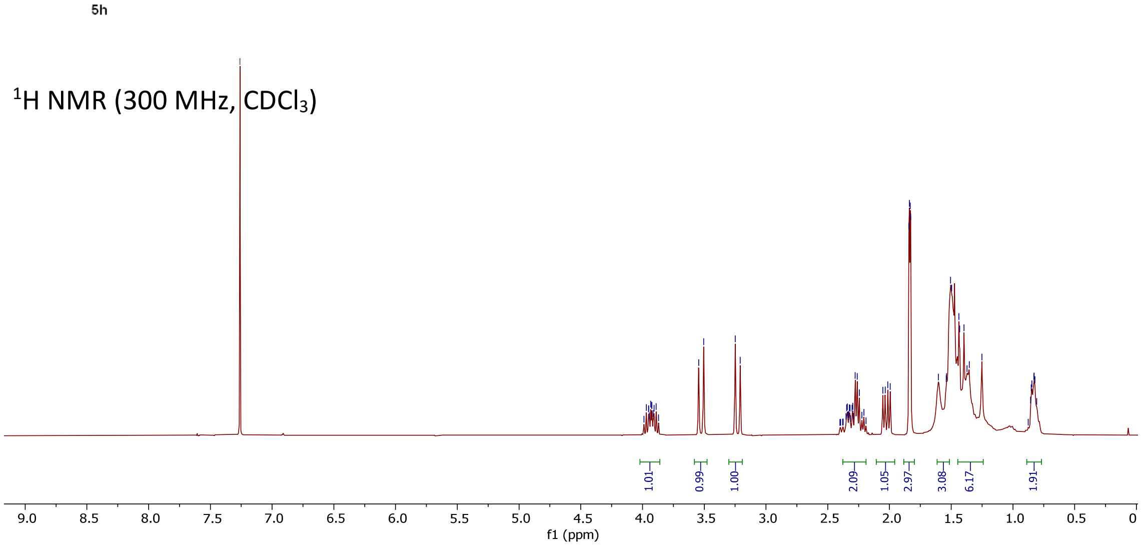

5h

^{19}F NMR (282 MHz, $CDCl_3$)

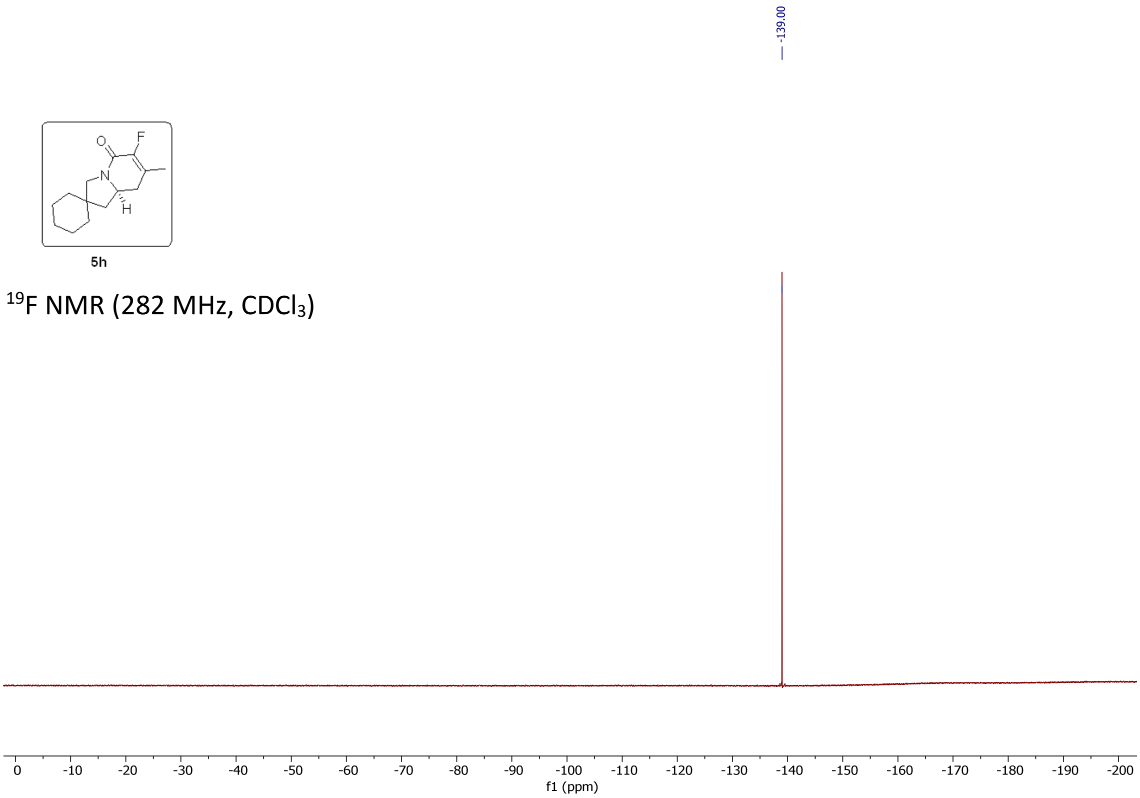

5h

^{13}C NMR (126 MHz, $CDCl_3$)

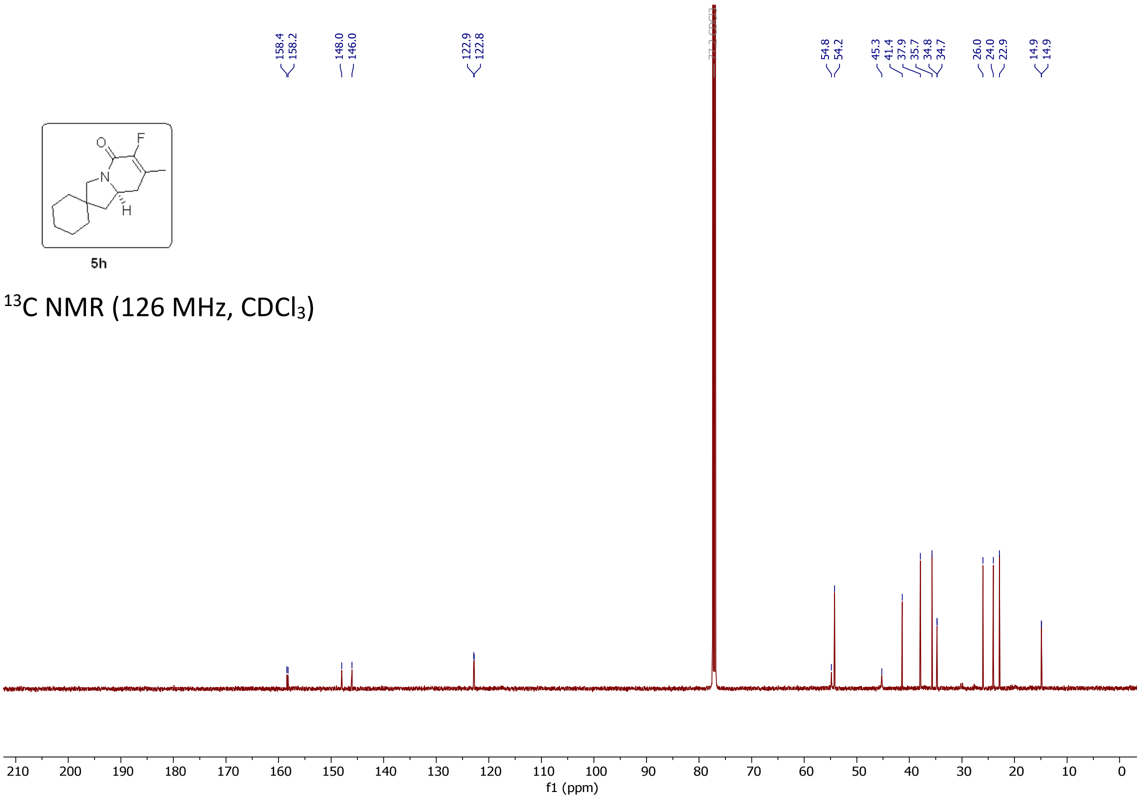

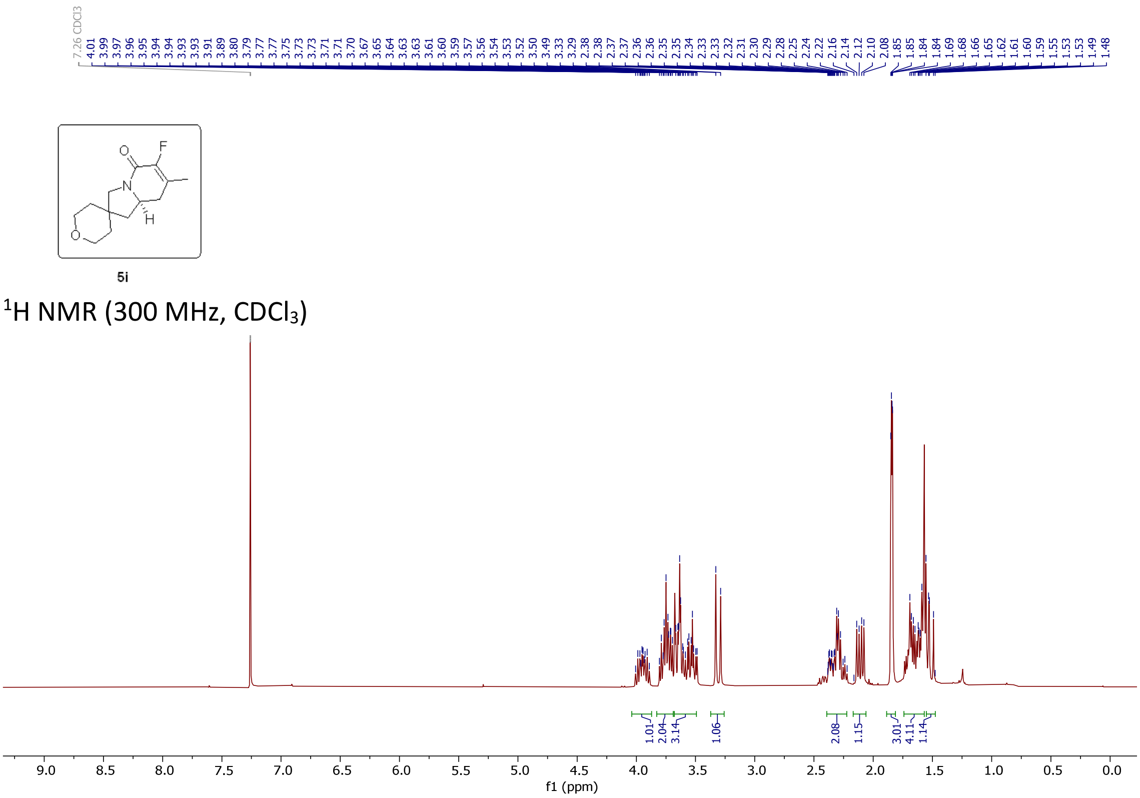

5i

^{1}H NMR (300 MHz, $CDCl_3$)

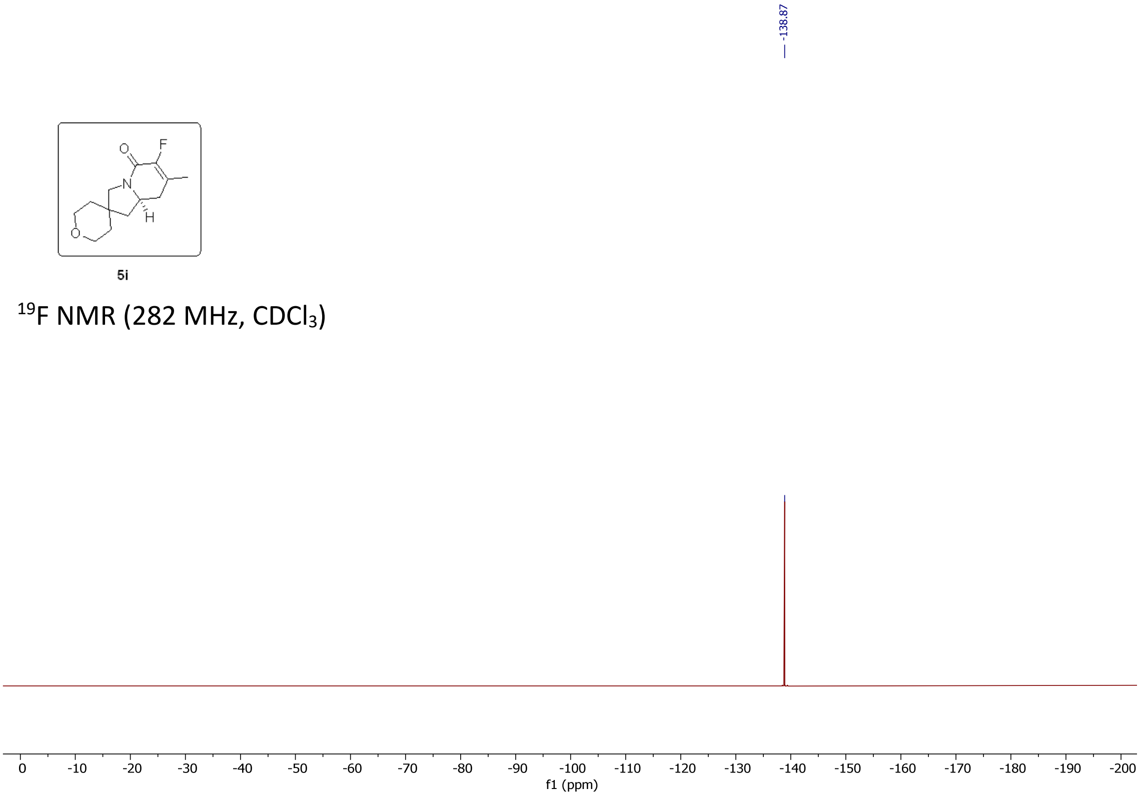

5i

^{19}F NMR (282 MHz, $CDCl_3$)

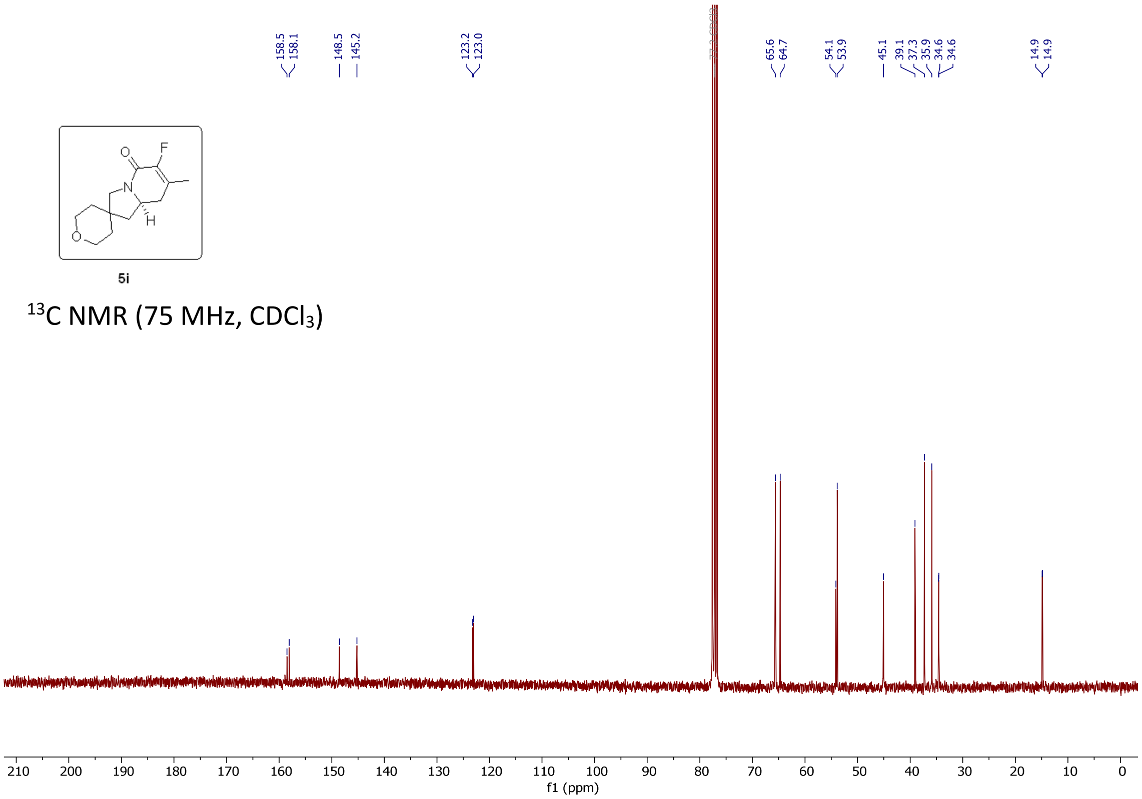

158.5
158.1
148.5
145.2
123.2
123.0
65.6
64.7
54.1
53.9
45.1
39.1
37.3
35.9
34.6
34.6
14.9
14.9
O
F
N
H
O
5i
^{13}C NMR (75 MHz, CDCl$_3$)
210 200 190 180 170 160 150 140 130 120 110 100 90 80 70 60 50 40 30 20 10 0
f1 (ppm)

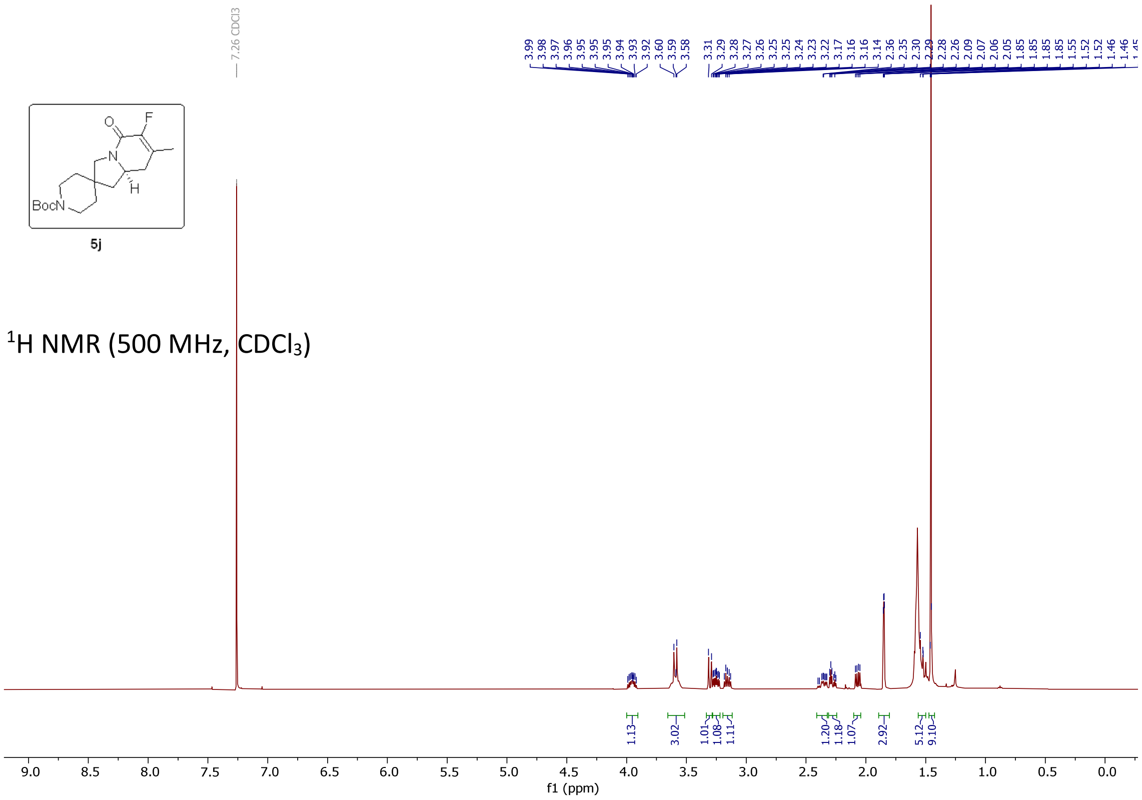

7.26 CDCl3
3.99 3.98 3.97 3.96 3.95 3.95 3.95 3.94 3.93 3.92 3.60 3.59 3.58 3.31 3.29 3.28 3.27 3.26 3.25 3.25 3.24 3.23 3.22 3.17 3.16 3.16 3.14 2.36 2.35 2.30 2.29 2.28 2.26 2.09 2.07 2.06 2.05 1.85 1.85 1.85 1.55 1.52 1.52 1.46 1.46
O
F
N
H
BocN
5j
^{1}H NMR (500 MHz, CDCl$_3$)
1.13
3.02
1.01
1.08
1.11
1.20
1.18
1.07
2.92
5.12
9.10
9.0 8.5 8.0 7.5 7.0 6.5 6.0 5.5 5.0 4.5 4.0 3.5 3.0 2.5 2.0 1.5 1.0 0.5 0.0
f1 (ppm)

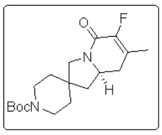

5j

^{19}F NMR (471 MHz, $CDCl_3$)

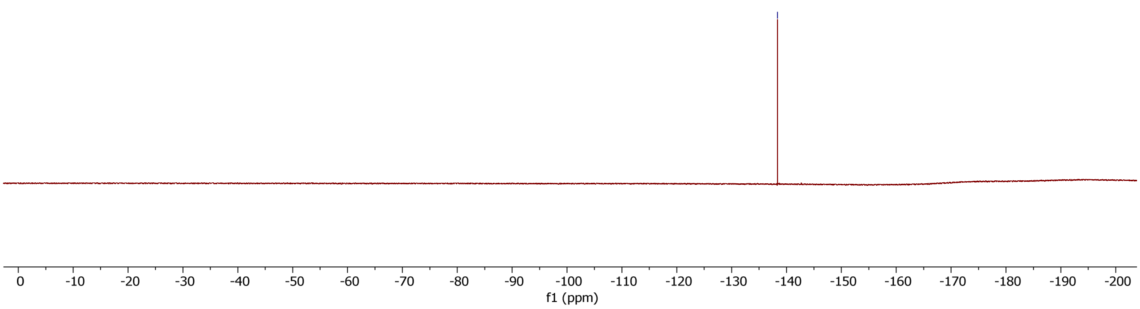

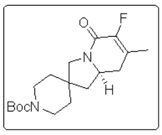

5j

^{13}C NMR (126 MHz, $CDCl_3$)

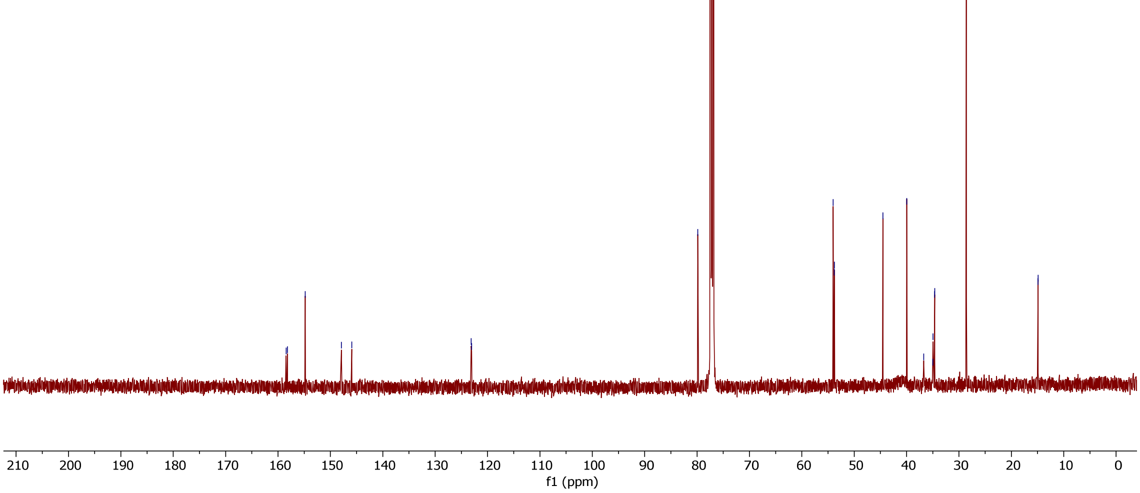

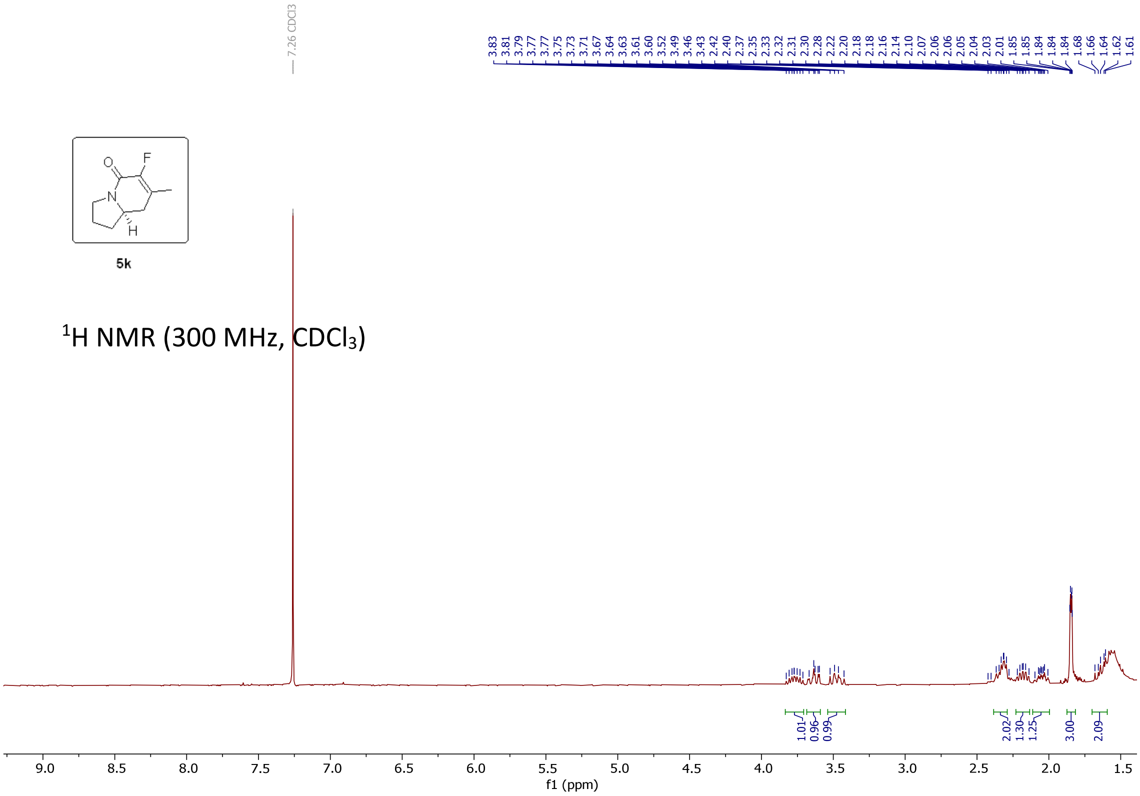

^{1}H NMR (300 MHz, $CDCl_3$)

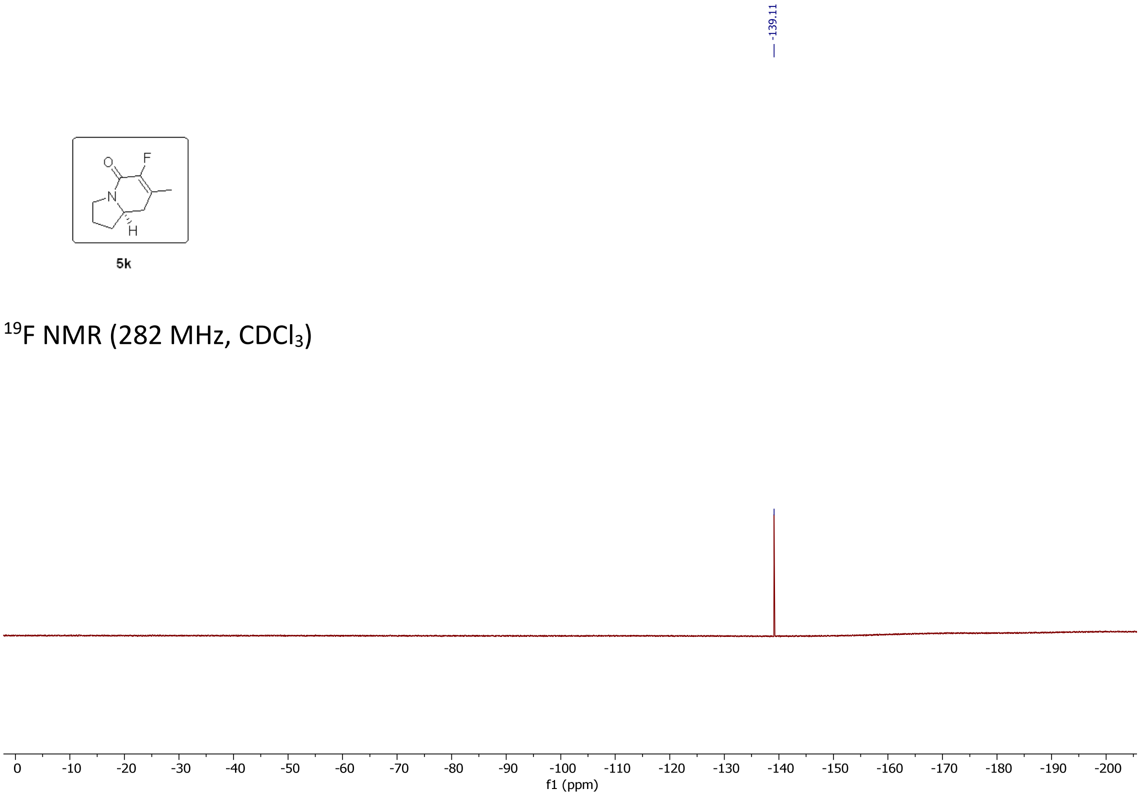

^{19}F NMR (282 MHz, $CDCl_3$)

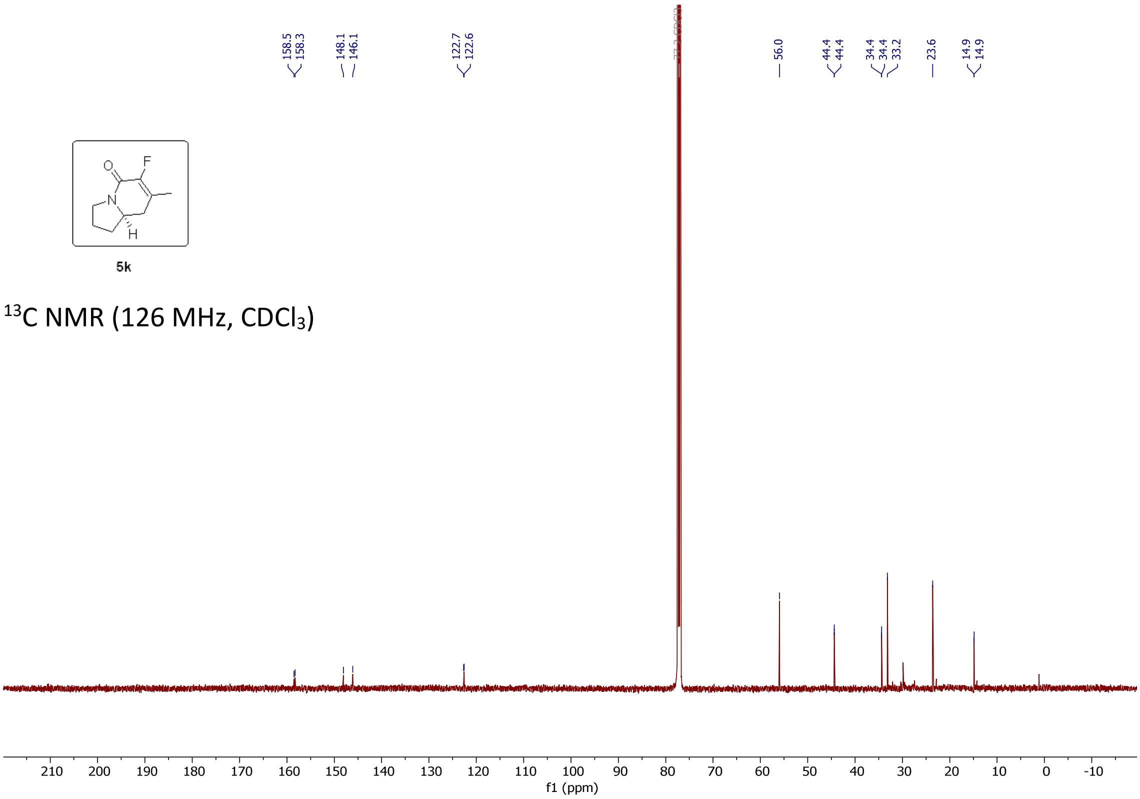

5k

^{13}C NMR (126 MHz, $CDCl_3$)

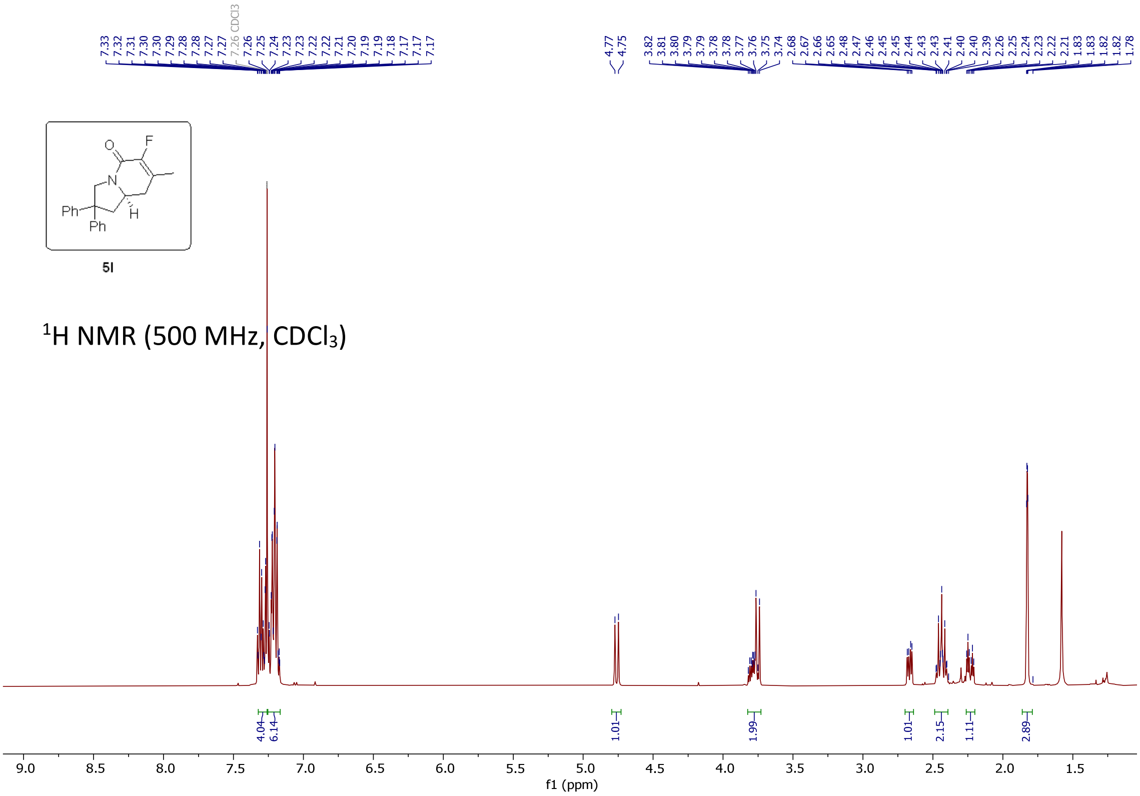

5l

^{1}H NMR (500 MHz, $CDCl_3$)

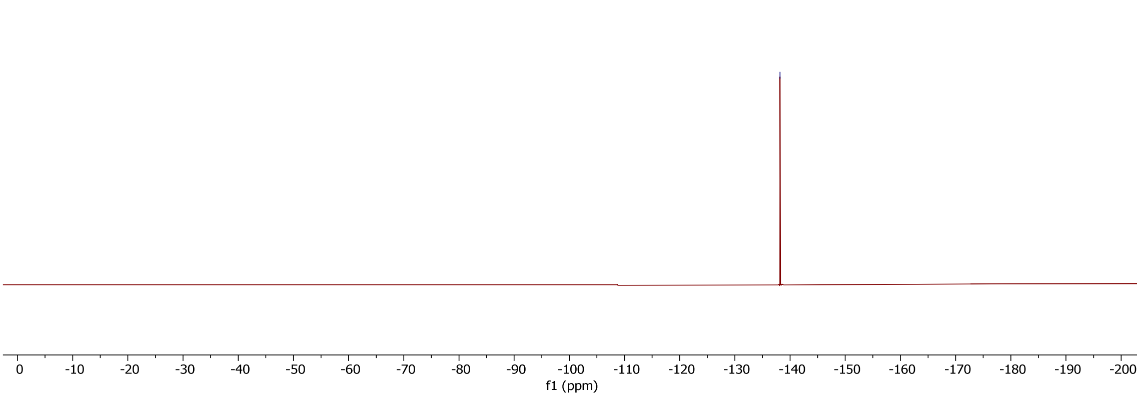

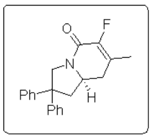

5l

^{19}F NMR (471 MHz, $CDCl_3$)

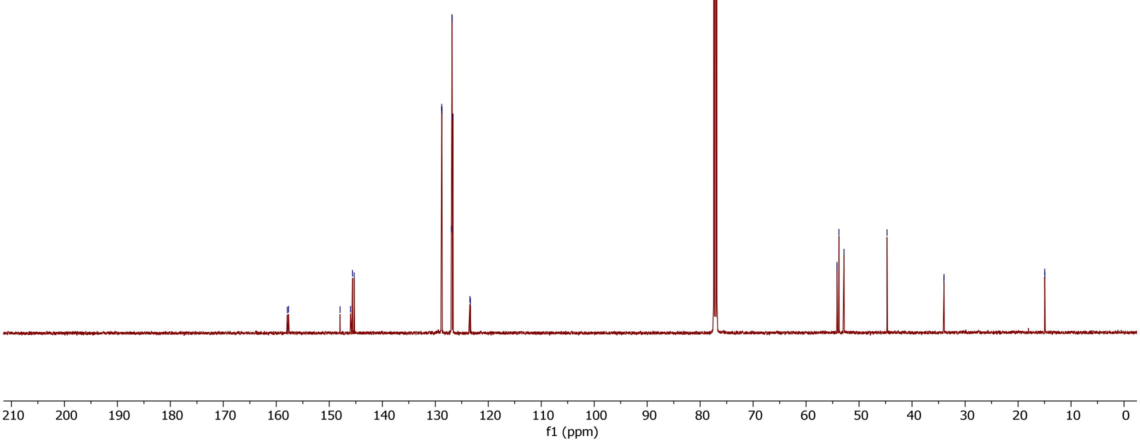

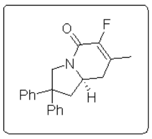

5l

^{13}C NMR (126 MHz, $CDCl_3$)

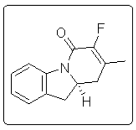

5m

^{1}H NMR (500 MHz, $CDCl_3$)

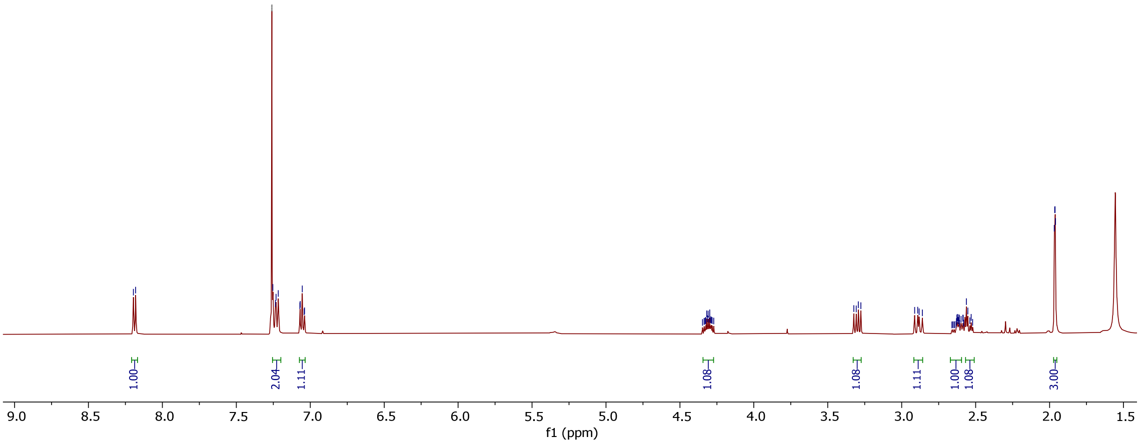

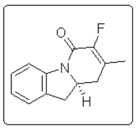

5m

^{19}F NMR (471 MHz, $CDCl_3$)

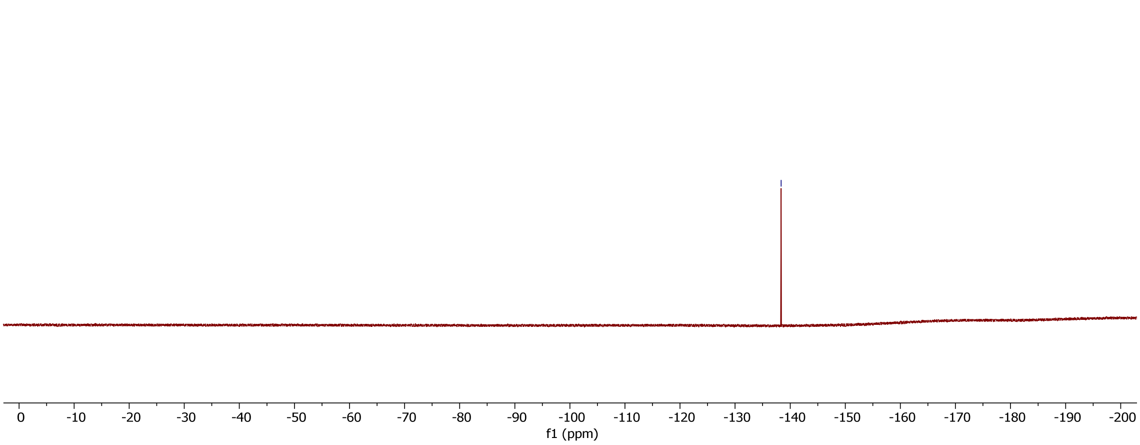

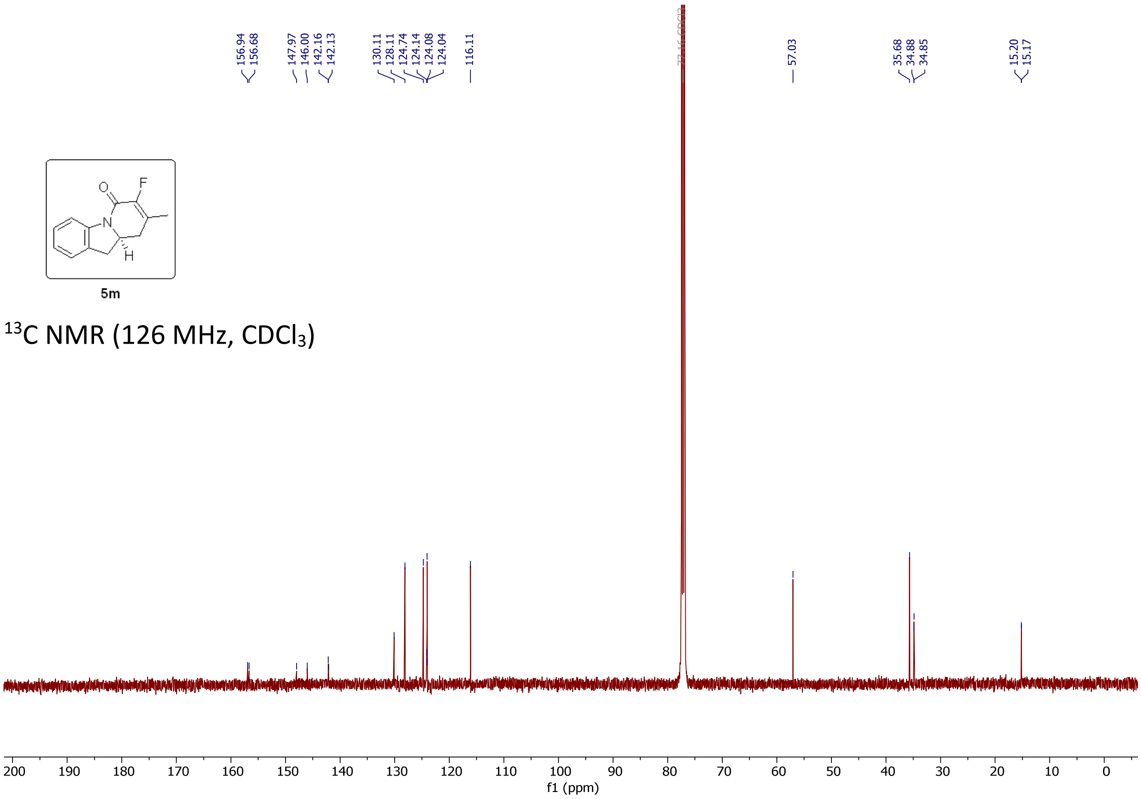

156.94
156.68
147.97
146.00
142.16
142.13
130.11
128.11
124.74
124.14
124.08
124.04
116.11
77.16 CDCl3
57.03
35.68
34.88
34.85
15.20
15.17
O
F
N
H
5m
^{13}C NMR (126 MHz, CDCl$_3$)
200 190 180 170 160 150 140 130 120 110 100 90 80 70 60 50 40 30 20 10 0
f1 (ppm)

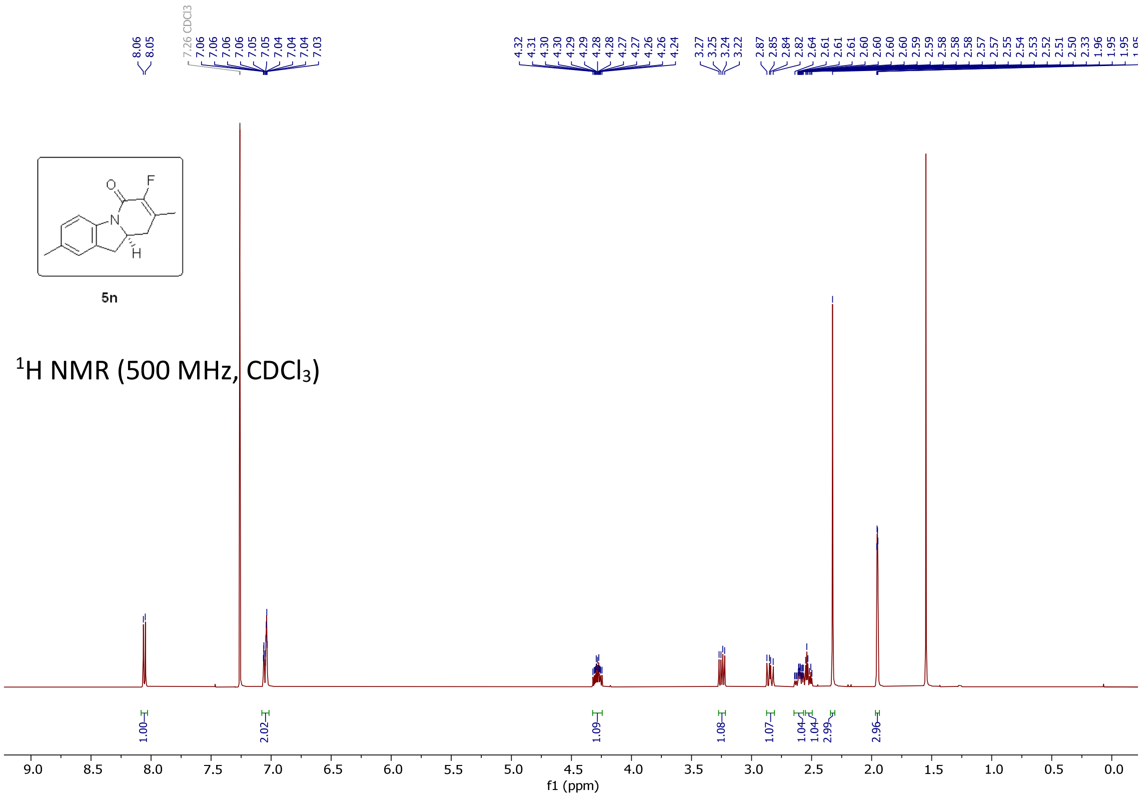

8.06
8.05
7.26 CDCl3
7.06 7.06 7.06 7.06 7.05 7.05 7.04 7.04 7.04 7.03
4.32 4.31 4.30 4.30 4.29 4.29 4.28 4.28 4.27 4.27 4.26 4.26 4.24
3.27 3.25 3.24 3.22
2.87 2.85 2.84 2.82 2.64 2.61 2.61 2.61 2.60 2.60 2.60 2.60 2.59 2.59 2.58 2.58 2.58 2.57 2.57 2.55 2.54 2.53 2.52 2.51 2.50 2.33 1.96 1.95
O
F
N
H
5n
^{1}H NMR (500 MHz, CDCl$_3$)
1.00
2.02
1.09
1.08
1.07
1.04
1.04
2.99
2.96
9.0 8.5 8.0 7.5 7.0 6.5 6.0 5.5 5.0 4.5 4.0 3.5 3.0 2.5 2.0 1.5 1.0 0.5 0.0
f1 (ppm)

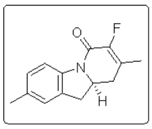

5n

^{19}F NMR (471 MHz, $CDCl_3$)

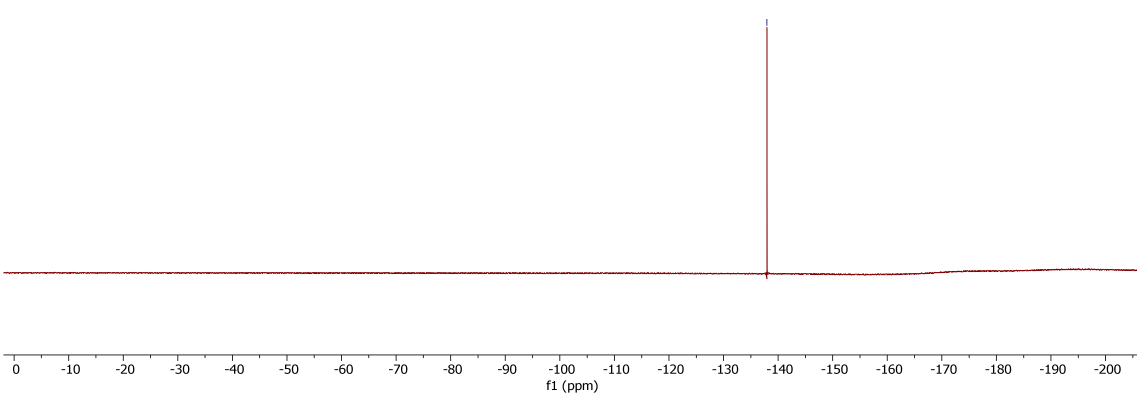

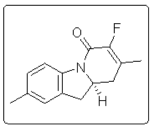

5n

^{13}C NMR (126 MHz, $CDCl_3$)

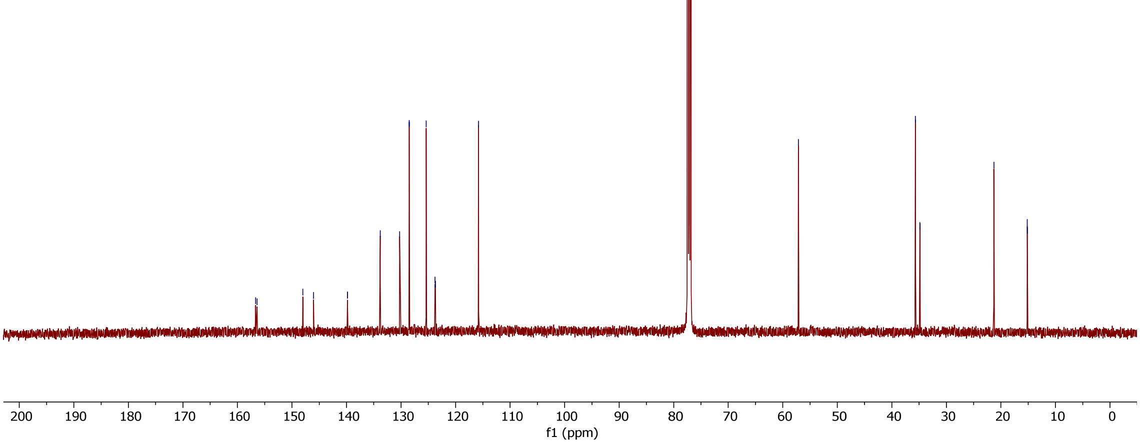

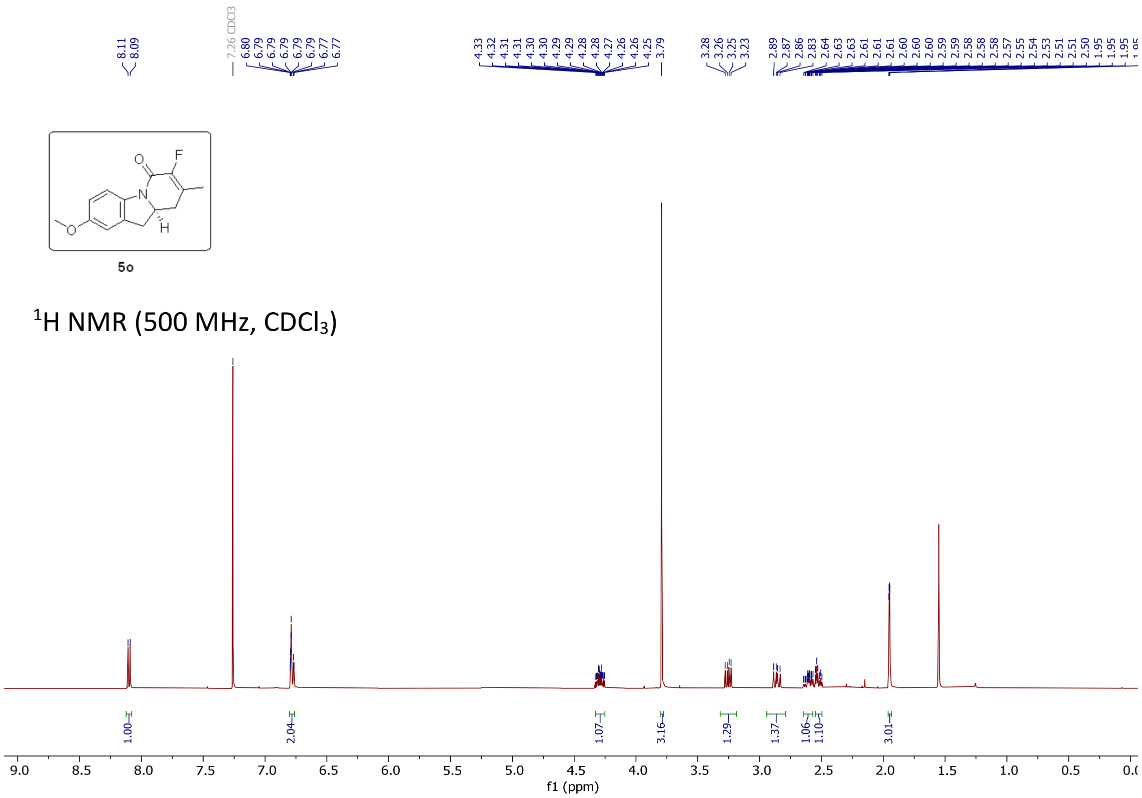
5o
^{1}H NMR (500 MHz, CDCl$_3$)
7.26 CDCl3
f1 (ppm)

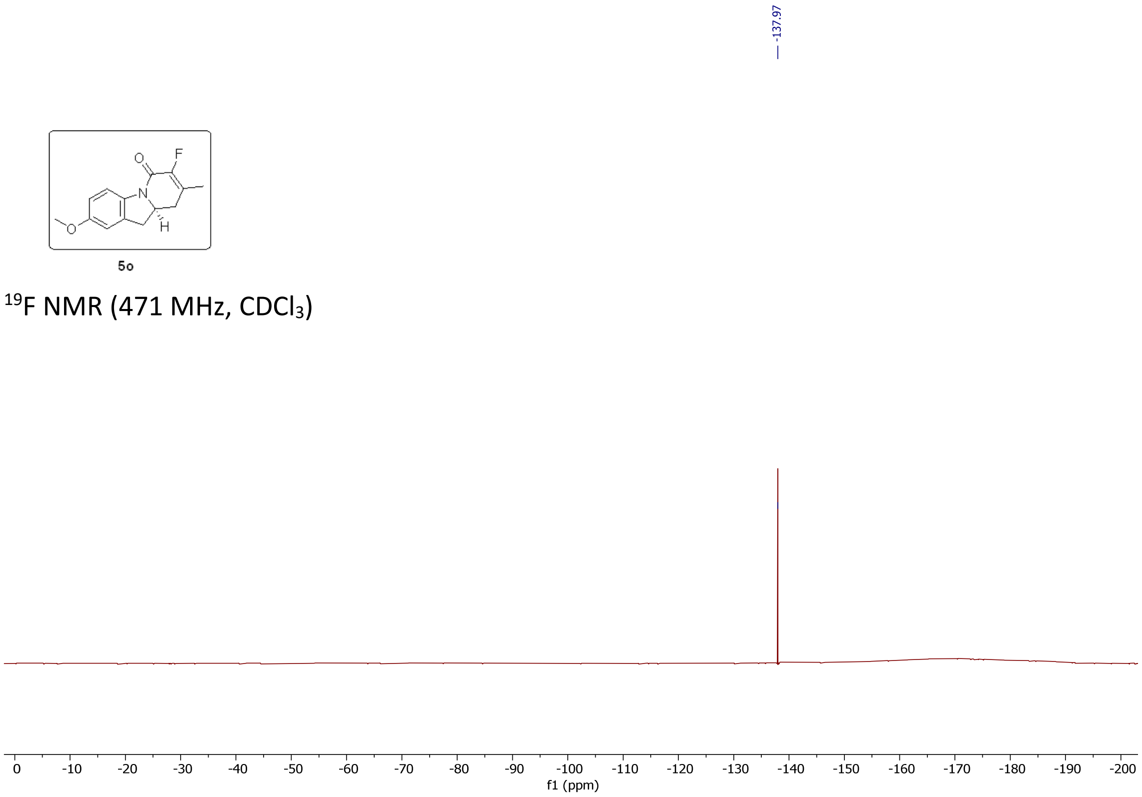
-137.97
5o
^{19}F NMR (471 MHz, CDCl$_3$)
f1 (ppm)

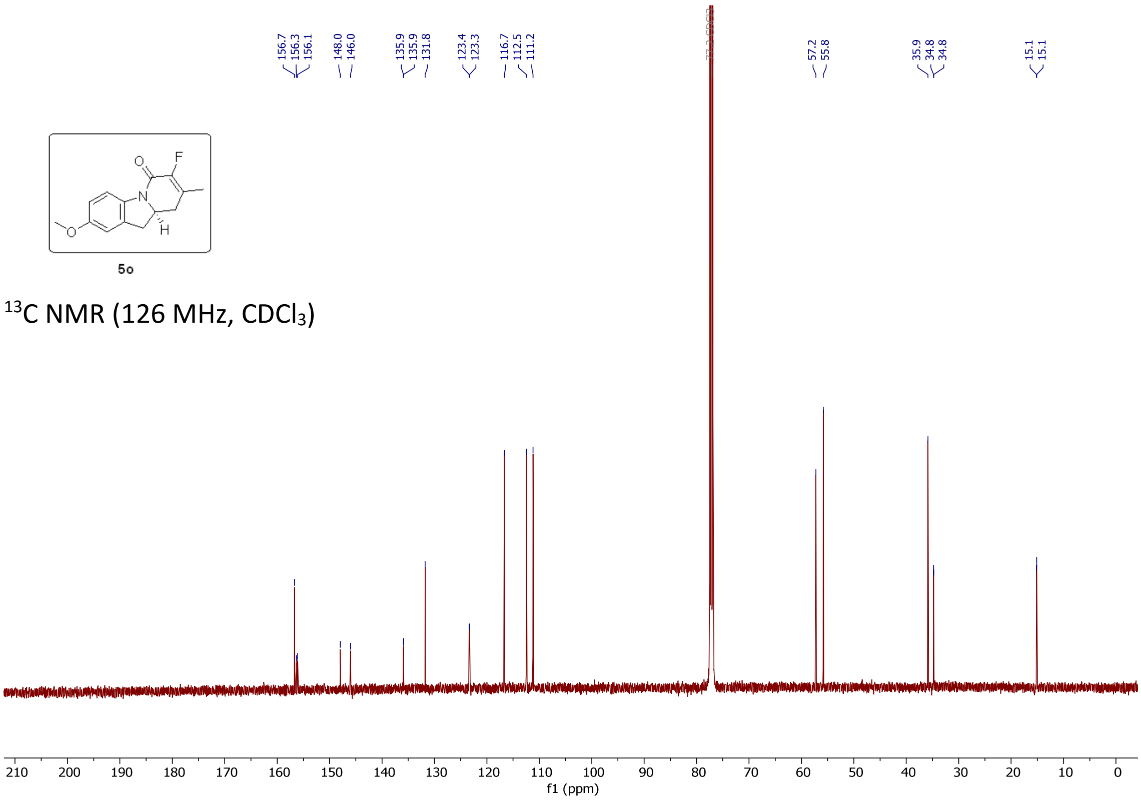
5o
^{13}C NMR (126 MHz, CDCl$_3$)
f1 (ppm)

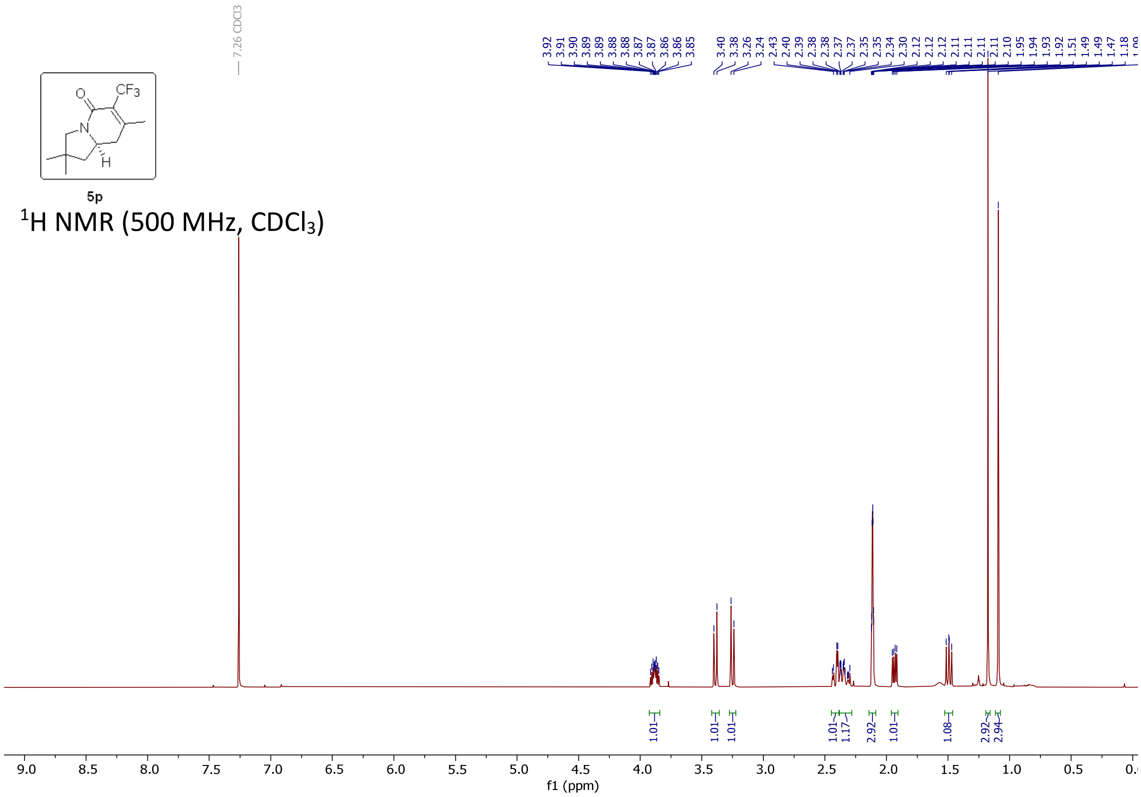
5p
^{1}H NMR (500 MHz, CDCl$_3$)
f1 (ppm)

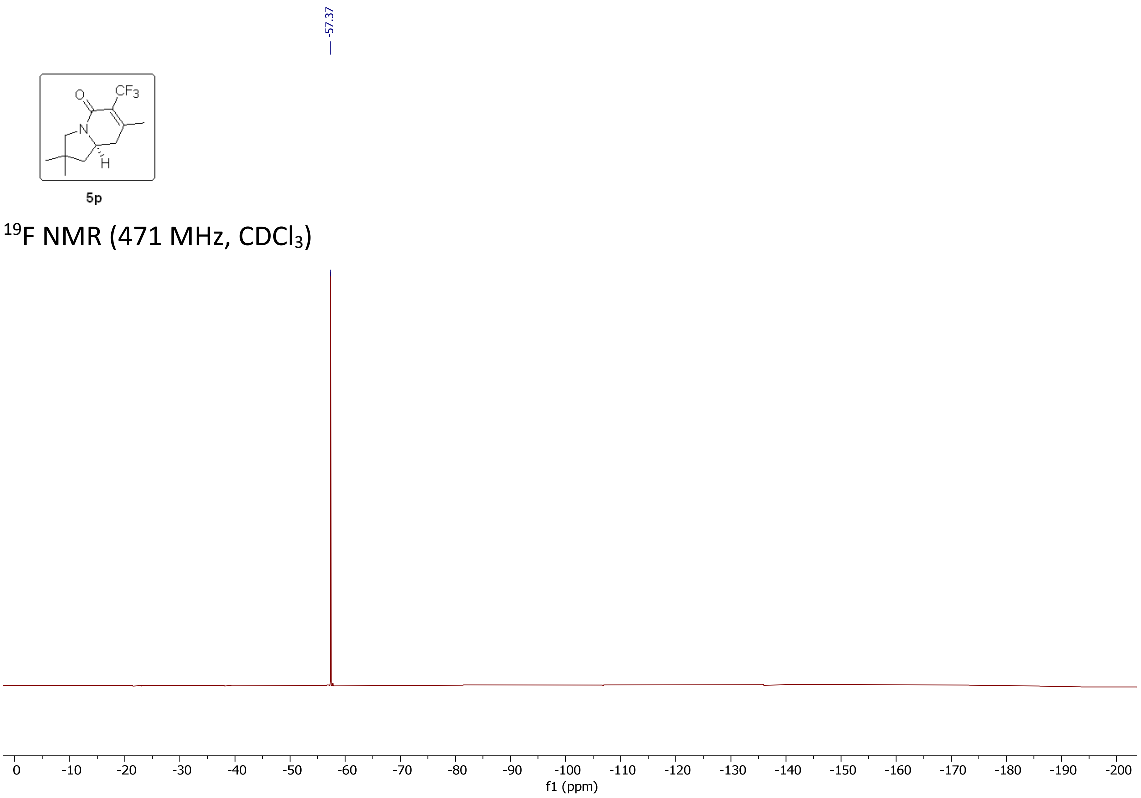

^{19}F NMR (471 MHz, $CDCl_3$)

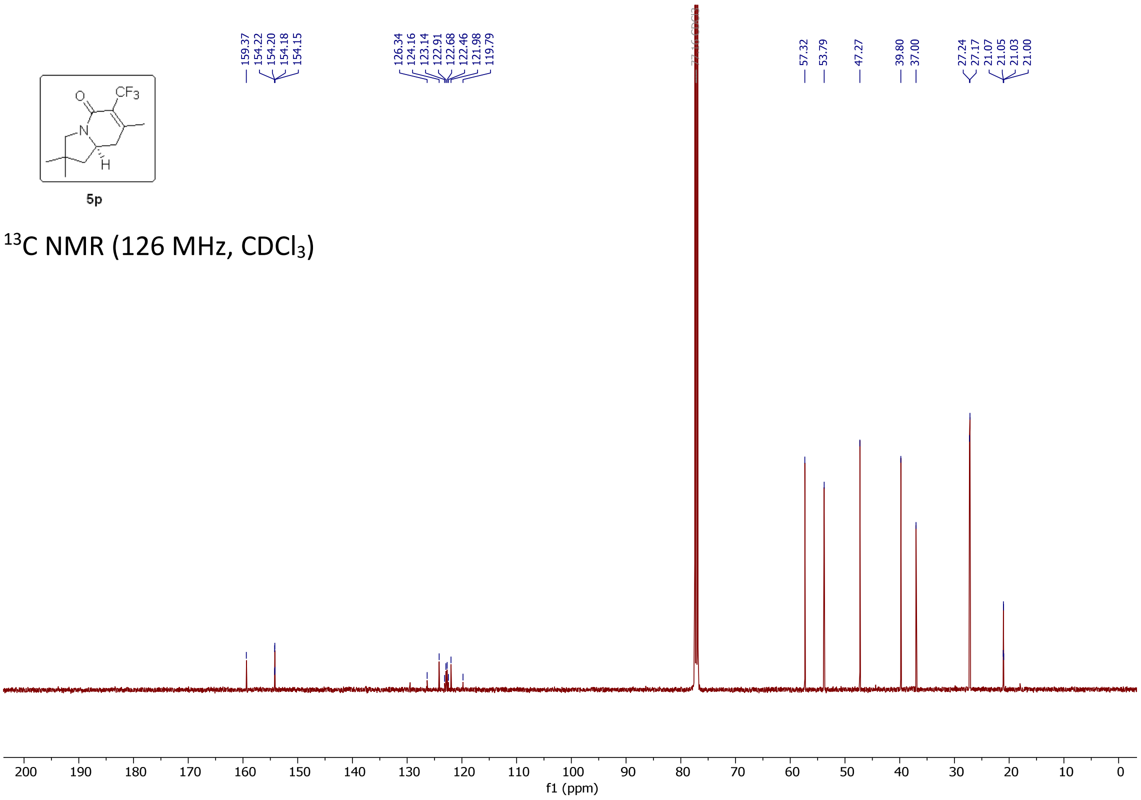

^{13}C NMR (126 MHz, $CDCl_3$)

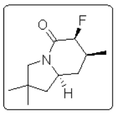

7

^{1}H NMR (500 MHz, $CDCl_3$)

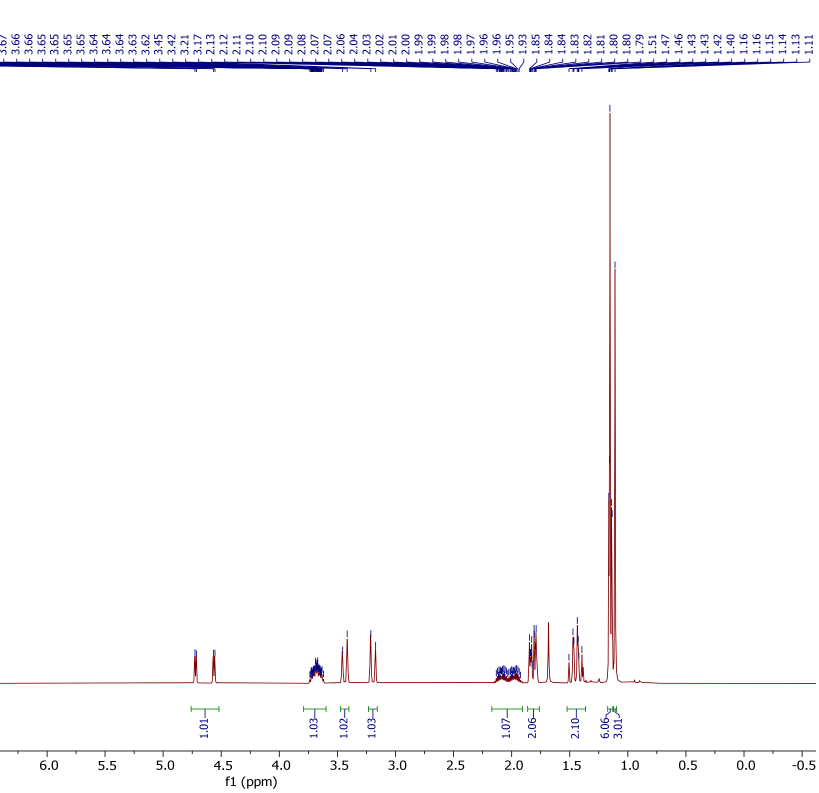

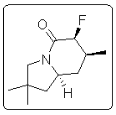

7

^{19}F NMR (471 MHz, $CDCl_3$)

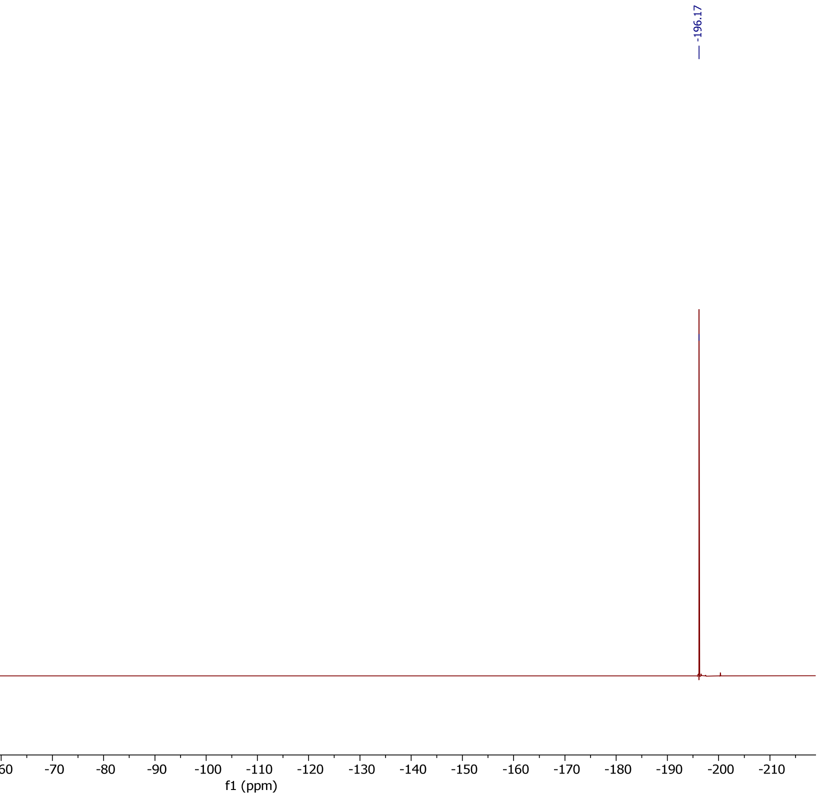

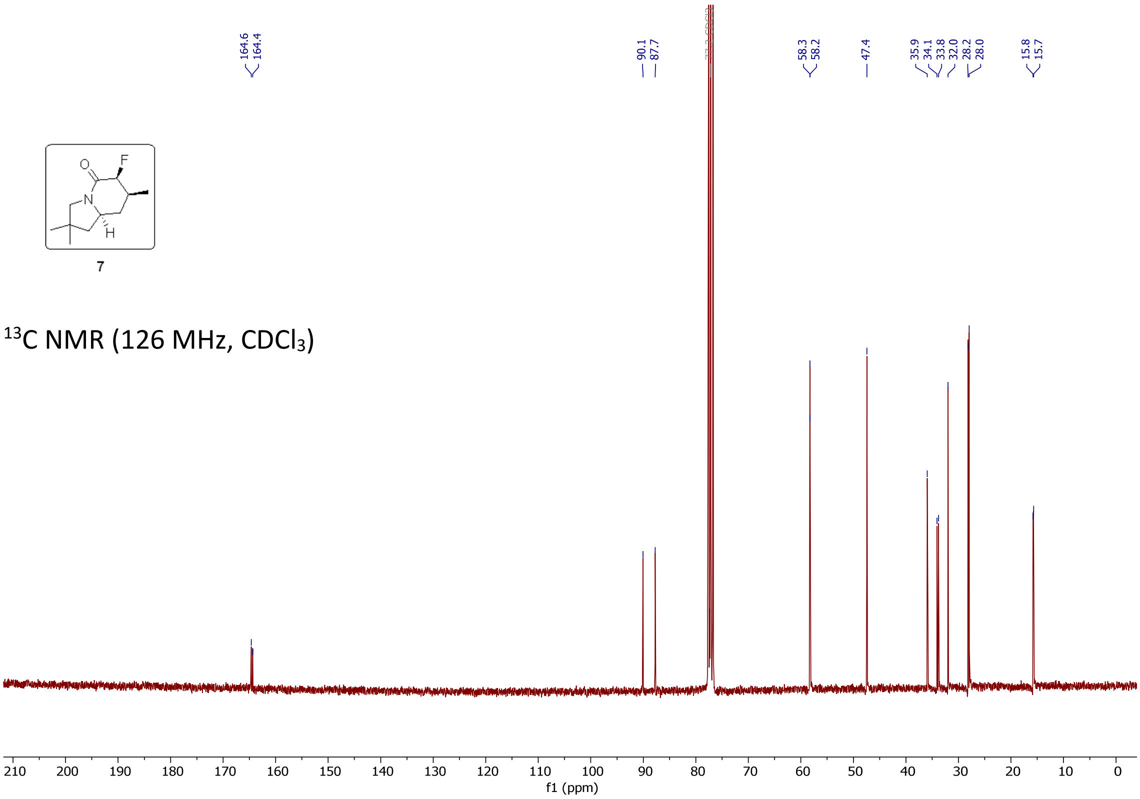

7

^{13}C NMR (126 MHz, $CDCl_3$)
